# Experimental and Theoretical Study of Zirconocene-Catalyzed Oligomerization of 1-Octene

**DOI:** 10.3390/polym12071590

**Published:** 2020-07-17

**Authors:** Ilya Nifant’ev, Alexander Vinogradov, Alexey Vinogradov, Stanislav Karchevsky, Pavel Ivchenko

**Affiliations:** 1 A.V. Topchiev Institute of Petrochemical Synthesis RAS, 29 Leninsky Pr., 119991 Moscow, Russia; amvvin@mail.ru (A.V.); vinasora@gmail.com (A.V.); phpasha1@yandex.ru (P.I.); 2 Chemistry Department, M.V. Lomonosov Moscow State University, 1 Leninskie Gory Str., Building 3, 119991 Moscow, Russia; 3 Joint-Stock Company “Institute of Petroleum Refining and Petrochemistry”, 12 Iniciativnaya Str., 450065 Ufa, Republic of Bashkortostan, Russia; st_karchevsky@mail.ru

**Keywords:** coordination polymerization, density functional theory, methylenealkanes, α-olefins, oligomerization, zirconocenes

## Abstract

Zirconocene-catalyzed coordination oligomerization of higher α-olefins is of theoretical and practical interest. In this paper, we present the results of experimental and theoretical study of α-olefin oligomerization, catalyzed by (η^5^-C_5_H_5_)]_2_ZrX_2_ **1**/**1′** and O[SiMe_2_(η^5^-C_5_H_4_)]_2_ZrX_2_ **2**/**2′** (X = Cl, Me) with the activation by modified methylalymoxane MMAO-12 or by perfluoroalkyl borate [PhNMe_2_H][B(C_6_F_5_)_4_] (NB^F^) in the presence and in the absence of organoaluminium compounds, Al(CH_2_CHMe_2_)_3_ (TIBA) and/or Et_2_AlCl. Under the conditions providing a conventional mononuclear reaction mechanism, **1′** catalyzed dimerization with low selectivity, while **2′** initiated the formation of oligomers in equal mass ratio. The presence of TIBA and especially Et_2_AlCl resulted in an increase of the selectivity of dimerization. Quantum chemical simulations of the main and side processes performed at the M-06x/ DGDZVP level of the density functional theory (DFT) allowed to explain experimental results involving traditional mononuclear and novel Zr-Al_1_ and Zr-Al_2_ mechanistic concepts.

## 1. Introduction

**Scheme 1:**
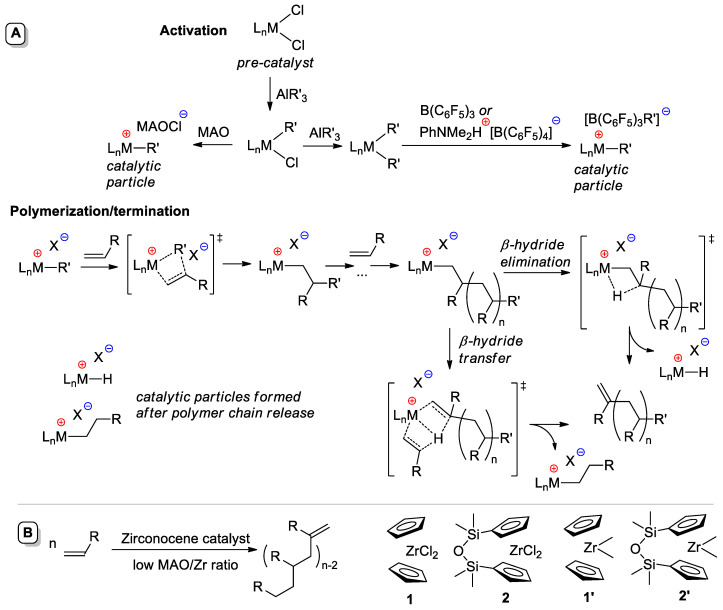
(**A**) Cossee-Arlman polymerization mechanism. (**B**) α-Olefin dimerization, pre-catalysts of selective α-olefin dimerization (**1**, **2**) and pre-catalysts studied in this work (**1′**, **2′**).

Zirconocenes hold a significant position among single-site catalysts of α-olefin polymerization due to high catalytic activity, excellent copolymerization homogeneity, and wide boundaries of regio- and sterecontrol [1,2,3,4,5,6]. Even in recent years, many of the studies of the reaction mechanisms [7,8,9,10] and structure–activity relationships [11,12,13,14,15,16] for these catalysts were performed using a generally accepted cationic concept (Scheme 1A) based on the fundamental research of Cossee and Arlman [17,18,19,20,21,22,23,24,25,26].

It is known that zirconocene dichloride (η^5^-C_5_H_5_)_2_ZrCl_2_ (**1**, Scheme 1B) [27,28,29,30], disubstituted zirconocenes (η^5^-RC_5_H_4_)_2_ZrCl_2_ [31,32,33,34,35], *ansa*-zirconocenes Z(η^5^-C_5_H_4_)_2_ZrCl_2_ (where Z is one- or two-membered bridge between cylopentadienyl rings) [36,37], and thiapentalene zirconium complex [38] catalyze the oligomerization of α-olefins RCH=CH_2_ with a formation of the mixtures of methylenealkanes and higher oligomers (Scheme 1B). For **1**, this reaction results in selective formation of methylenealkanes (*DP_n_* = 2, Scheme 1B) in the presence of a minor excess of methylaluminoxane (MAO). Christoffers and Bergman [28,29] demonstrated that chloride is an indispensable component of the catalyst system that provides the high selectivity of dimerization. Their observation was confirmed experimentally by an increase of the selectivity of zirconocene/MAO catalyzed dimerization in the presence of R_2_AlCl [30,39]. SiOSi-bridged zirconium complex **2** (Scheme 1B) demonstrated the best performance in terms of catalytic activity and α-olefin dimer yield [36,40].

In the early 1990s, zirconocene-catalyzed oligomerization of higher α-olefins had been considered as a particular case of single-site polymerization, keeping in mind that, at higher MAO/Zr ratios, **1** and its analogs catalyzed the formation of lower oligomers and matched the Flory distribution in full compliance with traditional cationic mononuclear coordination-insertion concept presented in Scheme 1A [10,38,41]. However, in recent years, the coordination oligomerization of higher α-olefins has attracted the major attention of researchers because of the importance of this process for the synthesis of hydrogenated oligomers of α-olefins (in most cases, 1-decene) which are high quality polyolefin oil basestocks (PAOs). Such oligomerization provides the high structural homogeneity of the oligomers, but requires abandoning the high Al/Zr ratios in a catalytic system to suppress monomer transfer to Al [38]. Thus, the task of a more thorough investigation of the α-olefin oligomerization in the presence of minimal amounts of organoaluminium compounds became relevant again.

In this paper, we report the results of the experimental study of 1-octene oligomerization using zirconocenes **1** and **2** and their dimethyl derivatives **1′** and **2′** (Scheme 1B), activated by modified methylalymoxane MMAO-12 and perfluoroalkyl borate [PhNMe_2_H][B(C_6_F_5_)_4_] (NB^F^) with and without organoaluminium components and molecular hydrogen. The express purpose of the study was to compare the impact of different activators (MMAO-12 or NB^F^), trialkylaluminium (TIBA), R_2_AlCl, and H_2_ on the reaction rate, oligomer distribution, and molecular structure of the reaction products. In addition, we tried to interpret and visualize the results of the oligomerization experiments using the density functional theory (DFT) modeling of the possible reaction pathways to extend the mechanistic insights of single-site polymerization mechanism within and beyond the commonly accepted Cossee–Arlman coordination–insertion mechanism.

## 2. Materials and Methods

### 2.1. General Experimentsl Remarks

TIBA (1 M solution in hexane, Merck, Kenilworth, NJ, USA), MMAO-12 (1.52 M solution in toluene, Merck, NJ, USA), (η^5^-C_5_H_5_)_2_ZrCl_2_ (Merck, NJ, USA), [PhNMe_2_H][B(C_6_F_5_)_4_] (Fischer Scientific, Waltham, MA, USA), *d*-solvents C_6_D_6_ and CDCl_3_ (99.8% ^2^H, Cambridge Isotope Laboratories, Inc., Tewksbury, MA, USA) were used as purchased. Further, 1-Octene (Merck, NJ, USA) was stored over Na wire and distilled under argon. SiOSi-bridged zirconocene dichloro complex **2** [42] and (η^5^-C_5_H_5_)_2_ZrMe_2_ **1′** [43] (Scheme 1) were synthesized according to previously reported procedures.

The ^1^H NMR spectra were recorded on a Bruker AVANCE 400 spectrometer (400 MHz, Bruker, Billerica, MA, USA) at 20 °C. The chemical shifts are reported in ppm relative to the solvent residual peaks (7.26 ppm). Elemental analysis was performed on a Perkin Elmer Series II CHNS/O Analyzer 2400 (Perkin Elmer, Inc., Waltham, MA, USA). The distribution of oligomers produced in zirconocene-catalyzed reactions was measured by the gas chromatography (GC) method. GC analysis was carried out with a KRISTALL-2000M gas chromatograph (Meta-chrom Ltd., Yoshkar-Ola, Russia) equipped with a SolGel-1ms (60 m × 0.25 mm × 0.25 μm) column and a flame ionization detector. Helium was used as a carrier gas at a rate of 1.364 cc/min and with a split ratio of 73.3:1. The injection temperature was 320 °C, and the column temperature was 200 °C within 5 min and then increased from 200 to 300 °C at a rate of 10 °C/min.

### 2.2. Synthesis of Zirconocene 2′

Zirconocene dichloro complex **2** (2.12 g, 5 mmol) was suspended in 50 mL of ether, cooled to −40 °C, and LiMe (11 mL of 1M solution in ether, 1 mmol) was added dropwise. The mixture was allowed to warm to the room temperature, the solution was filtered off and evaporated. The residue was recrystallized from n-hexane, the colorless crystals of **2′** were dried in vacuo. The yield was 3.13 g (82%). For C_16_H_26_OSi_2_Zr calculated C, 50.34; H, 6.86; O, 4.19. Found: C, 50.38; H, 6.89; O, 4.21. ^1^H NMR (C_6_D_6_, 20 °C) δ: 6.27 (dd, ^3^*J* = 6.06 & 6.32 Hz, 4H); 6.01 (dd, ^3^*J* = 6.06 & 6.32 Hz, 4H); 0.22 (s, 12H); −0.05 (s, 6H). ^13^C NMR (C_6_D_6_, 20 °C) δ: 117.53; 117.22; 112.97; 30.41; 0.97. For NMR spectra, see Section S2 in the Supporting Information.

### 2.3. Oligomerization Experiments

Briefly, 1-Octene (15.7 mL, 100 mmol), *n*-decane (internal standard, 1 mL), and TIBA (1 mL of 1M solution in hexane, 1 mmol) were placed in three-necked flask, prefilled with argon. Then zirconocene precatalyst (0.05 mmol) was added. After maintaining the external bath at 60 °C for 5 min, the given volume of MMAO-12 (1.2M solution in toluene) or NB^F^ (40 mg, 0.05 mmol) was added. After 2 h of stirring, the mixture was cooled, and 2 mL of saturated aqueous solution of Na_2_CO_3_ was added. The sample of the organic fraction was analyzed by NMR spectroscopy and GC.

### 2.4. DFT Calculations

The initial cartesian coordinates of the stationary points had been found by PRIRODA program (version 4.0, Moscow, Russia) [44] using the 3ζ basis. The final optimization and determination of the thermodynamic parameters for stationary points and transition states were carried out using Gaussian 09 program [45] for gas phase at 298.15 K. The M-06x functional [46] and DGDZVP basis [47,48] were used in the optimizations. As demonstrated earlier, M-06x functional is one of the most correct for calculations of the free energies in the DFT modeling of zirconocene-catalyzed reactions [49]. Transition states were found by energy scanning with sequential changing of key geometric parameters with a step of 0.01 Å followed by Berny optimization. DFT calculations data are provided in the Supporting Information.

## 3. Results and Discussion

### 3.1. Oligomerization Experiments and End-Group Analysis

**Figure 1:**
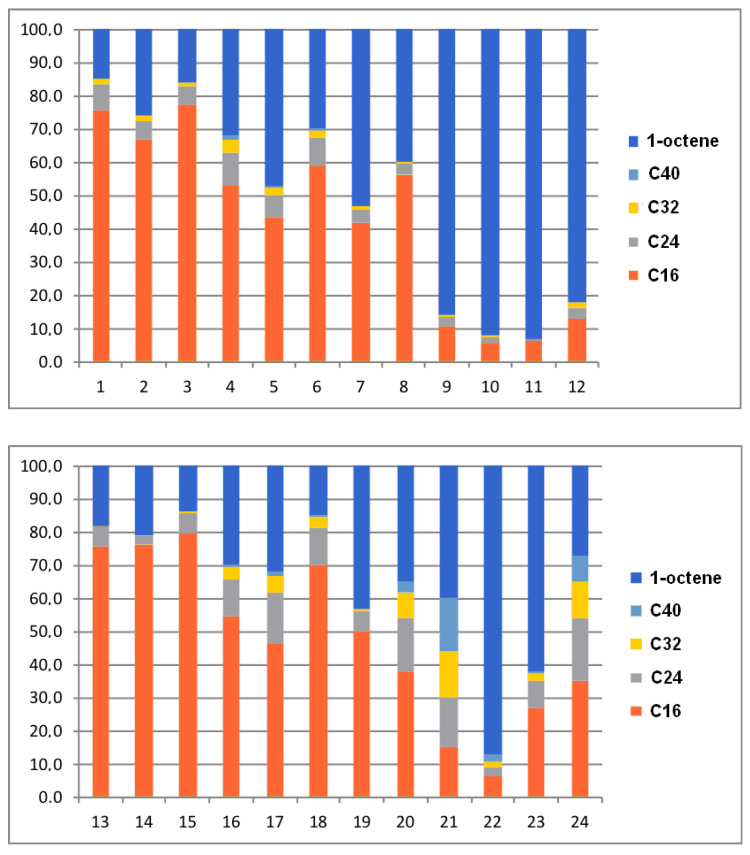
The weight distribution of the reaction products and unreacted 1-octene in bulk oligomerization, catalyzed by **1**/**1′** (top) and **2**/**2′** (bottom). Run numbers are given below the x-axes.

To compare the catalytic activities and mechanisms of the chain termination events, we studied four types of pre-catalysts, namely, zirconocene dichloride **1**, its dimethyl derivative **1′**, SiOSi-bridged dichloro **2,** and dimethyl **2′** *ansa*-complexes (Scheme 1B). MMAO-12 and NB^F^ were used as activators. TIBA was added as an additional activator in most of the experiments (Table 1). The reactions were carried out in liquid 1-octene media (see Section 2.3 for experimental details), and the low values of the degree of oligomerization *DP_n_* simplified the end-group identification by the analysis of ^1^H NMR spectra of the reaction mixtures and oligomer fractions. The basic principles of the similar end-group analysis were summarized in our recent publication [50], based on previous research of α-olefin polymerization [51,52,53,54,55,56]. NMR spectra were accompanied by the results of GC analysis and compared with the recent results of Kissin [10]. The weight distribution of the reaction products taking into account unreacted 1-octene is presented in Figure 1.

The first experiment (Table 1, Run 1) was performed using 1-octene after activation of zirconocene dichloride **1** by 20 eq. TIBA and 10 eq. MMAO-12. The results of the experiment were in good agreement with the results of Christoffers and Bergman [28,29], and previously reported data for oligomerization of 1-hexene in the presence of **1**, activated by TIBA and 10 eq. MAO [36,40]. The main product of the reaction was 7-methylenepentadecane, and the C_16_ fraction contained ~3% each of the products with –CH = CH– and >C = CH– structural fragments. The first fragment represents linear hexadecenes formed by the secondary 1-octene insertion to Cp_2_Zr–*n*–C_8_H_17_ species followed by β-hydride elimination or β-hydride transfer. The formation of the products with >C = CH– fragment can be attributed to intermediate formation of allyl intermediates [55] or to 1,2-rearrangement of the primary alkyl intermediate proposed by Crowther et al. [51,56]. In view of the fact that this reaction was carried out in the absence of molecular hydrogen, the latter explanation appeared to be more realistic, and we evaluated its feasibility by the DFT calculations (see Section 3.2.1).

In the presence of Et_2_AlCl (Table 1, Run 2), the selectivity of the formation of 7-methylenepentadecane increased, with low decrease in catalytic activity. It is of critical importance that the formation of the isomers of 7-methylenepentadecane containing >C = CH– fragment was significantly reduced. In the presence of the molecular hydrogen (Table 1, Run 3), we detected an increase of the selectivity of dimerization in comparison with Run 1 (Table 1), and the presence of the side products with >C = CH– fragment was also negligible. When 200 eq. MMAO-12 was used (Table 1, Run 4), the selectivity of the dimer formation was lower, and the content of the products with –CH = CH– fragments was slightly increased.

Replacement of MMAO-12 by NB^F^ in experiments with zirconocene **1** pre-treated by TIBA (Table 1, Runs 5 and 6) resulted in significant reduction of the catalytic activity. In terms of the selectivity of dimer formation, the results of these experiments were closely related with the results of 1-octene oligomerization in the presence of 200 eq. MMAO-12, but the formation of side products was lower. Therefore, the use of NB^F^ instead of MAO seems feasible in terms of the yield of structurally uniform α-olefin oligomers. Note that it is this activation method that was used in prospective technologies of the production of PAOs [57,58,59,60,61,62,63,64].

In experiments with dimethyl derivative **1′** activated by 10 eq. MMAO-12 in the absence and in the presence of Et_2_AlCl (Table 1, Runs 7 and 8), the catalytic activity was lower, but the selectivity of dimerization was substantially higher in comparison with Runs 1 and 2. The result of Rin 7 is somewhat inconsistent with the prior assertion that the presence of chlorides is essential to provide the selectivity of α-olefin dimerization [28,29,36,40].

Activation of **1′** by NB^F^ (Table 1, Run 9) resulted in a slow formation of the mixture of oligomers. The addition of TIBA (Table 1, Run 10) or Et_2_AlCl (Table 1, Run 11) slowed the reaction rate. Even so, the presence of Et_2_AlCl provided the high selectivity of the dimer formation without any side reactions! The presence of the molecular hydrogen (Table 1, Run 12) in experiment with NB^F^ activation of **1′** did not affect the product distribution, but slightly accelerated the reaction. However, it must be noted that, in the presence of H_2_ the signals of >C = CH– were detected (these signals were fully absent for Runs 10 and 11).

Being activated by 20 eq. TIBA and 10 eq. MMAO-12, zirconocene **2** catalyzed the selective dimerization of 1-octene (Table 1, Run 13), and the addition of 1 eq. Et_2_AlCl increased the selectivity of dimerization (Table 1, Run 14) in accordance with previously published results [36]. The reaction mixtures contained minimal amounts of the product with internal –CH = CH– double bond, >C = CH– fragments were absent. The presence of the molecular hydrogen (Table 1, Run 15) resulted in modest acceleration of the reaction, with the appearance of the >C = CH– signals. In the presence of 200 eq. MMAO-12 (Table 1, Run 16), we detected increasing of the higher oligomer content, lower amounts of –CH = CH– products, and significant (~8%) formation of the products containing >C=CH– fragment. When **2** was activated by TIBA and NB^F^ (Table 1, Run 17), >C = CH– signals were not detected, this signal appeared in the presence of the molecular hydrogen (Table 1, Run 18).

Dimethyl derivatives **1′** and **2′** demonstrated qualitatively different behavior being activated by MMAO-12 (Table 1, Runs 7 and 19, respectively). SiOSi-bridged dimethyl complex turned out to be the catalyst of the oligomerization in contrast with **1′** that was moderately selective dimerization catalyst. The addition of Et_2_AlCl (Table 1, Run 20) led to increasing of the selectivity of dimerization. The fundamental difference in catalytic behavior between **1′** and **2′** was detected in experiments with NB^F^ activator: **2′** in the presence of 1 eq. of perfuoroaryl borate (Table 1, Run 21) catalyzed the formation of oligomers in parity by weight. The addition of 20 eq. TIBA (Table 1, Run 22) resulted in a shift to the preference of the dimer formation with a slowing down of the reaction, and the addition of Et_2_AlCl (Table 1, Run 23) slightly increased both reaction rate and dimerization selectivity. The influence of the molecular hydrogen (Table 1, Run 24) was insignificant.

### 3.2. DFT Modeling of the Reaction Pathways for (η^5^-C_5_H_5_)_2_Zr-Based Catalytic Species

Despite the fact that complex **1** was historically the first pre-catalyst used in zirconocene polymerization, only a few works devoted to the oligomerization of higher α-olefins in the presence of **1**, activated by MAO [27,28,29,30,31,36,37,38,39,40,65,66,67] or (perfluorophenyl)borates [68], have been published to date. The recent investigation of Kissin [10] comprised a comprehensive analysis of the products of 1-hexene oligomerization, catalyzed by **1**/MAO at high Zr:Al ratios (~1:200). The use of a large excess of MAO in the experiments of Kissin obviously resulted in the formation of significant amounts of the C_7_, C_13,_ and C_19_ products of 1-hexene oligomerization, initiated by Cp_2_ZrMe^+^ species. In our experiments, the percentage of such products was expected as null and void. In addition, when TIBA was used as an organoaluminium activator, C_12_ and C_20_ reaction products were not detected in the reaction mixtures. In experiments with dimethyl derivatives **1′** and **2′**, the contribution of C_9_, C_17_, and C_25_ reaction products was also negligible. All the above allowed us to stay focused on the processes involving the hydride catalytic species formed after the activation of zirconocenes, and C_8_, C_16_, and C_24_ derivatives that are the products of the insertion of 1-octene. In this section, we discuss the reaction mechanism with the involvement of the results of the modeling using the density functional theory (DFT) at M06-2X/DGDZVP [46,47,48,49] level. Optimized geometries, cartesian coordinates, and energy parameters for all stationary points and transition states mentioned below are presented in the Supporting Information.

#### 3.2.1. Mononuclear Reaction Mechanism

**Scheme 2:**
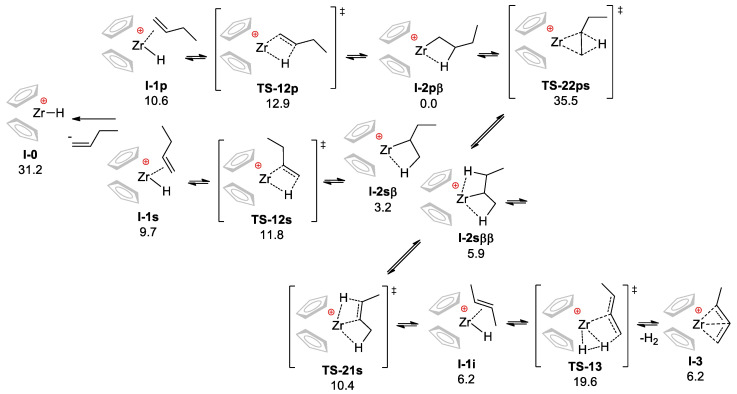
The first group of the transformations of zirconocene butyl and isobutyl cationic complexes: the reactions in the absence of the second molecule of 1-butene. The values of calculated free energies (kcal/mol) relative to **I-2pβ** are given.

In our calculations, we used 1-butene as a model olefin. In the framework of the traditional mechanism of zirconocene-catalyzed polymerization of α-olefins, catalytic species represent a Cp_2_Zr–alkyl cationic complexes. First, we optimized the different conformations of [(η^5^-C_5_H_5_)_2_Zr–*n*-Bu]^+^ and [(η^5^-C_5_H_5_)_2_Zr–*sec*-Bu]^+^ and found that β-agostic *n*-butyl complex **I-2pβ** (Scheme 2, here and below index p means “primary”) had the minimal free energy. Additional stabilization of *sec*-alkyl complexes **I-2sβ** and **I-2sββ** (here and below s—“secondary”) by hyperconjugation, proposed by Kissin [10], was not confirmed by our calculations. The free energy of primary alkyl α-agostic **I-2pα** and γ-agostic **I-2pγ** complexes were 10 and 4 kcal/mol higher than **I-2sβ**, which rules out these complexes from further consideration.

**Figure 2:**
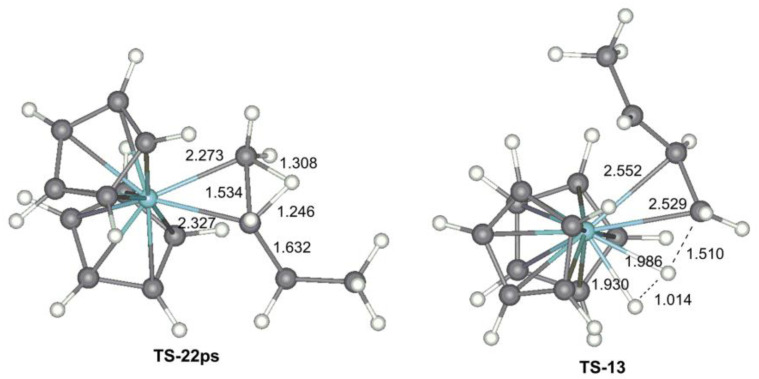
Optimized geometries of the transition states **TS-22ps** and **TS-13**.

The β-agostic alkyl complexes can be involved in different processes (Scheme 2). The first reaction is β-hydride elimination resulting in zirconocene hydride cation with π-coordinated olefin molecule. Depending on the type of the complex (primary or secondary) and position of β-agostic hydrogen in butyl fragment, this reaction may result in different products via transition states that are also diverse in their energy. For [(η^5^–C_5_H_5_)_2_Zr–*sec*-Bu]^+^, β-hydride elimination with a formation of (*E*)-2-butene complex **I-1i** was found to be preferable in comparison with the formation of 1-butene complex **I-1s**. The activation barrier for β-hydride elimination in **I-2pβ** with the formation of 1-butene complex **I-1p** was the highest (Scheme 2). In view of the reversibility of the processes presented in Scheme 2, the results of calculations contradicted the common concept that postulates the preference of the olefin insertion with a formation of the primary alkyl complex. However, this claim is true for the insertion of α-olefin molecule in Zr–alkyl complexes (see below), and the reaction seems to be more complex at the stage of the insertion of the first olefin molecule at Zr–H bond. First, the formation of **I-1p** and **I-1s** via β-hydride elimination is energetically unfavorable, the formation of naked hydride cation **I-0** seems impossible. This allows us to exclude **I-0**, **I-1**, and **TS-12** from the list of actual catalytic species. However, in light of the study of the subsequent processes, the possibility of intramolecular rearrangement between primary **I-2p** and secondary **I-2s** alkyl complexes should be considered. We optimized the structure of the corresponding transition state **TS-22ps** (Scheme 2, Figure 2, left) and found that ΔG^≠^ for this reaction is 35.5 kcal/mol. Such a value is too high for freely occurrence of such rearrangement. Another possible process is a formation of allyl complex **I-3** from **I-1i** via **TS-13** (Scheme 2, Figure 2, right). The activation barrier for this reaction is only 13.4 kcal/mol. Therefore, the formation of the allyl species should not be excluded in the detailed analysis of the chemical behavior of cationic zirconocene alkyl complexes. Note that the formation of allyl complexes in the zirconocene-catalyzed polymerization of α-olefins was proven experimentally in a number of publications [55,69,70,71,72,73,74,75].

**Scheme 3:**
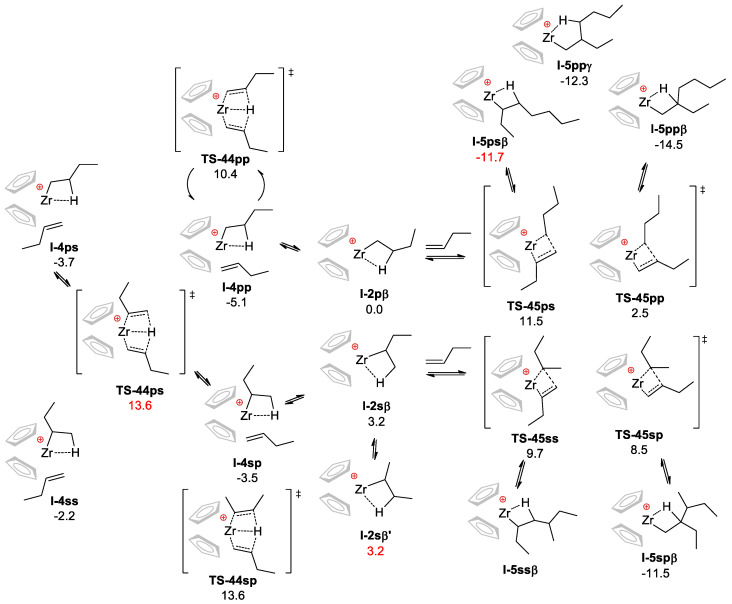
Transformations of zirconocene butyl and isobutyl cationic complexes with the participance of the second molecule of 1-butene. The values of calculated free energies (kcal/mol) relative to **I-2pβ** are given.

**Figure 3:**
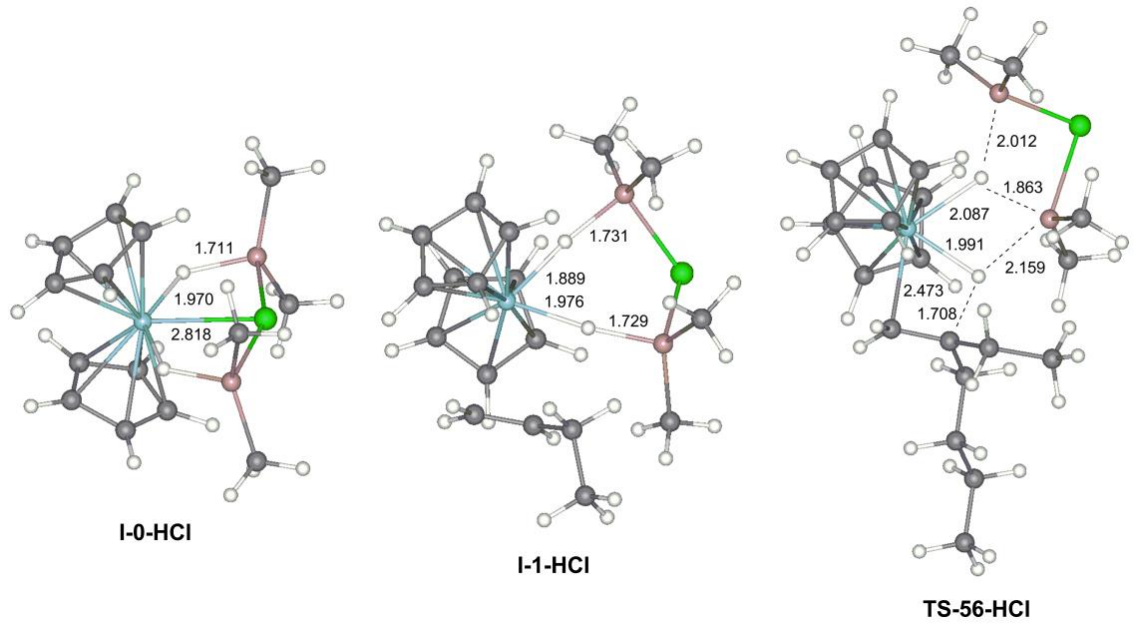
Optimized geometries of the stationary points **I-0-HCl** (left), **I-1-HCl** (center) and transition state **TS-56-HCl** (right).

Given the instability of cationic hydride complexes in the framework of a mononuclear mechanism, we decided to focus on the processes involving alkyl zirconocene cations and 1-butene molecule. At the first stage, π-complexes of different structures **I-4** can be formed (Scheme 3). This process is exergonic, the relative energies of π-complexes are dependent on the nature of Zr–alkyl (*n*-butyl or *sec*-butyl) and orientation of the coordinated 1-butene molecule. Optimized geometries and energy parameters of these structures are presented in the Supporting Information, the most stable structure **I-4pp** corresponds to a “head to head” direction of the subsequent polymerization (1,2-insertion of the monomer in primary alkyl complex) in line with the conventional reaction mechanism. The values of the relative free energies (Scheme 3) of the corresponding transition states **TS-45** (Figure 3) clearly indicate the preference of the primary insertion. The experimental estimation of the rates of primary and secondary insertions of 1-hexene made by Kissin (*k*_prim_/*k*_sec_ ~2000) [10] is in line with the results of our calculations (*k*_prim_/*k*_sec_ ~3000 based on **TS-45pp** and **TS-45ps** values under equal Arrhenius factors). Thus, the main pathway of the insertion of the second 1-butene molecule results in the formation of branched primary alkyl complex **I-5pp**.

Another possible way of the transformations of **I-4** is β-hydride transfer. The free activation energies **TS-44** (Scheme 3) for these processes were found to be substantially higher in comparison with **TS-45**, the energy of degenerate process **TS-44pp** was only 3 kcal/mol lower in comparison with **TS-44ps** resulting in the formation of π-complex of *sec*-butyl substituted zirconocene **I-4sp** (Scheme 3). Isomeric **TS-44sp** results in the formation of 2-butene π-complex **I-4pi**. This relatively small difference between **TS-44pp** and **TS-44sp** helps to explain the formation of 2-alkenes in the zirconocene-catalyzed oligomerization of α-olefins.

**Scheme 4:**
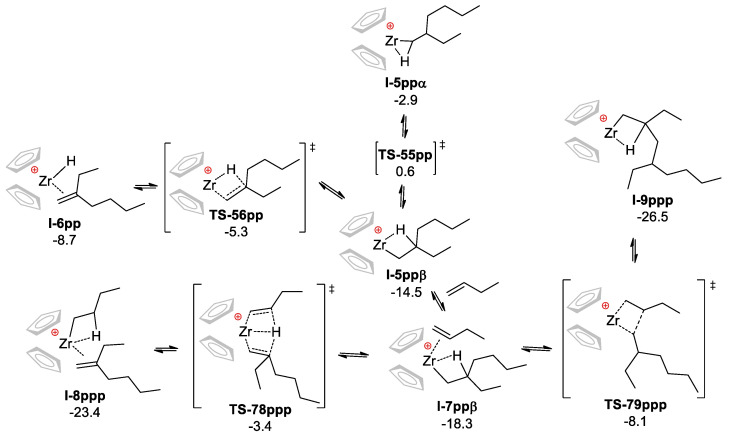
Transformations of β-agostic complex **I-5ppβ**. The values of calculated free energies (kcal/mol) relative to **I-2pβ** are given.

So, the main direction of the reaction of [(η^5^–C_5_H_5_)_2_Zr–*n*-Bu]^+^ with 1-butene was the formation of the relatively stable β-agostic complex **I-5ppβ**. This intermediate can undergo β-hydride elimination via **TS-56pp**, and coordination of the second 1-butene molecule with a formation of π-complex **I-7ppβ**. The latter can undergo β-hydride transfer via **TS-78ppp** to form a more stable π-complex **I-8ppβ** or coordination-insertion of the 1-butene molecule via transition state **TS-89ppp** with a formation of branched alkyl complex **I-9ppp** (Scheme 4). These reactions are key processes for the estimation of the selectivity of dimerization, the subject of our study. The values of the free activation energy for all three possible processes are close, therefore in the frameworks of the mononuclear mechanism the chain release (dimer formation) and chain propagation are possible. The question of the chain release mechanism remains open, however. The free energy of alkyl π-complex **I-8ppp** is 15 kcal/mol lower in comparison with hydride π-complex **I-6pp**, thus confirming the conventional wisdom that β-hydride transfer is a main pathway of the chain release for mononuclear mechanism [10].

The DFT modeling of the competition between chain release and chain propagation after the second α-olefin insertion predicted the possibility of the formation of higher oligomers. However, the experiments on 1-octene oligomerization under conditions providing a cationic mononuclear mechanism (Table 1, Runs 4, 9) demonstrated the preference of the dimerization. In terms of the analysis of the energy reaction profiles, this contradiction can be explained by the assumption of a significant difference of the Arrhenius pre-exponential factors for chain termination (TS for β-hydride elimination and β-hydride transfer are based on stable β-agostic complex) and chain propagation (TS based on high-energy α-agostic complex). Note that direct transformation of **I-5ppβ** to **I-5ppα** without the participance of the α-olefin molecule (Scheme 4) requires more than 15 kcal/mol in G scale.

#### 3.2.2. The Effect of the Formation of Zr-Al_1_ Species on the Reaction Pathway

**Scheme 5:**
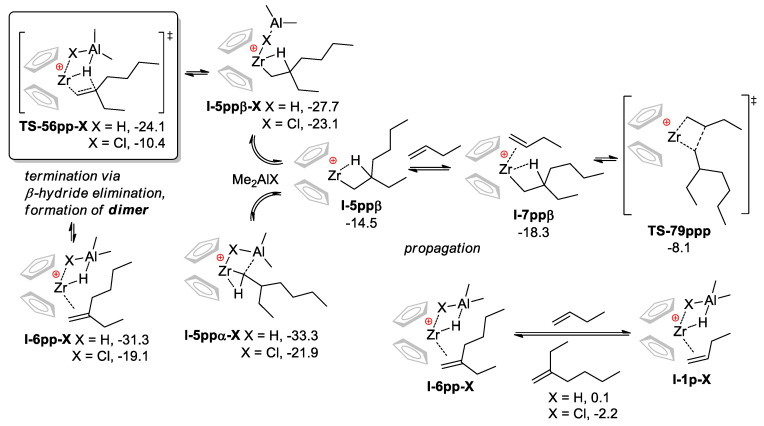
Transformations of β-agostic complex **I-5ppβ** with the participance of Me_2_AlX species. The values of calculated free energies (kcal/mol) relative to **I-2pβ** are given.

The formation of zirconium–aluminium heterometallic complexes in the reaction of zirconocenes with organoaluminium compounds is a well-known and proven fact [76,77,78,79,80,81,82,83,84,85,86,87]. In our previous works [36,40] we proposed that the increasing of the selectivity of dimerization of α-olefins can be attributed to direct participance of R_2_AlCl in the formation of the Zr-Al_1_ species prone to β-hydride elimination. The idea of such assistance was emerged by Hessen et al. for zwitterionic zirconocenes [88]. Very recently, we performed comparative DFT modeling of propylene dimerization catalyzed by (η^5^-C_5_H_5_)_2_Zr(μ-H)(μ-X)AlR_2_ (X = H, Cl, Me) [65]. The results of our calculations predicted the preference of β-hydride elimination after the second insertion of propylene molecule in these bimetallic species for X = H and Cl. Such a preference was attributed to Zr-Al cooperative effect in binuclear complexes (Scheme 5). The modeling of the reactions of higher α-olefins using propylene as a monomer needed to be clarified, and in the present work we report the results of DFT calculations for the key stage of 1-butene dimerization, namely Me_2_AlX-assisted β-hydride elimination (X = H, Cl) after the insertion of the second molecule of 1-butene. Assuming that the coordination of Me_2_AlX (**I-5ppβ-X**, **I-5ppα-X**) and 1-butene (**I-7ppβ**) are reversible and competing, we optimized the structures of the key stationary points and transition states and compared the relative free energies of these species (Scheme 5).

The significant difference between propylene [65] and 1-butene (this work) was detected. Our calculations demonstrate the extremely high stability of the α-agostic complex **I-5ppα-H** (−5.6 kcal/mol) in comparison with β-agostic **I-5ppβ-H** which is the stage preceding the transition state of the dimer formation **TS-56-H**. Therefore, “pure” hydride Zr-Al complex should not be highly active. On the other hand, for the complex formed with Me_2_AlCl, the relative stability of the intermediates **I-5** was found to be inverse: the difference in free energies between **5ppα-Cl** and **I-5ppβ-Cl** was 1.2 kcal/mol. Thus, the coordination of R_2_AlCl should promote β-hydride elimination via a low-energy intermediate and lower energy transition state, the calculated difference of the free energies of **TS-79ppp** and **TS-56-Cl** was 2.3 kcal/mol.

To evaluate the thermodynamics of the further reaction, we calculated the free energies of the π-complexes of (η^5^-C_5_H_5_)_2_Zr(μ-H)(μ-X)AlMe_2_ species with 1-butene (Scheme 5) and found that replacement of 3-methyleneheptane (**I-6pp-X**) by 1-butene (**I-1p-X**) is energetically favorable process for X = Cl (-2.2 kcal/mol).

At first sight, the simple model presented in Scheme 5 can explain the fact of the increasing of the selectivity of dimerization in the presence of R_2_AlCl species (Table 1, Runs 1–6, 8, 11). However, cationic binuclear Zr-Al_1_ species (except well-known and inert (η^5^-C_5_H_5_)_2_Zr(μ-Me)_2_AlMe_2_ [89,90,91,92,93,94,95,96]) were not detected in significant amounts in the reaction mixtures obtained by the reaction of zirconocene with AlR_3_ in the presence of MAO or perfluoroaryl borates. Moreover, NMR spectra indicated the presence of Zr-Al_2_ complexes [97,98,99]. This does not mean that cationic Zr-Al_1_ species could not participate zirconocene-catalyzed oligomerization. However, our theoretical research of the possible reaction pathways would be incomplete without the analysis of the direct participation of Zr-Al_2_ species in zircononcene-catalyzed dimerization and polymerization.

#### 3.2.3. Theoretical Analysis of the Possible Participation of Zr-Al_2_ Species

**Scheme 6:**
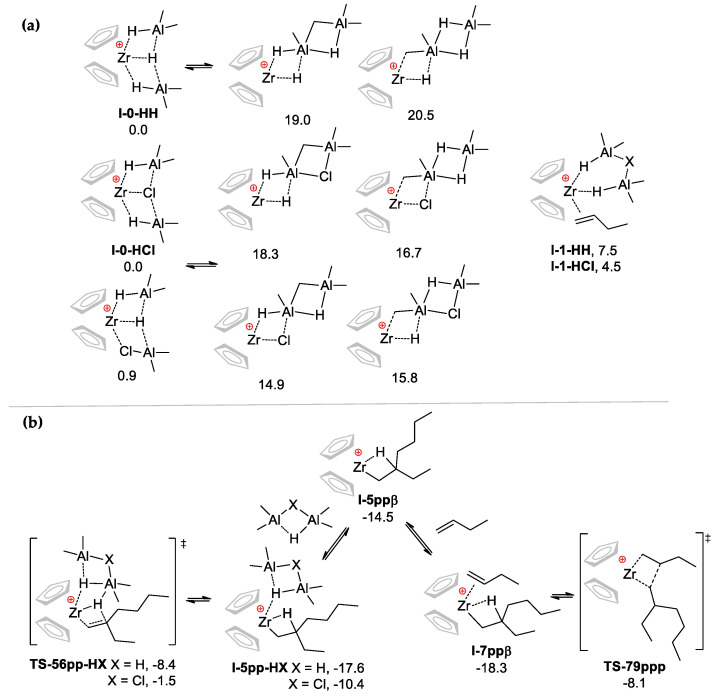
Relative free energies of Zr-Al2 species (**a**); transformations of β-agostic complex **I-5ppβ** with the participance of Me_2_Al(μ-H)(μ-X)AlMe_2_ species. The values of calculated free energies (kcal/mol) relative to **I-2pβ** are given (**b**)**.**

Trinuclear Zr-Al_2_ cationic complexes have been identified among the products of the reactions of zirconocene dichlorides with an excess of TIBA or diisobutyl aluminum hyrdide (DIBAL-H) [97,98,99]. These species (Figure 3, Scheme 6a) represent resting states of the oligomerization which were seen as unconducive to π-bonding with α-olefin molecules due to the coordination saturation of the Zr atom [97]. At the first stage of the modeling, we analyzed the relative stability of the experimentally detected stable species **I-0-HX** and hypothetically “opened” complexes that are capable of π-bonding with α-olefin. The calculated differences in free energies of the “opened” complexes and **I-0-HX** (Scheme 6a) exceeded the value of 15 kcal/mol. Coordination of 1-butene with a formation of **I-1-HX** (Scheme 6a, Figure 3) was found to be endergonic process, thus confirming high stability of **I-0-HX**.

Another DFT probe of the evaluation of the possible role of Zr-Al_2_ species was in comparison of the relative energies of the stationary points and transition states, formed by **I-5ppβ** and Me_2_Al(μ-H)(μ-X)AlMe_2_ (Scheme 6b), with the energies of dinuclear Zr-Al_1_ and mononuclear species (Scheme 5). Trinuclear transition states **TS-56pp-HX** are aesthetically beautiful (Figure 3, for animation file see the Supporting Information), and it might be tempting to assume that these TS are involved in dimerization. However, the results of our calculations demonstrate that the coordination of Me_2_Al(μ-H)(μ-X)AlMe_2_ is weak, and therefore, bearing in mind the difference of orders of magnitude between the concentrations of α-olefin and organoaluminum component, the participance of such species in the catalytic process is unlikely.

### 3.3. DFT Modeling of the Reaction Pathways for O[SiMe_2_(η^5^-C_5_H_4_)]_2_Zr-Based Catalytic Species

The key factor of the difference between (η^5^-C_5_H_5_)]_2_Zr-and O[SiMe_2_(η^5^-C_5_H_4_)]_2_Zr-based catalytic systems is the ability of the bridged oxygen to coordinate Zr atom. Additionally, the non-symmetrical structure of the O[SiMe_2_(η^5^-C_5_H_4_)]_2_Zr implies greater variability of the ligand environment, which made it difficult to perform exhaustive DFT modeling. In our research, we only optimized the molecular structures of the most significant stationary points and transition states for O[SiMe_2_(η^5^-C_5_H_4_)]_2_Zr-based species.

**Scheme 7:**
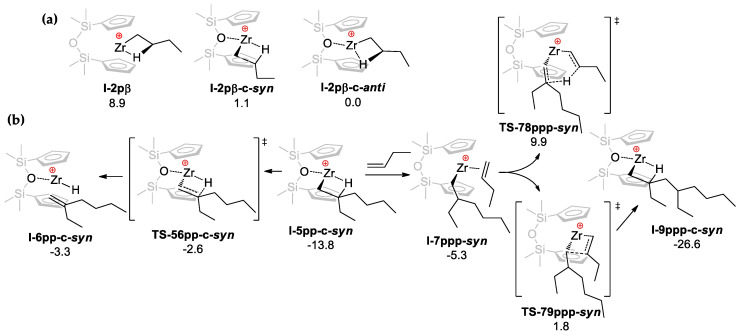
Relative free energies of O[SiMe_2_(η^5^-C_5_H_4_)]_2_Zr–Bu cationic complexes (**a**); transformations of β-agostic complex **I-5pp**, the values of calculated free energies (kcal/mol) relative to **I-2pβ-c** are given (**b**)**.**

The energy gain due to Zr–O bonding can be estimated by the value of ~9 kcal/mol (the difference of the free energies of **I-2pβ-c-*anti*** and **I-2pβ**, Scheme 7a. Such a significant difference inevitably affects the energies of the stationary points and transition states for the key stage of the reaction (Scheme 7b). The activation energy for β-hydride elimination, assisted by Zr–O coordination (**TS-56pp-c-*syn***), shown in Scheme 7b, is relatively low, but this process results in high-energy intermediate **I-6pp-c-*syn***, dissociation of this complex with a formation of hydride **I-0** requires more than 32 kcal/mol and therefore seems improbable. Our attempt to optimize the structure of the π-complex **I-6pp-c-*anti*** failed, but we found that this complex transformed into **I-5pp-c-*anti*** spontaneously.

The coordination of the third 1-butene molecule (**I-7ppp**) is endergonic, but exothermic process (ΔG = 8.5 kcal/mol, ΔH = –1.3 kcal/mol), and the following chain propagation (**TS-79ppp**) is preferable by the value of ~8 kcal/mol in comparison with chain termination via β-hydride transfer (**TS-78ppp**). Note that the free activation energy of the chain propagation for SiOSi-bridged complex **2** (15.6 kcal/mol) is formally ~3 kcal/mol lower in comparison with zirconocene **1**. Hence, in the absence of organoaluminum compounds, the SiOSi-bridged *ansa*-complex should catalyze oligomerization of α-olefins. Such chemical behavior was observed experimentally (Table 1, Run 22).

**Scheme 8:**
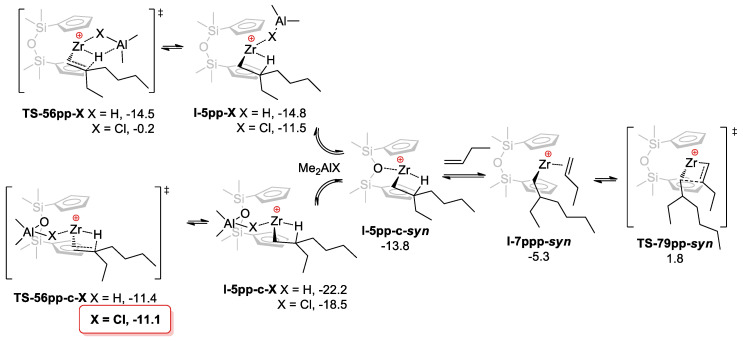
Transformations of O[SiMe_2_(η^5^-C_5_H_4_)]_2_Zr–derived β-agostic complex **I-5pp** with the participance of Me_2_AlX species. The values of calculated free energies (kcal/mol) relative to **I-2pβ-c** are given.

**Figure 4:**
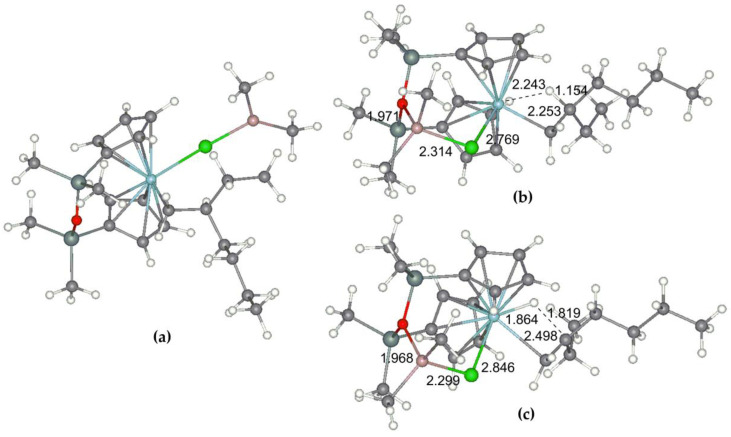
Optimized geometries of O[SiMe_2_(η^5^-C_5_H_4_)]_2_Zr–derived stationary points **I-5pp-Cl** (**a**), **I-5pp-c-Cl** (**b**) and transition state **TS-56pp-c-Cl** (**c**)**.**

To compare the impact of R_2_AlX on the course of oligomerization, we optimized the key stationary points and transition states involved in the transformations of branched alkyl complex **I-5pp** (Scheme 8). Organoaluminum fragment can occupy one of the two positions towards SiMe_2_OSiMe_2_ bridge and alkyl fragment, but the difference of the free energies of **I-5pp-*syn*** and **I-5pp-*anti*** was found to be ~ 1 kcal/mol. Coordination of R_2_AlX was energetically favorable in comparison with 1-butene even though such coordination results in the loss of Zr-O bonding, and Zr-Al_1_ complexes **I-5pp-X** were readily subjected by β-hydride elimination similarly to zirconocene **1**. The substantial difference between Zr-Al_1_ species formed by zirconocenes **1** and **2** is in the formation of low-energy complexes **I-5pp-c-X** containing O–Al bond (Scheme 8) that also can eliminate the molecule of methylenealkane via transition states **TS-56pp-c-X** with comparable (X = H) or substantially lower (X = Cl, only 7.4 kcal/mol) activation barriers. In all cases, the coordination of Me_2_AlX greatly facilitates the formation of α-olefin dimer via β-hydride elimination. However, precisely for SiOSi-bridged zirconocene and X = Cl, dual coordination of R_2_AlCl (Figure 4c) provides the fast dimer formation.

## 4. Conclusions

Numerous theoretical studies of the polymerization mechanisms of zirconocene-catalyzed polymerization were limited by ethylene and propylene as substrates. In the present work, we refused to the use of propylene as a model of α-olefin molecule, and 1-butene looked more adequate. In the framework of the mononuclear mechanism of zirconocene-catalyzed oligomerization, DFT modeling predicted that oligomerization is preferred over dimerization, both for (η^5^-C_5_H_5_)]_2_Zr- (**1**/**1′**) and O[SiMe_2_(η^5^-C_5_H_4_)]_2_Zr-based (**2**/**2′**) catalytic systems. In practice, in the absence of organoaluminum compounds, the complex **1′** catalyzed oligomerization (although mostly producing dimer), while zirconocene **2′** catalyzed the formation of the oligomer mixture in an equal mass ratio. These results correlate with the differences of the free energies of the isomeric transition states of chain propagation and chain release via β-hydride transfer for zirconocenes **1** and **2**.

In the presence of TIBA, MMAO-12, or both organoaluminum components, the selectivity of the dimerization increased. The presence of Al-Cl heightened the dimerization selectivity in all cases, the effect of R_2_AlCl was most pronounced for **2**/**2′**. This experimental observation is consistent with the results of the modeling that predicted the facilitation of β-hydride elimination due to the Zr–Al cooperative effect in Zr–Al_1_ bimetallic complexes. On the other hand, for (η^5^-C_5_H_5_)]_2_Zr-based catalyst our calculations predicted the minimal activation barriers of the elimination for Zr-Al hydride species. The activation barriers for chloro complexes were found to be substantially higher. We can only assume that the formation of Zr-Al_1_ hydrides is hampered by additional factors, namely by the higher stability of R_2_AlH dimer and trimer (see Appendix A) as well as Zr–Al_2_ trihydride complexes in comparison with chloro complexes.

The results of both oligomerization experiments and DFT calculations demonstrate the qualitative difference between (η^5^-C_5_H_5_) _2_Zr- and O[SiMe_2_(η^5^-C_5_H_4_)]_2_Zr-based catalytic systems that can be attributed to the ability of the bridged oxygen to coordinate the metal atoms, both Zr and Al. Such coordination plays a crucial role in combination with R_2_AlCl coordination that results in dramatic lowering of the activation barrier of β-hydride elimination with a formation of methylenealkanes. SiOSi-bridged zirconocenes have long been known, but this structural aspect of their reactivity is undervalued. We expect that the –SiOSi– bridge, and potentially also –SiOR substituents in cyclopentadienyl rings, can be used in the design of novel efficient metallocene catalysts for α-olefin oligomerization and polymerization.

## Figures and Tables

**Table 1:** Results of 1-octene oligomerization experiments.

Run	Pre- cat.	TiBA/Zr Ratio	Activator	[Act]/ [Precat] Ratio	H_2_	Conv. %	Dimer (C16), Trimer (C24), Tetramer (C32) and Pentamer (C40) wt. % in the Products			
							C16	C24	C32	C40
1	**1**	20	MMAO-12	10	–	85	88.8	9.2	2.1	–
2	**1**	20	MMAO-12 +1 eq. Et_2_AlCl	10	–	74	90.3	7.8	1.9	–
3	**1**	20	MMAO-12	10	1 bar	84	92.0	6.7	1.3	–
4	**1**	20	MMAO-12	200	–	68	77.6	15.1	5.4	1.9
5	**1**	20	NB^F^	1	–	53	81.6	13.2	4.2	1.0
6	**1**	20	NB^F^	1	1 bar	70	84.6	11.6	3.3	0.5
7	**1′**	–	MMAO-12	10	–	47	88.7	8.6	2.6	–
8	**1′**	–	MMAO-12 +1 eq. Et_2_AlCl	10	–	60	93.4	5.6	1.0	–
9	**1′**	–	NB^F^	1	–	14	78.2	17.7	4.0	–
10	**1′**	20	NB^F^	1	–	8	75.2	18.9	5.8	–
11	**1′**	–	NB^F^ +1 eq. Et_2_AlCl	1	–	7	90.7	8.2	1.0	–
12	**1′**	20	NB^F^	1	1 bar	18	72.3	16.8	9.2	1.7
13	**2**	20	MMAO-12	10	–	82	92.4	7.1	0.5	–
14	**2**	20	MMAO-12 +1 eq. Et_2_AlCl	10	–	79	96.3	3.5	0.2	–
15	**2**	20	MMAO-12	10	1 bar	86	92.3	7.2	0.5	–
16	**2**	20	MMAO-12	200	–	70	78.1	15.8	5.7	0.4
17	**2**	20	NB^F^	1	–	68	67.7	23.3	7.6	1.4
18	**2**	20	NB^F^	1	1 bar	85	82.2	13.6	3.4	0.8
19	**2′**	–	MMAO-12	10	–	65	58.4	25.1	11.5	5.0
20	**2′**	–	MMAO-12 +1 eq. Et_2_AlCl	10	–	57	87.6	11.2	1.2	–
21	**2′**	–	NB^F^	1	–	60	25.2	25.2	22.9	26.7
22	**2′**	20	NB^F^	1	–	13	47.4	21.7	15.6	15.2
23	**2′**	–	NB^F^ +1 eq. Et_2_AlCl	1	–	38	70.3	22.1	6.3	1.3
24	**2′**	–	NB^F^	1	1 bar	73	48.3	26.0	14.6	11.1

## Supplementary Materials

The following are available online at https://www.mdpi.com/2073-4360/12/7/1590/s1, DFT calaulations data: plots of the molecular geometries, energies and cartesian coordinates for all stationary points and transition states mentioned in the article, Figure S1: ^1^H NMR spectrum (C_6_D_6_, 400 MHz, 20 °C) of **2′**, Figure S2: ^13^C NMR spectrum (C_6_D_6_, 101 MHz, 20 °C) of **2′**, in pdf format.

## Appendix A

The relative stability of R_2_AlX dimers and trimers (X = H, Cl) is of direct relevance to the process of reversible and competetive bonding of R_2_AlX with zirconocene cations. We optimized the structures of Me_2_AlX monomers, dimers, and trimers and calculated the relative free energies and free enthalpies of dimer and trimer formation. The results are presented below in kcal/mol.

Thus, cyclic trimers of R_2_AlH represent the most stable species. The release of free monomeric R_2_AlH seems less likely than the release of monomeric R_2_AlCl. In particular, the release of Me_2_AlCl in the reaction between Me_2_AlH dimer with Me_2_Al(μ-H)(μ-Cl)AlMe_2_, resulting in the formation of stable Me_2_AlH trimer, requires only 5 kcal/mol in G scale.
