# Supplementary material for: Experimental and Theoretical Study of Zirconocene-Catalyzed Oligomerization of 1-Octene
Source: Polymers (Basel). 2020 Jul 17;12(7):1590. doi: 10.3390/polym12071590 (PMC7407618; doi:10.3390/polym12071590)
Supplement: Supplementary file 1 [file polymers-12-01590-s001.pdf]

Supporting Information

Experimental and Theoretical Study of Zirconocene-Catalyzed Oligomerization of 1-Octene

Ilya Nifant'ev [1,2,*], Alexander Vinogradov [1], Alexey Vinogradov [1], Stanislav Karchevsky [3] and Pavel Ivchenko [1,2]

1 A.V. Topchiev Institute of Petrochemical Synthesis RAS, 29 Leninsky Pr., 119991 Moscow, Russia; amvvin@mail.ru (A.V.); vinasora@gmail.com (A.V.); phpasha1@yandex.ru (P.I.)
2 Chemistry Department, M.V. Lomonosov Moscow State University, 1 Leninskie Gory Str., Building 3, 119991 Moscow, Russia
3 Joint-Stock Company "Institute of Petroleum Refining and Petrochemistry", 12 Iniciativnaya Str., 450065 Ufa, Republic of Bashkortostan, Russia; st_karchevsky@mail.ru
* Correspondence: ilnif@yahoo.com; Tel.: +7-495-939-4098

S1. DFT calculations data: molecular structures, energies and cartesian coordinates 2

S1.1. Olefins and organoaluminium compounds 2

S1.2. Mononuclear (η^5-C_5H_5)$_2$Zr-based catalytic species 8

S1.3. Zr-Al$_1$ (η^5-C_5H_5)$_2$Zr-based catalytic species 62

S1.4. Zr-Al$_2$ (η^5-C_5H_5)$_2$Zr-based catalytic species 73

S1.5. Mononuclear O[SiMe$_2$(η^5-C_5H_4)]$_2$Zr-based catalytic species 103

S1.7. Zr-Al$_1$ O[SiMe$_2$(η^5-C_5H_4)]$_2$Zr-based catalytic species 119

S2. NMR spectra of **2'** 133

S1. DFT calculations data: molecular structures, energies and cartesian coordinates

S1.1. Olefins and organoaluminium compounds

1-Butene

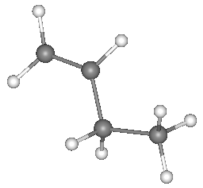

Zero-point vibrational energy	286742.8 (Joules/Mol)
	68.53317 (Kcal/Mol)
Zero-point correction=	0.109215 (Hartree/Particle)
Thermal correction to Energy=	0.113803
Thermal correction to Enthalpy=	0.114747
Thermal correction to Gibbs Free Energy=	0.082595
Sum of electronic and zero-point Energies=	-157.025929
Sum of electronic and thermal Energies=	-157.021340
Sum of electronic and thermal Enthalpies=	-157.020396
Sum of electronic and thermal Free Energies=	-157.052549

cartesian

6	-1.96500003	-0.02060000	0.00000000	1	2.68330002	-0.47279999	0.00000000
6	-0.69260001	0.37729999	0.00000000	1	1.91320002	0.85070002	-0.88380003
6	0.50480002	-0.54879999	0.00000000	1	1.91320002	0.85079998	0.88360000
6	1.83130002	0.21120000	0.00000000	1	-0.47900000	1.44679999	0.00010000
1	0.45539999	-1.20360005	-0.87610000	1	-2.78270006	0.69360000	0.00000000
1	0.45550001	-1.20350003	0.87620002	1	-2.23000002	-1.07620001	-0.00010000

3-Methyleneheptane

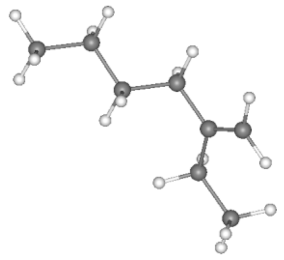

Zero-point vibrational energy	591267.0 (Joules/Mol)
	141.31621 (Kcal/Mol)
Zero-point correction=	0.225202 (Hartree/Particle)
Thermal correction to Energy=	0.235559
Thermal correction to Enthalpy=	0.236503
Thermal correction to Gibbs Free Energy=	0.188858
Sum of electronic and zero-point Energies=	-314.098930
Sum of electronic and thermal Energies=	-314.088573
Sum of electronic and thermal Enthalpies=	-314.087628
Sum of electronic and thermal Free Energies=	-314.135274

cartesian

6	1.83260000	-0.84930003	0.32130000	1	-0.44150001	1.75759995	0.64310002
6	1.22140002	0.51819998	0.11420000	1	-1.17900002	0.24910000	-1.22239995
6	-0.18500000	0.69270003	0.64010000	1	-1.02260005	-1.14450002	-0.16200000
6	-1.23969996	-0.06980000	-0.17440000	1	-2.88120008	1.22860003	0.33899999
6	-2.65770006	0.15549999	0.35010001	6	-3.70880008	-0.59710002	-0.46489999
6	1.86010003	1.52110004	-0.49680001	1	-4.71570015	-0.43120000	-0.07280000
6	3.25830007	-1.02479994	-0.19170000	1	-3.51650000	-1.67429996	-0.44999999
1	1.18390000	-1.59700000	-0.15090001	1	-3.69790006	-0.27300000	-1.50999999
1	3.31310010	-0.86420000	-1.27199996	1	1.38759995	2.49300003	-0.61580002
1	3.61649990	-2.03640008	0.01450000	1	2.86409998	1.41429996	-0.89630002
1	3.94260001	-0.32110000	0.29030001	1	-2.70869994	-0.15970001	1.39900005
1	-0.22910000	0.35150000	1.68330002	1	1.79789996	-1.07190001	1.39569998

Me_2AlH

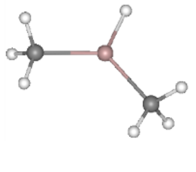

Zero-point vibrational energy	206187.7 (Joules/Mol)
	49.28004 (Kcal/Mol)
Zero-point correction	0.078533 (Hartree/Particle)
Thermal correction to Energy	0.084839
Thermal correction to Enthalpy	0.085783
Thermal correction to Gibbs Free Energy	0.049544
Sum of electronic and zero-point Energies	-322.675915
Sum of electronic and Thermal Energies	-322.669609
Sum of electronic and Thermal Enthalpies	-322.668665
Sum of electronic and Thermal Free Energies	-322.704904

cartesian

13	0.00000995	0.69409001	0.00005000	1	-2.31759000	0.04249001	-0.87424999
6	-1.71979010	-0.24191001	0.00005000	1	2.31751013	0.04159001	-0.87465000
6	1.71980989	-0.24191001	0.00005000	1	2.31810999	0.04289000	0.87395000
1	-1.62759006	-1.33160996	-0.00025000	1	1.62750995	-1.33160996	0.00085000
1	-2.31788993	0.04209000	0.87424999	1	-0.00009005	2.28389001	-0.00005000

Me_2AlCl

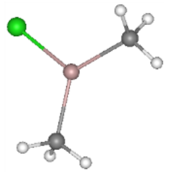

Zero-point vibrational energy	193594.6 (Joules/Mol)
	46.27022 (Kcal/Mol)
Zero-point correction	0.073736 (Hartree/Particle)
Thermal correction to Energy	0.081041
Thermal correction to Enthalpy	0.081985
Thermal correction to Gibbs Free Energy	0.041705
Sum of electronic and zero-point Energies	-782.313017
Sum of electronic and Thermal Energies	-782.305712
Sum of electronic and Thermal Enthalpies	-782.304768
Sum of electronic and Thermal Free Energies	-782.345048

cartesian

17	-2.68446016	-0.00012997	0.00012000	1	-0.02626008	-2.33113003	0.87361997
13	-0.55946004	-0.00002997	0.00052000	1	-0.01906008	-2.32693005	-0.87867999
6	0.28773999	-1.74942994	0.00012000	1	-0.01836008	2.32646990	-0.87927997
6	0.28753996	1.74947011	0.00022000	1	-0.02736008	2.33156991	0.87301999
1	1.37994003	-1.69572997	0.00462000	1	1.37974000	1.69587004	0.00572000

Me_2AlH dimer

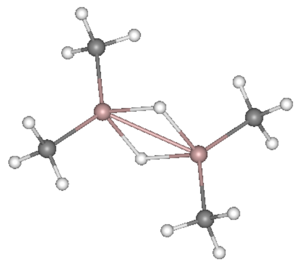

Zero-point vibrational energy 206187.7 (Joules/Mol)

49.28004 (Kcal/Mol)

Zero-point correction= 0.078533 (Hartree/Particle)

Thermal correction to Energy= 0.084839

Thermal correction to Enthalpy= 0.085783

Thermal correction to Gibbs Free Energy= 0.049544

Sum of electronic and zero-point Energies= -322.675915

Sum of electronic and thermal Energies= -322.669609

Sum of electronic and thermal Enthalpies= -322.668665

Sum of electronic and thermal Free Energies= -322.704904

cartesian

1	0.00000000	-0.00010000	-1.14639997	1	2.81500006	1.88979995	-0.87849998
1	-0.00010000	-0.00010000	1.14590001	13	-1.32050002	0.00000000	-0.00020000
13	1.32040000	-0.00010000	-0.00020000	6	-2.17490005	-1.75619996	0.00010000
6	2.17490005	-1.75619996	0.00010000	6	-2.17449999	1.75629997	0.00010000
6	2.17459989	1.75619996	0.00010000	1	-2.80819988	-1.89260006	0.88349998
1	2.81310010	-1.89030004	0.88029999	1	-1.44410002	-2.57110000	-0.00700000
1	2.81509995	-1.88979995	-0.87879997	1	-1.44350004	2.57100010	-0.00700000
1	1.44400001	-2.57110000	-0.00100000	1	-2.81949997	1.88779998	-0.87559998
1	2.81250000	1.89059997	0.88050002	1	-2.80789995	1.89289999	0.88340002
1	1.44360006	2.57100010	-0.00130000	1	-2.81990004	-1.88750005	-0.87550002

Me_2AlCl dimer

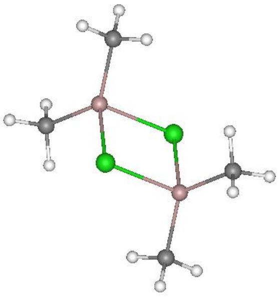

Zero-point vibrational energy	387211.0 (Joules/Mol)
	92.54566 (Kcal/Mol)
Zero-point correction=	0.147481 (Hartree/Particle)
Thermal correction to Energy=	0.159552
Thermal correction to Enthalpy=	0.160496
Thermal correction to Gibbs Free Energy=	0.109141
Sum of electronic and zero-point Energies=	-1564.675813
Sum of electronic and thermal Energies=	-1564.663742
Sum of electronic and thermal Enthalpies=	-1564.662798
Sum of electronic and thermal Free Energies=	-1564.714154

cartesian

17	0.00000000	-0.00020000	1.64549994	1	3.10080004	-1.91480005	0.88160002
17	0.00000000	-0.00020000	-1.64549994	13	-1.65450001	0.00010000	0.00000000
13	1.65450001	0.00000000	-0.00010000	6	-2.46810007	1.76269996	0.00000000
6	2.46840000	1.76250005	0.00000000	6	-2.46889997	-1.76220000	0.00010000
6	2.46860003	-1.76240003	0.00010000	1	-3.10109997	1.91499996	-0.88099998
1	3.10120010	1.91489995	-0.88110000	1	-1.71130002	2.55419993	-0.00070000
1	3.10039997	1.91509998	0.88160002	1	-1.71239996	-2.55399990	-0.00050000
1	1.71169996	2.55410004	-0.00040000	1	-3.10089993	-1.91470003	0.88169998
1	3.10130000	-1.91480005	-0.88110000	1	-3.10179996	-1.91429996	-0.88099998
1	1.71200001	-2.55399990	-0.00010000	1	-3.09999990	1.91550004	0.88169998

Me_2AlH trimer

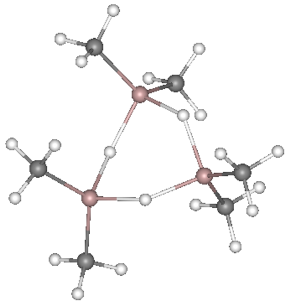

Zero-point vibrational energy	640581.7 (Joules/Mol)
	153.10270 (Kcal/Mol)
Zero-point correction=	0.243985 (Hartree/Particle)
Thermal correction to Energy=	0.263940
Thermal correction to Enthalpy=	0.264884
Thermal correction to Gibbs Free Energy=	0.195245
Sum of electronic and zero-point Energies=	-968.123175
Sum of electronic and thermal Energies=	-968.103220
Sum of electronic and thermal Enthalpies=	-968.102276
Sum of electronic and thermal Free Energies=	-968.171914

cartesian

1	-1.00189996	0.11320000	0.76520002	1	0.75209999	2.31599998	2.18379998
1	0.72960001	1.14390004	-0.73250002	1	-2.10960007	1.34309995	-1.98510003
13	-1.02119994	-1.48889995	0.15700001	1	-3.00430012	2.21770000	-0.74390000
6	-0.79879999	-2.66030002	1.70449996	1	-1.79219997	3.05640006	-1.70570004
6	-2.37450004	-1.59909999	-1.25119996	1	-0.89039999	2.94180012	2.34069991
1	-0.66740000	-3.70350003	1.39600003	1	0.49620000	-1.36609995	-0.62989998
1	-1.67470002	-2.63000011	2.36179996	13	1.74460006	-0.20930000	-0.45860001
1	0.07300000	-2.38569999	2.30749989	6	2.27169991	-0.19710000	1.42680001
1	-2.66860008	-2.63619995	-1.44669998	6	2.89269996	-0.37189999	-2.02880001
1	-2.02180004	-1.17869997	-2.19880009	1	2.91930008	0.65679997	1.65750003
1	-3.28360009	-1.05209994	-0.97619998	1	2.83509994	-1.10090005	1.68659997
13	-0.65890002	1.66550004	0.12700000	1	1.41680002	-0.14470001	2.11089993
6	-0.07530000	2.77020001	1.62909997	1	3.61170006	0.45260000	-2.08500004
6	-2.01690006	2.10710001	-1.20679998	1	2.31609988	-0.36680001	-2.95889997
1	0.26150000	3.75550008	1.28750002	1	3.47199988	-1.30130005	-2.00720000

Me_2AlCl trimer

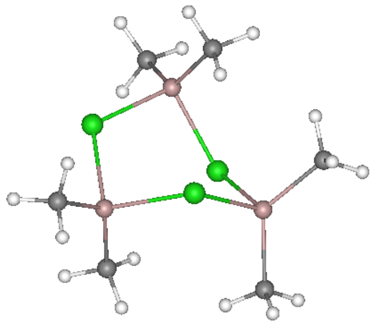

Zero-point vibrational energy	590783.9 (Joules/Mol)
	141.20075 (Kcal/Mol)
Zero-point correction=	0.225018 (Hartree/Particle)
Thermal correction to Energy=	0.248130
Thermal correction to Enthalpy=	0.249075
Thermal correction to Gibbs Free Energy=	0.172983
Sum of electronic and zero-point Energies=	-2347.021455
Sum of electronic and thermal Energies=	-2346.998343
Sum of electronic and thermal Enthalpies=	-2346.997399
Sum of electronic and thermal Free Energies=	-2347.073490

cartesian

17	0.69290000	-1.35420001	-1.16960001	1	2.54010010	1.79770005	-1.69980001
17	0.82120001	1.26180005	1.18270004	1	2.34480000	-1.62360001	2.07960010
13	-1.17089999	-1.91520000	0.10870000	1	3.52609992	-2.15700006	0.87959999
6	-2.18969989	-3.11969995	-1.02090001	1	3.86929989	-0.75340003	1.88849998
6	-0.60360003	-2.14660001	1.95360005	1	3.41269994	0.36590001	-2.24259996
1	-3.19400001	-3.28929996	-0.61830002	17	-2.29909992	0.11910000	-0.02200000
1	-1.70599997	-4.09829998	-1.10780001	13	-0.96190000	2.02880001	-0.10150000
1	-2.30739999	-2.71950006	-2.03309989	6	-0.37390000	2.21199989	-1.94509995
1	-1.46759999	-2.40380001	2.57890010	6	-1.85969996	3.31520009	1.04110003
1	-0.14700000	-1.24389994	2.38949990	1	0.34410000	3.03110003	-2.06329989
1	0.11690000	-2.96749997	2.05080009	1	-1.23409998	2.44849992	-2.58229995
13	2.28259993	-0.11370000	-0.00200000	1	0.08340000	1.30560005	-2.35060000
6	3.16230011	0.96429998	-1.35920000	1	-1.23109996	4.19220018	1.23029995
6	3.07380009	-1.27180004	1.34119999	1	-2.11579990	2.88010001	2.01239991
1	4.09899998	1.38740003	-0.98040003	1	-2.78999996	3.67499995	0.58890003

S1.2. Mononuclear (η^5-C_5H_5)$_2$Zr-based catalytic species

I-0

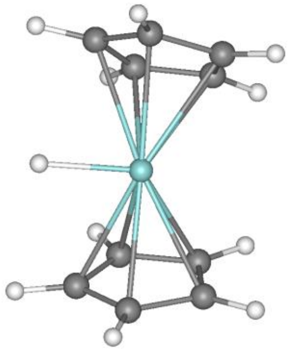

Zero-point vibrational energy 463865.2 (Joules/Mol)

110.86645 (Kcal/Mol)

Zero-point correction= 0.176677 (Hartree/Particle)

Thermal correction to Energy= 0.186380

Thermal correction to Enthalpy= 0.187324

Thermal correction to Gibbs Free Energy= 0.139040

Sum of electronic and zero-point Energies= -3927.913963

Sum of electronic and thermal Energies= -3927.904260

Sum of electronic and thermal Enthalpies= -3927.903316

Sum of electronic and thermal Free Energies= -3927.951600

cartesian

6	1.65699542	1.25530446	0.24621817	6	-1.66550457	0.60030454	-1.02108181
6	1.66519547	0.60110456	-1.02058184	6	-2.18300462	-0.71069545	-0.83978182
6	2.18269539	-0.70999545	-0.84048182	6	-2.45650458	-0.87599546	0.54441822
6	2.45659542	-0.87649548	0.54351819	1	-2.87130451	-1.76639557	1.00431824
6	2.14969540	0.34390455	1.20781815	1	-2.27640462	0.54650456	2.26601815
1	2.27709556	0.54470456	2.26621819	1	-1.35900450	2.27930450	0.43321818
1	1.35929549	2.27910447	0.43511820	1	-2.35560465	-1.44319546	-1.61608183
1	2.35509539	-1.44189548	-1.61728179	40	-0.00000458	-0.59369546	0.34441817
1	2.87139559	-1.76719546	1.00261819	1	-0.00000458	-1.95329547	-0.85748184
6	-2.14930463	0.34480453	1.20771813	1	-1.38110459	1.04340446	-1.96748185
6	-1.65690458	1.25540447	0.24521819	1	1.38059545	1.04500449	-1.96658182

I-1i

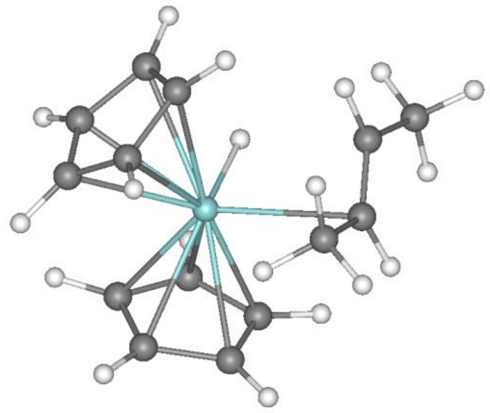

Zero-point vibrational energy	759781.4 (Joules/Mol)
	181.59211 (Kcal/Mol)
Zero-point correction=	0.289385 (Hartree/Particle)
Thermal correction to Energy=	0.305043
Thermal correction to Enthalpy=	0.305987
Thermal correction to Gibbs Free Energy=	0.246554
Sum of electronic and zero-point Energies=	-4085.001165
Sum of electronic and thermal Energies=	-4084.985507
Sum of electronic and thermal Enthalpies=	-4084.984563
Sum of electronic and thermal Free Energies=	-4085.043996

cartesian

6	0.76459414	-2.41164994	-0.10807648	1	2.92599416	1.76524997	0.99532354
6	1.15639412	-2.03804994	1.20182359	40	0.45469412	0.07124995	0.02912353
6	2.30499411	-1.21235001	1.08972359	6	-3.10710597	-0.28455004	1.41622353
6	2.64779425	-1.11285007	-0.28757647	6	-2.00620580	-0.03995004	-0.83797646
6	1.69699419	-1.84695005	-1.02777648	6	-2.26280594	-0.76605004	0.27522352
1	1.69559407	-1.98095012	-2.10337639	6	-1.30410588	-0.55125004	-2.06827641
1	-0.04720587	-3.08245015	-0.36167648	1	-2.39580584	0.97504997	-0.88787645
1	0.68149412	-2.33995008	2.12512350	1	-0.28130585	-0.06285004	1.68142354
1	2.84499407	-0.76925004	1.91732359	1	-3.23430586	0.79924995	1.39302349
6	-0.25680590	2.48624992	0.29372352	1	2.40589404	1.91874993	-1.64567649
6	0.75989413	2.23724985	1.25402355	1	3.49249411	-0.57285005	-0.69727647
6	1.95989418	1.96434999	0.54932356	1	-4.09570599	-0.74905002	1.34012353
6	1.68679416	2.04224992	-0.84537643	1	-1.96060586	-1.81375003	0.29972351
6	0.31779414	2.37835002	-0.99677646	1	-2.67760587	-0.57435006	2.37552357
1	-0.19510588	2.53524995	-1.93927646	1	-1.81420588	-0.19475004	-2.96667647
1	-1.28470588	2.74044991	0.52082354	1	-1.24440587	-1.63925004	-2.10117650
1	0.64169413	2.27944994	2.32812357	1	-0.27000588	-0.15005004	-2.21087646

I-1p

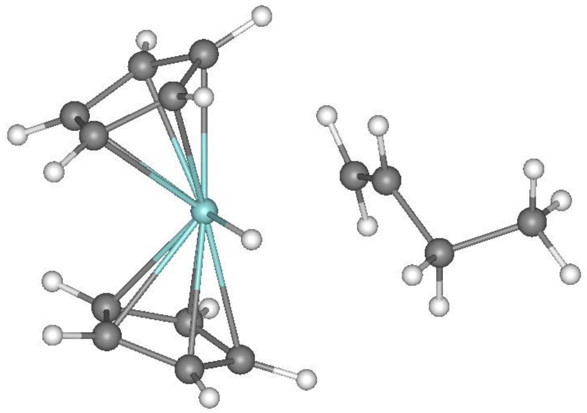

Zero-point vibrational energy 756060.2 (Joules/Mol)

180.70272 (Kcal/Mol)

Zero-point correction= 0.287968 (Hartree/Particle)

Thermal correction to Energy= 0.303396

Thermal correction to Enthalpy= 0.304340

Thermal correction to Gibbs Free Energy= 0.243656

Sum of electronic and zero-point Energies= -4084.992614

Sum of electronic and thermal Energies= -4084.977187

Sum of electronic and thermal Enthalpies= -4084.976243

Sum of electronic and thermal Free Energies= -4085.036927

cartesian

6	0.16258821	2.43669415	0.11241176	1	3.07198834	-1.06710589	-1.94758832
6	0.84528822	2.24869418	-1.11828828	40	0.85608822	0.02799408	0.03311176
6	2.19518828	1.92649412	-0.81548822	6	-1.33541179	-0.27660590	1.48071170
6	2.34908819	1.94129407	0.60001177	6	-1.94301188	-0.63380593	0.32611176
6	1.09398818	2.25769401	1.16951168	6	-2.90651178	0.22179408	-0.43478823
1	0.88478822	2.35419416	2.22921181	1	-1.54541183	0.68739408	1.93991172
1	-0.88071179	2.70729399	0.22041176	1	-0.79901183	-1.01350594	2.08411193
1	0.42028821	2.35759401	-2.10648823	1	-0.04791179	-0.10350592	-1.54458833
1	2.98108840	1.74629414	-1.53848827	1	-2.59021163	0.27479410	-1.48088825
6	1.02858818	-2.45150590	0.29931179	1	-2.91211176	1.23619413	-0.02648824
6	1.35248816	-2.17570591	-1.05388832	1	3.89708829	-0.76340592	0.60071176
6	2.55658841	-1.42200589	-1.06338823	1	3.26838827	1.76959407	1.14551175
6	2.99238825	-1.26410592	0.27961177	1	-1.79771173	-1.64610589	-0.05708824
6	2.04638815	-1.89020586	1.12311172	6	-4.31761169	-0.38760591	-0.35628822
1	2.10538816	-1.95560586	2.20391178	1	-4.33391190	-1.39830589	-0.77048820
1	0.18588823	-3.03900599	0.64541179	1	-5.01581192	0.22319408	-0.93028826
1	0.79908824	-2.49660587	-1.92558825	1	-4.66731167	-0.43250591	0.67701179

I-1s

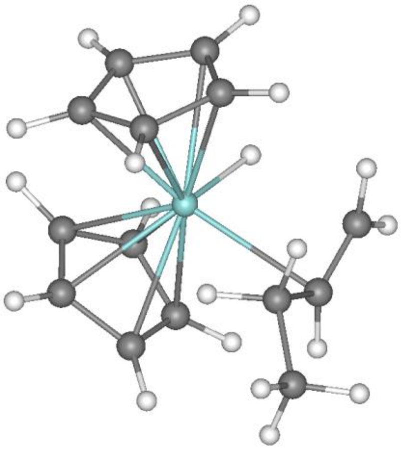

Zero-point vibrational energy 759148.5 (Joules/Mol)

181.44085 (Kcal/Mol)

Zero-point correction= 0.289144 (Hartree/Particle)
Thermal correction to Energy= 0.304733
Thermal correction to Enthalpy= 0.305677
Thermal correction to Gibbs Free Energy= 0.246188
Sum of electronic and zero-point Energies= -4084.995512
Sum of electronic and thermal Energies= -4084.979923
Sum of electronic and thermal Enthalpies= -4084.978979
Sum of electronic and thermal Free Energies= -4085.038468

cartesian

6	-0.89250588	-2.39487338	-0.09580000	1	-1.93080580	2.46002650	-1.86830008
6	-1.92790580	-1.90707350	-0.93240005	40	-0.57580590	0.08722650	-0.21869999
6	-2.77040577	-1.08417344	-0.14060000	1	1.41029418	-0.17687351	-2.95099998
6	-2.27920580	-1.10017347	1.19489992	6	1.94189405	-0.08997350	-0.89910001
6	-1.11970592	-1.90367353	1.22329998	6	1.41169417	-0.68287349	-1.99150002
1	-0.52530587	-2.13007355	2.10090017	6	2.05259418	-0.70897347	0.46720001
1	-0.11560586	-3.09157348	-0.38639998	1	2.34969425	0.91442651	-1.00220001
1	-2.06720591	-2.13227344	-1.98080003	1	-0.87230587	0.07692650	-2.01419997
1	-3.65690589	-0.56957346	-0.49010000	1	-3.06630588	1.86662650	0.50360000
6	0.23879412	2.30972648	0.66880000	1	-2.72120595	-0.59117347	2.04240012
6	-0.02320585	2.50942659	-0.70890003	1	1.09299421	-1.72267354	-1.98650002
6	-1.41680586	2.33972645	-0.92430001	1	1.91019416	-1.79077351	0.41440001
6	-2.01060581	2.02582645	0.32480001	1	1.25659418	-0.32607350	1.16399992
6	-0.98590589	1.99742651	1.31190002	6	3.37799406	-0.35647351	1.15509999
1	-1.12250590	1.81852651	2.37129998	1	3.42599416	-0.79687345	2.15140009
1	1.20559418	2.39282656	1.15349996	1	3.49209428	0.72602654	1.24899995
1	0.70109415	2.77032661	-1.47070003	1	4.21269417	-0.73887348	0.56500000

TS-12p

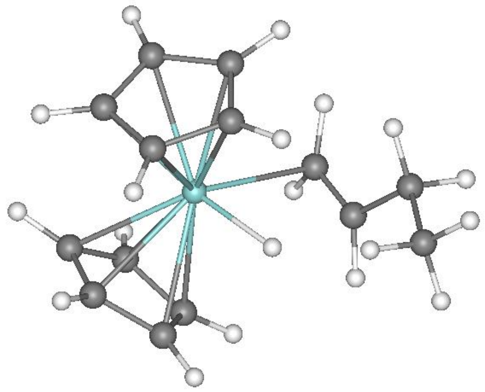

Zero-point vibrational energy 756894.6 (Joules/Mol)

180.90216 (Kcal/Mol)

Zero-point correction= 0.288286 (Hartree/Particle)

Thermal correction to Energy= 0.303365

Thermal correction to Enthalpy= 0.304309

Thermal correction to Gibbs Free Energy= 0.245909

Sum of electronic and zero-point Energies= -4084.990878

Sum of electronic and thermal Energies= -4084.975799

Sum of electronic and thermal Enthalpies= -4084.974855

Sum of electronic and thermal Free Energies= -4085.033255

cartesian

6	-1.62395000	-2.23232365	0.10300002	1	-0.99885011	2.12287641	-2.20259976
6	-1.68215001	-1.68292356	-1.20679998	40	-0.76035011	0.13127647	0.25560001
6	-2.62465024	-0.62232351	-1.18789995	6	2.89914989	-0.49122351	0.24830002
6	-3.15805006	-0.52522349	0.12920001	6	0.91845000	-0.88432354	1.79149997
6	-2.54235005	-1.52662361	0.91960001	6	1.63744986	-1.19702363	0.66180003
1	-2.74355006	-1.72002351	1.96759999	1	0.27724990	-1.63742352	2.24780011
1	-1.01355004	-3.07262349	0.41180000	1	1.24884999	-0.06342353	2.42580009
1	-1.13035011	-2.03152347	-2.06949997	1	0.57144988	-0.47682351	-0.85920000
1	-2.91625023	-0.01702353	-2.03719997	1	2.86554980	0.55537647	0.56819999
6	0.43184993	2.25657654	0.81309998	1	-2.73735023	2.59167647	-0.19799998
6	0.54814994	2.09257650	-0.59119999	1	-3.92595005	0.16257647	0.46110001
6	-0.75555003	2.19087648	-1.14909995	1	3.68834996	-0.96252352	0.85000002
6	-1.66955006	2.44427633	-0.09309999	1	1.45474994	-2.16042352	0.18470001
6	-0.94185007	2.47227645	1.12010002	6	3.25854993	-0.62192351	-1.22950006
1	-1.35605001	2.65717649	2.10500002	1	3.30314994	-1.67282355	-1.52740002
1	1.24965000	2.27427649	1.52300000	1	4.23734999	-0.17982353	-1.42250001
1	1.46665001	1.95087647	-1.14619994	1	2.52374983	-0.12432353	-1.86699998

TS-12s

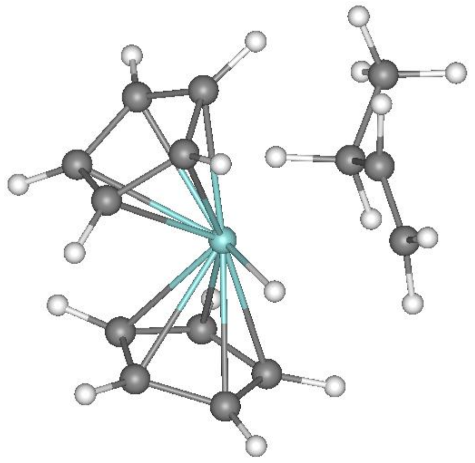

Zero-point vibrational energy	759435.9 (Joules/Mol)
	181.50953 (Kcal/Mol)
Zero-point correction=	0.289254 (Hartree/Particle)
Thermal correction to Energy=	0.303928
Thermal correction to Enthalpy=	0.304873
Thermal correction to Gibbs Free Energy=	0.247845
Sum of electronic and zero-point Energies=	-4084.993624
Sum of electronic and thermal Energies=	-4084.978950
Sum of electronic and thermal Enthalpies=	-4084.978005
Sum of electronic and thermal Free Energies=	-4085.035033

cartesian

6	-0.78683531	-2.43991756	-0.18392354	1	-3.04903555	1.92268240	-0.81862354
6	-1.88723540	-1.94051766	-0.93052351	40	-0.56333536	0.06638239	-0.20472354
6	-2.70213556	-1.19051766	-0.04462354	1	1.42966461	-0.14731762	-2.95102334
6	-2.12263536	-1.25421762	1.25497639	6	1.95846474	-0.01621761	-0.90242350
6	-0.94553536	-2.02771759	1.16777647	6	1.43906462	-0.63921762	-1.98452353
1	-0.29013535	-2.27791762	1.99407637	6	2.09046459	-0.60241765	0.47587648
1	-0.01223534	-3.09881759	-0.55652350	1	2.34716463	0.99058235	-1.03282356
1	-2.08783531	-2.12401772	-1.97702360	1	-0.81273532	0.02618239	-2.00782347
1	-3.62843513	-0.69351763	-0.30432355	1	-2.44873524	1.68238235	1.79807639
6	0.16706467	2.47228241	-0.12262353	1	-2.52833557	-0.81991762	2.15957665
6	-0.88513535	2.40268230	-1.07062352	1	1.14896464	-1.68621767	-1.96792352
6	-2.06903529	2.04288244	-0.37382355	1	1.99616468	-1.69061768	0.44527647
6	-1.75183535	1.91548240	1.00347638	1	1.27506471	-0.24371763	1.16597641
6	-0.36913535	2.16678238	1.16167641	6	3.39176464	-0.17221761	1.16487646
1	0.17296466	2.17588234	2.10017657	1	3.46116447	-0.60121763	2.16487646
1	1.18296468	2.78498244	-0.32502353	1	3.44246483	0.91578239	1.25057638
1	-0.81193537	2.61288238	-2.12892342	1	4.24886465	-0.51161760	0.58057648

I-2pβ

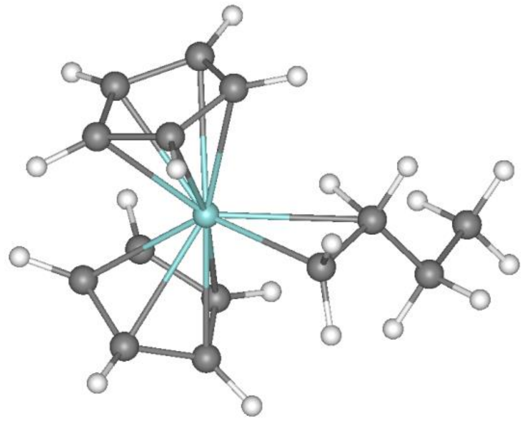

Zero-point vibrational energy 767543.1 (Joules/Mol)
183.44720 (Kcal/Mol)
Zero-point correction= 0.292342 (Hartree/Particle)
Thermal correction to Energy= 0.307725
Thermal correction to Enthalpy= 0.308669
Thermal correction to Gibbs Free Energy= 0.248286
Sum of electronic and zero-point Energies= -4085.009831
Sum of electronic and thermal Energies= -4084.994448

Sum of electronic and thermal Enthalpies= -4084.993504

Sum of electronic and thermal Free Energies= -4085.053887

cartesian

6	-1.68857360	-2.21914697	0.01587060	1	-0.88127351	2.18315315	-2.28752947
6	-1.86707354	-1.62954712	-1.26812935	40	-0.81597352	0.14815296	0.15697059
6	-2.80777335	-0.57834709	-1.13932943	6	2.87242651	-0.49894705	0.30767059
6	-3.20367336	-0.50874704	0.22667059	6	0.64512646	-0.75884706	1.59477055
6	-2.52037334	-1.53784704	0.93197060	6	1.46682644	-1.10404706	0.36857060
1	-2.61157346	-1.75504708	1.98967063	1	0.28522643	-1.63554704	2.13237047
1	-1.03837359	-3.05384684	0.24777059	1	1.17102647	-0.08434703	2.26997042
1	-1.38737357	-1.94804704	-2.18682957	1	0.97752643	-0.74024707	-0.61832941
1	-3.16787338	0.05685296	-1.93942940	1	2.81662655	0.56685293	0.55627060
6	0.37502643	2.25965309	0.81577063	1	-2.72747350	2.63665295	-0.38092941
6	0.56722641	2.10095310	-0.58132941	1	-3.93737340	0.17075296	0.64367062
6	-0.69787353	2.22935295	-1.22032940	1	3.46242642	-0.96894705	1.09997058
6	-1.66907358	2.47515297	-0.21652940	1	1.49332643	-2.18104696	0.17817059
6	-1.00977349	2.48095298	1.04067063	6	3.54752660	-0.69804704	-1.04612935
1	-1.47777355	2.65235305	2.00377059	1	4.55872631	-0.28744704	-1.04432940
1	1.14662647	2.24735308	1.57407057	1	2.98832655	-0.20184702	-1.84722936
1	1.51672637	1.94145298	-1.08052945	1	3.61852646	-1.75974703	-1.29772937

I-2pγ

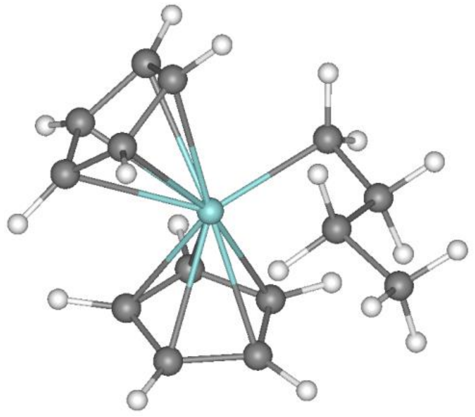

Zero-point vibrational energy 769435.9 (Joules/Mol)

183.89959 (Kcal/Mol)

Zero-point correction= 0.293063 (Hartree/Particle)

Thermal correction to Energy= 0.308352

Thermal correction to Enthalpy= 0.309297

Thermal correction to Gibbs Free Energy= 0.250571

Sum of electronic and zero-point Energies= -4085.005088

Sum of electronic and thermal Energies= -4084.989798

Sum of electronic and thermal Enthalpies= -4084.988854

Sum of electronic and thermal Free Energies= -4085.047579

cartesian

6	0.02609998	2.43440294	-0.79644120	1	-0.92530000	-2.33589697	2.03135896
6	0.46290001	2.32010293	0.54675877	1	-2.88689995	-0.49869704	2.03465891
6	-0.68680000	2.16950297	1.37475884	1	-3.67050004	-0.11799704	-0.52084118
6	-1.82659996	2.18830299	0.53865880	40	-0.56150001	0.10010297	-0.02474118
6	-1.38660002	2.33120298	-0.80594122	1	4.03739977	-1.24199700	0.45235881
1	-2.02090001	2.38210297	-1.68314111	6	1.91050005	-0.87109703	0.44155884
1	0.65630007	2.57820296	-1.66414118	6	3.30040002	-0.72149700	1.06795883
1	1.48870003	2.38040304	0.89325881	1	1.24640000	-0.37009704	1.19915891
1	-0.69070005	2.08130288	2.45505881	1	1.61919999	-1.92509699	0.43045881
1	-2.85559988	2.10950303	0.86635882	6	0.60060000	-0.31739706	-1.86384118
6	-1.20630002	-2.34359694	-0.18834117	6	1.90560007	-0.28809702	-1.00964117
6	-1.43849993	-1.95219707	1.15645885	1	0.68299997	0.39480299	-2.68374109
6	-2.46910000	-0.98189700	1.15985882	1	0.44219998	-1.31299698	-2.28194118
6	-2.87759995	-0.77689701	-0.18864118	1	2.32159996	0.72260296	-0.96914119
6	-2.10260010	-1.62889707	-1.01844120	1	3.58800006	0.33110297	1.12435889
1	-2.18149996	-1.71429706	-2.09464121	1	2.64899993	-0.90399700	-1.52784109
1	-0.48160002	-3.07559705	-0.52414119	1	3.33069992	-1.14539707	2.07255888

I-2sβ

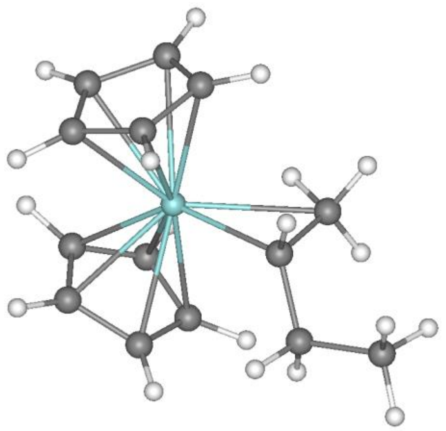

Zero-point vibrational energy	767541.7 (Joules/Mol)
	183.44688 (Kcal/Mol)
Zero-point correction=	0.292341 (Hartree/Particle)
Thermal correction to Energy=	0.307954
Thermal correction to Enthalpy=	0.308898
Thermal correction to Gibbs Free Energy=	0.248891
Sum of electronic and zero-point Energies=	-4085.005421
Sum of electronic and thermal Energies=	-4084.989809
Sum of electronic and thermal Enthalpies=	-4084.988865
Sum of electronic and thermal Free Energies=	-4085.048871

cartesian

6	1.43306470	-2.32816172	0.20122647	1	3.05096459	2.36543822	0.15992647
6	2.40716481	-1.62706172	0.96572649	40	0.81196463	0.11903825	0.11032648
6	3.13486457	-0.78976178	0.08472648	6	-3.78713512	-0.77926177	-0.41227350
6	2.60246468	-0.96296173	-1.22457349	6	-1.27423537	-0.71456176	-0.05177352
6	1.56166470	-1.92986178	-1.14807355	6	-2.48923540	0.00353825	-0.64057350
1	0.96446466	-2.28976178	-1.97737348	6	-1.23523533	-0.74016178	1.47282648
1	0.72446465	-3.05116177	0.58692652	1	-1.22933531	-1.73406172	-0.44317350
1	2.58186460	-1.73876166	2.03002644	1	-2.34323525	0.14233825	-1.71707356
1	3.96546459	-0.15026174	0.35572648	1	-3.72723532	-1.77036166	-0.87017351
6	-0.26923534	2.35703826	0.55022651	1	-3.98643541	-0.91466171	0.65492648
6	1.03576469	2.41673827	1.11562645	1	1.68206465	2.14283824	-2.15267348
6	1.97356462	2.32723832	0.05962648	1	2.96896458	-0.49046177	-2.12777352
6	1.25096464	2.19793820	-1.16017354	1	-4.64173555	-0.25736177	-0.84877348
6	-0.13743535	2.23583841	-0.85257351	1	-1.32773530	-1.73546171	1.91102648
1	-0.94943535	2.17923832	-1.56667352	1	-1.97113538	-0.06876175	1.92142653
1	-1.20013535	2.41803837	1.10142648	1	-0.26063535	-0.37566173	1.94832647
1	1.26866472	2.53953838	2.16762638	1	-2.58883524	1.00373828	-0.20397352

I-2sβ isomer

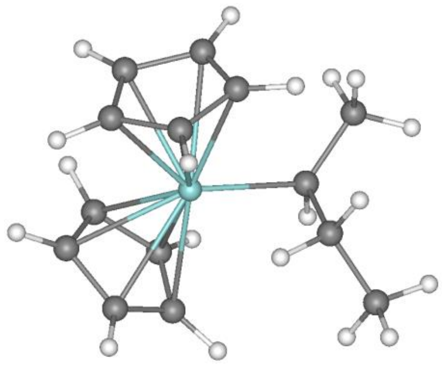

Zero-point vibrational energy	767439.9 (Joules/Mol)
	183.42255 (Kcal/Mol)
Zero-point correction=	0.292302 (Hartree/Particle)
Thermal correction to Energy=	0.307000
Thermal correction to Enthalpy=	0.307945
Thermal correction to Gibbs Free Energy=	0.251073
Sum of electronic and zero-point Energies=	-4085.005571
Sum of electronic and thermal Energies=	-4084.990873
Sum of electronic and thermal Enthalpies=	-4084.989929
Sum of electronic and thermal Free Energies=	-4085.046800

cartesian

6	-0.27051759	2.55976772	0.16380000	1	-3.41411757	-0.98283225	1.23199999
6	-1.41171753	2.30216765	0.97729999	40	-0.64891756	0.06776772	0.09780000
6	-2.44231772	1.81676781	0.14049999	6	1.44638240	-0.12403227	-0.69890004
6	-1.93701756	1.75356781	-1.18900001	6	2.00608230	-1.29063225	-1.50899994
6	-0.60001761	2.23916769	-1.17349994	6	1.88318241	-0.10843228	0.76519996
1	0.04708242	2.34506774	-2.03460002	1	1.74558246	0.81886768	-1.16499996
1	0.67508245	2.95826769	0.51029998	1	1.61228240	-1.28783226	-2.52869987
1	-1.48601758	2.47736788	2.04489994	1	-3.02131772	-1.36483228	-1.40919995
1	-3.44331765	1.54736781	0.45260000	1	-2.49591756	1.45506775	-2.06789994
6	-0.49101758	-2.42633224	0.49570000	1	2.14428234	-1.11663222	1.09860003
6	-1.39841759	-1.91103220	1.46200001	1	1.04318249	0.17866772	1.49070001
6	-2.53241777	-1.41413224	0.77499998	1	1.77318251	-2.26063228	-1.06089997
6	-2.32131767	-1.60993218	-0.61980003	6	3.01028228	0.87326771	1.09759998
6	-1.06271756	-2.25133228	-0.78620005	1	3.90788245	0.58136773	0.54699999
1	-0.61651760	-2.54823232	-1.72789991	1	3.24468231	0.87276769	2.16390014
1	0.46198240	-2.89403224	0.70980000	1	2.75268221	1.89076781	0.79509997
1	-1.25831759	-1.92463219	2.53719997	1	3.09808230	-1.22253227	-1.58639991

I-2sββ

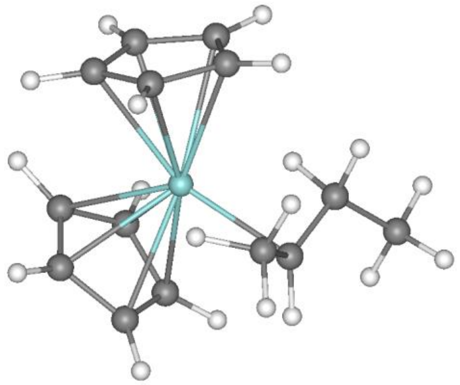

Zero-point vibrational energy	765116.3 (Joules/Mol)
	182.86719 (Kcal/Mol)
Zero-point correction=	0.291417 (Hartree/Particle)
Thermal correction to Energy=	0.306578
Thermal correction to Enthalpy=	0.307522
Thermal correction to Gibbs Free Energy=	0.249454
Sum of electronic and zero-point Energies=	-4085.002546
Sum of electronic and thermal Energies=	-4084.987386
Sum of electronic and thermal Enthalpies=	-4084.986442
Sum of electronic and thermal Free Energies=	-4085.044510

cartesian

6	1.02261174	-2.32203221	-0.10365590	1	2.79541159	2.00646758	0.88334411
6	1.23131180	-1.88383234	1.23014414	40	0.45761171	0.15116765	-0.09405590
6	2.30131173	-0.96073228	1.23184407	6	-3.19598842	-0.51383233	1.17954409
6	2.78151155	-0.84523231	-0.10825590	6	-1.65798831	-0.18283236	-0.81155592
6	1.99811172	-1.69053245	-0.92445588	6	-1.88668823	-0.88133234	0.47744411
1	2.13411164	-1.84573233	-1.98825586	6	-1.04658818	-0.72993231	-2.05065608
1	0.31511173	-3.07663226	-0.42265591	1	-2.26508832	0.70636767	-0.95565587
1	0.67461169	-2.20613217	2.10214400	1	-1.07458830	-0.56633234	1.26044405
1	2.71111155	-0.46363238	2.10264397	1	-3.29088831	0.57056767	1.27524412
6	-0.48908830	2.42916775	0.44534409	1	2.08021164	2.35656762	-1.69165587
6	0.61161172	2.20136762	1.31174409	1	3.61791158	-0.24053237	-0.43705589
6	1.78681183	2.14296770	0.51614410	1	-4.03948832	-0.87883234	0.59104413
6	1.41041183	2.32946777	-0.84015590	1	-3.24868846	-0.95253235	2.17714405
6	0.00321171	2.50696778	-0.88155591	1	-1.51808834	-0.34483236	-2.95595598
1	-0.58978826	2.67766762	-1.77245593	1	-0.96698827	-1.81723237	-2.09925604
1	-1.52408838	2.53316784	0.74784410	1	0.04261172	-0.36083236	-2.20175600
1	0.56561172	2.11606765	2.39104390	1	-1.74718833	-1.96443236	0.41594410

I-2sγ

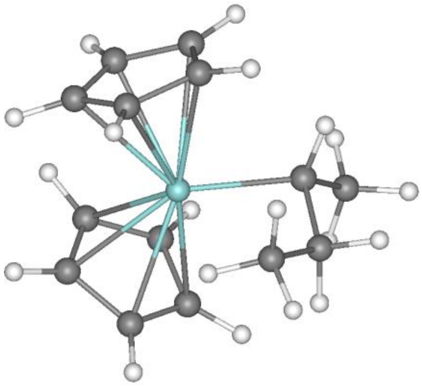

Zero-point vibrational energy 769938.7 (Joules/Mol)

184.01977 (Kcal/Mol)

Zero-point correction= 0.293254 (Hartree/Particle)

Thermal correction to Energy= 0.308503

Thermal correction to Enthalpy= 0.309447

Thermal correction to Gibbs Free Energy= 0.251378

Sum of electronic and zero-point Energies= -4085.001574

Sum of electronic and thermal Energies= -4084.986326

Sum of electronic and thermal Enthalpies= -4084.985382

Sum of electronic and thermal Free Energies= -4085.043451

cartesian

6	1.91058528	1.46632648	0.09813823	1	-2.96571469	-0.30227351	1.94233823
6	1.21238530	1.98782647	1.21753824	1	-3.04281473	1.95622647	0.48903820
6	0.07278533	2.68432665	0.73853827	1	-2.36211467	1.33212650	-2.04856181
6	0.07158531	2.58932662	-0.67856175	40	-0.35461468	0.29732651	0.17573823
6	1.20638537	1.83012652	-1.07336187	6	0.47648531	-1.46557355	1.99873817
1	1.49168539	1.58682644	-2.08986163	1	0.15478532	-0.42937350	2.27583838
1	2.84018517	0.91412652	0.13313822	1	-0.41091466	-2.09997344	1.98583817
1	1.51628530	1.90342653	2.25483823	6	0.81888533	-1.37477350	-0.69706178
1	-0.64981467	3.21842670	1.34343815	6	1.37758529	-1.66057348	0.73863822
1	-0.64811468	3.04882669	-1.34336185	1	0.12338533	-2.18297338	-0.94166178
6	-2.33051467	-1.27657354	0.02883823	1	2.31668520	-1.12617350	0.89683825
6	-2.72421479	-0.21477349	0.88873821	1	1.62558532	-2.72747350	0.75993824
6	-2.76461482	0.97472650	0.12593822	1	1.06188536	-1.73167348	2.88063836
6	-2.39231467	0.64862651	-1.20946181	6	1.93208539	-1.36547351	-1.73576176
6	-2.14191461	-0.74697351	-1.26966178	1	1.52628529	-1.18087351	-2.73396158
1	-1.86141467	-1.30477345	-2.15366173	1	2.45158529	-2.33077335	-1.77076185
1	-2.22451472	-2.31797338	0.30843821	1	2.68648529	-0.59957349	-1.53546178

TS-21s

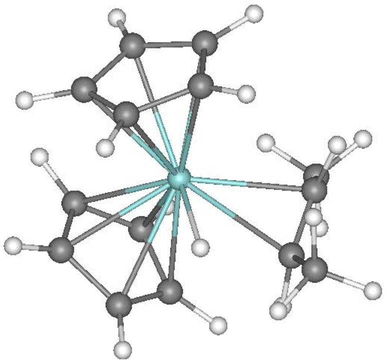

Zero-point vibrational energy 757832.2 (Joules/Mol)

181.12624 (Kcal/Mol)

Zero-point correction= 0.288643 (Hartree/Particle)
Thermal correction to Energy= 0.303483
Thermal correction to Enthalpy= 0.304427
Thermal correction to Gibbs Free Energy= 0.247207
Sum of electronic and zero-point Energies= -4084.995892
Sum of electronic and thermal Energies= -4084.981052
Sum of electronic and thermal Enthalpies= -4084.980108
Sum of electronic and thermal Free Energies= -4085.037329

cartesian

6	1.54655004	-2.04687357	-0.06479710	1	2.10435009	2.59432650	1.02310288
6	1.66604996	-1.53167355	1.25260293	40	0.39675000	0.19422647	-0.08339710
6	2.47175002	-0.36907351	1.19360292	6	-2.84864998	-1.07797348	1.34870291
6	2.88155007	-0.18247354	-0.15939710	6	-1.71504998	-0.67937350	-0.87049711
6	2.31605005	-1.22037351	-0.93109709	6	-1.69665003	-1.27807355	0.38870293
1	2.45965004	-1.36887348	-1.99499702	6	-0.90015006	-1.05137348	-2.06959701
1	1.03915000	-2.96147346	-0.34559709	1	-2.45254993	0.10362647	-1.02689707
1	1.23995006	-1.96067345	2.15010285	1	-0.61355001	-0.46497351	1.34140289
1	2.75895000	0.24342647	2.03940296	1	-3.22574997	-0.05447353	1.30210292
6	-1.12115002	2.19852638	0.26030290	1	1.59975004	2.68272638	-1.61969709
6	-0.09534998	2.26682663	1.23740292	1	3.52845001	0.60442650	-0.52749711
6	1.13495004	2.46932650	0.55840290	1	-3.65974998	-1.75237358	1.06090295
6	0.86984998	2.51862669	-0.83619708	1	-1.15935004	-2.22147369	0.49030292
6	-0.52715003	2.35332632	-1.01799703	1	-2.56194997	-1.30067348	2.37640285
1	-1.04945004	2.36032629	-1.96799707	1	-1.45844996	-0.87137353	-2.99009705
1	-2.17694998	2.06992650	0.46300292	1	-0.53205001	-2.07757330	-2.05809712
1	-0.23384999	2.20392656	2.30920291	1	0.01405001	-0.39237350	-2.23179698

TS-22ps

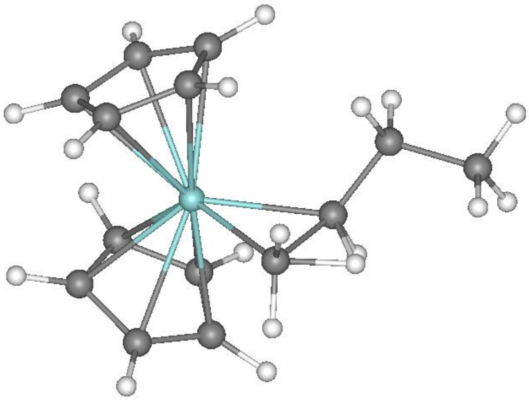

Zero-point vibrational energy	759158.3 (Joules/Mol)
	181.44320 (Kcal/Mol)
Zero-point correction=	0.289148 (Hartree/Particle)
Thermal correction to Energy=	0.304417
Thermal correction to Enthalpy=	0.305361
Thermal correction to Gibbs Free Energy=	0.246329
Sum of electronic and zero-point Energies=	-4084.954491
Sum of electronic and thermal Energies=	-4084.939223
Sum of electronic and thermal Enthalpies=	-4084.938278
Sum of electronic and thermal Free Energies=	-4084.997311

cartesian

6	2.57056165	-0.06239410	0.54035884	6	-3.11093831	-0.70169413	-0.32094118
6	0.79236168	-0.65559411	-1.48384118	6	-2.30413842	-1.66719401	-0.97794116
6	1.34106159	-0.83279413	0.04265882	6	-1.52053833	-2.32119393	0.00495882
1	1.21446180	-1.82539403	0.47915882	6	-1.84503841	-1.77109408	1.26985884
1	1.35176158	0.05990590	-2.08414125	1	1.20566177	2.40440583	-1.19604123
1	0.49206167	-1.54579401	-2.03684115	1	1.06966162	2.24510598	1.49175882
1	1.83106160	-1.29099405	-1.00694120	1	-1.53483820	2.23430586	2.18775892
1	2.41536164	0.15900590	1.59995878	1	-2.99563837	2.39930606	-0.06764118
6	3.88486171	-0.82829410	0.36685881	1	-1.29963827	2.48910594	-2.15654111
1	2.62866163	0.90170586	0.02635882	1	-3.84273839	-0.05809411	-0.79314119
6	-1.14513826	2.26070595	1.17715883	1	-2.30463839	-1.87939405	-2.04034114
6	0.22916168	2.26740599	0.80885881	1	-0.81513834	-3.12319398	-0.17584118
6	0.30096167	2.35310602	-0.60364121	1	-1.42893839	-2.07719398	2.22315884
6	-1.02223825	2.40310597	-1.11224115	1	-3.30313826	-0.17899412	1.84795880
6	-1.91513824	2.35690594	-0.01224118	1	4.72446203	-0.25719410	0.76835883
40	-0.77973831	0.12100589	-0.03724118	1	3.84956145	-1.78839409	0.88825881
6	-2.82773829	-0.76719409	1.07255876	1	4.09366179	-1.02299404	-0.69044119

TS-13

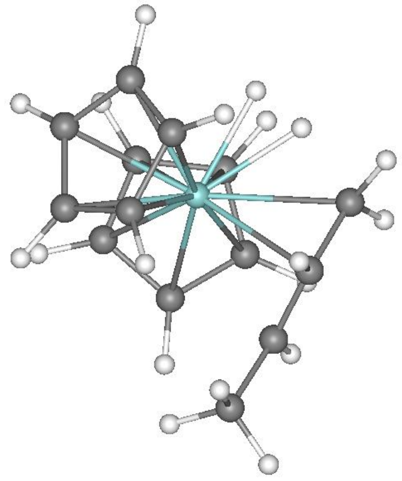

Zero-point vibrational energy	750227.7 (Joules/Mol)
	179.30872 (Kcal/Mol)
Zero-point correction=	0.285747 (Hartree/Particle)
Thermal correction to Energy=	0.300588
Thermal correction to Enthalpy=	0.301532
Thermal correction to Gibbs Free Energy=	0.243977
Sum of electronic and zero-point Energies=	-4084.980959
Sum of electronic and thermal Energies=	-4084.966118
Sum of electronic and thermal Enthalpies=	-4084.965174
Sum of electronic and thermal Free Energies=	-4085.022728

cartesian

6	-1.30813241	2.12246466	-0.66419411	1	3.37846780	1.25706482	-0.04809412
6	-0.01893239	2.72636461	-0.69659412	40	0.36096761	0.28086478	-0.27119413
6	0.54806763	2.60826468	0.59270591	6	-1.38443244	-1.72543514	0.29610586
6	-0.39403236	1.94616485	1.43370593	6	-1.71703243	-1.07723522	-0.86249411
6	-1.54393244	1.67076480	0.66010588	6	-0.88513237	-1.01093519	-2.05249405
1	-2.44833231	1.20066476	1.02490592	1	-0.58613241	-2.46323538	0.25650588
1	-2.00643229	2.07846475	-1.49149406	1	-0.28503239	-1.89573514	-2.26369405
1	0.43846762	3.20656466	-1.55189407	1	-1.40103245	-0.64363521	-2.93829417
1	1.51676762	2.98296475	0.89810592	1	2.24756765	0.48836482	2.27800584
6	1.89066756	-1.72643518	-0.19949412	1	-0.27253240	1.73666477	2.48960590
6	2.63666773	-0.69933522	-0.82209408	1	0.16536760	0.05756479	-2.23519421
6	2.83526778	0.33676481	0.12700588	1	0.88926762	0.74726480	-2.06749415
6	2.24136758	-0.07093520	1.35080588	1	-2.60943222	-0.45383519	-0.85059410
6	1.65026760	-1.34053516	1.15210593	6	-2.25353241	-1.74493515	1.52250588
1	1.15006757	-1.93013513	1.91050589	1	-1.68123245	-1.52443516	2.42770600
1	1.60786760	-2.66683531	-0.65709412	1	-2.68523240	-2.74223542	1.64990592
1	2.99916768	-0.69993520	-1.84229410	1	-3.07573223	-1.03143525	1.44440591

I-3

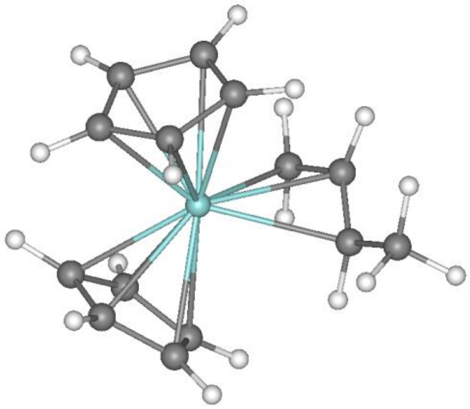

Zero-point vibrational energy 707637.1 (Joules/Mol)

169.12933 (Kcal/Mol)

Zero-point correction= 0.269525 (Hartree/Particle)

Thermal correction to Energy= 0.284510

Thermal correction to Enthalpy= 0.285454

Thermal correction to Gibbs Free Energy= 0.227267

Sum of electronic and zero-point Energies= -4083.836596

Sum of electronic and thermal Energies= -4083.821611

Sum of electronic and thermal Enthalpies= -4083.820667

Sum of electronic and thermal Free Energies= -4083.878854

cartesian

6	-1.31758118	1.99387813	-0.90163124	1	2.74901867	-0.51732188	-2.07753134
6	-0.03578123	2.59907818	-0.82313126	1	3.14261866	1.55257821	-0.40253124
6	0.35321876	2.62687826	0.54226875	40	0.29631877	0.22127812	-0.13903123
6	-0.67338121	2.01577806	1.30446875	1	-2.54138136	-0.65292192	-1.09473121
6	-1.71018124	1.62957811	0.41186875	6	-1.28668118	-1.78422189	0.17986877
1	-2.64968109	1.17317820	0.69686878	6	-1.59128118	-1.18372178	-1.03123116
1	-1.89678133	1.85457814	-1.80553126	6	-0.61878121	-0.92382187	-2.03413129
1	0.53221881	2.99967813	-1.65503120	1	-0.44398123	-2.47582173	0.19726877
1	1.27141881	3.04617810	0.93356878	1	0.13121878	-1.68912184	-2.22103119
6	2.07591867	-1.60772181	-0.24953124	1	-0.93958127	-0.38612187	-2.92163134
6	2.55641890	-0.51462191	-1.01123118	1	2.46281862	0.75147808	2.08506870
6	2.75551891	0.57877815	-0.12873124	1	-0.68008125	1.89567816	2.38206863
6	2.39881897	0.15627812	1.18226874	6	-2.26518106	-1.90562177	1.32006872
6	1.98031878	-1.19712186	1.10446870	1	-1.79118121	-1.71082187	2.28656864
1	1.66541874	-1.81532180	1.93766880	1	-2.66938114	-2.92162180	1.36446869
1	1.84301877	-2.59342194	-0.63363123	1	-3.10338116	-1.21552181	1.20146871

I-4pp

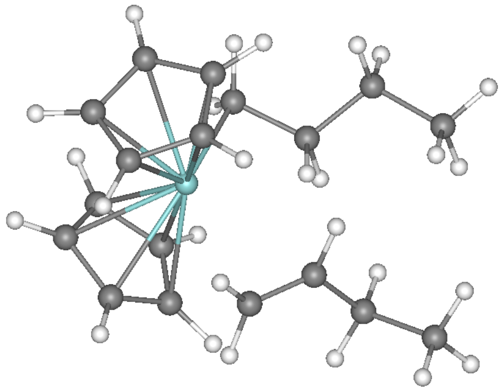

Zero-point vibrational energy 1066924.1 (Joules/Mol)

255.00097 (Kcal/Mol)

Zero-point correction= 0.406370 (Hartree/Particle)

Thermal correction to Energy= 0.427875

Thermal correction to Enthalpy= 0.428819

Thermal correction to Gibbs Free Energy= 0.356248

Sum of electronic and zero-point Energies= -4242.064461

Sum of electronic and thermal Energies= -4242.042956

Sum of electronic and thermal Enthalpies= -4242.042012

Sum of electronic and thermal Free Energies= -4242.114583

cartesian							
6	-1.13041949	-2.18315434	1.29257178	6	0.73448044	1.21644568	1.28717184
6	-2.20881963	-2.29235435	0.36767179	1	-1.02631950	1.45084572	2.56047177
6	-3.22101951	-1.39325428	0.78277177	1	-0.89991951	2.63454556	1.23827183
6	-2.75811958	-0.69845438	1.92747176	1	0.84788042	0.59374565	0.34037179
6	-1.47111952	-1.20485425	2.24927163	6	0.26508045	-1.90605426	-1.59152818
1	-0.85471952	-0.88965434	3.08187175	6	1.31588054	-1.16865432	-1.18062818
1	-0.21211952	-2.75745440	1.27957177	6	2.33648062	-1.64095426	-0.18192823
1	-2.27771950	-2.98015451	-0.46572822	1	-0.33521953	-1.63145435	-2.45302820
1	-3.30001950	0.06064566	2.47727180	1	0.09368044	-2.89345431	-1.17022824
6	-1.17771959	0.90874565	-2.36262822	1	1.53768063	-0.23275433	-1.70022821
6	-2.39941955	0.20784566	-2.20712829	1	2.46238041	-0.91015434	0.62357175
6	-3.19531941	0.93554562	-1.27762818	1	2.00128055	-2.57885432	0.27117178
6	-2.45441961	2.05844545	-0.84402823	1	-4.20981979	0.70004565	-0.98572826
6	-1.19911957	2.03544569	-1.50772822	1	-4.18861961	-1.27285433	0.31467178
1	-0.40891954	2.76894546	-1.40112817	1	1.11618042	0.54444563	2.06367159
1	-0.36501953	0.63614565	-3.02582836	6	3.10498047	1.91944563	0.63787174
1	-2.70211959	-0.68585438	-2.73942828	1	3.77968073	2.76994562	0.52077174
1	-2.79581952	2.80984569	-0.14512824	1	3.07608056	1.38934565	-0.32132822
40	-1.26951957	-0.00315434	0.01357177	1	3.54328060	1.24864566	1.38317180
6	1.71118045	2.37874556	1.05697179	6	3.69058037	-1.84905434	-0.87802827
1	1.29238057	3.05054545	0.30047178	1	4.43428040	-2.19595432	-0.15862824
1	1.76248050	2.95424557	1.98577178	1	4.05408049	-0.91565436	-1.31602824
6	-0.70691955	1.60324574	1.53177178	1	3.60828066	-2.59165454	-1.67452824

I-4ps

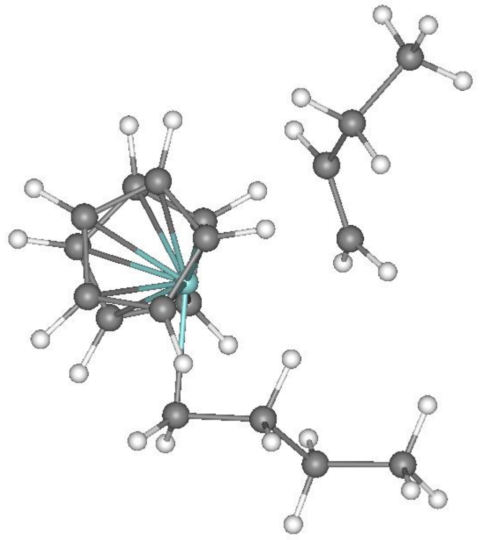

Zero-point vibrational energy	1065197.8 (Joules/Mol)
	254.58839 (Kcal/Mol)
Zero-point correction=	0.405712 (Hartree/Particle)
Thermal correction to Energy=	0.426456
Thermal correction to Enthalpy=	0.427400
Thermal correction to Gibbs Free Energy=	0.357211
Sum of electronic and zero-point Energies=	-4242.063811
Sum of electronic and thermal Energies=	-4242.043068
Sum of electronic and thermal Enthalpies=	-4242.042124
Sum of electronic and thermal Free Energies=	-4242.112313

cartesian

6	-0.96328700	-0.86793905	-2.08737397	6	1.86171305	1.32086086	-0.89157391
6	-1.26568699	-2.03503895	-1.33207393	1	2.95131302	-0.42053905	-1.65477395
6	-0.13538697	-2.88793898	-1.38657391	1	3.29831314	-0.00233907	0.03772607
6	0.87221301	-2.24213910	-2.14517403	1	0.75441301	1.30026090	-0.64547390
6	0.35241303	-0.99923909	-2.58637404	6	-2.18358707	0.38396093	0.73392612
1	0.87681305	-0.27253905	-3.19407392	6	-1.17978692	1.28356087	0.77452612
1	-1.62598693	-0.02973905	-2.26517391	1	-1.16708696	2.10706115	0.06332608
1	-2.20298719	-2.26643896	-0.84187388	1	-2.25248718	-0.38283905	1.50772607
1	1.85631299	-2.63673902	-2.36377406	6	-3.34878683	0.47316095	-0.20707393
6	0.21421303	-1.09773910	2.48492599	1	-0.47978699	1.34366095	1.60622609
6	0.00441302	-2.36943913	1.90632606	1	1.44911301	-3.76863885	0.91372609
6	1.25301301	-2.80973887	1.37482607	1	-0.06658697	-3.87293887	-0.94457388
6	2.21341324	-1.80203903	1.61072600	1	1.83291304	1.63116086	-1.94127393
6	1.56481302	-0.72553909	2.27402616	6	1.72181308	3.77126122	-0.18637392
1	2.03621292	0.18896094	2.61432600	1	2.19051313	4.55076122	0.41752607
1	-0.52858698	-0.50863904	3.00992608	1	0.67311305	3.70406103	0.12072608
1	-0.91988695	-2.93443894	1.91392601	1	1.74511302	4.09916115	-1.22937393
1	3.25991297	-1.85473907	1.34562600	6	-4.61348677	0.85376096	0.58252609
40	0.53221303	-0.87883902	-0.04627392	1	-3.52008677	-0.48553905	-0.70637387
6	2.44381285	2.43726110	-0.01677392	1	-3.15198708	1.22426093	-0.97707391
1	2.41761303	2.12226105	1.03332603	1	-5.47198677	0.90516090	-0.08897392
1	3.50071287	2.54796100	-0.27697393	1	-4.49188709	1.82636082	1.06342602
6	2.52541304	-0.03653905	-0.72967392	1	-4.83248663	0.11336097	1.35572600

I-4sp

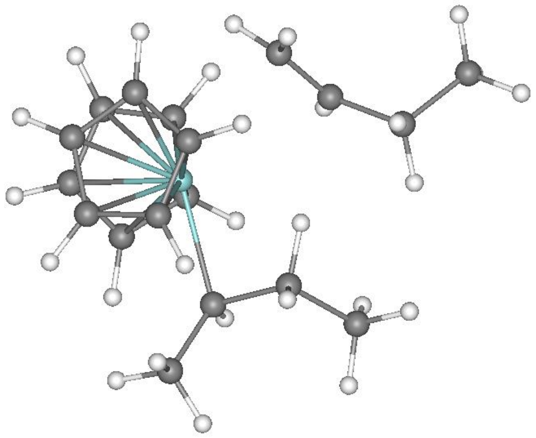

Zero-point vibrational energy 1064985.4 (Joules/Mol)

254.53761 (Kcal/Mol)

Zero-point correction= 0.405632 (Hartree/Particle)

Thermal correction to Energy= 0.427438

Thermal correction to Enthalpy= 0.428382

Thermal correction to Gibbs Free Energy= 0.355317

Sum of electronic and zero-point Energies= -4242.061697

Sum of electronic and thermal Energies= -4242.039891

Sum of electronic and thermal Enthalpies= -4242.038946

Sum of electronic and thermal Free Energies= -4242.112012

cartesian

6	-0.74049342	-0.22657827	2.56087613	6	0.42900655	1.99022174	0.03117613
6	-1.37999344	-1.46457827	2.26637626	1	-0.67459345	1.64072168	-1.75012386
6	-2.62489343	-1.17447829	1.66487610	1	0.92830658	1.04532170	0.39107615
6	-2.75189328	0.23702174	1.56297612	6	1.38330650	-1.96627831	0.83497614
6	-1.59859347	0.81612170	2.14307618	6	2.10270667	-1.22327828	-0.03032387
1	-1.40769351	1.87352180	2.25887609	6	3.15630674	-0.23577826	0.38197613
1	0.21260658	-0.09737827	3.05997610	1	0.78930652	-2.81767821	0.51007611
1	-1.00279343	-2.45397830	2.49487615	1	1.52380657	-1.84397829	1.90637612
1	-3.59199333	0.77502167	1.13977611	1	2.03340673	-1.43387830	-1.09902382
6	-0.52679348	-2.22637820	-1.78032386	1	3.11220670	0.65452170	-0.25122386
6	-1.74299347	-2.49937820	-1.11792386	1	2.99550676	0.07672173	1.41867614
6	-2.64289331	-1.43237829	-1.41332388	1	-3.67549324	-1.36207831	-1.09572387
6	-1.97089350	-0.50497830	-2.23912382	1	-3.35799336	-1.90367830	1.34577620
6	-0.64729345	-0.97967827	-2.44372392	1	0.26080653	2.55202174	0.95417613
1	0.11320657	-0.50347829	-3.05232382	6	4.54410648	-0.88427830	0.23427613
1	0.34740654	-2.86657834	-1.78432381	1	5.32240629	-0.16887826	0.50547612
1	-1.96899343	-3.38267827	-0.53312385	1	4.72160625	-1.20277822	-0.79582387
1	-2.39739347	0.40032175	-2.65022397	1	4.63370657	-1.75807834	0.88287616
40	-0.86419344	-0.43867826	0.03567613	6	-1.97569346	2.76202178	-0.48172385
6	1.46300650	2.70492172	-0.84632385	1	-2.89329338	2.48612165	-1.01032388
1	1.72290647	2.09912181	-1.72002387	1	-1.65849352	3.73532176	-0.87622386
1	1.04560649	3.64762163	-1.20982385	1	-2.23219347	2.90992165	0.57037616
6	-0.89179349	1.71172166	-0.67912388	1	2.37810659	2.93352175	-0.29442388

I-4ss

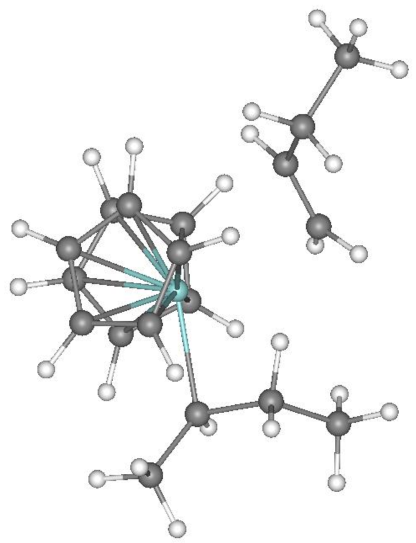

Zero-point vibrational energy	1066737.4 (Joules/Mol)
	254.95635 (Kcal/Mol)
Zero-point correction=	0.406299 (Hartree/Particle)
Thermal correction to Energy=	0.427776
Thermal correction to Enthalpy=	0.428720
Thermal correction to Gibbs Free Energy=	0.357425
Sum of electronic and zero-point Energies=	-4242.061138
Sum of electronic and thermal Energies=	-4242.039661
Sum of electronic and thermal Enthalpies=	-4242.038716
Sum of electronic and thermal Free Energies=	-4242.110011

cartesian

6	0.67319781	-0.01255652	-2.22748470	6	-1.29440212	-2.07295656	0.04171524
6	0.84199780	1.37774348	-1.97778475	1	-2.60480237	-0.79905653	1.14601517
6	-0.43050218	1.98954356	-2.07058477	1	-0.22770220	-1.75775647	-0.13668476
6	-1.38960218	0.98184347	-2.35218477	6	2.44369769	0.03374347	0.59121525
6	-0.70030224	-0.24865654	-2.46878457	6	1.64999783	-0.86565650	1.20841515
1	-1.14290214	-1.20795655	-2.70158458	1	1.69179785	-1.91555643	0.92491525
1	1.45779788	-0.75895655	-2.25828457	1	2.46879768	1.06344342	0.95351523
1	1.77569783	1.89424348	-1.78978479	6	3.45909762	-0.31975654	-0.45518476
1	-2.45480227	1.13284349	-2.47848463	1	1.09679782	-0.62715650	2.11491537
6	0.08519779	1.84184349	2.09411526	1	-2.04070210	3.29954338	-0.02338476
6	-0.14790221	2.77424359	1.05941522	1	-0.63010222	3.04744339	-1.96588480
6	-1.52620220	2.68434358	0.70321524	1	-1.49720216	-2.66895652	-0.85418475
6	-2.12590218	1.68954349	1.50651515	6	4.86989784	-0.22285655	0.15141524
6	-1.12180221	1.14574349	2.35081530	1	3.39179778	0.36604348	-1.30548477
1	-1.26860213	0.37554348	3.09961534	1	3.28079772	-1.33415651	-0.82368475
1	1.02579784	1.68964350	2.61071539	1	5.61999798	-0.44945654	-0.60798472
1	0.57529777	3.46664357	0.64601523	1	4.98659801	-0.92985654	0.97511524
1	-3.16980219	1.40624344	1.48661518	1	5.06699800	0.78234351	0.53201526
40	-0.48710221	0.52944350	-0.03918476	6	-3.47180223	-0.97825646	-0.80018473
6	-1.26790214	-2.95685649	1.29151523	1	-4.08440208	-0.07375652	-0.73598474
1	-1.01400220	-2.37075639	2.18111539	1	-4.11160231	-1.82285643	-0.51678479
1	-2.25360227	-3.39795661	1.45781517	1	-3.19820237	-1.12135649	-1.84908485
6	-2.25640225	-0.88945651	0.11341524	1	-0.54300219	-3.76945639	1.19851518

TS-44pp

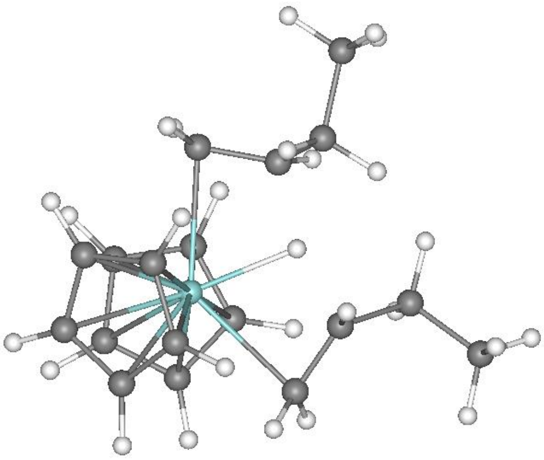

Zero-point vibrational energy 1057299.8 (Joules/Mol)

252.70071 (Kcal/Mol)

Zero-point correction= 0.402704 (Hartree/Particle)
Thermal correction to Energy= 0.423186
Thermal correction to Enthalpy= 0.424130
Thermal correction to Gibbs Free Energy= 0.354927
Sum of electronic and zero-point Energies= -4242.041794
Sum of electronic and thermal Energies= -4242.021313
Sum of electronic and thermal Enthalpies= -4242.020368
Sum of electronic and thermal Free Energies= -4242.089571

cartesian

6	1.11193919	1.96889782	1.63639784	1	-1.45756078	-0.41500217	1.73959780
6	2.27643919	2.11419797	0.83039784	1	0.53123921	-1.68970215	2.32829785
6	3.14853930	1.04749787	1.13959789	1	0.28073919	-2.77140212	0.86919785
6	2.51793933	0.21929783	2.10579777	1	-0.84306079	-0.00000218	-0.00000217
6	1.27003920	0.81369781	2.42899776	1	-2.80406070	-1.07440209	-0.31190217
1	0.55843920	0.44699782	3.15879774	6	0.10193920	1.80989790	-1.34130216
1	0.27073920	2.64959788	1.66799784	6	-1.07796073	1.12799788	-1.00260210
1	2.48333907	2.91999793	0.13809782	6	-2.15206075	1.80929780	-0.16950217
1	2.93903923	-0.67310220	2.55189776	1	0.53123921	1.68969786	-2.32830215
6	1.27003920	-0.81370223	-2.42900205	1	0.28073919	2.77139783	-0.86910212
6	2.51793933	-0.21930218	-2.10580206	1	-1.45756078	0.41499782	-1.73960209
6	3.14853930	-1.04750216	-1.13960218	1	-2.80406070	1.07439780	0.31189781
6	2.27643919	-2.11420226	-0.83040214	1	-1.68166065	2.39579797	0.62469786
6	1.11193919	-1.96890211	-1.63640213	1	4.13633919	-0.90340221	-0.72350216
1	0.27073920	-2.64960217	-1.66800213	1	4.13633919	0.90339780	0.72349787
1	0.55843920	-0.44700217	-3.15880203	6	-2.99176073	-2.72510219	1.06859791
1	2.93913937	0.67309779	-2.55190206	1	-2.37056065	-3.49870205	1.52549791
1	2.48333907	-2.92000222	-0.13810217	1	-3.77636099	-3.21180224	0.48639783
40	1.15343916	-0.00000218	-0.00000217	1	-3.47006083	-2.15610218	1.86999786
6	-2.15206075	-1.80930209	0.16949782	6	-2.99176073	2.72509789	-1.06860220
1	-1.68166065	-2.39580226	-0.62470216	1	-2.37046075	3.49869776	-1.52550220
6	0.10193920	-1.80990219	1.34129786	1	-3.77626085	3.21189785	-0.48640218
6	-1.07796073	-1.12800217	1.00259781	1	-3.47006083	2.15609789	-1.87000215

TS-44ps

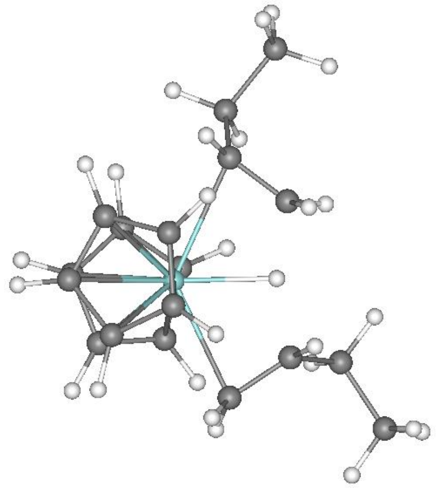

Zero-point vibrational energy	1056121.2 (Joules/Mol)
	252.41902 (Kcal/Mol)
Zero-point correction=	0.402255 (Hartree/Particle)
Thermal correction to Energy=	0.422990
Thermal correction to Enthalpy=	0.423934
Thermal correction to Gibbs Free Energy=	0.354123
Sum of electronic and zero-point Energies=	-4242.034531
Sum of electronic and thermal Energies=	-4242.013797
Sum of electronic and thermal Enthalpies=	-4242.012852
Sum of electronic and thermal Free Energies=	-4242.082664

cartesian

6	1.04344141	-1.78280008	2.06298709	1	-2.11485863	0.26599994	1.81568706
6	1.82744133	-2.34019995	1.01608706	1	-2.55615854	-1.82430005	0.70718700
6	0.99344140	-3.22140002	0.28188697	1	-2.81715870	-0.96510005	-0.88641298
6	-0.30585861	-3.17129993	0.83668703	1	-0.64265859	0.69789994	0.74868703
6	-0.26765859	-2.28489995	1.94918704	1	-2.09925866	2.43409991	0.55328703
1	-1.09535861	-2.05069995	2.60838699	6	1.84484136	0.87309992	0.68188703
1	1.39244139	-1.09870005	2.82708693	6	0.63174140	1.35949993	1.18968701
1	2.88304138	-2.17090011	0.84298700	1	2.42714143	0.23799995	1.34338701
1	-1.15985870	-3.74600005	0.50148702	1	0.26914138	2.31509995	0.80748701
6	0.26484138	0.52959991	-2.26921296	1	1.66914129	-2.49300003	-2.44311309
6	1.42824137	-0.29520005	-2.23361301	1	1.29794133	-3.84060001	-0.55101299
6	1.01254141	-1.63490009	-2.39191294	6	-4.13225842	1.72979987	0.37358698
6	-0.40375859	-1.65440011	-2.50291300	1	-4.69065857	0.91789997	-0.09781301
6	-0.85155863	-0.31040007	-2.46311307	1	-4.49445820	2.67709994	-0.02991302
1	-1.87535870	0.01919994	-2.57701302	1	-4.34955835	1.71379983	1.44458699
1	0.23914139	1.61159980	-2.21981311	6	2.68154144	1.72899997	-0.24511302
1	2.45284128	0.03869995	-2.17111301	1	2.06614137	2.18199992	-1.02761304
1	-1.02365863	-2.53099990	-2.64661288	1	3.44704127	1.12259996	-0.73451298
40	0.13234138	-0.88670003	-0.15721302	6	3.37864137	2.83690000	0.56068701
6	-2.62735868	1.58139992	0.11648699	1	2.64724135	3.50270009	1.02618706
1	-2.42735863	1.58959997	-0.95901299	1	4.02134132	3.43530011	-0.08861301
6	-2.35665870	-0.95350003	0.09538699	1	3.99974132	2.41120005	1.35278702
6	-2.12945867	0.27989992	0.72268701	1	0.36954141	1.16399992	2.22918701

TS-44sp

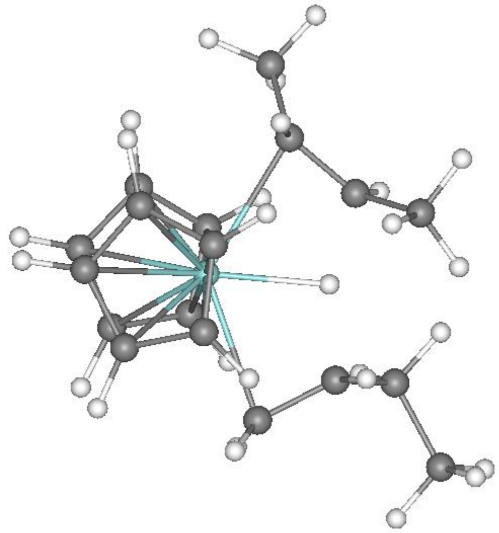

Zero-point vibrational energy	1054671.2 (Joules/Mol)
	252.07246 (Kcal/Mol)
Zero-point correction=	0.401703 (Hartree/Particle)
Thermal correction to Energy=	0.422511
Thermal correction to Enthalpy=	0.423456
Thermal correction to Gibbs Free Energy=	0.354149
Sum of electronic and zero-point Energies=	-4242.037165
Sum of electronic and thermal Energies=	-4242.016356
Sum of electronic and thermal Enthalpies=	-4242.015412
Sum of electronic and thermal Free Energies=	-4242.084718

cartesian

6	-1.60845208	1.03785646	-2.12395000	1	1.95194769	0.26445648	-1.57985008
6	-2.75705218	0.57785648	-1.42115009	1	0.88714784	-1.82434356	-2.12644982
6	-2.83185220	-0.82424355	-1.60195005	1	1.35474777	-2.52784348	-0.50305003
6	-1.70885205	-1.23894346	-2.35885000	1	0.92714781	0.56365651	-0.02185002
6	-0.96415216	-0.07474352	-2.69614983	1	3.07174778	0.59935647	0.65994996
1	-0.06505215	-0.04394351	-3.30014992	6	-0.92995214	1.99015641	0.75704998
1	-1.30065215	2.06985641	-2.22994995	6	0.46264786	1.87835646	0.59264994
1	-3.47905231	1.19275653	-0.89815003	6	1.19914794	2.71275640	-0.44245002
1	-1.49225211	-2.25314355	-2.67024994	1	-1.44175220	2.51385641	-0.04655002
6	-0.54085213	-0.90824354	2.33715010	1	1.02864790	1.69105649	1.50924993
6	-1.93475223	-0.82654351	2.05855012	1	2.22014785	2.36605644	-0.61685002
6	-2.27215219	-1.92184353	1.23025000	1	0.66744787	2.73425651	-1.39585006
6	-1.09235215	-2.66024351	0.96155000	1	-3.26775217	-2.16794348	0.88814998
6	-0.03195214	-2.04314351	1.67394996	1	-3.62295222	-1.46824348	-1.24384999
1	0.99004787	-2.39454341	1.71634996	6	4.22764826	-0.93614352	-0.32665002
1	0.03024787	-0.24874352	2.97925019	1	4.11884785	-1.97004354	-0.66135001
1	-2.63255215	-0.10614352	2.46025014	1	5.03304815	-0.89394349	0.40904999
1	-1.02385211	-3.56064343	0.36354998	1	4.52404785	-0.33074352	-1.18704998
40	-0.79885215	-0.40514350	-0.15275002	1	1.25694776	3.73915648	-0.06865002
6	2.92044783	-0.41774350	0.28494999	6	-1.55185223	2.23745656	2.11475015
1	2.63614774	-1.03514349	1.14154994	1	-1.07405210	1.67365646	2.91735005
6	1.15514779	-1.63974357	-1.09364998	1	-2.62275219	2.02685642	2.12005019
6	1.81814790	-0.44824353	-0.76185006	1	-1.43575215	3.30095649	2.35365009

TS-45pp

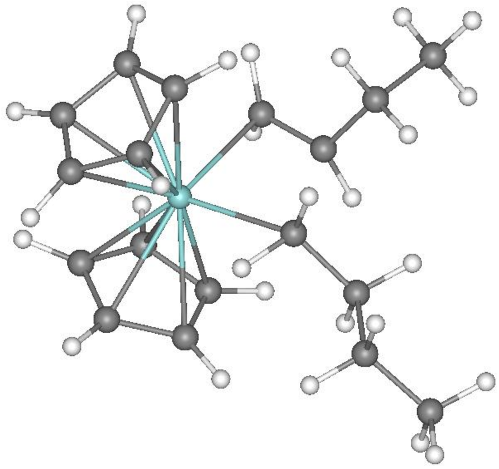

Zero-point vibrational energy 1064953.8 (Joules/Mol)

254.53006 (Kcal/Mol)

Zero-point correction= 0.405619 (Hartree/Particle)

Thermal correction to Energy= 0.426191

Thermal correction to Enthalpy= 0.427135

Thermal correction to Gibbs Free Energy= 0.356217

Sum of electronic and zero-point Energies= -4242.053111

Sum of electronic and thermal Energies= -4242.032540

Sum of electronic and thermal Enthalpies= -4242.031595

Sum of electronic and thermal Free Energies= -4242.102514

cartesian

6	-0.64325875	-1.62509561	2.02947402	6	2.12654138	-0.70929563	-0.23982611
6	-0.58155870	-2.48299551	0.90697390	6	2.77844119	-1.60429561	-1.30152619
6	-1.89845872	-2.65659547	0.40837389	1	0.22854125	-0.97259563	-1.17842615
6	-2.78085876	-1.92839563	1.25257385	1	0.95074129	0.59470433	-1.52352619
6	-2.00705862	-1.28979564	2.25027394	6	4.12694120	-2.15409565	-0.83932608
1	-2.39375877	-0.67489564	3.05347395	1	2.10264134	-2.43509555	-1.54062617
1	0.19684130	-1.30899560	2.63677406	1	2.90424132	-1.03189564	-2.22712612
1	0.31474125	-2.92929554	0.49197388	6	-0.74955875	1.55680442	1.46617389
1	-2.18415880	-3.27699566	-0.43192610	6	0.54994130	1.38140440	0.94117391
1	-3.85895872	-1.89009559	1.16257381	6	1.21394122	2.51380444	0.19007388
6	-1.86325872	1.53300440	-1.72462618	1	-0.93655872	1.18860447	2.47027397
6	-1.95565867	0.28390434	-2.38622594	1	-1.26165867	2.47960448	1.20777380
6	-3.05125880	-0.42689565	-1.83822620	1	1.22584128	0.75270438	1.51857388
6	-3.65535879	0.39980435	-0.84932613	1	2.04374123	2.15880442	-0.42512611
6	-2.92635870	1.61110437	-0.78562611	1	0.48724127	2.99590445	-0.47072610
1	-3.16325879	2.45660448	-0.15362611	6	1.74354124	3.53490448	1.20867383
1	-1.13705873	2.30830431	-1.93342614	1	1.98644137	-1.28509557	0.68437392
1	-1.29835868	-0.07179564	-3.17102599	1	4.56934118	-2.79989552	-1.60022616
1	-3.39385867	-1.40679562	-2.14642596	1	4.01914120	-2.74129558	0.07717388
1	-4.53985882	0.16130435	-0.27232611	1	4.83194113	-1.34329557	-0.63702613
40	-1.38945866	-0.23329563	0.03237388	1	2.24924135	4.35130405	0.69007391
1	2.82184124	0.10230436	0.00657388	1	2.46204138	3.07240438	1.89047384
6	0.80494130	-0.11409564	-0.70462608	1	0.93074131	3.95560431	1.80467379

TS-45ps

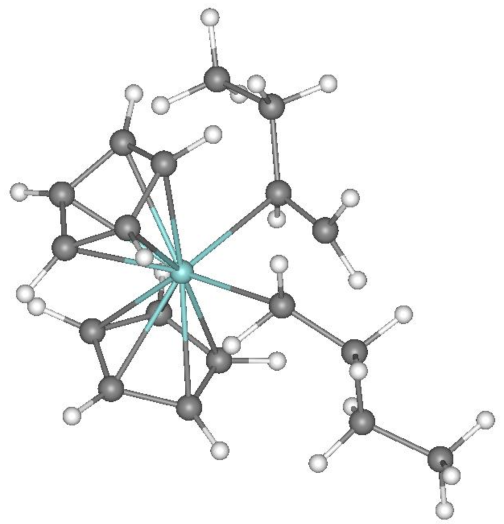

Zero-point vibrational energy	1067303.0 (Joules/Mol)
	255.09154 (Kcal/Mol)
Zero-point correction=	0.406514 (Hartree/Particle)
Thermal correction to Energy=	0.426734
Thermal correction to Enthalpy=	0.427679
Thermal correction to Gibbs Free Energy=	0.358769
Sum of electronic and zero-point Energies=	-4242.040298
Sum of electronic and thermal Energies=	-4242.020078
Sum of electronic and thermal Enthalpies=	-4242.019134
Sum of electronic and thermal Free Energies=	-4242.088044

cartesian

6	0.57700217	-1.01192403	2.07597828	6	2.67000222	0.42967600	-1.27762175
6	0.98290229	-2.06912398	1.23297822	6	3.48700213	-0.18782401	-0.14462173
6	-0.15289778	-2.87442398	0.95167828	1	1.11640227	-1.06952405	-1.35772181
6	-1.25439775	-2.33092403	1.66587818	1	0.77670228	0.41697598	-2.28192186
6	-0.80999780	-1.17442393	2.34857821	6	-0.69279778	1.63777590	0.79457825
1	-1.41619778	-0.54572403	2.98937821	6	0.48940220	1.69477606	0.05067827
1	1.21660221	-0.23212400	2.47197819	1	0.51790226	2.30357599	-0.85082173
1	1.98520231	-2.24412394	0.86657828	1	-0.55239779	1.42437601	1.85297823
1	-0.15969779	-3.77952385	0.35707828	6	-1.86509776	2.56727600	0.51287830
1	-2.25519776	-2.74102402	1.69827819	1	1.44390225	1.56157589	0.54937828
6	-1.95169771	0.17917600	-2.14242172	1	3.52810216	-1.27392399	-0.28652173
6	-1.48719776	-1.11162400	-2.49042177	6	4.91110182	0.36687601	-0.10602173
6	-2.11899781	-2.05482411	-1.64232183	1	2.99930215	-0.01352400	0.82317829
6	-3.00369763	-1.34342408	-0.78642172	1	-1.51569772	3.59367609	0.67457825
6	-2.90259767	0.03187600	-1.09792173	6	-3.04199767	2.31227612	1.45517826
1	-3.49059772	0.82137603	-0.65232170	1	-2.16659784	2.52247596	-0.53722173
1	-1.66559780	1.11047602	-2.61712170	1	-3.89649773	2.94437599	1.20467818
1	-0.76269782	-1.34362411	-3.26202178	1	-2.75689769	2.53797603	2.48657823
1	-1.98409772	-3.12892389	-1.67242181	1	-3.36999774	1.26747608	1.43147826
1	-3.67129779	-1.77612400	-0.05272173	1	4.90580177	1.44417596	0.08067827
40	-0.65899777	-0.67362404	-0.12682173	1	5.42130184	0.19437599	-1.05742180
1	2.75660229	1.52307606	-1.24482179	1	3.12710214	0.13977599	-2.23232174
6	1.19260228	0.06357598	-1.33652174	1	5.49890184	-0.10872400	0.68127829

TS-45sp

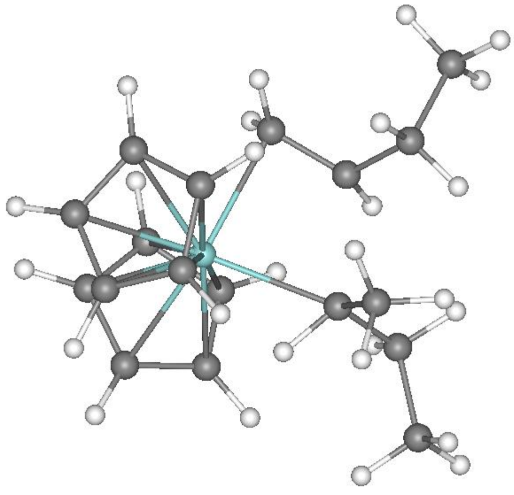

Zero-point vibrational energy 1067302.4 (Joules/Mol)

255.09140 (Kcal/Mol)

Zero-point correction= 0.406514 (Hartree/Particle)

Thermal correction to Energy= 0.426536

Thermal correction to Enthalpy= 0.427480

Thermal correction to Gibbs Free Energy= 0.359982

Sum of electronic and zero-point Energies= -4242.046437

Sum of electronic and thermal Energies= -4242.026415

Sum of electronic and thermal Enthalpies= -4242.025471

Sum of electronic and thermal Free Energies= -4242.092969

cartesian

6	1.20636964	1.76183474	1.79718041	6	-1.58583021	1.90753472	-0.42791957
6	1.58266973	2.33663464	0.56078047	6	-1.69003034	2.93343472	-1.56351960
6	2.82716966	1.77803481	0.17288043	1	0.15366966	1.03563476	-1.28691959
6	3.23776960	0.88353473	1.19798040	6	-1.53823018	-0.08536523	-1.97731960
6	2.23726964	0.86913478	2.19688034	6	-0.15213037	-1.18326521	1.73818040
1	2.27216959	0.30143476	3.11818027	6	-1.23773026	-0.51096523	1.13118041
1	0.31136966	1.99213481	2.36338043	6	-2.40643024	-1.31106520	0.61028045
1	1.02346969	3.07743478	0.00148043	1	0.21416965	-0.78986526	2.68128037
1	3.39016962	2.03303480	-0.71621954	1	-0.14373034	-2.26746535	1.65668046
1	4.16806984	0.33203477	1.22498047	1	-1.49923038	0.46053475	1.54718041
6	0.89476967	-2.33386517	-1.14061952	1	-1.12593031	2.38053465	0.44458044
6	1.39166975	-1.40846527	-2.08961964	1	-2.61553025	0.09203477	-1.93861961
6	2.70326972	-1.03786516	-1.70111954	1	-1.38153028	-1.16526520	-1.97951949
6	3.02756977	-1.76446521	-0.52241957	1	-1.19683027	0.29643476	-2.94561958
6	1.91206956	-2.56186533	-0.17591958	1	-0.69783038	3.25023484	-1.90231955
1	1.85896969	-3.24466515	0.66228044	1	-2.22783017	3.82213473	-1.22671962
1	-0.07343036	-2.81926513	-1.16371953	1	-2.22413039	2.52833486	-2.42591977
1	0.86676967	-1.04466522	-2.96441960	6	-3.21713018	-1.82706523	1.81078041
1	3.35906982	-0.36226523	-2.23611975	1	-2.05173039	-2.15806532	0.01478042
1	3.97406983	-1.74016523	0.00138042	1	-3.05803037	-0.70566523	-0.01991957
40	1.20576966	-0.14296523	0.10238042	1	-4.09423018	-2.37406516	1.45998049
1	-2.59703016	1.61163473	-0.12311958	1	-3.56263018	-0.99816525	2.43388033
6	-0.82233030	0.64783478	-0.83511955	1	-2.61883020	-2.49476528	2.43418026

TS-45ss

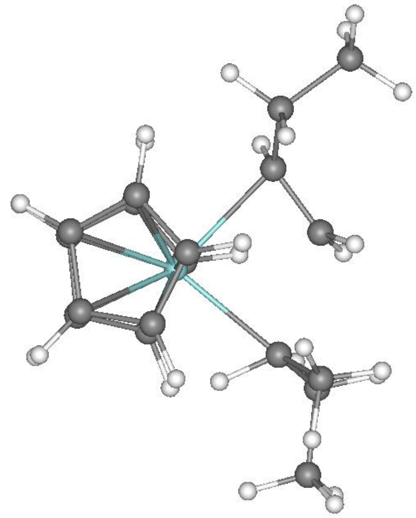

Zero-point vibrational energy	1066331.4 (Joules/Mol)
	254.85931 (Kcal/Mol)
Zero-point correction=	0.406144 (Hartree/Particle)
Thermal correction to Energy=	0.426511
Thermal correction to Enthalpy=	0.427455
Thermal correction to Gibbs Free Energy=	0.358603
Sum of electronic and zero-point Energies=	-4242.043368
Sum of electronic and thermal Energies=	-4242.023002
Sum of electronic and thermal Enthalpies=	-4242.022058
Sum of electronic and thermal Free Energies=	-4242.090909

cartesian

6	0.94766092	-0.96293044	2.30748916	6	2.42146087	1.48466957	0.30708918
6	1.90216088	-1.56743050	1.45608926	6	3.82706094	1.32716954	-0.28841081
6	1.34396088	-2.76593041	0.94378924	1	1.69876099	-0.12893045	-0.91681081
6	0.05046094	-2.91853046	1.51338923	6	1.16486096	1.74336946	-1.85461080
6	-0.19493906	-1.80733049	2.35188913	6	-1.52563906	0.76626956	1.03218925
1	-1.08643901	-1.65003049	2.94558907	6	-0.48773906	1.60166943	0.59838921
1	1.07426095	-0.03993043	2.86078906	1	-0.67903906	2.27456975	-0.23531079
1	2.89106083	-1.18743050	1.22768927	1	-1.46963906	0.47606954	2.07968903
1	1.83726096	-3.46603036	0.28118917	6	-2.94603896	0.97106957	0.52628922
1	-0.61973906	-3.75393033	1.35958922	1	0.25976095	1.93846953	1.30868924
6	-1.43143904	-0.51863045	-2.12801099	1	2.40516090	1.03616953	1.30408919
6	-0.23483907	-1.14043045	-2.55321097	1	1.12876093	2.80456972	-1.59111083
6	-0.19943906	-2.45073032	-2.01181102	1	0.26676095	1.51196957	-2.42731094
6	-1.40053904	-2.64843035	-1.27921081	1	2.02446103	1.61256945	-2.52111101
6	-2.15753913	-1.45583045	-1.34341073	1	4.06556129	0.27426955	-0.47231081
1	-3.13723898	-1.30593050	-0.91091079	1	4.57526112	1.72386944	0.40088919
1	-1.76143909	0.47856954	-2.39191079	1	3.92966104	1.86466944	-1.23331082
1	0.52986091	-0.69883049	-3.18111086	6	-3.59563899	2.16536975	1.23808920
1	0.57866091	-3.18623042	-2.17401099	1	-3.54753900	0.07796955	0.71598923
1	-1.69923902	-3.56113029	-0.78081077	1	-2.95173907	1.14516950	-0.55411077
40	-0.05933906	-0.91983044	-0.02421081	1	-4.62773895	2.29796958	0.90588921
1	2.21766090	2.55196953	0.44628918	1	-3.04723907	3.08796954	1.03038919
6	1.32536089	0.90196955	-0.58941078	1	-3.60573912	2.01606965	2.32098913

I-5ppα

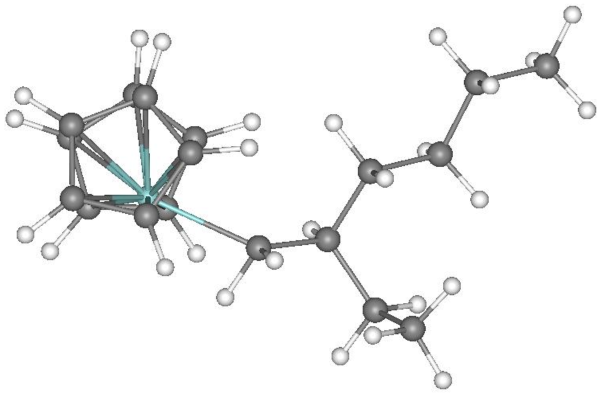

Zero-point vibrational energy 1071068.6 (Joules/Mol)

255.99154 (Kcal/Mol)

Zero-point correction= 0.407948 (Hartree/Particle)

Thermal correction to Energy= 0.428718

Thermal correction to Enthalpy= 0.429662

Thermal correction to Gibbs Free Energy= 0.357099

Sum of electronic and zero-point Energies= -4242.060134

Sum of electronic and thermal Energies= -4242.039364

Sum of electronic and thermal Enthalpies= -4242.038420

Sum of electronic and thermal Free Energies= -4242.110983

cartesian

6	2.78344131	-0.51814568	2.13369989	6	0.51014137	-0.84554565	-0.88810003
6	2.07874131	-1.63544559	1.60859990	6	-1.70355844	-1.02274561	-2.13490009
6	2.93124151	-2.29194570	0.68759996	6	-1.57245851	-0.15174565	0.27719998
6	4.17754126	-1.60334563	0.67100000	1	0.41194141	-1.88164556	-0.54650003
6	4.08984137	-0.51234567	1.56430006	1	1.05724144	-0.90144569	-1.88510013
40	2.46054149	-0.02204566	-0.27010003	1	-2.59345865	-0.43424568	-2.38000011
1	-0.76895863	0.77755433	-1.48320007	1	-1.12165856	-1.08494556	-3.06220007
6	1.24084139	2.16895437	-0.34870002	1	-0.93605858	0.42645434	0.96429998
6	2.36904144	2.32435441	0.50199997	6	-2.96135855	0.48455432	0.23249999
6	3.54254150	2.21475434	-0.29700002	6	-2.13275862	-2.42674565	-1.70799994
6	3.13544154	1.97775435	-1.63269997	1	-2.67585850	-2.91894555	-2.51760006
6	1.71474147	1.94615436	-1.66380000	1	-2.79665852	-2.40084553	-0.83930004
1	2.68864131	-3.18174553	0.11799999	1	-1.27785850	-3.06334567	-1.45970011
1	5.04864120	-1.87784564	0.08559998	1	-3.63725877	-0.13564566	-0.36810002
1	1.06854141	-1.92654562	1.86579990	1	-2.90305853	1.45705438	-0.27580002
1	2.41274142	0.16865434	2.88450003	6	-3.56785846	0.67255431	1.62419987
1	4.87864113	0.19605434	1.78460002	1	-3.62115836	-0.30004567	2.12719989
1	4.56354141	2.31695437	0.04929998	1	-2.90235853	1.29905438	2.23009992
1	2.34114146	2.54515433	1.56159997	6	-4.95915890	1.30155444	1.57240009
1	0.20234138	2.21825433	-0.04510002	1	-5.37925863	1.42355442	2.57309985
1	1.10194135	1.78835440	-2.54380012	1	-5.64745855	0.67915434	0.99359995
1	3.79374146	1.86095440	-2.48720002	1	-4.92465878	2.28785443	1.10030007
6	-0.87535864	-0.24234566	-1.09100008	1	-1.64495850	-1.15604556	0.71639997

I-5ppβ

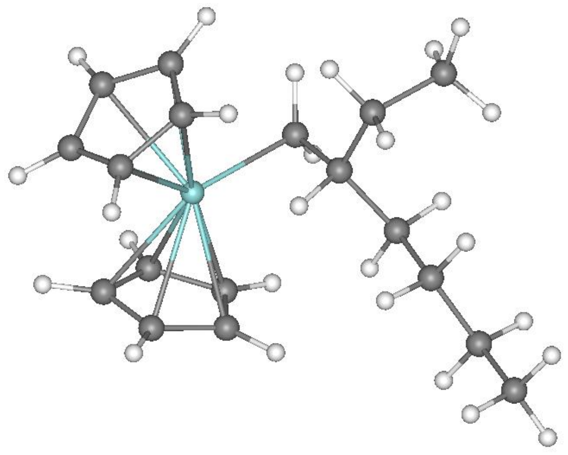

Zero-point vibrational energy	1069255.4 (Joules/Mol)
	255.55818 (Kcal/Mol)
Zero-point correction=	0.407258 (Hartree/Particle)
Thermal correction to Energy=	0.427909
Thermal correction to Enthalpy=	0.428853
Thermal correction to Gibbs Free Energy=	0.356928
Sum of electronic and zero-point Energies=	-4242.079434
Sum of electronic and thermal Energies=	-4242.058783
Sum of electronic and thermal Enthalpies=	-4242.057838
Sum of electronic and thermal Free Energies=	-4242.129764

cartesian

6	-1.86273265	-2.58442163	-0.77045006	6	-0.55303264	0.82247823	1.59654999
6	-0.65123260	-2.42352176	-0.04475001	6	0.79486746	2.26617837	-0.03015001
6	-0.97683263	-2.28432178	1.33095002	6	1.71216750	-0.00902174	0.75724995
6	-2.38793254	-2.35822177	1.45344996	1	-0.23073259	0.24647826	2.46445012
6	-2.93503261	-2.55212164	0.15865000	1	-0.88493264	1.81587815	1.90004992
40	-1.90143251	-0.26272175	0.15005000	1	1.23366737	2.21177840	-1.03205001
1	0.03496742	0.33747825	-0.48435000	1	-0.13963258	2.82907844	-0.12435001
6	-2.32263255	1.65037823	-1.41735005	1	1.39886737	-0.98322177	1.14884996
6	-3.07503247	0.54847825	-1.90815008	6	2.62586737	-0.21682173	-0.44975001
6	-4.09293270	0.26117826	-0.96375000	6	1.73826742	3.02247834	0.90704995
6	-3.95693254	1.16857815	0.11864999	1	1.82406735	4.06387806	0.59094995
6	-2.86763263	2.03587842	-0.16515000	1	2.74366736	2.59467840	0.90154999
1	-0.27763259	-2.17712164	2.14915013	1	1.36876750	3.01127839	1.93664992
1	-2.94913244	-2.30072165	2.37954998	1	3.07336736	0.73777825	-0.75285006
1	0.34796745	-2.42882156	-0.46724999	1	2.03306746	-0.56242174	-1.31095004
1	-1.95143270	-2.73802161	-1.83934999	6	3.73956728	-1.22692180	-0.17005001
1	-3.98463249	-2.67932177	-0.07345001	1	4.31836748	-0.89392173	0.69854999
1	-4.85263252	-0.50372171	-1.06064999	1	3.29286766	-2.18922162	0.10884999
1	-2.92313266	0.04417826	-2.85514998	6	4.66826725	-1.41702175	-1.36785007
1	-1.49303257	2.12577844	-1.92745006	1	5.44806767	-2.15062165	-1.15335000
1	-2.54003263	2.86277843	0.45074999	1	5.15826750	-0.47692174	-1.63575006
1	-4.59253263	1.21037817	0.99624997	1	4.11326742	-1.76542163	-2.24424982
6	0.46736735	0.84777826	0.47885001	1	2.26726747	0.46977824	1.57155001

I-5ppγ

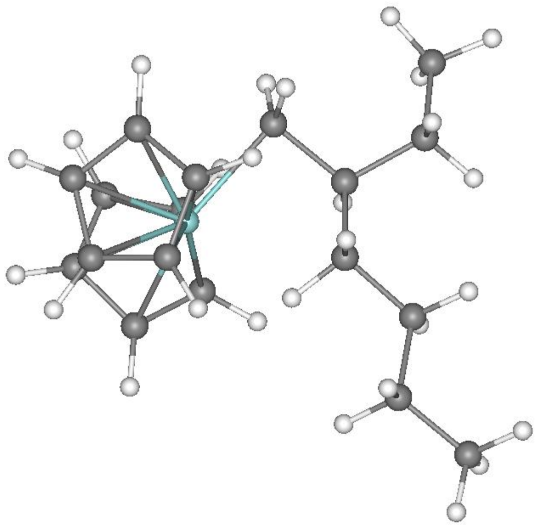

Zero-point vibrational energy 1070858.8 (Joules/Mol)

	255.94140 (Kcal/Mol)
Zero-point correction=	0.407869 (Hartree/Particle)
Thermal correction to Energy=	0.428848
Thermal correction to Enthalpy=	0.429792
Thermal correction to Gibbs Free Energy=	0.356644
Sum of electronic and zero-point Energies=	-4242.074821
Sum of electronic and thermal Energies=	-4242.053841
Sum of electronic and thermal Enthalpies=	-4242.052897
Sum of electronic and thermal Free Energies=	-4242.126046

cartesian

6	1.56395221	0.89554560	-2.33568263	6	-2.49964786	-0.75365436	-0.06118261
6	0.93345213	-0.36845440	-2.44528270	6	-2.57954788	-2.27315426	0.09111739
6	1.90665221	-1.37375438	-2.17508268	1	-0.45444793	-0.97035438	-0.06228261
6	3.13185215	-0.72605437	-1.89688253	1	-1.04424787	-0.29155439	1.47051740
6	2.91745210	0.67744559	-1.97588253	6	0.55205214	1.81214571	0.34591737
1	3.66625214	1.44594562	-1.82398260	6	-0.85654789	1.27724564	-0.05428261
1	1.09885216	1.85884571	-2.49798274	6	-1.96434784	2.18304563	0.53141737
1	-0.10004789	-0.54615438	-2.72148275	1	0.80305213	2.69464564	-0.24418262
1	1.74375224	-2.44525433	-2.20028281	1	0.54665214	2.10074568	1.40231740
1	4.07025194	-1.21435428	-1.66518259	1	-0.95854789	1.31584573	-1.14688253
6	1.70355225	-0.26385438	2.49931741	1	-2.68494773	-0.47155440	-1.10508263
6	1.83245206	-1.59075427	2.00961733	1	-1.82144785	-2.74105430	-0.55158257
6	3.03525209	-1.66935432	1.26651740	6	-3.95984769	-2.82155442	-0.26578259
6	3.65045214	-0.38595438	1.29751742	1	-2.32854772	-2.54725432	1.12301743
6	2.82945228	0.47754562	2.07101727	6	-1.80024791	3.65304565	0.15211739
1	3.02695227	1.51964569	2.28831720	1	-1.97114778	2.07374573	1.62321746
1	0.88945210	0.11524563	3.10521722	1	-2.93444777	1.83234572	0.16771738
1	1.13595212	-2.40435433	2.18051720	1	-2.67134786	4.22624588	0.47601739
1	3.42475224	-2.55295444	0.77601743	1	-1.71244788	3.76914573	-0.93298256
1	4.59965229	-0.12365437	0.84651744	1	-0.91824788	4.09994555	0.61501741
40	1.56925213	-0.13025437	-0.02738261	1	-3.98974776	-3.90835428	-0.16788261
1	-3.28684783	-0.28895438	0.53931737	1	-4.22714806	-2.56895423	-1.29558253
6	-1.14104795	-0.19225436	0.38221738	1	-4.72654819	-2.40455437	0.39231738

I-5psβ

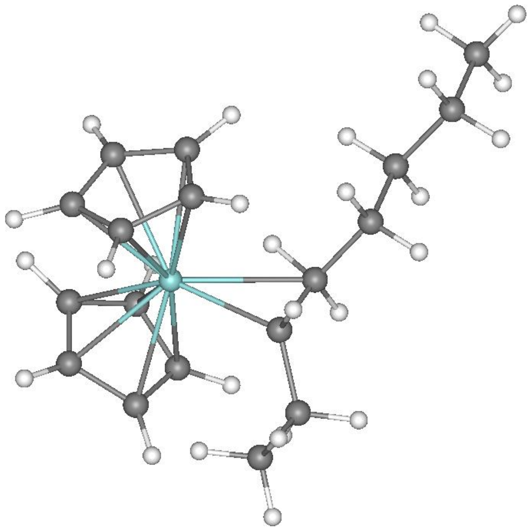

Zero-point vibrational energy	1070901.1 (Joules/Mol)
	255.95151 (Kcal/Mol)
Zero-point correction=	0.407885 (Hartree/Particle)
Thermal correction to Energy=	0.428681
Thermal correction to Enthalpy=	0.429625
Thermal correction to Gibbs Free Energy=	0.357492
Sum of electronic and zero-point Energies=	-4242.076195
Sum of electronic and thermal Energies=	-4242.055398
Sum of electronic and thermal Enthalpies=	-4242.054454
Sum of electronic and thermal Free Energies=	-4242.126588

cartesian

6	-1.08013272	-2.99231744	0.23504564	6	0.68306726	0.91188252	-0.21675438
6	0.02376729	-2.33691740	0.84674567	6	-0.41823271	1.27078259	0.76334566
6	-0.45723271	-1.62581742	1.97764564	6	2.05626726	0.60018253	0.38614562
6	-1.85733271	-1.83771741	2.06174564	1	-0.07563269	1.08568251	1.78544569
6	-2.24173260	-2.68801737	0.99224567	1	1.93686724	-0.11141744	1.21304572
40	-1.47733271	-0.54511744	-0.05985437	6	3.05856729	0.05658257	-0.62965435
1	0.45146728	-0.03011744	-0.84865433	1	3.17276716	0.77508259	-1.45095432
6	-2.12393260	0.87838256	-2.05145431	1	2.66286731	-0.86561739	-1.08085430
6	-2.05963278	-0.47131744	-2.50475430	6	-0.97333270	2.69188261	0.67064565
6	-3.08493280	-1.19761741	-1.85455430	1	-0.15733272	3.40798259	0.83504564
6	-3.76703286	-0.30891743	-0.97755432	1	-1.35583270	2.89968276	-0.33455437
6	-3.18153286	0.98208249	-1.12325430	6	-2.06063271	2.94298267	1.71514571
1	0.13886732	-1.04991746	2.67274570	1	-2.89133286	2.23448277	1.60794568
1	-2.51673269	-1.43551743	2.82304573	1	-2.46813273	3.95318270	1.63654566
1	1.05686736	-2.39401746	0.52114564	1	-1.66073275	2.82188272	2.72574568
1	-1.03783274	-3.63611746	-0.63545436	6	4.42826700	-0.23371744	-0.01475437
1	-3.24323273	-3.05161738	0.79724568	1	4.82276726	0.68548262	0.43184564
1	-3.30693269	-2.24841738	-1.99465430	1	4.31206703	-0.95191741	0.80524564
1	-1.36903262	-0.86251748	-3.24335432	6	5.42226744	-0.77681744	-1.03985429
1	-1.47973275	1.68598258	-2.37745428	1	6.39416742	-0.97321749	-0.58275431
1	0.76346725	1.66098249	-1.01215434	1	5.57406712	-0.06201744	-1.85355437
1	-3.49023271	1.88338244	-0.60765433	1	5.06316710	-1.71261752	-1.47815430
1	-4.62423277	-0.55541742	-0.36205438	1	2.43836713	1.52618253	0.83094567

I-5spβ

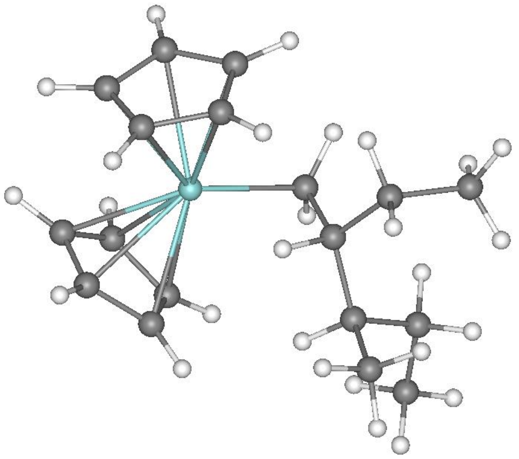

Zero-point vibrational energy 1069167.5 (Joules/Mol)

255.53716 (Kcal/Mol)

Zero-point correction= 0.407224 (Hartree/Particle)

Thermal correction to Energy= 0.427851

Thermal correction to Enthalpy= 0.428795

Thermal correction to Gibbs Free Energy= 0.357495

Sum of electronic and zero-point Energies= -4242.075347

Sum of electronic and thermal Energies= -4242.054720

Sum of electronic and thermal Enthalpies= -4242.053776

Sum of electronic and thermal Free Energies= -4242.125076

cartesian

6	-2.46473026	-2.19953918	-1.12493479	6	0.12246972	0.36896086	1.28886521
6	-1.07043028	-2.34663916	-0.89393479	6	1.11176968	2.07616091	-0.34003478
6	-0.87113029	-2.51353908	0.50326520	6	1.88716984	-0.43433911	-0.40473479
6	-2.13943028	-2.46583915	1.13286519	1	0.56056976	-0.45133913	1.85886526
6	-3.12513018	-2.27853918	0.12896521	1	0.06296974	1.26846087	1.90316522
40	-1.74993026	-0.17673913	0.15316522	1	1.26876974	2.21766090	-1.41443479
1	0.01156974	0.37066084	-0.91413480	1	0.23366970	2.67276096	-0.07623479
6	-2.32383013	2.07346082	-0.79143476	1	1.41006970	-1.41803908	-0.31773478
6	-3.37883019	1.19576097	-1.16203475	6	2.36506963	-0.28273913	-1.85033476
6	-4.05993032	0.80966085	0.02026522	6	3.07606983	-0.45373911	0.58046520
6	-3.41643023	1.43256092	1.12106526	6	3.65566993	-1.86153913	0.73436522
6	-2.34873033	2.22246099	0.62016523	1	3.86236978	0.21716087	0.22386523
1	0.07546973	-2.67833900	1.00086522	1	2.77086973	-0.08013914	1.56306517
1	-2.32523012	-2.57253909	2.19586515	1	2.92236972	-2.54223919	1.17976522
1	-0.29853028	-2.35913920	-1.65583479	1	3.95656991	-2.27973914	-0.23053479
1	-2.94263029	-2.07843924	-2.08973479	6	2.31356978	2.62426090	0.43226522
1	-4.19453049	-2.23603916	0.29066521	1	2.33896971	3.71276093	0.34566522
1	-4.93123055	0.16966085	0.07156521	1	3.25806975	2.24526095	0.04096521
1	-3.64003038	0.90336084	-2.17213488	1	2.25916982	2.37256098	1.49506521
1	-1.63973022	2.56226087	-1.47553480	1	3.05966973	-1.08483911	-2.11193466
1	-1.69593024	2.84886098	1.21336520	1	2.89096975	0.66546082	-1.99703479
1	-3.70813036	1.34376097	2.16166520	1	1.53236961	-0.32093912	-2.56313467
6	0.78156972	0.59636086	-0.05363478	1	4.53666973	-1.85533905	1.37936521

I-5spγ

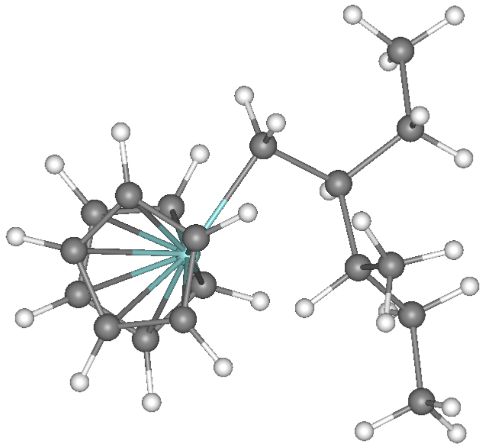

Zero-point vibrational energy	1069055.2 (Joules/Mol)
	255.51033 (Kcal/Mol)
Zero-point correction=	0.407182 (Hartree/Particle)
Thermal correction to Energy=	0.428066
Thermal correction to Enthalpy=	0.429010
Thermal correction to Gibbs Free Energy=	0.357000
Sum of electronic and zero-point Energies=	-4242.074660
Sum of electronic and thermal Energies=	-4242.053776
Sum of electronic and thermal Enthalpies=	-4242.052832
Sum of electronic and thermal Free Energies=	-4242.124842

cartesian

6	1.15657830	2.59237599	-0.15006310	6	-2.18392181	-0.24042389	-1.74776316
6	0.88397825	2.05407596	-1.43136311	6	-1.98592186	-1.17322385	-2.94096303
6	2.09027815	1.49587619	-1.94506311	1	-0.28642178	-0.66542393	-1.03886318
6	3.10217834	1.68937612	-0.97926307	6	-1.60322165	-2.00192404	-0.00646309
6	2.52307820	2.34637594	0.14163691	6	-0.15192175	0.47237611	1.55553687
1	3.04457831	2.64287591	1.04433692	6	-1.36082172	0.49877611	0.57633692
1	0.45147824	3.10097599	0.49423692	6	-2.69432163	0.40757611	1.35143685
1	-0.06482172	2.09397602	-1.95346320	1	-0.11302173	1.40427613	2.12383676
1	2.21577835	1.02907610	-2.91546321	1	-0.26682174	-0.34422389	2.27663684
1	4.13717842	1.38587618	-1.07426310	1	-1.36832178	1.46887612	0.06183691
6	1.69477820	-2.12382388	1.26813686	1	-1.98132181	0.79347610	-2.04816318
6	2.13367820	-2.32952404	-0.06576309	1	-2.68492174	-2.13322401	0.09513691
6	3.29347825	-1.53952384	-0.27266309	1	-1.16232181	-2.16992402	0.97873694
6	3.56497836	-0.84202391	0.93663692	1	-1.23862171	-2.77782393	-0.68396306
6	2.57517815	-1.20732391	1.88793683	1	-0.93622172	-1.19052386	-3.25866318
1	2.50737810	-0.85122389	2.90843678	1	-2.58122182	-0.84142393	-3.79356313
1	0.84507823	-2.59852386	1.73993683	1	-2.28662181	-2.19762397	-2.70986319
1	1.67867827	-2.99432397	-0.79196310	6	-2.92612171	1.60807610	2.26743674
1	3.87997818	-1.49302387	-1.18226314	1	-2.70732164	-0.51312393	1.94373691
1	4.40097809	-0.17712387	1.11493683	1	-3.52412152	0.34067613	0.63923693
40	1.44827819	0.09547611	0.04153691	1	-3.91592193	1.55097616	2.72503686
1	-3.23112178	-0.26902390	-1.43016315	1	-2.87332177	2.54707599	1.70743680
6	-1.30512178	-0.59412390	-0.53706306	1	-2.19372177	1.64997613	3.07693696

I-5ssβ

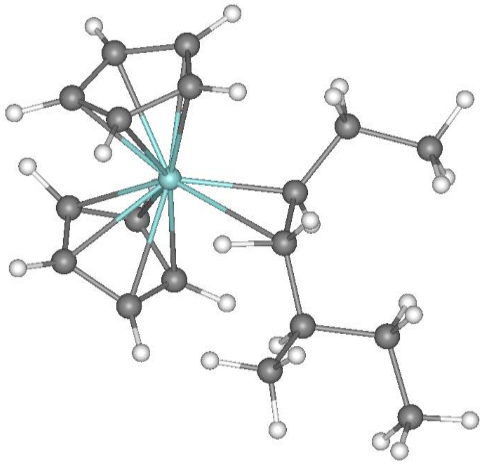

Zero-point vibrational energy 1068502.6 (Joules/Mol)

255.37824 (Kcal/Mol)

Zero-point correction= 0.406971 (Hartree/Particle)

Thermal correction to Energy= 0.427753

Thermal correction to Enthalpy= 0.428697

Thermal correction to Gibbs Free Energy= 0.357061

Sum of electronic and zero-point Energies= -4242.078281

Sum of electronic and thermal Energies= -4242.057499

Sum of electronic and thermal Enthalpies= -4242.056555

Sum of electronic and thermal Free Energies= -4242.128191

cartesian

6	-2.38674355	-2.57104349	-0.14384128	6	0.23705643	0.79995656	0.78665876
6	-0.98744357	-2.59124351	0.10375872	6	1.91845655	-0.68974346	-0.57194126
6	-0.76754355	-2.12124348	1.42605865	1	0.69125646	0.22665651	1.60045874
6	-2.02974343	-1.80954349	1.99245870	1	1.64235640	-1.50204349	0.11485873
6	-3.03064346	-2.09194350	1.02695870	6	2.08635640	-1.27194345	-1.97664130
40	-1.68894362	-0.18504348	0.10475872	6	3.21835661	-0.06394348	-0.04904127
1	-0.02884352	-0.14454348	-1.21894133	6	4.38745642	-1.04774344	0.01685873
6	-2.13434362	1.90345657	-1.25644135	1	3.48745656	0.78455651	-0.69184124
6	-2.76564360	0.82115650	-1.93564129	1	3.03815651	0.34735650	0.94975877
6	-3.80804348	0.33845651	-1.11004126	1	4.11515617	-1.94644344	0.57925874
6	-3.80744338	1.10015655	0.09175873	1	4.71785641	-1.35694349	-0.97694129
6	-2.78184342	2.08265662	-0.01564127	1	2.81415653	-2.08534360	-1.98744142
1	0.18755645	-2.04624343	1.92845869	1	2.43285656	-0.50254351	-2.67534113
1	-2.20094347	-1.43534350	2.99585891	1	1.14235640	-1.67314351	-2.36434126
1	-0.22474355	-2.93514347	-0.58614123	1	5.24305630	-0.58924347	0.51685876
1	-2.87714338	-2.89304352	-1.05494130	6	0.29925644	2.29865646	1.09945869
1	-4.09884357	-1.97644341	1.16555870	1	-0.34714356	2.51745653	1.95725870
1	-4.48504353	-0.47274351	-1.34764135	1	-0.09634352	2.87745643	0.25825873
1	-2.51404357	0.45885652	-2.92594123	6	1.71395659	2.79435658	1.41595864
1	-1.31044352	2.49295664	-1.63964128	1	2.14035654	2.25175643	2.26455879
1	-2.53544354	2.83015656	0.72905874	1	1.70235658	3.85755658	1.66855872
1	-4.50834370	0.99635649	0.91175878	1	2.38105655	2.66025662	0.55975878
6	0.78415650	0.35155654	-0.55004126	1	1.03465652	1.21085656	-1.18654132

TS-56pp

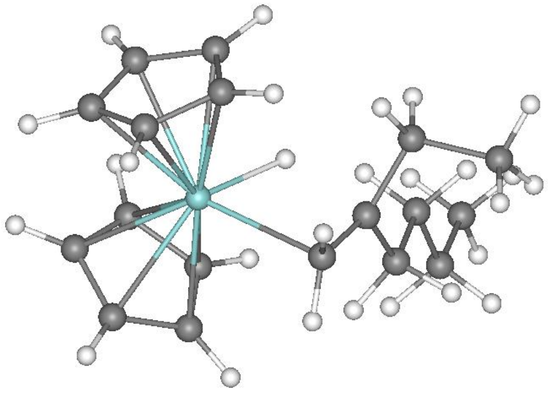

Zero-point vibrational energy	1057832.4 (Joules/Mol)
	252.82802 (Kcal/Mol)
Zero-point correction=	0.402907 (Hartree/Particle)
Thermal correction to Energy=	0.423376
Thermal correction to Enthalpy=	0.424320
Thermal correction to Gibbs Free Energy=	0.353850
Sum of electronic and zero-point Energies=	-4242.065863
Sum of electronic and thermal Energies=	-4242.045395
Sum of electronic and thermal Enthalpies=	-4242.044450
Sum of electronic and thermal Free Energies=	-4242.114920

cartesian

6	2.71340656	2.05842185	-0.35810435	1	0.97960663	1.95292163	1.94269562
6	2.28870678	1.45702171	-1.57280433	1	0.30930659	0.36012179	2.60949564
6	3.13440657	0.34232178	-1.82000434	1	0.23930660	0.12382177	-0.63380438
6	4.09050655	0.27102178	-0.77070439	1	-1.13989341	2.01392174	-1.05110431
6	3.82530642	1.32562172	0.13409564	6	-1.88399351	3.16652179	0.62649566
1	4.38730669	1.54832172	1.03429568	1	0.11210662	2.85622168	-0.13770436
1	2.30630660	2.95472169	0.09099565	6	-2.50349331	-0.24217823	-0.37990433
1	1.48810661	1.80232179	-2.21390414	1	-1.40239346	-0.84567827	1.39009559
1	3.08500671	-0.31317824	-2.68080425	1	3.82790661	-2.70157814	-0.21440436
6	0.96130657	-2.23797822	1.44009566	1	4.89440680	-0.44857824	-0.68530434
6	0.51580662	-2.36057830	0.09899565	1	-2.42519331	0.54622173	1.62109566
6	1.65930653	-2.53897834	-0.72510439	6	-3.58129311	-1.30377829	-0.15080436
6	2.80600643	-2.55457830	0.11059565	1	-1.80579340	-0.58277822	-1.15660441
6	2.37990665	-2.35397816	1.44499564	1	-2.97819328	0.66612172	-0.76960438
1	3.01940680	-2.33187819	2.32019567	6	-4.36619329	-1.61367822	-1.42400432
1	0.33450660	-2.13117814	2.31689572	1	-4.26619339	-0.96037823	0.63249564
1	-0.51169336	-2.34817815	-0.24290435	1	-3.11579323	-2.22167826	0.22969565
1	1.64940667	-2.67757821	-1.79930437	1	-5.13019323	-2.37247825	-1.24420440
40	1.84180665	-0.23027822	0.22549565	1	-3.70489311	-1.98417830	-2.21270418
6	-1.73679340	0.07642176	0.90369564	1	-4.86679316	-0.71777827	-1.80180442
6	0.41810662	1.03692174	1.76209569	1	-1.99759328	4.09722185	0.06799565
6	-0.57649338	1.04362178	0.78599566	1	-1.60179341	3.41582179	1.65259564
6	-0.81549335	2.28692174	-0.04420435	1	-2.85709333	2.67052174	0.64869565

TS-56sp

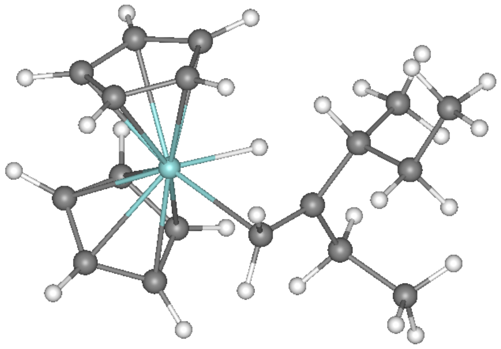

Zero-point vibrational energy 1059823.1 (Joules/Mol)

253.30381 (Kcal/Mol)

Zero-point correction= 0.403665 (Hartree/Particle)

Thermal correction to Energy= 0.424034

Thermal correction to Enthalpy= 0.424978

Thermal correction to Gibbs Free Energy= 0.355164

Sum of electronic and zero-point Energies= -4242.062775

Sum of electronic and thermal Energies= -4242.042407

Sum of electronic and thermal Enthalpies= -4242.041462

Sum of electronic and thermal Free Energies= -4242.111276

cartesian

1	-0.38274568	0.20253044	-0.94143265	6	0.23645431	0.41673043	1.49586749
6	-2.44944572	-2.16086936	-1.09883261	6	1.07245433	2.03063059	-0.23113263
6	-1.06764567	-2.33286953	-0.81823260	6	1.85865438	-0.46956956	-0.22583261
6	-0.93124568	-2.51956940	0.58356738	1	0.45685434	-0.47736958	2.07796741
6	-2.22704554	-2.47586942	1.16256750	1	-0.06084567	1.29573047	2.06796741
6	-3.16444588	-2.26336956	0.12426740	1	1.06245434	2.04673052	-1.32383251
40	-1.76854563	-0.17406955	0.24446741	1	0.22475433	2.62743044	0.11156739
6	-2.27514553	2.03603053	-0.83513260	1	1.35275424	-1.43236959	-0.10283261
6	-3.30634546	1.14203048	-1.22813261	6	2.22635412	-0.30906957	-1.70123255
6	-4.06624556	0.81173044	-0.07593260	6	3.10945415	-0.54466957	0.68496740
6	-3.49074554	1.47443044	1.03386736	6	3.99665403	-1.74636960	0.35456738
6	-2.38444567	2.23633051	0.56566739	1	3.69715405	0.37263045	0.60036737
1	-0.00994569	-2.71536946	1.11776745	1	2.78365421	-0.61106956	1.72816741
1	-2.46034575	-2.60716939	2.21336722	1	3.42015409	-2.67726946	0.35696739
1	-0.27024567	-2.35006952	-1.55073261	1	4.47355413	-1.64516962	-0.62233263
1	-2.88444567	-2.01666951	-2.08023262	6	2.36475420	2.68863058	0.28136739
1	-4.23884583	-2.21026945	0.24316740	1	2.32995415	3.75873065	0.06706739
1	-4.94124556	0.17523044	-0.05163261	1	3.25125456	2.28573060	-0.21003261
1	-3.50054550	0.80543041	-2.23923278	1	2.47805452	2.56003046	1.36106741
1	-1.55624568	2.49863052	-1.49863255	1	2.75715446	-1.19306958	-2.06013274
1	-1.77454567	2.89503050	1.17086744	1	2.88085413	0.55383044	-1.86183250
1	-3.85164547	1.43423045	2.05556750	1	1.33435428	-0.18176955	-2.32373261
6	0.90425432	0.61073041	0.28076738	1	4.79095411	-1.84526956	1.09726739

TS-55

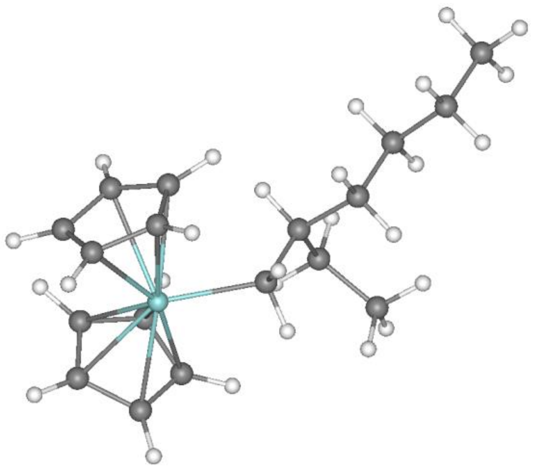

Zero-point vibrational energy	1069126.3 (Joules/Mol)
	255.52732 (Kcal/Mol)
Zero-point correction=	0.407209 (Hartree/Particle)
Thermal correction to Energy=	0.427500
Thermal correction to Enthalpy=	0.428444
Thermal correction to Gibbs Free Energy=	0.357115
Sum of electronic and zero-point Energies=	-4242.055366
Sum of electronic and thermal Energies=	-4242.035075
Sum of electronic and thermal Enthalpies=	-4242.034131
Sum of electronic and thermal Free Energies=	-4242.105460

cartesian

6	-2.26693487	-2.05887175	-1.24224341	6	-0.42563474	0.20872831	0.96615648
6	-1.33283472	-2.37737179	-0.21904346	6	1.01336527	1.78082836	-0.44704345
6	-2.06243467	-2.69567180	0.94965649	6	2.02956533	-0.31327170	0.61745650
6	-3.45203471	-2.61007166	0.64375651	1	-0.23913473	-0.61537170	1.67915654
6	-3.57833481	-2.22737169	-0.70924348	1	-0.50563478	1.12482834	1.57265651
40	-2.53643465	-0.27297169	0.44725654	1	1.71226525	1.77662838	-1.28934348
1	0.55036521	-0.24207167	-0.91894346	1	0.07846528	2.19272828	-0.84434348
6	-2.60283470	1.62182832	-1.20364344	1	1.80286527	-1.36137164	0.85985649
6	-3.83713484	0.92452836	-1.31684339	6	3.26826549	-0.27697170	-0.27784348
6	-4.54463482	1.08242834	-0.10114347	6	1.55416536	2.71032834	0.64085650
6	-3.74763465	1.88702822	0.76385653	1	1.63736534	3.73192835	0.26295653
6	-2.55793476	2.23272824	0.07335653	1	2.54926515	2.40292835	0.97305650
1	-1.63843477	-2.98367167	1.90525651	1	0.90976524	2.73892832	1.52585661
1	-4.26963472	-2.82257175	1.32415652	1	3.57806516	0.76012832	-0.45484349
1	-0.25433475	-2.36567163	-0.31364346	1	3.02246523	-0.69747168	-1.26294339
1	-2.02533484	-1.79247165	-2.26464367	6	4.44666481	-1.04627168	0.32185653
1	-4.50953484	-2.08837175	-1.24404347	1	4.68106556	-0.63057166	1.30855656
1	-5.52763462	0.68252832	0.12055653	1	4.15286541	-2.08937168	0.48735654
1	-4.17943478	0.38272831	-2.18964362	6	5.68856525	-0.99627167	-0.56654352
1	-1.84423470	1.69702828	-1.97444332	1	6.52006531	-1.54527164	-0.11884347
1	-1.75723481	2.85382843	0.45425653	1	6.01596546	0.03542832	-0.72414351
1	-4.02433491	2.21752834	1.75945652	1	5.48696518	-1.43567169	-1.54794347
6	0.77496529	0.32572833	-0.00304347	1	2.24636531	0.17372832	1.57705653

I-6pp

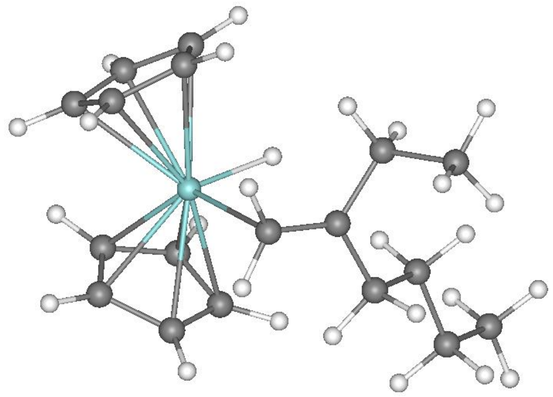

Zero-point vibrational energy 1061614.2 (Joules/Mol)

253.73188 (Kcal/Mol)

Zero-point correction= 0.404347 (Hartree/Particle)

Thermal correction to Energy= 0.425518
Thermal correction to Enthalpy= 0.426462
Thermal correction to Gibbs Free Energy= 0.352776
Sum of electronic and zero-point Energies= -4242.068807
Sum of electronic and thermal Energies= -4242.047636
Sum of electronic and thermal Enthalpies= -4242.046692
Sum of electronic and thermal Free Energies= -4242.120378

cartesian

6	-2.43449354	-2.22607183	-1.36543691	6	1.87830663	0.23922828	-0.98443699
6	-2.64599347	-2.53537178	0.00566303	1	-0.32979345	0.54842830	-2.51753712
6	-1.38689351	-2.58017182	0.64986300	1	-0.95649344	2.11442828	-1.72883689
6	-0.38769346	-2.32197165	-0.32663697	1	0.09460652	3.00462818	0.18336302
6	-1.03099346	-2.11447167	-1.56873691	1	1.53190660	-0.59777170	-1.60183692
1	-0.54319346	-1.92667174	-2.51813698	1	2.66700649	0.72862834	-1.57463694
1	-3.20009351	-2.14927173	-2.12813711	1	-1.20719337	-2.78817177	1.69686306
1	-3.60539341	-2.71327186	0.47456303	1	-3.79079342	-0.56047171	2.28606296
1	0.67950660	-2.30717182	-0.14223698	1	1.13760662	1.94852829	1.14036310
6	-3.68499351	1.38762820	-0.42513695	6	2.20270658	3.23442817	-0.24373695
6	-4.20049334	0.25292829	0.24116305	1	3.12990665	2.65792823	-0.21823695
6	-3.59849334	0.18882829	1.52776313	1	2.30470657	4.06182861	0.46006307
6	-2.72799349	1.30292821	1.66646302	1	2.08290672	3.65112829	-1.24663699
6	-2.77239347	2.03452826	0.45306304	6	2.49310660	-0.26737171	0.32656303
1	-2.24499345	2.95782828	0.24996305	1	2.99670672	0.55622828	0.84546304
1	-3.95589328	1.71502829	-1.42273688	1	1.70320654	-0.61647171	1.00356305
1	-4.93089342	-0.43947172	-0.15833697	6	3.50670671	-1.38697171	0.08176304
1	-2.15459347	1.55722821	2.54706287	1	3.02120662	-2.21657181	-0.44793695
40	-1.76559353	-0.20327172	-0.09513697	1	4.29420662	-1.02077174	-0.58633697
1	-0.60669345	-0.07097171	1.30896306	6	4.12430668	-1.89807177	1.38186312
6	-0.29929346	1.24772823	-1.67993689	1	4.64600658	-1.09437168	1.90866303
6	0.77960652	1.25962830	-0.84633696	1	4.84480667	-2.69577169	1.19156313
6	0.98650652	2.37382817	0.14306304	1	3.35580683	-2.29257178	2.05336285

I-6sp

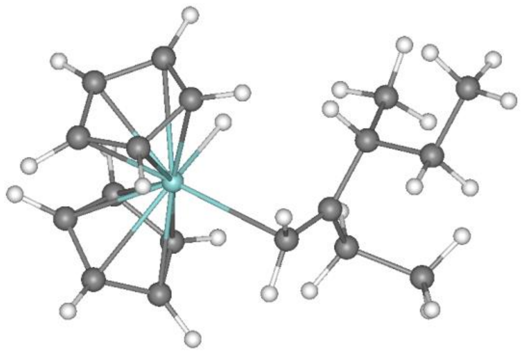

Zero-point vibrational energy	1060063.8 (Joules/Mol)
	253.36133 (Kcal/Mol)
Zero-point correction=	0.403757 (Hartree/Particle)
Thermal correction to Energy=	0.425009
Thermal correction to Enthalpy=	0.425954
Thermal correction to Gibbs Free Energy=	0.353027
Sum of electronic and zero-point Energies=	-4242.067694
Sum of electronic and thermal Energies=	-4242.046441
Sum of electronic and thermal Enthalpies=	-4242.045497
Sum of electronic and thermal Free Energies=	-4242.118423

cartesian

1	-0.84984571	-0.06730002	-1.47304130	6	0.34155434	0.22889999	1.51695871
6	-3.07784581	-2.05730009	-0.79414129	6	1.27545428	2.01299977	0.05205870
6	-1.71184564	-2.42020011	-0.93614131	6	1.95055413	-0.49270004	-0.25024128
6	-1.16774571	-2.55510020	0.36685872	1	0.40425426	-0.77509999	1.93645871
6	-2.19814563	-2.29700017	1.31005871	1	-0.07434571	1.00970006	2.15715861
6	-3.37914586	-1.99419999	0.59475869	1	1.13875437	2.11099982	-1.02954125
40	-1.79744577	-0.12880002	0.08655870	1	0.50135428	2.60689998	0.54585868
6	-2.08444571	2.25519991	-0.70904130	1	1.35945415	-1.41690004	-0.23404132
6	-3.10784578	1.48920000	-1.32804132	6	2.26745415	-0.16290002	-1.71094131
6	-3.98144579	1.02740002	-0.31494129	6	3.22465420	-0.78119999	0.58585870
6	-3.48784566	1.47920001	0.93755871	6	4.04165411	-1.94700003	0.02815870
6	-2.32134581	2.25559998	0.68515867	1	3.85125446	0.11449998	0.63075870
1	-0.15404570	-2.85370016	0.60395867	1	2.92415428	-1.00480008	1.61485875
1	-2.10214567	-2.33950019	2.38935852	1	3.42095423	-2.83940005	-0.10154131
1	-1.19094563	-2.58850002	-1.86874127	1	4.49275446	-1.70410001	-0.93594134
1	-3.77674580	-1.89339995	-1.60534132	6	2.65175414	2.58539987	0.44325870
1	-4.34464550	-1.76880002	1.03055871	1	2.65605426	3.66169977	0.26195872
1	-4.87344599	0.43329999	-0.46834126	1	3.45925426	2.14689994	-0.14414130
1	-3.20374584	1.30739999	-2.39104128	1	2.86025429	2.41529989	1.50235879
1	-1.27734566	2.75499988	-1.22854125	1	2.71445417	-1.02970004	-2.20104146
1	-1.73964572	2.77819991	1.43525875	1	2.97725415	0.66549999	-1.79594123
1	-3.95094585	1.31389999	1.90305877	1	1.36085415	0.09599998	-2.26474142
6	1.12655425	0.56919998	0.45235872	1	4.85225439	-2.20080018	0.71425867

I-7ppα

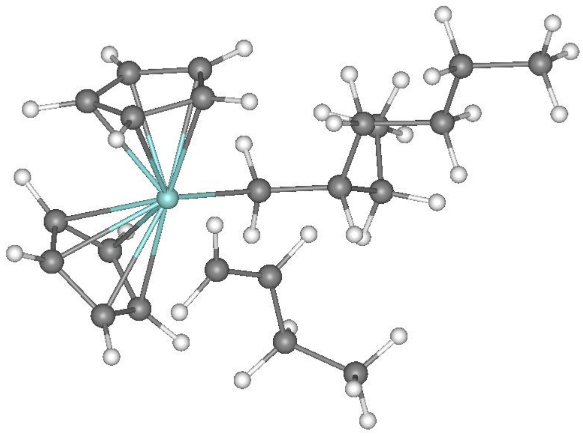

Zero-point vibrational energy 1366326.3 (Joules/Mol)

326.55983 (Kcal/Mol)

Zero-point correction=	0.520406 (Hartree/Particle)
Thermal correction to Energy=	0.547631
Thermal correction to Enthalpy=	0.548575
Thermal correction to Gibbs Free Energy=	0.462917
Sum of electronic and zero-point Energies=	-4399.124315
Sum of electronic and thermal Energies=	-4399.097091
Sum of electronic and thermal Enthalpies=	-4399.096147
Sum of electronic and thermal Free Energies=	-4399.181805

cartesian

6	-0.88686043	-2.15953255	1.55422068	6	2.15003967	-0.40403265	-3.30817938
6	-0.76276040	-2.61913252	0.22542071	1	1.26043963	1.51396739	-2.88487935
6	-2.06486034	-2.92103267	-0.25467929	6	-1.60036039	0.98956740	2.47672057
6	-2.98586035	-2.70193267	0.80712074	6	-0.67346042	1.45906734	1.61552072
6	-2.26626039	-2.21523261	1.91542065	6	-0.75516045	2.76496744	0.88022071
1	-2.69096041	-1.96003258	2.87942076	1	-1.39236045	0.13176736	3.10872078
1	-0.06976044	-1.86383259	2.20252061	1	-2.51706028	1.54066741	2.67642069
1	0.15583956	-2.71893263	-0.33357930	1	0.26983958	0.91106737	1.52312064
1	-2.30566025	-3.30953264	-1.23737931	1	-0.59856045	2.60166740	-0.19157931
1	-4.05476046	-2.86923265	0.76852071	1	-1.74396038	3.21376729	1.01612067
6	-3.18776035	1.46536744	-1.17477930	6	0.33933961	3.71366739	1.39942062
6	-3.38506031	0.28746736	-1.93347931	1	1.91653955	-1.63183260	-0.83347929
6	-4.20766020	-0.58783263	-1.17967939	6	3.31963968	-0.28853261	0.09222070
6	-4.52026033	0.05186737	0.05022070	1	1.35203946	-0.91433263	0.66422075
6	-3.89396048	1.32286739	0.04612070	1	2.61363935	-0.03883263	-4.22747898
1	-3.97706032	2.07106733	0.82462072	1	1.23913956	-0.93893266	-3.59447932
1	-2.61456037	2.32936740	-1.48487937	1	2.83953953	-1.12743258	-2.86287928
1	-2.97346020	0.08736739	-2.91537929	6	3.96943951	-1.25953257	1.07892072
1	-4.56106043	-1.55933261	-1.50137937	1	3.32093954	0.72356737	0.52212071
1	-5.14966011	-0.34663263	0.83662075	1	3.93443966	-0.23683262	-0.81397927
40	-2.06796026	-0.45483261	0.09542070	6	5.41253948	-0.88303268	1.41042066
1	1.11193955	1.28226745	-0.44917929	1	3.93913984	-2.27233267	0.66022074
6	-0.35906044	0.00246738	-1.37567937	1	3.37613964	-1.28853261	2.00162077
6	1.11503959	0.36836734	-1.06147933	1	5.85383987	-1.58443260	2.12192059
6	1.85683954	0.75896740	-2.35997939	1	5.46563959	0.11766738	1.84902072
6	1.88793957	-0.69053268	-0.26837930	1	6.03293991	-0.88473260	0.50992072
1	-0.36896041	-0.89243257	-2.02507925	1	0.29933959	4.66086721	0.85932070
1	-0.73506045	0.80656743	-2.02097940	1	1.33223963	3.28046751	1.25172067
1	2.79873943	1.25136745	-2.09497929	1	0.20703959	3.91526723	2.46432066

I-7ppβ

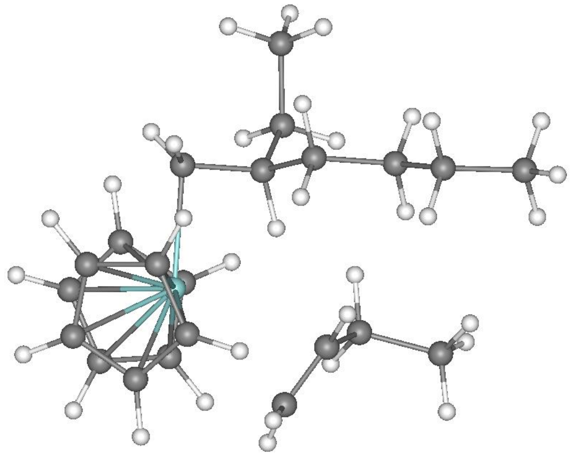

Zero-point vibrational energy	1369251.0 (Joules/Mol)
	327.25885 (Kcal/Mol)
Zero-point correction=	0.521520 (Hartree/Particle)
Thermal correction to Energy=	0.548064
Thermal correction to Enthalpy=	0.549008
Thermal correction to Gibbs Free Energy=	0.466066
Sum of electronic and zero-point Energies=	-4399.132701
Sum of electronic and thermal Energies=	-4399.106157
Sum of electronic and thermal Enthalpies=	-4399.105213
Sum of electronic and thermal Free Energies=	-4399.188156

cartesian

6	1.83804107	0.44294834	-2.72208619	6	-1.60145879	-3.12655163	1.73891377
6	1.79394114	-0.95955163	-2.63588619	1	-0.16575891	-1.70115161	2.48201394
6	3.01764107	-1.41035163	-2.06988621	6	0.95854115	2.68174815	-0.61968625
6	3.83144116	-0.27415165	-1.84488618	6	-0.15755886	2.09634829	-0.14138621
6	3.09674120	0.88044840	-2.21048617	1	1.17244112	2.70964837	-1.68098629
1	3.46124101	1.89984834	-2.17478609	1	-0.82335889	1.59904838	-0.85348624
1	1.05584109	1.06824839	-3.13618612	1	-1.73615885	-2.32715178	-1.00288618
1	0.96864116	-1.58715165	-2.94608617	6	-2.76145887	-0.52465165	-0.39778620
1	3.28884101	-2.44105172	-1.87858617	1	-1.03495884	-0.85935163	-1.64798617
1	4.84174109	-0.28495166	-1.45648623	1	-1.89605880	-3.42075181	2.74831390
6	1.95274115	0.25854835	2.30371380	1	-0.89265883	-3.87025166	1.36451375
6	2.65044117	-0.94745159	2.04571390	1	-2.49535894	-3.16865182	1.11121380
6	3.80744123	-0.63245159	1.29371381	6	-3.63495874	-0.25935164	-1.62528622
6	3.85314107	0.78324836	1.12931383	1	-2.51355886	0.43474835	0.08041380
6	2.71554112	1.32684839	1.75701380	1	-3.34355879	-1.08295166	0.34511381
1	2.47964096	2.37944818	1.83721375	6	-4.93905878	0.45224836	-1.27178621
1	1.03494108	0.35954836	2.87141395	1	-3.85435867	-1.21055162	-2.12328601
1	2.36514115	-1.93405163	2.38111401	1	-3.06775880	0.34164834	-2.34758615
1	4.55244112	-1.34015167	0.95201373	1	-5.53665876	0.65384841	-2.16318607
1	4.63214111	1.34424841	0.62831378	1	-4.74355888	1.40934837	-0.77778625
40	1.91674113	-0.07485165	-0.22248620	1	-5.54435873	-0.15485165	-0.59318626
1	-0.18685889	-0.20655166	0.53251380	1	1.59394109	3.28944826	0.01931379
6	0.83314109	-2.06965184	0.00131379	6	-0.69205892	2.29824829	1.25161374
6	-0.42565882	-1.31225157	0.37691379	1	0.09194112	2.70194817	1.90061378
6	-0.98405886	-1.72725165	1.75301373	1	-1.02855885	1.35114837	1.68721378
6	-1.48375893	-1.29255164	-0.74168622	6	-1.88135886	3.27184820	1.19861376
1	0.72724110	-2.62035179	-0.93218625	1	-1.56715894	4.24574852	0.81711376
1	1.16554117	-2.75705171	0.77681375	1	-2.30425882	3.40844822	2.19531393
1	-1.73035884	-1.00025165	2.09261394	1	-2.67055893	2.88944817	0.54501379

TS-78pp

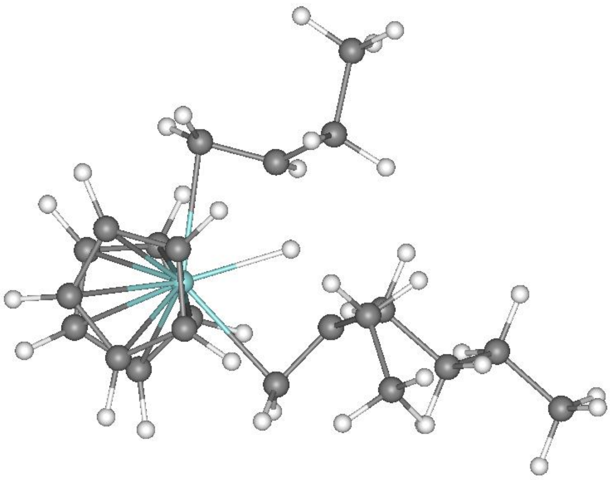

Zero-point vibrational energy	1357963.2 (Joules/Mol)
	324.56098 (Kcal/Mol)
Zero-point correction=	0.517221 (Hartree/Particle)
Thermal correction to Energy=	0.543246
Thermal correction to Enthalpy=	0.544190
Thermal correction to Gibbs Free Energy=	0.462535
Sum of electronic and zero-point Energies=	-4399.109685
Sum of electronic and thermal Energies=	-4399.083659
Sum of electronic and thermal Enthalpies=	-4399.082715
Sum of electronic and thermal Free Energies=	-4399.164370

cartesian

6	0.14888614	-1.29520178	-1.21351731	6	0.80708617	-0.14750175	-0.71981722
6	-0.98201382	-2.42150187	1.51108265	6	1.86998606	-0.35320175	0.35668272
6	-1.78741384	-3.00070190	0.48988274	1	-0.13251385	-1.34960175	-2.25881720
6	-3.11691380	-2.57720184	0.70018274	1	0.41858619	-2.24830174	-0.76951724
6	-3.13631392	-1.70180178	1.81768274	6	1.07018614	1.03929830	-1.66351736
6	-1.81381392	-1.63490176	2.33168292	1	2.02988601	0.57529825	0.91828275
1	-1.49651384	-1.08280182	3.20818281	1	1.53478599	-1.11040175	1.07278275
1	0.07558614	-2.59550190	1.66088271	1	-4.84831381	-0.99250174	-0.92991722
1	-1.45401382	-3.68160176	-0.28271726	1	-3.97311401	-2.88160181	0.11348274
1	-4.01311398	-1.22290170	2.23638272	6	0.00268614	3.75079823	2.15518284
6	-2.16101384	0.36469823	-2.36541724	1	-1.00611389	3.98919797	2.49948287
6	-3.01361394	-0.75190175	-2.18951726	1	0.48368615	4.67469835	1.82828271
6	-4.01301384	-0.38330173	-1.24751735	1	0.56748611	3.35829806	3.00488281
6	-3.74831390	0.93109822	-0.81161726	6	3.21028614	-0.79700172	-0.24751727
6	-2.59051394	1.39449823	-1.49981725	1	3.05998611	-1.70530176	-0.84401727
1	-2.15381384	2.38259816	-1.41551733	6	4.26078653	-1.05540180	0.83448279
1	-1.32171392	0.41929823	-3.04621720	1	3.58508611	-0.02650176	-0.93161726
1	-2.94411397	-1.69900179	-2.71051717	1	1.81198621	1.68869829	-1.18941724
1	-4.35131359	1.49449825	-0.11251727	1	0.17338616	1.65139830	-1.79361725
40	-1.82891393	-0.50060177	0.02248273	6	1.59568620	0.61259824	-3.04011726
6	-0.03431386	2.73599815	1.00488269	1	1.89768600	1.49449825	-3.60911727
1	-0.61631382	3.13929820	0.17018273	1	0.83788615	0.08629824	-3.62541723
6	-2.05301380	1.36509824	1.66838264	1	2.45958614	-0.04970175	-2.95081711
6	-0.66691387	1.44569826	1.49658275	6	5.59328651	-1.51450169	0.24468273
1	-0.04851383	0.86829829	2.18928289	1	3.88478589	-1.81270170	1.53198266
1	-2.47021389	0.85609829	2.52818274	1	4.41048622	-0.14010176	1.41818273
1	-2.65301394	2.17579818	1.26558268	1	6.33428621	-1.68470168	1.02838266
1	-0.14761385	0.57529825	0.27058274	1	5.99768639	-0.76480174	-0.44101727
1	0.98058611	2.56159806	0.64238274	1	5.47408628	-2.44820189	-0.31211728

TS-79ppp

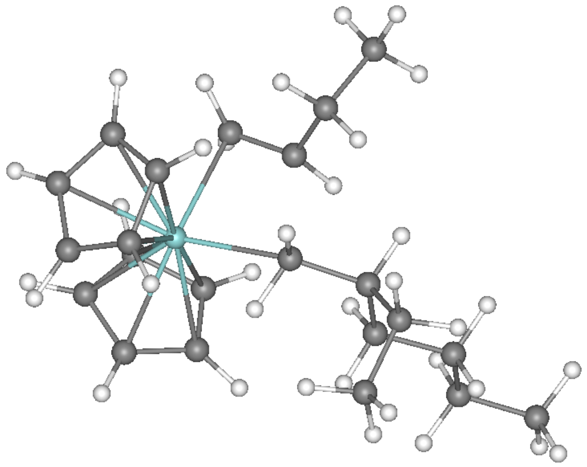

Zero-point vibrational energy	1369151.9 (Joules/Mol)
	327.23517 (Kcal/Mol)
Zero-point correction=	0.521482 (Hartree/Particle)
Thermal correction to Energy=	0.547170
Thermal correction to Enthalpy=	0.548114
Thermal correction to Gibbs Free Energy=	0.465838
Sum of electronic and zero-point Energies=	-4399.116317
Sum of electronic and thermal Energies=	-4399.090630
Sum of electronic and thermal Enthalpies=	-4399.089686
Sum of electronic and thermal Free Energies=	-4399.171962

cartesian

6	-0.77587074	-2.20993447	1.51526892	6	1.71932924	0.14966545	-3.21183109
6	-0.59727079	-2.63623452	0.18186897	1	1.27302921	2.10236549	-2.40933108
6	-1.86227071	-3.02583456	-0.33373103	6	-1.76697075	0.76986545	2.03346896
6	-2.81587076	-2.88233447	0.70946896	6	-0.67927074	1.33716536	1.34116888
6	-2.15147066	-2.36583447	1.84506893	6	-0.73677075	2.79386544	0.94016898
1	-2.60737085	-2.15743446	2.80516887	1	-1.54577076	0.06206545	2.82516885
1	0.00182927	-1.85823464	2.18226886	1	-2.62987089	1.40556550	2.21036887
1	0.33932924	-2.67193460	-0.35683101	1	0.30962926	0.94856542	1.57706892
1	-2.05067062	-3.42433453	-1.32323110	1	-0.11097074	3.00906539	0.07096897
1	-3.86787081	-3.13003445	0.64696896	1	-1.76807082	3.05876541	0.68646896
6	-3.31697083	1.32556534	-1.12743104	6	-0.25947076	3.64886546	2.12406898
6	-3.27077055	0.22566545	-2.02303100	1	1.85872924	-1.32733464	-0.86183101
6	-4.04667091	-0.82203454	-1.47233105	6	3.53272915	-0.28973454	-0.00763103
6	-4.57417059	-0.36993456	-0.23153102	1	1.58842933	-0.76633453	0.78146899
6	-4.13397074	0.96096545	-0.02913103	1	2.07782936	0.55616546	-4.15993071
1	-4.40677071	1.59536552	0.80346900	1	0.68662930	-0.17943455	-3.37223101
1	-2.84817076	2.28906560	-1.28163111	1	2.32482910	-0.73343456	-2.98893118
1	-2.74097061	0.19396544	-2.96843100	6	4.18952942	-1.47553444	0.70056897
1	-4.22357082	-1.78913450	-1.92613113	1	3.72532940	0.62916547	0.56186897
1	-5.23087072	-0.92693454	0.42496899	1	4.00832939	-0.15343454	-0.98593104
40	-2.08267069	-0.56403458	0.06776897	6	5.70152950	-1.30663466	0.84036899
1	1.51122916	1.58876538	-0.04623104	1	3.97112942	-2.39413452	0.14306897
6	-0.22587076	0.57916546	-0.76193100	1	3.73912954	-1.59993458	1.69266891
6	1.29502928	0.75326544	-0.72683102	1	6.15302944	-2.16223454	1.34716892
6	1.82102931	1.20196533	-2.10793114	1	5.94352913	-0.40943456	1.41726887
6	2.02562904	-0.47943455	-0.18573102	1	6.17562914	-1.20983458	-0.14033103
1	-0.43127078	-0.36803454	-1.35383105	1	-0.30107075	4.70766544	1.86226892
1	-0.66787076	1.39436555	-1.34003103	1	0.77352929	3.40666556	2.38766885
1	2.86532927	1.50926542	-1.99713111	1	-0.88377076	3.48626542	3.00546885

TS-79pps

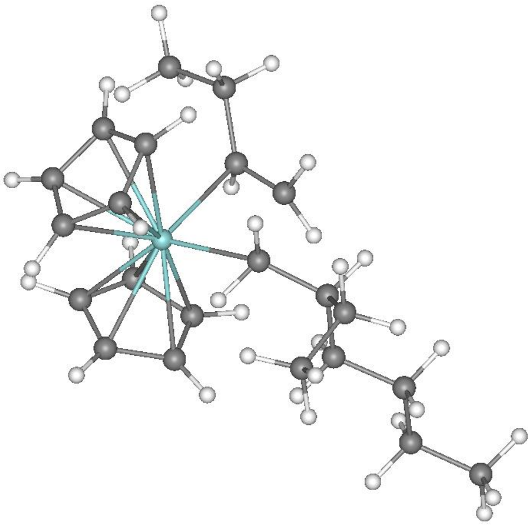

Zero-point vibrational energy	1367894.9 (Joules/Mol)
	326.93473 (Kcal/Mol)
Zero-point correction=	0.521004 (Hartree/Particle)
Thermal correction to Energy=	0.546784
Thermal correction to Enthalpy=	0.547729
Thermal correction to Gibbs Free Energy=	0.464714
Sum of electronic and zero-point Energies=	-4399.108742
Sum of electronic and thermal Energies=	-4399.082961
Sum of electronic and thermal Enthalpies=	-4399.082017
Sum of electronic and thermal Free Energies=	-4399.165031

cartesian

6	-0.51174998	-1.81132412	-1.62193799	6	2.41825008	3.12177587	-0.22803797
6	-0.10194996	-0.66702414	-2.33933806	1	1.69394994	2.85977578	1.78736210
6	-1.23985004	-0.11772415	-2.98803806	6	-1.72705007	-1.50252414	1.32236207
6	-2.34704995	-0.96102417	-2.70363808	6	-0.53394997	-0.83272415	1.59956205
6	-1.90454984	-1.99742413	-1.84903800	1	-0.48124996	-0.19122416	2.47696185
1	-2.51585007	-2.80512404	-1.46443796	1	-1.61395001	-2.42492414	0.75456202
1	0.12805003	-2.45242405	-1.02723801	6	-2.87744999	-1.53152418	2.31846189
1	0.90445000	-0.27572414	-2.39453816	1	0.41045004	-1.25592411	1.27356207
1	-1.24514997	0.74657589	-3.64043808	1	2.40035009	0.49997586	-1.10923803
1	-3.35115004	-0.84092414	-3.08853817	6	3.85565019	-0.24812415	0.28656203
6	-2.98624992	1.78307593	0.94576204	1	1.88975000	-0.93642414	-0.23633796
6	-2.54555011	2.53467584	-0.16963796	1	-2.51324987	-2.02652407	3.22636199
6	-3.19845009	2.03837585	-1.32553792	6	-4.07524967	-2.32332420	1.79346204
6	-4.07154989	0.99437588	-0.91523796	1	-3.15895009	-0.52152413	2.62846184
6	-3.94254994	0.83977586	0.48386204	1	-4.91484976	-2.28502417	2.49076200
1	-4.52145004	0.16337585	1.09616208	1	-3.80474997	-3.37482405	1.66156209
1	-2.67954993	1.92747593	1.97496212	1	-4.41925001	-1.95392418	0.82166207
1	-1.82045007	3.34017587	-0.15223797	1	2.83695006	4.10547590	-0.00473797
1	-3.08354998	2.41707587	-2.33353806	1	1.45125008	3.28657579	-0.71633798
1	-4.74814987	0.43787584	-1.55093801	1	3.08454990	2.64427590	-0.95173794
40	-1.71455002	0.16097584	-0.54463798	6	4.53005028	-1.27632415	-0.62263799
1	1.68705010	0.42697585	1.85726202	1	3.88505006	-0.60342413	1.32516205
6	0.15085006	1.04737580	0.52856207	1	4.43405008	0.68277586	0.26126206
6	1.63444996	0.90627587	0.86946201	6	5.98395014	-1.53692412	-0.23253797
6	2.28154993	2.29817581	1.05236208	1	4.48385000	-0.92542410	-1.66043794
6	2.40625000	0.02667585	-0.11873797	1	3.96574998	-2.21612406	-0.58713794
1	0.07285005	1.43107593	-0.53833795	1	6.44685030	-2.27242422	-0.89403796
1	-0.28344995	1.81987584	1.16806209	1	6.05225039	-1.91652417	0.79096204
1	3.26985002	2.16167593	1.50126207	1	6.57445002	-0.61812413	-0.28753796

I-8ppp

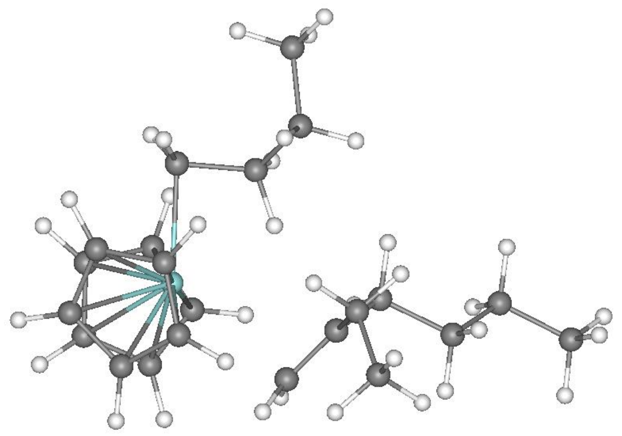

Zero-point vibrational energy	1366876.7 (Joules/Mol)
	326.69138 (Kcal/Mol)
Zero-point correction=	0.520616 (Hartree/Particle)
Thermal correction to Energy=	0.547725
Thermal correction to Enthalpy=	0.548669
Thermal correction to Gibbs Free Energy=	0.462453
Sum of electronic and zero-point Energies=	-4399.138187
Sum of electronic and thermal Energies=	-4399.111078
Sum of electronic and thermal Enthalpies=	-4399.110134
Sum of electronic and thermal Free Energies=	-4399.196349

cartesian

6	-1.19726038	-1.51092935	2.34296560	6	1.11563969	-0.77722937	-0.68483448
6	-1.96926033	-2.47922945	1.63776553	6	2.04603958	-0.38382936	0.43176553
6	-3.31056023	-2.03622937	1.62966561	1	-0.18166035	-2.31652951	-1.41983449
6	-3.36966038	-0.78272939	2.29336548	1	0.39983964	-2.48752952	0.32466552
6	-2.06446028	-0.47632936	2.75486565	6	1.24603963	0.01257063	-1.97413445
1	-1.78076041	0.40357065	3.31746554	1	2.05753946	0.70667064	0.55336553
1	-0.13756034	-1.57152939	2.55926561	1	1.72343957	-0.82352936	1.38156557
1	-1.60996032	-3.41132951	1.21976554	1	-4.96426058	-1.21932936	-0.60563451
1	-4.25726032	-0.18132937	2.44746566	1	-4.14676046	-2.56972933	1.19766557
6	-2.14956045	-0.70692939	-2.32293439	6	-0.28426036	4.32447052	0.69906551
6	-3.05796051	-1.60912943	-1.71853447	1	-1.35526037	4.51477051	0.80526549
6	-4.08706045	-0.83422935	-1.10823441	1	0.15413964	5.15467072	0.14176552
6	-3.79066038	0.53167063	-1.30833447	1	0.15803963	4.32057047	1.69936562
6	-2.57966042	0.61157066	-2.04883456	6	3.47853947	-0.85912937	0.11906552
1	-2.09446049	1.52397060	-2.37623453	1	3.47523975	-1.94892931	0.00076552
1	-1.27396035	-0.97722936	-2.89993453	6	4.46633959	-0.45752937	1.21546555
1	-3.01196051	-2.69072938	-1.75853443	1	3.81113958	-0.43882936	-0.83813453
1	-4.39006042	1.36577058	-0.97013456	1	1.99993956	0.79287064	-1.83303440
40	-2.00236034	-0.41452938	0.20976552	1	0.30853963	0.54087061	-2.18553448
6	-0.02706033	2.99647069	-0.01063448	6	1.63293970	-0.86002934	-3.17553449
1	-0.45996034	3.01727057	-1.01803446	1	1.74333966	-0.24602938	-4.07143450
6	-2.14486027	1.77507067	0.81926548	1	0.88113964	-1.62632942	-3.38073444
6	-0.63176036	1.81637049	0.76606548	1	2.58053970	-1.37182927	-2.99033451
1	-0.18616036	1.74427056	1.76466560	6	5.88223934	-0.95012939	0.92246550
1	-2.55736017	2.01157045	1.79806554	1	4.12533951	-0.86262935	2.17496562
1	-2.58586025	2.43507051	0.07176552	1	4.46673965	0.63337064	1.31916559
1	-0.20456034	0.90567058	0.24016553	1	6.57343960	-0.65432936	1.71436560
1	1.05133963	2.84517050	-0.13273448	1	6.25553942	-0.53712940	-0.01893448
6	0.32153964	-1.87082934	-0.56593448	1	5.90963936	-2.04052925	0.84406549

I-9ppp

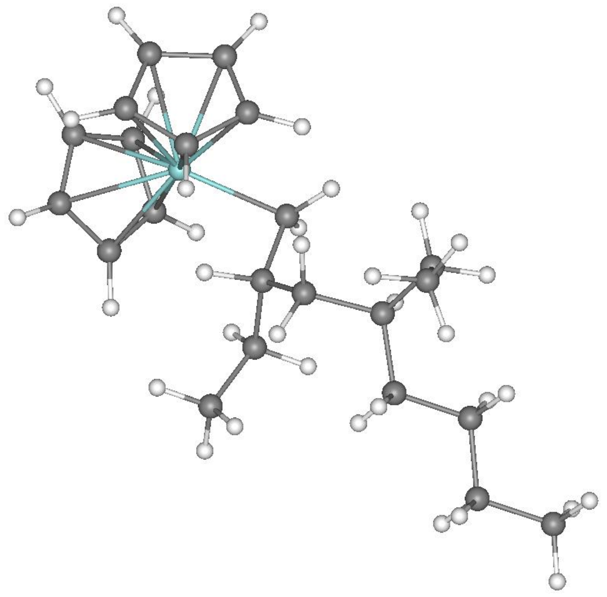

Zero-point vibrational energy	1371387.3 (Joules/Mol)
	327.76944 (Kcal/Mol)
Zero-point correction=	0.522334 (Hartree/Particle)
Thermal correction to Energy=	0.548235
Thermal correction to Enthalpy=	0.549180
Thermal correction to Gibbs Free Energy=	0.464929
Sum of electronic and zero-point Energies=	-4399.143885
Sum of electronic and thermal Energies=	-4399.117984
Sum of electronic and thermal Enthalpies=	-4399.117040
Sum of electronic and thermal Free Energies=	-4399.201290

cartesian

6	-3.50950336	-2.25191903	1.10231900	6	2.50149632	2.93548107	-0.44958106
6	-3.44910336	-2.38861895	-0.30758104	1	1.16249645	2.49868107	1.19021893
6	-4.56980324	-1.71431899	-0.86758101	6	-1.18450344	-0.13461898	1.29341900
6	-5.32660341	-1.16921902	0.19841895	6	-0.55600345	-0.49981901	-0.03758105
6	-4.66440344	-1.48451900	1.41441894	6	-0.05500346	-1.95261896	-0.09798105
1	-5.00180340	-1.21721900	2.40961885	1	-1.12240350	-0.93751895	2.02871895
1	-2.82050347	-2.67801905	1.81921899	1	-0.79750347	0.79448104	1.71381903
1	-2.69870329	-2.93681908	-0.86558104	1	-1.35970342	-0.48041901	-0.89028108
1	-4.82020330	-1.65951896	-1.92068100	1	0.86139655	-2.00371885	0.49991894
1	-6.25310326	-0.61761898	0.10251895	1	-0.77510345	-2.60191894	0.41121894
6	-2.44760346	2.48588109	0.15741895	6	0.20319653	-2.47291899	-1.51038098
6	-2.76600361	2.12258101	-1.17938101	1	3.11519670	0.37238100	-1.31598103
6	-4.15530348	1.82708108	-1.23498106	6	4.21709681	-0.10261898	0.47851896
6	-4.69210339	2.00928092	0.06741895	1	2.64949656	-1.13591897	-0.56068110
6	-3.63490343	2.41148114	0.92501891	1	2.61089659	3.97738099	-0.14018105
1	-3.72240353	2.63558102	1.98221898	1	1.78099644	2.90848112	-1.27468097
1	-1.47780347	2.79398108	0.52521896	1	3.46659660	2.60808110	-0.84558105
1	-2.07630348	2.10238099	-2.01608109	6	5.31859684	-0.93911898	-0.17478105
1	-4.71420336	1.54218102	-2.11848116	1	4.01149654	-0.49171898	1.48501897
1	-5.73020315	1.89438105	0.35261896	1	4.58409643	0.92228103	0.61271894
40	-3.13090324	0.05438101	0.18061896	6	6.61489677	-0.93461895	0.63341892
1	1.53419650	0.07238101	1.27921903	1	5.51099682	-0.55431896	-1.18308103
6	0.47139657	0.53178102	-0.54308105	1	4.96459675	-1.96921897	-0.29898104
6	1.74149644	0.60538101	0.34051895	1	7.38929653	-1.53281891	0.14771895
6	2.05699635	2.06198096	0.72461891	1	6.45389652	-1.34491897	1.63461900
6	2.92669630	-0.09611899	-0.34108105	1	7.00129652	0.08228101	0.74811900
1	0.74059653	0.30218101	-1.58028102	1	0.50729656	-3.52151895	-1.48548102
1	-0.01230347	1.51438105	-0.57068110	1	0.99489653	-1.91421902	-2.01518106
1	2.82949638	2.07388115	1.49971902	1	-0.69710350	-2.40221906	-2.13268113

I-9pps

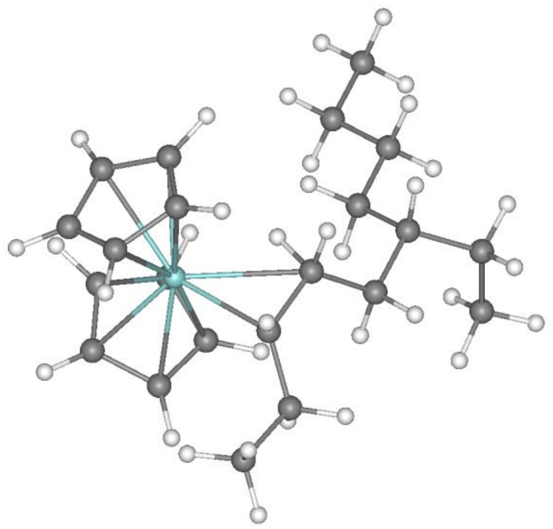

Zero-point vibrational energy	1371841.3 (Joules/Mol)
	327.87794 (Kcal/Mol)
Zero-point correction=	0.522507 (Hartree/Particle)
Thermal correction to Energy=	0.548632
Thermal correction to Enthalpy=	0.549576
Thermal correction to Gibbs Free Energy=	0.464812
Sum of electronic and zero-point Energies=	-4399.142423
Sum of electronic and thermal Energies=	-4399.116297
Sum of electronic and thermal Enthalpies=	-4399.115353
Sum of electronic and thermal Free Energies=	-4399.200117

cartesian

6	-3.12104988	-0.74019659	2.34745169	6	-1.85114992	1.56940353	0.65155172
6	-2.30074978	-1.87689650	2.10945153	6	-0.51894993	1.00540352	1.13125169
6	-2.92624998	-2.66809654	1.11155176	1	-2.48425007	1.81070352	1.50925171
6	-4.13074970	-2.01709652	0.72775173	6	-1.83644986	2.76520348	-0.30004832
6	-4.24965000	-0.82949656	1.49925172	1	2.08015013	-0.09439655	-0.89394826
1	-5.06164980	-0.11289655	1.44555175	6	3.50055027	-1.10869658	0.36915168
1	-2.92565012	0.04680346	3.06465149	1	1.37325013	-1.02589655	0.42065170
1	-1.37484992	-2.11279655	2.62165165	1	-1.29784989	3.59930348	0.16845171
1	-2.56094980	-3.61259651	0.72745174	6	-3.25845003	3.22730350	-0.62154830
1	-4.85034990	-2.38299656	0.00535170	1	-1.29644990	2.54460359	-1.22614825
6	-0.88924992	-0.07669654	-2.09534836	1	-3.25684977	4.08530331	-1.29734826
6	-0.82594991	-1.48219645	-1.86904824	1	-3.78485012	3.52260351	0.29075170
6	-2.12704992	-2.00939655	-2.04794836	1	-3.84674978	2.42960358	-1.09184825
6	-3.00215006	-0.93109655	-2.35674834	1	4.42725039	2.99610353	-0.59954828
6	-2.22434998	0.26100343	-2.40754843	1	2.71595001	2.90260339	-0.99534827
1	-2.59275007	1.25080347	-2.64704847	1	3.76395035	1.49700356	-1.24014831
1	-0.05044991	0.60750341	-2.05154848	6	3.48625040	-2.45669651	-0.35284832
1	0.07005012	-2.04619646	-1.63154829	1	3.67615032	-1.26749647	1.44105172
1	-2.41044998	-3.05089641	-1.95954823	1	4.34615040	-0.51669657	0.00115170
1	-4.05975008	-1.01249647	-2.57884836	6	4.79975033	-3.22249651	-0.20204830
40	-2.25754976	-0.54999655	-0.02024830	1	3.27834988	-2.29109645	-1.41754830
1	2.01165009	0.65590346	2.06815171	1	2.66035032	-3.06649661	0.03625170
6	0.76475012	1.70410347	0.66855174	1	4.76415014	-4.18219662	-0.72264826
6	2.05945015	0.94840342	1.00905168	1	5.01824999	-3.41969657	0.85125172
6	3.27594995	1.87880349	0.85785174	1	5.63455009	-2.64909649	-0.61434829
6	2.19444990	-0.33679652	0.17345171	1	-0.50634992	0.88120347	2.21895170
1	4.16135025	1.37240350	1.25385177	1	-0.35794991	-0.08869655	0.79705173
6	3.55465031	2.33980346	-0.57424825	1	0.77905011	2.68990350	1.14905167
1	3.12144995	2.75540352	1.49695170	1	0.72005010	1.89130354	-0.40874830

S1.3. Zr-Al1 (η^5-C_5H_5)$_2$Zr-based catalytic species

I-5ppα-H

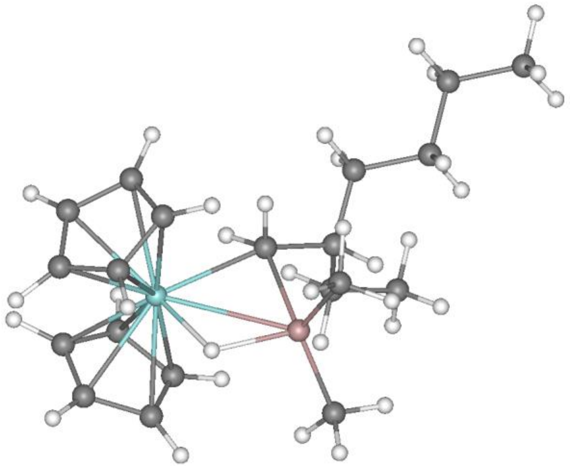

Zero-point vibrational energy	1288585.7 (Joules/Mol)
	307.97937 (Kcal/Mol)
Zero-point correction=	0.490796 (Hartree/Particle)
Thermal correction to Energy=	0.518180
Thermal correction to Enthalpy=	0.519124
Thermal correction to Gibbs Free Energy=	0.432776
Sum of electronic and zero-point Energies=	-4564.806381
Sum of electronic and thermal Energies=	-4564.778997
Sum of electronic and thermal Enthalpies=	-4564.778053
Sum of electronic and thermal Free Energies=	-4564.864401

cartesian

6	4.45959616	1.31899095	0.23541072	1	0.90699631	2.88209105	-1.78188920
6	3.37179613	2.05249119	-0.30858928	1	0.65829635	-1.78280902	-3.42958927
6	2.39509630	2.20219111	0.70631069	1	-0.67620373	-1.95780897	-2.29128933
6	2.88979626	1.58079100	1.88811076	1	-0.83950365	-0.91210896	-3.69558930
6	4.16749620	1.04419112	1.59961081	1	0.50279629	-0.07010894	1.20801079
40	2.53909636	-0.25800896	0.22561073	1	-0.03780371	-1.33390903	0.09291072
6	0.13229632	-0.25460893	0.16191073	6	-1.27340376	0.41959107	0.15181072
1	2.32219648	0.03039106	-1.65118921	6	-2.29250383	-0.42710894	0.95461071
6	2.30589628	-2.65840888	-0.50638932	6	-1.19440365	1.87019110	0.64781070
6	3.59729624	-2.22910881	-0.92298931	1	-1.65280378	0.44499105	-0.88638932
6	4.36429644	-1.96390891	0.23651072	1	-0.76360369	1.86429095	1.65981078
6	3.54829645	-2.21790886	1.37441075	1	-0.50660372	2.42959118	0.00701072
6	2.28329635	-2.66550899	0.90731072	6	-3.66910362	-0.53320897	0.29161072
1	1.45669627	2.73189116	0.61471069	1	-1.90740359	-1.44420886	1.09141076
1	2.38769627	1.54819107	2.84891057	1	-2.39090347	-0.00410894	1.96341074
1	3.30239630	2.43129110	-1.32038927	6	-4.66700363	-1.32360888	1.13791072
1	5.37099648	1.05679107	-0.28798929	1	-3.55440378	-1.02200890	-0.68518931
1	4.81229639	0.52369106	2.29691052	1	-4.07530355	0.46379107	0.08871073
1	5.39659643	-1.63780904	0.25651070	1	-4.78000355	-0.83580893	2.11291075
1	3.93419647	-2.13830876	-1.94788921	1	-4.26090336	-2.32250881	1.33631074
1	1.49159622	-2.93850899	-1.16478920	6	-2.53230381	2.60919118	0.67171067
1	1.44399631	-2.95140886	1.53091073	1	-3.23780346	2.15399122	1.37111080
1	3.85409641	-2.14210892	2.41081071	1	-2.99320364	2.61949110	-0.32058927
13	0.55689633	0.28729105	-1.90858924	1	-2.38480377	3.64639115	0.98061067
6	0.39659631	2.16079116	-2.42878938	6	-6.03190374	-1.44720888	0.46251073
6	-0.14010370	-1.23320889	-2.91638947	1	-6.47570372	-0.46190894	0.29341072
1	0.81279629	2.29039121	-3.43568945	1	-6.72670364	-2.02610898	1.07481074
1	-0.65190375	2.47559118	-2.49458933	1	-5.94510365	-1.94520903	-0.50758928

I-5ppα-Cl

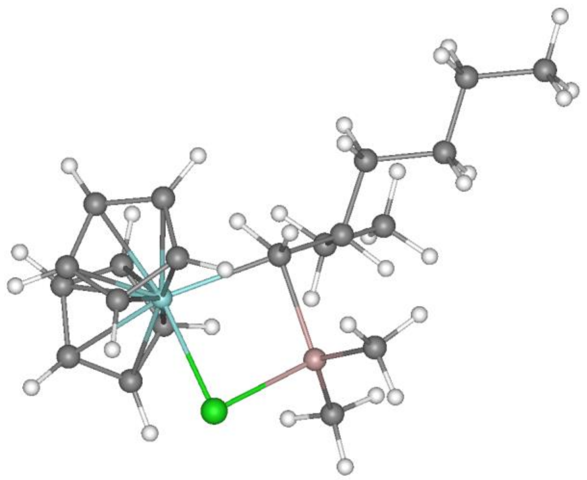

Zero-point vibrational energy	1275030.4 (Joules/Mol)
	304.73959 (Kcal/Mol)
Zero-point correction=	0.485633 (Hartree/Particle)
Thermal correction to Energy=	0.514279
Thermal correction to Enthalpy=	0.515223
Thermal correction to Gibbs Free Energy=	0.426600
Sum of electronic and zero-point Energies=	-5024.427301
Sum of electronic and thermal Energies=	-5024.398655
Sum of electronic and thermal Enthalpies=	-5024.397711
Sum of electronic and thermal Free Energies=	-5024.486334

cartesian

6	4.48234320	0.99796426	1.04477680	1	1.05284297	3.41516447	-0.74232322
6	3.51844311	1.97026420	0.66127676	1	-0.19665700	0.63826424	-4.28652287
6	2.36324310	1.77636421	1.44557679	1	0.09514302	-0.87523574	-3.44412327
6	2.61194301	0.69026423	2.33777690	1	-1.40735698	0.00886426	-3.18022323
6	3.92914319	0.23066425	2.10567689	1	0.53644300	-0.50793576	0.81107676
40	2.62434292	-0.31073573	0.04627681	1	-0.00335699	-1.13683581	-0.73952323
6	0.18514299	-0.19573574	-0.21192320	6	-1.22205687	0.40416425	0.12577681
17	2.84254313	0.81146425	-2.25372314	6	-2.20565701	-0.71743578	0.54187679
6	2.45324302	-2.43923569	-1.29342318	6	-1.12105703	1.50806427	1.18677676
6	3.83204317	-2.09383559	-1.25602317	1	-1.64595699	0.86146426	-0.78422320
6	4.27284288	-2.18453574	0.08327681	1	-0.64925700	1.08316422	2.08547688
6	3.16484308	-2.59023571	0.88387680	1	-0.46315700	2.29906440	0.81657678
6	2.05324292	-2.77053571	0.02227680	6	-3.60405707	-0.56803578	-0.06602320
1	1.46034300	2.36836433	1.40507674	1	-1.81265688	-1.69453573	0.23737679
1	1.92844296	0.31356427	3.09057689	1	-2.27355695	-0.74713576	1.63787675
1	3.64194298	2.70966434	-0.11992320	6	-4.59085703	-1.60673571	0.46627676
1	5.47974300	0.89496422	0.63347679	1	-3.52565694	-0.66493577	-1.15722322
1	4.42864275	-0.56673574	2.64077687	1	-3.99855685	0.43636426	0.12357680
1	5.27884293	-1.99573576	0.43677682	1	-4.69085693	-1.48383582	1.55107677
1	4.43124294	-1.79543579	-2.10732317	1	-4.18295717	-2.61143565	0.30437678
1	1.83094311	-2.45673561	-2.18032312	6	-2.45785689	2.14256430	1.57087684
1	1.06274307	-3.08523560	0.32907683	1	-3.14145684	1.41746426	2.01917672
1	3.18004298	-2.78353572	1.94927669	1	-2.94935703	2.57926440	0.69627678
13	0.51474303	1.13586426	-1.88732326	1	-2.30185699	2.94226432	2.29817677
6	0.38864303	3.04686427	-1.53212321	6	-5.96395731	-1.49533582	-0.19442320
6	-0.32765692	0.12886427	-3.32572317	1	-6.39865732	-0.50513577	-0.02992320
1	0.66944301	3.58736444	-2.44442320	1	-6.65955734	-2.23603559	0.20577680
1	-0.62755698	3.37266445	-1.28292322	1	-5.89195728	-1.65243578	-1.27452326

I-5ppβ-H

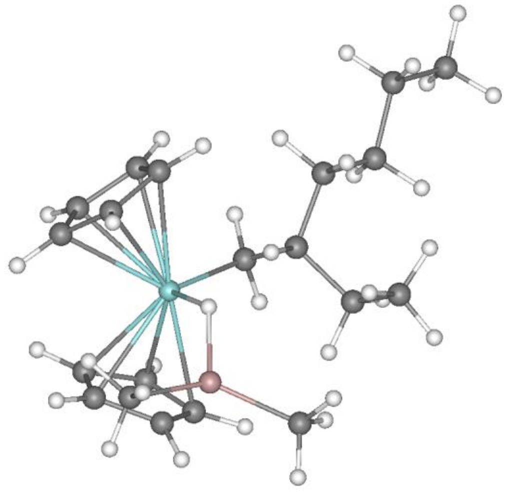

Zero-point vibrational energy	1285703.0 (Joules/Mol)
	307.29039 (Kcal/Mol)
Zero-point correction=	0.489698 (Hartree/Particle)
Thermal correction to Energy=	0.517408
Thermal correction to Enthalpy=	0.518352
Thermal correction to Gibbs Free Energy=	0.431283
Sum of electronic and zero-point Energies=	-4564.797004
Sum of electronic and thermal Energies=	-4564.769295
Sum of electronic and thermal Enthalpies=	-4564.768350
Sum of electronic and thermal Free Energies=	-4564.855420

cartesian

6	-0.00085723	-2.86897492	0.79341787	1	-2.99515724	4.06922531	0.07331786
6	-1.23375726	-3.35807490	0.29321784	1	-4.55895710	0.03382501	1.98551798
6	-2.27985716	-2.81337500	1.09281790	1	-4.73255730	1.64762497	2.66981792
6	-1.69585729	-1.96947503	2.05911779	1	6.14364290	-0.86877501	-1.22378218
6	-0.28455722	-1.98827493	1.86531782	1	-5.39565706	1.30492496	1.08651781
1	0.44264275	-1.45307493	2.46441793	6	0.74284273	-0.79437500	-1.24848211
1	0.97964281	-3.13837504	0.42711782	6	1.13684273	0.25922501	-0.22878215
1	-1.35005724	-4.06817532	-0.51618212	6	1.18464279	1.68622506	-0.80528212
1	-3.33765721	-3.01797509	0.98251784	6	2.39394283	-0.12377499	0.57821786
1	-2.22355723	-1.41227496	2.82291794	1	1.40534282	-1.65857494	-1.25978208
6	-2.06315732	0.35282499	-2.31748223	1	0.66514271	-0.38537499	-2.25718212
6	-1.98175716	-1.01657498	-2.64738202	1	1.96894288	1.72912502	-1.56818211
6	-2.97145724	-1.71017504	-1.89918208	1	0.24214274	1.88322496	-1.33208215
6	-3.67195725	-0.77277499	-1.10638213	6	3.69084263	0.02302501	-0.22018215
6	-3.11425710	0.51172501	-1.37108219	1	2.45584273	0.48232502	1.48991787
1	-3.59725714	1.46922505	-1.17038214	1	2.29724288	-1.16647506	0.90581787
1	-1.46765721	1.15042496	-2.74468207	6	4.89634275	-0.54947495	0.52761787
1	-1.29145718	-1.45997500	-3.35318208	1	3.87934303	1.08132505	-0.43848217
1	-3.17415714	-2.77267504	-1.94588208	1	3.58984280	-0.48107499	-1.19028211
1	-4.51425695	-0.97917497	-0.45668218	1	4.72604275	-1.61417496	0.72551787
40	-1.26575732	-0.92557496	-0.20288214	1	4.98094273	-0.06207499	1.50581789
1	-1.65945721	0.53772503	1.11371791	6	1.40924287	2.76292491	0.25431785
1	0.34384274	0.35622501	0.59041786	1	1.31224275	3.76262498	-0.17528215
13	-2.86965728	1.66552496	0.93121785	1	2.40554285	2.68852496	0.69831789
6	-2.19345713	3.40892506	0.42121783	1	0.67534274	2.67532492	1.06451786
6	-4.53965712	1.10072505	1.74041784	6	6.19904280	-0.37257499	-0.25048214
1	-1.72135735	3.91432500	1.27131784	1	7.04674292	-0.79567498	0.29291785
1	-1.43815732	3.35412502	-0.37058216	1	6.40914297	0.68612504	-0.42708215

I-5ppβ-Cl

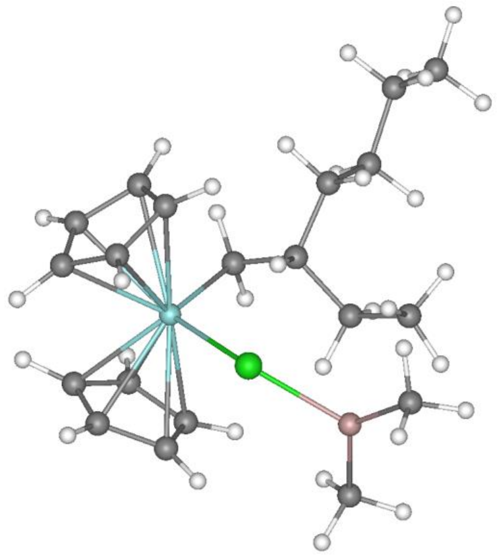

Zero-point vibrational energy	1270911.5 (Joules/Mol)
	303.75514 (Kcal/Mol)
Zero-point correction=	0.484065 (Hartree/Particle)
Thermal correction to Energy=	0.513411
Thermal correction to Enthalpy=	0.514356
Thermal correction to Gibbs Free Energy=	0.422425
Sum of electronic and zero-point Energies=	-5024.426614
Sum of electronic and thermal Energies=	-5024.397267
Sum of electronic and thermal Enthalpies=	-5024.396323
Sum of electronic and thermal Free Energies=	-5024.488254

cartesian

6	-0.52634823	-2.78612137	1.62479281	1	-3.03834820	3.73607850	-1.56190717
6	-1.69944811	-3.40342140	1.12609291	1	-1.94504821	5.10597849	2.24639273
6	-2.82124829	-2.72402143	1.67979288	1	-0.58234823	4.08457851	2.69309282
6	-2.34124804	-1.66992152	2.48369265	1	-0.38214821	5.35917854	1.47709286
6	-0.91774821	-1.69982147	2.44429302	6	0.33185184	-1.38172150	-0.86460710
1	-0.25524819	-1.03092146	2.98149300	6	0.84325182	-0.17432147	-0.07830715
1	0.48695183	-3.10202146	1.42069280	6	0.85875177	1.10917854	-0.92580712
1	-1.73244822	-4.26872158	0.47549284	6	2.17345190	-0.44052148	0.65429282
1	-3.86204815	-2.97092128	1.51339281	1	0.90485179	-2.28782129	-0.66920716
1	-2.94744825	-0.96192145	3.03569269	1	0.33015180	-1.19372153	-1.93970716
6	-2.32204819	-0.41482148	-2.27650738	1	1.55935192	0.98217845	-1.75730717
6	-2.35004807	-1.83642149	-2.23090696	1	-0.12814820	1.23707855	-1.38840711
6	-3.39644814	-2.22002149	-1.36190712	6	3.37395167	-0.54892147	-0.28700715
6	-4.00334835	-1.03972149	-0.84660715	1	2.35945177	0.34717855	1.39499283
6	-3.35294819	0.07007852	-1.44400716	1	2.07455182	-1.37362146	1.22289288
1	-3.60224819	1.10987854	-1.27320719	6	4.65075207	-0.96852148	0.44359285
1	-1.64574814	0.18237853	-2.87640715	1	3.55145192	0.41617849	-0.77900714
1	-1.69794822	-2.50402141	-2.77900696	1	3.15635180	-1.26872146	-1.08660710
1	-3.69074821	-3.23712134	-1.13620710	1	4.49185181	-1.94342148	0.91839284
1	-4.84594822	-0.99382150	-0.16790715	1	4.85345173	-0.25842148	1.25359285
40	-1.69994819	-1.12382150	0.10589285	6	5.85645199	-1.04162145	-0.49180716
17	-2.14654827	1.37957847	1.31709290	1	6.75885201	-1.33892155	0.04649284
1	0.14515179	0.06687853	0.77569282	1	6.04955196	-0.07112148	-0.95820713
13	-1.56714821	3.35557866	0.45969284	1	5.68855190	-1.76892149	-1.29140711
6	-1.97214818	3.51677871	-1.42560720	6	1.22135186	2.36377859	-0.13040715
6	-1.06384826	4.58897877	1.85059285	1	1.02395177	3.27747869	-0.70280713
1	-1.42364812	4.35137844	-1.87440717	1	2.28105187	2.38697863	0.13939285
1	-1.75254822	2.62057853	-2.01280737	1	0.68725181	2.39997864	0.83339286

TS-56pp-H

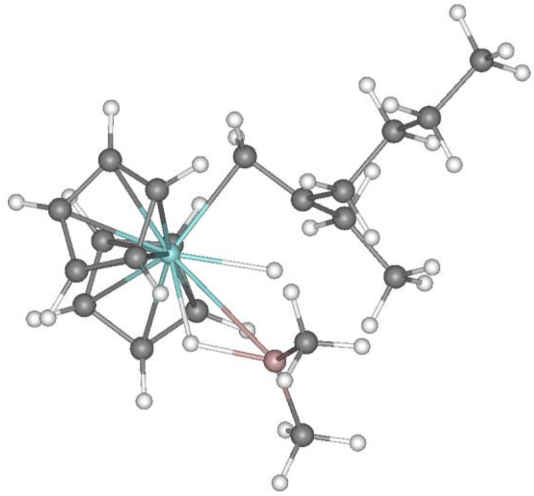

Zero-point vibrational energy	1276973.8 (Joules/Mol)
	305.20407 (Kcal/Mol)
Zero-point correction=	0.486374 (Hartree/Particle)
Thermal correction to Energy=	0.513319
Thermal correction to Enthalpy=	0.514263
Thermal correction to Gibbs Free Energy=	0.429911
Sum of electronic and zero-point Energies=	-4564.793310
Sum of electronic and thermal Energies=	-4564.766364
Sum of electronic and thermal Enthalpies=	-4564.765420
Sum of electronic and thermal Free Energies=	-4564.849773

cartesian

6	2.48650026	1.61367500	1.34110355	1	-2.07439995	0.48417497	-0.73809642
6	3.54620004	1.36877501	0.42170358	1	-1.56609988	-1.19452500	-0.64469641
6	4.19050026	0.17407498	0.80730361	1	0.26580012	-0.91702503	2.56700373
6	3.53990006	-0.32372501	1.97360361	1	-0.34339988	-2.03942490	1.26280355
6	2.50940013	0.58337498	2.31430364	13	0.61630011	1.54277503	-1.82299638
1	1.86970007	0.51047498	3.18340373	1	1.98970008	0.60527498	-1.49829638
1	1.82860017	2.47497511	1.33720362	6	-0.57199991	0.69937497	-3.11259627
1	3.81160021	1.99317491	-0.42189643	6	1.03820014	3.43117476	-1.63349640
1	5.04050016	-0.27702501	0.31110358	1	1.67440009	3.73217487	-2.47579646
1	3.81880021	-1.20802498	2.53350353	1	0.14660013	4.06447506	-1.68419635
6	3.14650011	-1.94472504	-1.51629639	1	1.58230019	3.69587517	-0.72169638
6	3.16980004	-2.65092516	-0.28459641	1	-1.52389979	1.23307502	-3.20789647
6	1.84850013	-3.04342508	0.02440359	1	-0.10969985	0.72237498	-4.10799646
6	1.00560009	-2.61162519	-1.04179645	1	-0.80299985	-0.34732503	-2.88979650
6	1.80860019	-1.95482504	-1.99639642	6	-3.15999985	-0.67682499	0.71790361
1	1.46780014	-1.52572501	-2.93069649	6	-4.24809980	-1.18212497	-0.23189640
1	4.00090027	-1.51832497	-2.02699637	1	-3.52929974	0.21187499	1.24380362
1	4.04730034	-2.85232496	0.31690359	1	-2.95429993	-1.43512499	1.48310363
1	1.54160011	-3.60972500	0.89460361	1	-3.89379978	-2.08742499	-0.73809642
1	-0.05759984	-2.79872513	-1.12629640	1	-4.41989994	-0.43452501	-1.01449645
40	1.88880014	-0.52022505	0.11140359	6	-1.44899988	2.52527475	1.03020358
6	-1.86859989	-0.33322501	-0.03989641	1	-1.63959980	3.33187485	1.74120367
6	-0.74609983	0.03097498	0.92300361	1	-2.37799978	2.34617519	0.48220357
6	-0.02059984	-1.03562498	1.52610362	1	-0.69609988	2.89267492	0.32460359
6	-0.97099984	1.27977502	1.76670361	6	-5.55809975	-1.47802496	0.49620357
1	-0.07149988	1.49787498	2.34780359	1	-5.41370010	-2.23492479	1.27210367
1	-1.72929978	0.97727501	2.49980354	1	-6.31739998	-1.84842503	-0.19539641
1	0.18650013	0.63827497	-0.12799640	1	-5.95239973	-0.57742500	0.97520363

TS-56pp-Cl

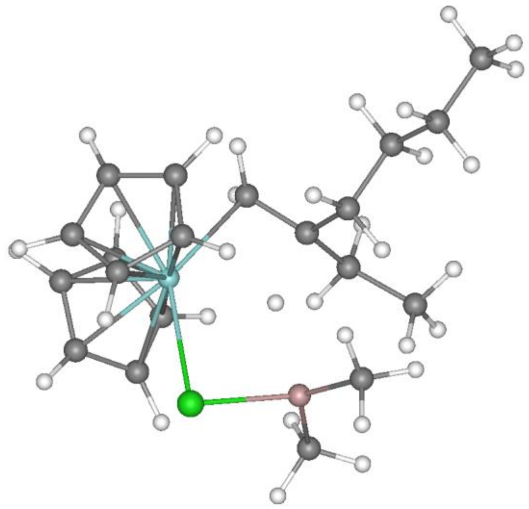

Zero-point vibrational energy	1260640.2 (Joules/Mol)
	301.30024 (Kcal/Mol)
Zero-point correction=	0.480152 (Hartree/Particle)
Thermal correction to Energy=	0.508587
Thermal correction to Enthalpy=	0.509532
Thermal correction to Gibbs Free Energy=	0.422021
Sum of electronic and zero-point Energies=	-5024.409974
Sum of electronic and thermal Energies=	-5024.381539
Sum of electronic and thermal Enthalpies=	-5024.380595
Sum of electronic and thermal Free Energies=	-5024.468106

cartesian

6	0.85526073	-2.59022856	-1.16351795	1	1.99346066	4.24497175	-0.06091788
6	2.06496072	-3.03452849	-0.57531786	1	-0.40633923	2.94827151	-3.38791776
6	3.13796091	-2.45162845	-1.31061792	1	-0.85433918	1.30927145	-2.94691777
6	2.58866072	-1.61972857	-2.30271792	1	-1.70003915	2.68637156	-2.22561789
6	1.16836071	-1.68252861	-2.20061779	6	0.04096079	-1.06392860	1.33878207
1	0.46546078	-1.16842854	-2.84601784	6	-0.72803921	-0.00952855	0.77488214
1	-0.13783926	-2.90322852	-0.87141788	6	-1.06083918	1.16377139	1.69598222
1	2.15756083	-3.74162841	0.24018213	6	-1.75293922	-0.33192855	-0.29401788
1	4.19386053	-2.62402844	-1.14761782	1	-0.26113921	-2.07642841	1.09028220
1	3.14656091	-1.03252852	-3.01961780	1	0.31586075	-0.93222857	2.38158226
6	2.63456082	0.53387141	2.12988210	1	-1.58353925	0.70837146	2.54608226
6	2.96206093	-0.84862858	2.24418211	1	-0.13573921	1.57557142	2.10678220
6	3.99386072	-1.12552857	1.32168221	6	-3.00693917	-0.95492858	0.34738213
6	4.27846050	0.06647145	0.60268211	1	-2.03573918	0.57407147	-0.83741790
6	3.45716071	1.09697139	1.13448215	1	-1.34483910	-1.02992857	-1.03001785
1	3.44966078	2.12967157	0.80818212	6	-4.10273933	-1.20182860	-0.69131786
1	1.90806079	1.06587148	2.73138213	1	-3.39693904	-0.29812855	1.13418221
1	2.52926087	-1.55562854	2.94008207	1	-2.74043918	-1.89912856	0.83728212
1	4.47986078	-2.08352852	1.19248223	1	-3.71533895	-1.86092854	-1.47671795
1	5.01926041	0.18587145	-0.17851788	1	-4.35733938	-0.25352854	-1.17811799
40	1.97376084	-0.58012855	-0.06831788	6	-1.93763924	2.27487159	1.12418222
17	2.52106071	1.44497144	-1.89311790	1	-2.04043913	3.07517147	1.85978222
1	0.33216077	0.75997144	-0.16921788	1	-2.94083929	1.91497147	0.88308209
13	0.63896078	2.39097166	-1.15871787	1	-1.52753925	2.72517157	0.21778211
6	1.06776071	3.71987152	0.20218211	6	-5.35613918	-1.81612861	-0.07061788
6	-0.71973926	2.31947160	-2.54581785	1	-5.13273954	-2.77832842	0.39858210
1	0.28546077	4.48027134	0.29498214	1	-6.12693930	-1.98302853	-0.82551789
1	1.21816075	3.29497147	1.20158219	1	-5.77533960	-1.15822852	0.69568211

I-6pp-H

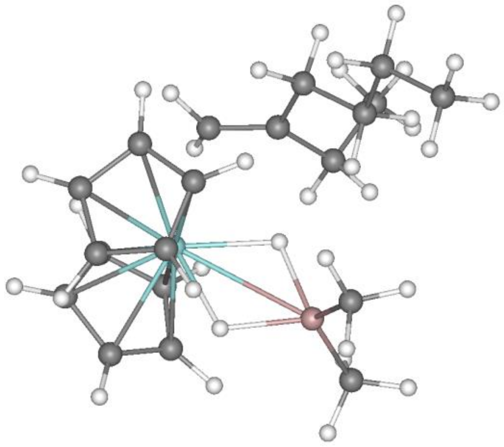

Zero-point vibrational energy	1280451.6 (Joules/Mol)
	306.03527 (Kcal/Mol)
Zero-point correction=	0.487698 (Hartree/Particle)
Thermal correction to Energy=	0.515378
Thermal correction to Enthalpy=	0.516322
Thermal correction to Gibbs Free Energy=	0.430317
Sum of electronic and zero-point Energies=	-4564.803800
Sum of electronic and thermal Energies=	-4564.776121
Sum of electronic and thermal Enthalpies=	-4564.775176
Sum of electronic and thermal Free Energies=	-4564.861181

cartesian

6	2.96923232	1.91133749	-0.14245892	1	0.75533229	1.93763745	-3.15405893
6	3.29763222	1.10773742	-1.25025892	1	1.46363235	0.73553741	-4.23525906
6	4.07853222	0.01073740	-0.78345895	1	-0.25226772	1.07903743	-4.31645870
6	4.26563263	0.17303741	0.61044109	6	-1.80306768	0.49703738	1.58104110
6	3.55433226	1.32443738	1.02104115	6	0.48053229	1.47573745	1.90024102
1	3.51853228	1.72133744	2.02834105	6	-0.68536770	1.43503737	1.19804108
1	2.39183235	2.82713723	-0.17765892	6	-0.98986769	2.48543739	0.15994111
1	3.00413227	1.28503740	-2.27765894	1	1.14573228	2.32423735	1.77114105
1	4.49273205	-0.77756256	-1.39945889	1	0.60323232	0.89463747	2.81074119
1	4.84213257	-0.47986260	1.25344110	1	0.16063231	0.21153739	-0.71915895
6	0.33043230	-2.21376276	0.98094106	1	-1.39076769	2.02063727	-0.74545890
6	1.33783233	-2.78446269	0.15814111	1	-0.06976771	3.00633740	-0.12135891
6	2.58553219	-2.60956264	0.80434108	1	-1.41326773	-0.31796259	2.20004106
6	2.34763241	-1.96686256	2.05374122	1	-2.45766759	1.07713747	2.24834108
6	0.95893228	-1.73866260	2.16384101	6	-2.01316762	3.49493742	0.70734107
1	0.46053231	-1.30296254	3.01954103	1	-2.97406769	3.01653719	0.91204107
1	-0.73346770	-2.20586276	0.77264106	1	-2.18326759	4.28483772	-0.02605890
1	1.17993236	-3.26656270	-0.79795891	1	-1.65436769	3.95493722	1.63134110
1	3.54533219	-2.94586277	0.43204111	6	-2.65756774	-0.06126260	0.43814111
1	3.09123230	-1.73046255	2.80484104	1	-3.17116761	0.75663745	-0.08145890
40	1.81263232	-0.30676261	0.25294110	1	-2.01986766	-0.55096257	-0.30975890
1	1.72513235	-0.99456257	-1.56185889	6	-3.69856763	-1.06166255	0.94374108
13	0.23393232	-0.45126259	-2.32055902	1	-3.19286776	-1.87516260	1.47914112
6	-0.97916770	-1.96386254	-2.53175902	1	-4.34646797	-0.56966257	1.67784107
6	0.58413231	0.96053743	-3.61985898	6	-4.54226780	-1.63906252	-0.19095889
1	-0.59976768	-2.66526270	-3.28465891	1	-5.08216763	-0.84796256	-0.71885890
1	-1.15746772	-2.54036260	-1.61735892	1	-5.27836800	-2.35196280	0.18584110
1	-1.95706773	-1.62896252	-2.89715886	1	-3.91386771	-2.16006279	-0.92025894

I-6pp-Cl

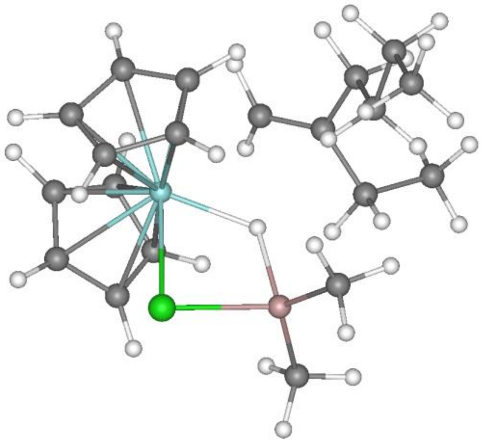

Zero-point vibrational energy	1260823.2 (Joules/Mol)
	301.34397 (Kcal/Mol)
Zero-point correction=	0.480222 (Hartree/Particle)
Thermal correction to Energy=	0.509108
Thermal correction to Enthalpy=	0.510052
Thermal correction to Gibbs Free Energy=	0.420944
Sum of electronic and zero-point Energies=	-5024.422693
Sum of electronic and thermal Energies=	-5024.393807
Sum of electronic and thermal Enthalpies=	-5024.392863
Sum of electronic and thermal Free Energies=	-5024.481970

cartesian

6	-3.03031969	1.47010350	-1.15663218	1	-1.49881983	3.48520350	0.79556793
6	-3.66151977	1.36430347	0.09606791	1	-1.22161973	3.88330364	2.48806787
6	-4.33851957	0.11310355	0.15056790	1	0.09528023	4.00930357	1.33376789
6	-4.17361975	-0.52159643	-1.10773218	6	2.07818031	-0.50499648	-1.51083219
6	-3.34041977	0.29100353	-1.90483212	6	-0.23961976	0.08630355	-2.25563216
1	-3.04241967	0.07600355	-2.92343211	6	0.93338031	0.45420355	-1.67383218
1	-2.43791986	2.31210351	-1.49503219	6	1.17658019	1.88660347	-1.27343214
1	-3.61041975	2.08830357	0.89956790	1	-0.92271972	0.86640358	-2.57493210
1	-4.91651964	-0.25779647	0.98856795	1	-0.34641975	-0.89209646	-2.72223210
1	-4.60221958	-1.46829653	-1.40973210	1	-0.39751974	0.67410356	0.52196789
6	-0.64631975	-2.31789637	1.18356788	1	1.56418037	1.92250359	-0.24783210
6	-2.02801967	-2.55759645	1.43786788	1	0.23138022	2.43880367	-1.28123212
6	-2.65141964	-2.86979628	0.21286792	1	1.80958033	-1.48429644	-1.91903210
6	-1.66961980	-2.79999638	-0.81553203	1	2.89798021	-0.14329645	-2.14523220
6	-0.42581975	-2.49799633	-0.19803210	6	2.19088030	2.56830359	-2.20743203
1	0.52748030	-2.40659642	-0.69903207	1	3.18368030	2.12280369	-2.11613202
1	0.09988028	-2.05959654	1.92626786	1	2.27658033	3.62500358	-1.94863224
1	-2.51101971	-2.50719643	2.40516782	1	1.87368035	2.49590373	-3.25063205
1	-3.69521976	-3.12429643	0.08456791	6	2.59108043	-0.62999642	-0.06743209
1	-1.83171964	-3.01059628	-1.86593223	1	1.74438024	-0.74979645	0.62226790
40	-1.91311979	-0.47199643	0.02286790	1	3.09048033	0.29990357	0.22916791
17	-2.10291982	0.34700352	2.62566781	6	3.56348038	-1.79649651	0.10726791
13	-0.25981975	1.56280351	2.00746799	1	3.06988025	-2.73149633	-0.18573208
6	1.31218028	0.92560357	2.96206784	1	4.40938044	-1.66749644	-0.57693207
6	-0.77291977	3.40120363	1.61216784	6	4.06588030	-1.90869653	1.54526782
1	1.34318042	1.35970354	3.96816778	1	4.57808018	-0.99319643	1.85456789
1	1.33508039	-0.16209646	3.08696795	1	4.76588011	-2.73899627	1.65786791
1	2.24528027	1.21900356	2.46766782	1	3.23558021	-2.07469654	2.23916793

S1.4. Zr-Al2 (η5-C5H5)2Zr-based catalytic species

I-0-HH

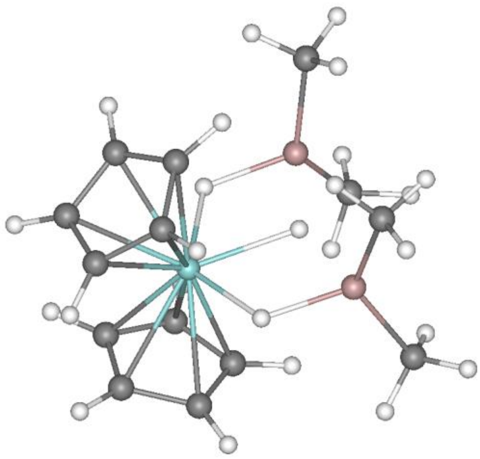

Zero-point vibrational energy	898440.1 (Joules/Mol)
	214.73234 (Kcal/Mol)
Zero-point correction=	0.342198 (Hartree/Particle)
Thermal correction to Energy=	0.365661
Thermal correction to Enthalpy=	0.366605
Thermal correction to Gibbs Free Energy=	0.288478
Sum of electronic and zero-point Energies=	-4573.407646
Sum of electronic and thermal Energies=	-4573.384183
Sum of electronic and thermal Enthalpies=	-4573.383239
Sum of electronic and thermal Free Energies=	-4573.461366

cartesian

6	-1.69726670	-2.21188569	-1.04008806	1	-0.28216669	-0.10748574	-1.79548812
6	-2.89036655	-1.67008567	-0.50788802	1	0.96563327	-0.00008574	0.00001196
6	-2.75876665	-1.62548566	0.91141200	13	1.44393337	-0.15688574	-1.77718806
6	-1.48796666	-2.15328574	1.24771190	6	2.21593332	1.55691433	-2.26258802
6	-0.81526673	-2.48628569	0.04271196	6	2.10483313	-1.97108567	-2.00168800
1	0.17123330	-2.92728567	-0.03978804	1	3.20093346	1.68751431	-1.80008805
1	-1.49176669	-2.39218569	-2.08768797	1	2.37713337	1.62261426	-3.34438801
1	-3.76496649	-1.37808573	-1.07548809	1	1.60393333	2.41741419	-1.97478807
1	-3.51536655	-1.29738569	1.61301196	1	2.51933336	-2.36268568	-1.06448805
1	-1.09586668	-2.27768564	2.24911189	1	1.32773328	-2.67098570	-2.32728815
6	-2.75876665	1.62571430	-0.91118801	1	2.90893316	-2.01708579	-2.74268818
6	-2.88996649	1.67041433	0.50821197	13	1.44393337	0.15681426	1.77721190
6	-1.69656670	2.21221423	1.04001188	6	2.10483313	1.97101426	2.00171185
6	-0.81486666	2.48621440	-0.04308804	6	2.21583319	-1.55708575	2.26261187
6	-1.48796666	2.15321422	-1.24788809	1	2.90873337	2.01701427	2.74301195
1	-1.09606671	2.27741432	-2.24938798	1	2.51963329	2.36241436	1.06461191
1	-3.51556683	1.29751432	-1.61258805	1	1.32763326	2.67091441	2.32691193
1	-3.76446676	1.37871432	1.07601190	1	2.37613344	-1.62318575	3.34451199
1	-1.49086666	2.39251423	2.08751202	1	1.60423326	-2.41758561	1.97391188
1	0.17173332	2.92711425	0.03911196	1	3.20133352	-1.68728566	1.80081189
40	-1.11656666	0.00001426	0.00001196	1	-0.28216669	0.10741426	1.79561198

I-0-HHa

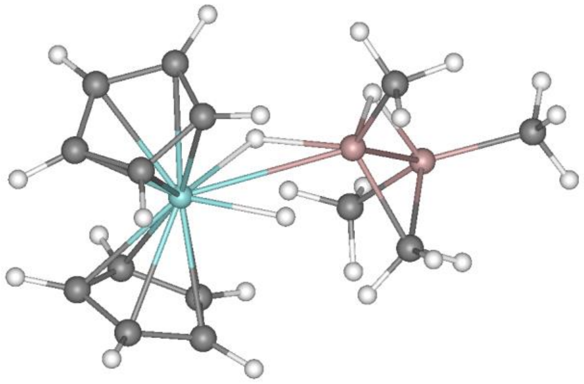

Zero-point vibrational energy 894899.9 (Joules/Mol)

213.88621 (Kcal/Mol)

Zero-point correction= 0.340849 (Hartree/Particle)

Thermal correction to Energy= 0.364245

Thermal correction to Enthalpy= 0.365189

Thermal correction to Gibbs Free Energy= 0.286784

Sum of electronic and zero-point Energies= -4573.376971

Sum of electronic and thermal Energies= -4573.353575

Sum of electronic and thermal Enthalpies= -4573.352631

Sum of electronic and thermal Free Energies= -4573.431036

cartesian

6	-4.01036692	0.85334766	0.52453572	6	4.99603319	0.57004762	0.20163569
6	-3.48206663	1.46234775	-0.64476430	1	-0.00276664	0.23454764	0.95993572
6	-2.36216664	2.25014758	-0.26426432	13	3.29613352	-0.29195234	0.56013572
6	-2.18256664	2.10604763	1.13733578	6	2.84433341	-2.04845238	1.28083575
6	-3.20376658	1.23984766	1.62303567	1	2.18783331	0.86094761	1.17033577
1	-3.35576653	0.94904763	2.65663552	1	1.87393332	-2.04925251	1.79103577
1	-4.88336658	0.21404764	0.56853575	1	2.81943345	-2.82355237	0.50553572
1	-3.88506651	1.37284768	-1.64666426	1	3.58413363	-2.37635231	2.01873565
1	-1.75916672	2.86234760	-0.92236429	13	1.09433341	1.01254773	-0.17706430
1	-1.42866659	2.59974766	1.73813570	6	2.10553336	-0.28835237	-1.40556431
6	-2.97126651	-2.30505252	0.22443569	6	1.06003332	2.91934752	-0.55656427
6	-2.73116660	-2.00485229	-1.14706433	1	1.52873349	-1.20785224	-1.53866422
6	-1.33146667	-2.07555246	-1.37356424	1	1.91923332	0.33864763	-2.29056430
6	-0.70426667	-2.39765239	-0.13906431	1	3.15523338	-0.57155234	-1.58476424
6	-1.72046661	-2.54815245	0.84193569	1	0.62623340	3.13954759	-1.53806424
1	-1.56186652	-2.80665231	1.88323569	1	0.50683337	3.50104761	0.18903570
1	-3.93996668	-2.36615252	0.70493573	1	2.08073354	3.31854749	-0.56036431
1	-3.48836660	-1.80075228	-1.89456427	1	-0.51546663	0.50754762	-1.14136434
1	-0.83376664	-1.92415226	-2.32306433	1	5.65833330	-0.05975236	-0.40226430
1	0.35683334	-2.54315233	0.02653570	1	5.53453350	0.79554766	1.12783575
40	-1.74406672	-0.13685235	0.14863570	1	4.86943340	1.51794767	-0.33236432

I-0-HHb

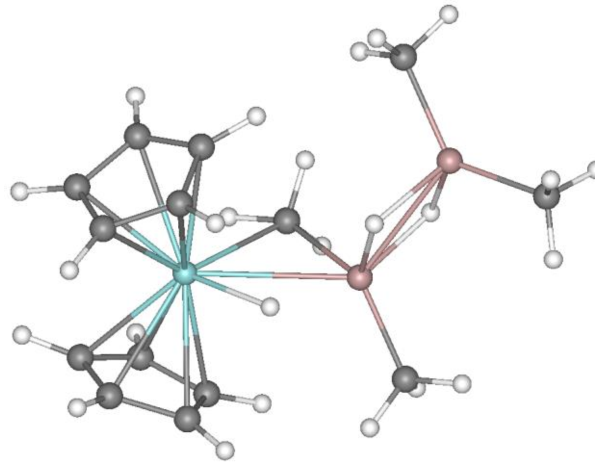

Zero-point vibrational energy	898557.1 (Joules/Mol)
	214.76029 (Kcal/Mol)
Zero-point correction=	0.342242 (Hartree/Particle)
Thermal correction to Energy=	0.365334
Thermal correction to Enthalpy=	0.366278
Thermal correction to Gibbs Free Energy=	0.288549
Sum of electronic and zero-point Energies=	-4573.374976
Sum of electronic and thermal Energies=	-4573.351884
Sum of electronic and thermal Enthalpies=	-4573.350940
Sum of electronic and thermal Free Energies=	-4573.428669

cartesian

6	3.94374061	-0.58069044	-0.31072381	6	-4.85415936	-0.63859046	0.93307620
6	3.17424059	-1.35409045	-1.21522379	1	0.30514055	-0.50219041	1.10587621
6	2.27844048	-2.15789032	-0.46142381	13	-3.42925954	0.27600956	-0.01482379
6	2.51374054	-1.90149045	0.91587621	6	-3.35415959	2.03750968	-0.86022383
6	3.53314042	-0.91979045	1.00997627	1	-1.94605947	0.12360954	0.97557622
1	3.95534062	-0.52919042	1.92757630	1	-3.22525954	2.83870959	-0.12382380
1	4.73364067	0.11110955	-0.57582378	1	-2.56775951	2.14680958	-1.61512375
1	3.26064062	-1.33829045	-2.29592371	1	-4.30105972	2.24430966	-1.37102377
1	1.56844068	-2.87279034	-0.86072379	13	-1.12245941	-1.05249047	0.04277620
1	2.01284051	-2.38079047	1.74687624	1	-2.54005957	-0.85519046	-0.98622376
6	2.25614047	2.13320971	1.39117622	6	-1.11685944	-2.95689034	0.43257621
6	2.59714055	2.49840975	0.05797621	1	-1.04355955	-3.54899049	-0.48722380
6	1.39104056	2.64380956	-0.67372382	1	-0.30845946	-3.27439046	1.09817624
6	0.30844057	2.35610962	0.19657621	1	-2.05775952	-3.25239038	0.90967619
6	0.84254056	2.05190969	1.47757626	1	-5.71835947	-0.80659044	0.28237620
1	0.26774055	1.80970955	2.36227632	1	-5.20745945	-0.05379046	1.78817630
1	2.95344043	1.97980952	2.20527625	1	-4.53765965	-1.61549044	1.31127620
1	3.59634042	2.67550969	-0.32032380	6	-0.08995944	-0.26049045	-1.70152378
1	1.31084061	2.93000960	-1.71652377	1	-0.78745943	0.51700956	-2.02682376
1	-0.74445945	2.38910961	-0.06012379	1	0.88294053	0.03530955	-2.16772366
40	1.59714055	0.22090954	-0.05252380	1	-0.33085942	-1.16639054	-2.27312374

I-0-HCl-center

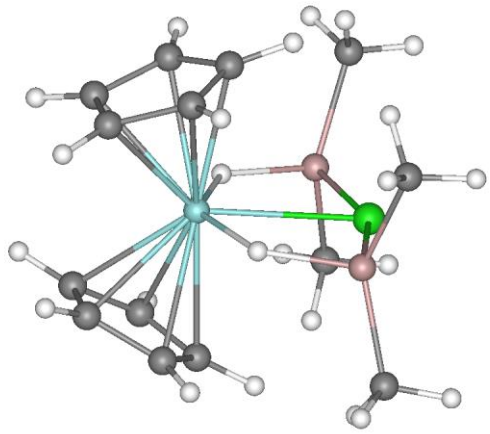

Zero-point vibrational energy 876986.5 (Joules/Mol)

209.60482 (Kcal/Mol)

Zero-point correction= 0.334027 (Hartree/Particle)

Thermal correction to Energy= 0.357184

Thermal correction to Enthalpy= 0.358128

Thermal correction to Gibbs Free Energy= 0.278707

Sum of electronic and zero-point Energies= -5033.032772

Sum of electronic and thermal Energies= -5033.009615

Sum of electronic and thermal Enthalpies= -5033.008671

Sum of electronic and thermal Free Energies= -5033.088091

cartesian

6	-0.71435958	-2.63070941	-1.62805474	6	-2.98605943	1.88949060	-1.78625476
6	0.71124041	-2.63160944	-1.62715471	1	-1.84045959	-0.19070935	-0.00225478
6	1.14864039	-1.40320933	-2.17885470	13	-2.38355947	1.43169057	-0.00185478
6	0.00034042	-0.62680936	-2.49125481	6	-2.98805952	1.88649058	1.78284526
6	-1.14945960	-1.40170932	-2.18015480	17	0.00294042	1.92179060	-0.00125478
1	-2.17775941	-1.10270941	-2.33895469	1	-2.26025939	1.62999058	2.55944538
1	-1.35255957	-3.44640946	-1.31355476	1	-3.18595958	2.95989060	1.86924529
1	1.34814036	-3.44800949	-1.31185472	1	-3.92515945	1.37409067	2.02754521
1	2.17754054	-1.10540938	-2.33645463	13	2.38804054	1.42429066	0.00154522
1	0.00124042	0.36519065	-2.92755485	6	2.99164057	1.87929058	1.78634524
6	-0.71925956	-2.62850928	1.62764525	6	2.99424052	1.87839067	-1.78265476
6	0.70634043	-2.63170934	1.62864530	1	3.93044043	1.36959064	2.03024530
6	1.14504039	-1.40370941	2.18004537	1	3.18634057	2.95329070	1.87354529
6	-0.00245958	-0.62530935	2.49064517	1	2.26504040	1.61999059	2.56324530
6	-1.15305960	-1.39840937	2.17854524	1	3.93294048	1.36789060	-2.02535462
1	-2.18115950	-1.09750938	2.33594537	1	2.26814055	1.61939061	-2.56005478
1	-1.35845959	-3.44330931	1.31284523	1	3.18984056	2.95219064	-1.86995471
1	1.34214044	-3.44950938	1.31484532	1	1.83984041	-0.19640934	0.00134522
1	2.17424059	-1.10760939	2.33894515	1	-2.26375961	1.62139058	-2.56405473
1	-0.00055958	0.36689067	2.92644525	1	-3.17015958	2.96519065	-1.87495470
40	-0.00135958	-0.89590931	-0.00025478	1	-3.93045950	1.38879061	-2.02715468

I-0-HCl-side

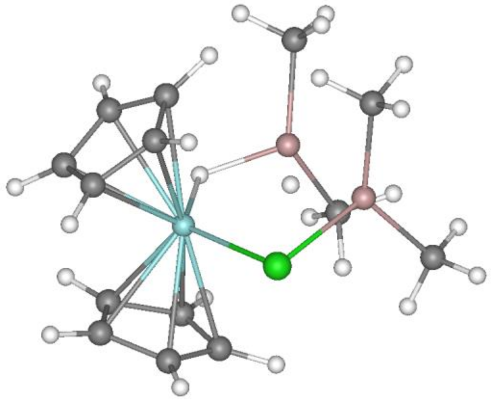

Zero-point vibrational energy	880567.6 (Joules/Mol)
	210.46070 (Kcal/Mol)
Zero-point correction=	0.335390 (Hartree/Particle)
Thermal correction to Energy=	0.360258
Thermal correction to Enthalpy=	0.361202
Thermal correction to Gibbs Free Energy=	0.280575
Sum of electronic and zero-point Energies=	-5033.031772
Sum of electronic and thermal Energies=	-5033.006904
Sum of electronic and thermal Enthalpies=	-5033.005960
Sum of electronic and thermal Free Energies=	-5033.086587

cartesian

6	-1.77106428	-1.78747857	-1.48394525	1	-0.49276429	0.44172147	-1.49874520
6	-2.92296433	-1.58867848	-0.69184518	1	0.91563570	0.00752145	0.09645481
6	-2.63826442	-2.05227852	0.62715483	13	1.22943568	0.56582147	-1.60924518
6	-1.31706429	-2.56647873	0.62825483	6	1.76583576	2.43032146	-1.51064515
6	-0.76546431	-2.37577868	-0.65984523	6	1.96303558	-0.96217853	-2.56184506
1	0.22763571	-2.66647863	-0.98254526	1	2.85033560	2.54152131	-1.61494517
1	-1.67116427	-1.54967856	-2.53524518	1	1.31663573	3.01772141	-2.32034516
1	-3.86506438	-1.17867851	-1.03334522	1	1.48293567	2.90622139	-0.56514513
1	-3.32526422	-2.06507874	1.46475482	1	2.45193577	-1.67187858	-1.88374531
1	-0.80906427	-2.99667859	1.48195481	1	1.19483566	-1.51847851	-3.11024499
6	-2.85406423	1.65762150	-0.40514520	1	2.71653557	-0.65227854	-3.29274511
6	-3.27596426	1.18682146	0.87085485	13	2.00663567	-0.38097855	1.56325483
6	-2.29786420	1.55472147	1.81785476	6	2.90443563	1.31622148	1.82565475
6	-1.27006423	2.27352142	1.13705480	6	2.66743565	-2.17017865	1.21175480
6	-1.63466430	2.36492133	-0.22544518	1	3.51273561	1.29752147	2.73665500
1	-1.07596433	2.87112141	-1.00264525	1	3.58613563	1.54862142	0.99965477
1	-3.39296436	1.55292141	-1.33864522	1	2.20713568	2.15422130	1.92345476
1	-4.18816423	0.64182144	1.07965481	1	3.06293559	-2.60897875	2.13525486
1	-2.31936431	1.33282149	2.87735486	1	1.89743567	-2.85657859	0.84455478
1	-0.38806430	2.70422125	1.59665477	1	3.49063563	-2.17307854	0.48965484
40	-1.20226431	-0.07247855	0.25825483	17	0.02723572	-0.47357857	2.66015482

I-0-HCla

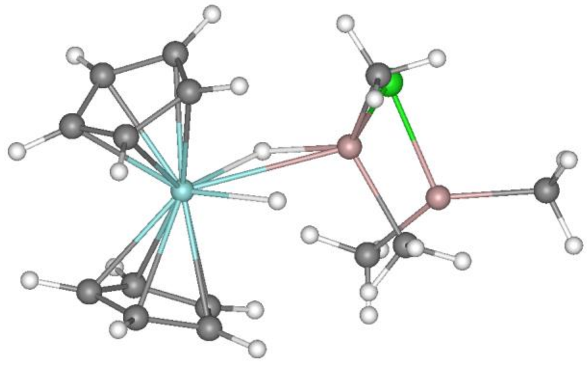

Zero-point vibrational energy 879705.0 (Joules/Mol)

210.25453 (Kcal/Mol)

Zero-point correction= 0.335062 (Hartree/Particle)

Thermal correction to Energy= 0.359592

Thermal correction to Enthalpy= 0.360536

Thermal correction to Gibbs Free Energy= 0.280281

Sum of electronic and zero-point Energies= -5033.002659

Sum of electronic and thermal Energies= -5032.978129

Sum of electronic and thermal Enthalpies= -5032.977185

Sum of electronic and thermal Free Energies= -5033.057440

cartesian

6	3.93201375	-1.10615003	0.80519050	6	-5.25398636	0.04845001	-0.26100948
6	3.53791380	-1.54434991	-0.48870951	1	-0.05798608	-0.36504999	0.91299051
6	2.36131382	-2.32835007	-0.34910947	13	-3.50658607	0.47905001	0.44649053
6	2.01241398	-2.35105014	1.02799058	6	-2.80458617	2.06204987	1.33039045
6	2.98791385	-1.59624994	1.73939061	17	-2.61808610	-1.36094999	1.44059062
1	3.01481390	-1.44075000	2.81219053	1	-1.83978593	1.86345005	1.81139040
1	4.80951405	-0.51615000	1.03879046	1	-2.68568611	2.91224980	0.64879048
1	4.06561375	-1.35014999	-1.41480947	1	-3.48388624	2.39415002	2.12329054
1	1.83111393	-2.83144999	-1.14720941	13	-1.08118606	-1.05394995	-0.32960951
1	1.17941391	-2.88485003	1.46969056	6	-2.01208615	0.42275003	-1.37230945
6	3.10881376	2.10104990	0.50649047	6	-0.98788607	-2.85915017	-1.04000950
6	2.85041380	1.91345012	-0.88070947	1	-1.49288607	1.38215005	-1.29680943
6	1.45611393	2.07774997	-1.09430957	1	-1.68128610	-0.02934999	-2.32160950
6	0.84971392	2.34134984	0.16449051	1	-3.05418611	0.64775002	-1.65010953
6	1.87491393	2.36284995	1.14839053	1	-0.46038607	-2.90965009	-1.99860954
1	1.73451400	2.55504990	2.20649052	1	-0.50598609	-3.56704998	-0.35610950
1	4.08061409	2.07445002	0.98339051	1	-2.00038624	-3.24454999	-1.20620942
1	3.59421372	1.72485006	-1.64550948	1	0.61431396	-0.46425000	-1.14290953
1	0.95001388	2.02635002	-2.04980946	1	-5.66178608	0.84675002	-0.89040953
1	-0.20048606	2.53694987	0.34509051	1	-5.97198629	-0.11124999	0.55059052
40	1.75791395	0.01245001	0.28019053	1	-5.24248600	-0.87064993	-0.85640949

I-0-HClb

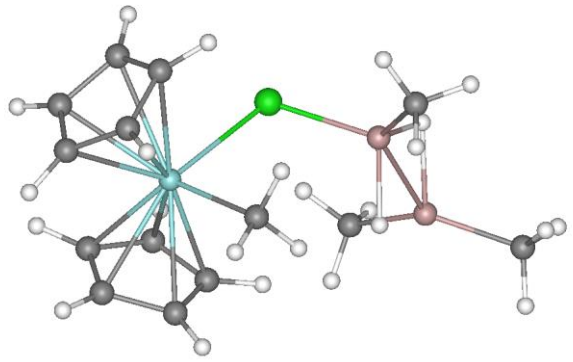

Zero-point vibrational energy 879013.6 (Joules/Mol)

210.08930 (Kcal/Mol)

Zero-point correction= 0.334799 (Hartree/Particle)

Thermal correction to Energy= 0.360165

Thermal correction to Enthalpy= 0.361109

Thermal correction to Gibbs Free Energy= 0.276719

Sum of electronic and zero-point Energies= -5033.001839

Sum of electronic and thermal Energies= -5032.976473

Sum of electronic and thermal Enthalpies= -5032.975529

Sum of electronic and thermal Free Energies= -5033.059919

cartesian

6	4.14971924	-0.48230714	-0.03010476	6	-5.38668060	0.15539289	-0.60420477
6	3.45801926	-1.64170718	-0.46990478	17	-0.33038086	-0.72120708	1.61779523
6	2.73161912	-2.15830708	0.62839520	13	-3.78298092	0.54069293	0.40879524
6	2.99611926	-1.33840716	1.75789523	6	-3.08348083	2.03019285	1.44269526
6	3.87081933	-0.30340713	1.35519528	1	-3.30988073	-0.97800708	1.25789523
1	4.27371931	0.47239289	1.99349523	1	-2.14778090	1.77719283	1.95209527
1	4.81611919	0.12599289	-0.62860477	1	-2.90128088	2.90789294	0.81289524
1	3.47471929	-2.05180717	-1.47140479	1	-3.79708099	2.34129286	2.21269512
1	2.08981919	-3.03110719	0.61469525	13	-2.00088072	-1.40010715	0.28139526
1	2.58911920	-1.47860718	2.75259519	1	-2.36278081	-0.01370711	-0.60550475
6	2.35901928	2.50109291	0.52619523	6	-1.86048079	-3.14220715	-0.53020477
6	2.51351929	2.25769281	-0.86910474	1	-1.63868093	-3.06390715	-1.59830475
6	1.22391915	2.04149294	-1.42140472	1	-1.06958079	-3.73770714	-0.06390476
6	0.28261915	2.11329293	-0.36760473	1	-2.79698086	-3.69770718	-0.42400473
6	0.98331916	2.40929294	0.83329523	1	-5.55958080	0.91299289	-1.37470472
1	0.53941917	2.53569293	1.81439519	1	-6.26908064	0.15059289	0.04329523
1	3.15351915	2.73059297	1.22469521	1	-5.34148073	-0.81750709	-1.10270476
1	3.44381928	2.28959274	-1.42230475	6	0.80381918	-0.90380710	-1.68710470
1	1.00291920	1.84249282	-2.46180487	1	-0.17338085	-0.47400713	-1.94940472
1	-0.78908080	1.97869289	-0.45950478	1	1.42491913	-0.80420709	-2.58750486
40	1.73621917	0.10659289	0.11429524	1	0.68471915	-1.98140717	-1.51540470

I-0-HClc

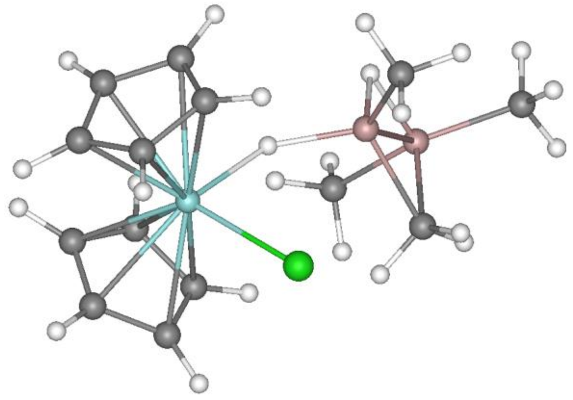

Zero-point vibrational energy	882582.4 (Joules/Mol)
	210.94225 (Kcal/Mol)
Zero-point correction=	0.336158 (Hartree/Particle)
Thermal correction to Energy=	0.360894
Thermal correction to Enthalpy=	0.361838
Thermal correction to Gibbs Free Energy=	0.281545
Sum of electronic and zero-point Energies=	-5033.008319
Sum of electronic and thermal Energies=	-5032.983583
Sum of electronic and thermal Enthalpies=	-5032.982639
Sum of electronic and thermal Free Energies=	-5033.062931

cartesian

6	3.94099283	-0.75460476	0.81541431	6	-5.12860680	-0.35420480	0.30531430
6	3.59019279	-1.74050474	-0.14318568	1	-0.02170712	-0.19470477	0.41471428
6	2.38469291	-2.35570478	0.27771431	13	-3.37100720	0.45449522	0.37671429
6	1.99259293	-1.75660479	1.50201428	6	-2.69480705	2.24429512	0.73421431
6	2.94679284	-0.75840479	1.83161426	1	-2.30240703	-0.66270477	1.20901430
1	2.93669295	-0.13570479	2.71761441	1	-1.64670706	2.22149515	1.05421436
1	4.82529259	-0.12990478	0.79031432	1	-2.76110721	2.89569521	-0.14468569
1	4.13869286	-1.97810471	-1.04728568	1	-3.25820708	2.73839521	1.53221428
1	1.85589290	-3.13740468	-0.25318569	13	-1.33070707	-1.14280474	-0.09928568
1	1.12399292	-2.02150488	2.09251428	6	-2.28390718	-0.08920478	-1.56568575
6	2.78189301	2.23279524	0.55151433	6	-1.07610714	-3.05680466	-0.22428568
6	3.10819292	2.09979534	-0.82718569	1	-1.56050706	0.63369524	-1.95488572
6	1.89889288	2.18589520	-1.57178569	1	-2.31330705	-0.92480475	-2.27998567
6	0.83259290	2.31779528	-0.65848565	1	-3.26820707	0.37069520	-1.75138569
6	1.37869287	2.34469533	0.66031432	1	-0.51560712	-3.32830477	-1.12448561
1	0.81879294	2.45849514	1.58141434	1	-0.56030715	-3.48190475	0.64281428
1	3.48549294	2.23649526	1.37441432	1	-2.04900718	-3.55770469	-0.28498566
1	4.10549259	2.00949526	-1.24148571	17	1.05659294	-0.88200474	-2.36698580
1	1.80969286	2.11309528	-2.64828563	1	-5.81530666	0.21779521	-0.32758570
1	-0.21810710	2.40469527	-0.90908569	1	-5.58560753	-0.40960479	1.29841435
40	1.84419286	0.04859522	-0.24878567	1	-5.09510708	-1.37520480	-0.08948568

I-0-HCld

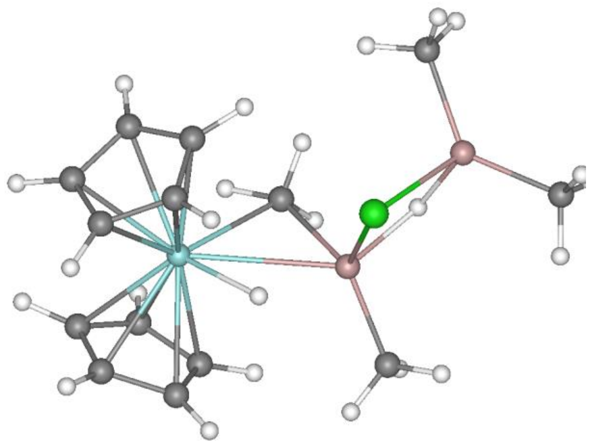

Zero-point vibrational energy 882356.6 (Joules/Mol)

210.88828 (Kcal/Mol)

Zero-point correction= 0.336072 (Hartree/Particle)

Thermal correction to Energy= 0.360493

Thermal correction to Enthalpy= 0.361437

Thermal correction to Gibbs Free Energy= 0.281293

Sum of electronic and zero-point Energies= -5033.006707

Sum of electronic and thermal Energies= -5032.982287

Sum of electronic and thermal Enthalpies= -5032.981343

Sum of electronic and thermal Free Energies= -5033.061486

cartesian

6	-3.98047161	0.67995721	-0.33802143	6	5.18192816	0.80075717	0.45827860
6	-3.12317157	1.43605709	-1.17612147	1	-0.41237158	0.40615720	1.24487853
6	-2.23697162	2.17725706	-0.35132143	13	3.71262836	-0.25124282	-0.23532140
6	-2.56417155	1.89945722	1.00357854	6	3.64652848	-1.86584282	-1.31992149
6	-3.63347149	0.96805722	1.01277864	17	2.17452836	-0.59994280	1.55387855
1	-4.12587166	0.57485718	1.89347851	1	4.17942810	-2.68434286	-0.82362139
1	-4.78547192	0.03485718	-0.66712141	1	2.63092852	-2.22454286	-1.52062142
1	-3.14567161	1.44965708	-2.26002121	1	4.13232803	-1.71834290	-2.29032135
1	-1.47247159	2.86635709	-0.69052136	13	1.06992841	0.92555720	0.26407859
1	-2.08937168	2.33265710	1.87457860	1	2.44922853	0.79655719	-0.78452146
6	-2.44317150	-2.10844278	1.49817860	6	1.10242844	2.81385708	0.71477854
6	-2.86867166	-2.43834281	0.18067858	1	1.07712841	3.44165707	-0.18372142
6	-1.71107161	-2.66884279	-0.60462141	1	0.28722847	3.13355708	1.37107861
6	-0.57187158	-2.47264290	0.22037858	1	2.03872848	3.05685711	1.22997856
6	-1.02487159	-2.14024282	1.52457857	1	5.82802820	1.17415714	-0.34262142
1	-0.39397156	-1.95264292	2.38417864	1	5.81182814	0.19915719	1.12207854
1	-3.08917165	-1.90444291	2.34297872	1	4.83782816	1.66595709	1.03337860
1	-3.89407158	-2.53384280	-0.15402141	6	0.08012842	0.16955717	-1.53212142
1	-1.69917154	-2.95464277	-1.65062141	1	0.78002846	-0.61374283	-1.83842146
1	0.46372843	-2.58994293	-0.07622142	1	-0.87547153	-0.11504281	-2.04062128
40	-1.69827151	-0.24424282	0.03627859	1	0.35472846	1.07835710	-2.08362126

I-1-HH

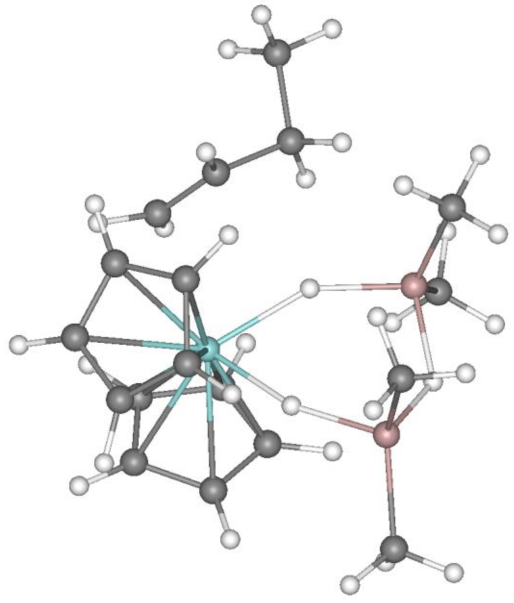

Zero-point vibrational energy	1189060.9 (Joules/Mol)
	284.19237 (Kcal/Mol)
Zero-point correction=	0.452889 (Hartree/Particle)
Thermal correction to Energy=	0.483083
Thermal correction to Enthalpy=	0.484027
Thermal correction to Gibbs Free Energy=	0.391706
Sum of electronic and zero-point Energies=	-4730.440734
Sum of electronic and thermal Energies=	-4730.410540
Sum of electronic and thermal Enthalpies=	-4730.409596
Sum of electronic and thermal Free Energies=	-4730.501917

cartesian

6	2.15147424	-2.10978317	-0.19224629	40	-0.53982592	-1.18148315	-0.02384629
6	2.47017407	-1.02818322	0.54865372	6	1.17147410	3.06691694	1.32835364
6	3.27487421	0.12691683	0.02995371	1	0.45387405	0.52261686	0.08645371
1	1.78397405	-3.03208303	0.25035372	13	0.64447403	2.19681692	-0.34174627
1	2.45037413	-2.15598321	-1.23674631	6	1.47857404	2.32401681	-2.10694623
1	2.94387412	1.05871677	0.50015372	1	-1.00902593	2.51861691	-0.64564633
1	3.14517426	0.22121683	-1.05274630	1	1.29077399	1.46261680	-2.75794625
6	-0.02172594	-2.42158318	2.13645387	1	1.10197401	3.20451689	-2.64024615
6	-0.99942595	-3.16658306	1.44055367	1	2.56487417	2.44711685	-2.03584623
6	-2.19392586	-2.38818312	1.40765369	13	-2.38362575	1.88601673	0.12135370
6	-1.94182599	-1.17208314	2.08425379	6	-3.83692575	1.86161673	-1.17824626
6	-0.58432597	-1.17038321	2.50375390	6	-2.36652589	2.40721703	2.00095391
1	-0.09322594	-0.38278317	3.06295371	1	-4.64362574	1.18701684	-0.86914629
1	0.98317409	-2.75618315	2.36355376	1	-4.28612566	2.85621691	-1.27434635
1	-0.87922597	-4.16828299	1.04575372	1	-3.52062583	1.56251681	-2.18314624
1	-3.14282584	-2.69348311	0.98445368	1	-3.23972583	2.04081702	2.55255389
1	-2.65732574	-0.38068318	2.26495385	1	-1.46862590	2.06041694	2.52615380
6	-0.27982596	-2.62828302	-2.04644608	1	-2.37962580	3.49921703	2.09865379
6	-1.66342592	-2.62898302	-1.74504626	1	-1.73952591	0.27581683	0.09515370
6	-2.15892601	-1.31678319	-1.93464625	1	2.27107406	-1.03338313	1.62085366
6	-1.08252597	-0.50618315	-2.39904618	1	0.49387401	3.88821673	1.58685374
6	0.07177405	-1.31168318	-2.46924615	1	2.17677426	3.49771690	1.26095366
1	1.05097401	-0.98068321	-2.79404616	1	1.16997409	2.37951684	2.18215370
1	0.37297404	-3.49218297	-2.02614617	6	4.76007414	-0.09208317	0.36835372
1	-2.24082589	-3.48548317	-1.42064631	1	4.90607405	-0.20038317	1.44575369
1	-3.17972589	-0.98588312	-1.78464627	1	5.35057402	0.76171684	0.03145371
1	-1.13992596	0.54761684	-2.65024614	1	5.13957405	-0.99198318	-0.12044629

I-1-HCl

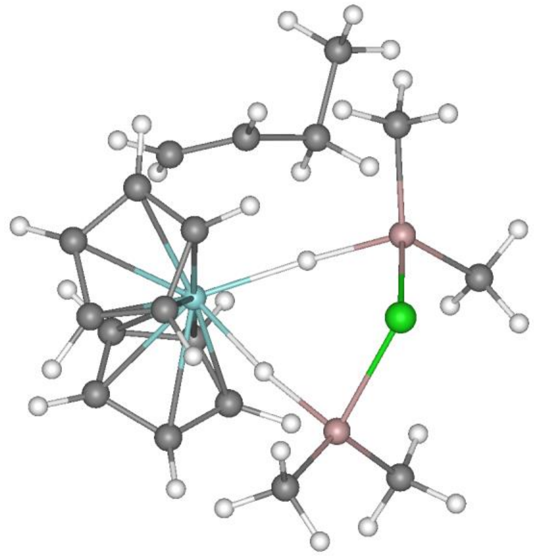

Zero-point vibrational energy	1173598.0 (Joules/Mol)
	280.49665 (Kcal/Mol)
Zero-point correction=	0.447000 (Hartree/Particle)
Thermal correction to Energy=	0.478137
Thermal correction to Enthalpy=	0.479081
Thermal correction to Gibbs Free Energy=	0.385397
Sum of electronic and zero-point Energies=	-5190.071804
Sum of electronic and thermal Energies=	-5190.040667
Sum of electronic and thermal Enthalpies=	-5190.039723
Sum of electronic and thermal Free Energies=	-5190.133406

cartesian

6	2.86440754	-1.07819450	0.27597591	40	-0.03089255	-1.23959446	0.18607591
6	2.63640761	0.24450552	0.41127589	6	0.87410742	2.93360543	1.86957586
6	2.98250747	1.24960554	-0.65062404	1	0.11870745	0.72460556	0.02527591
1	2.83760762	-1.76619446	1.11567593	13	-0.17389256	2.40510559	0.31057590
1	3.27610755	-1.46409440	-0.65342414	6	-0.18959256	3.27010560	-1.43802416
1	2.18420744	1.98620558	-0.78242409	17	-2.35549259	2.16470551	1.07497597
1	3.11900759	0.74660552	-1.61292410	1	0.05190745	2.58480549	-2.25862408
6	0.68140745	-1.43139446	2.60907602	1	-1.16329253	3.71680546	-1.66702414
6	0.37130743	-2.73189449	2.13797593	1	0.54630744	4.08190536	-1.47272408
6	-1.02999258	-2.77309442	1.88177586	13	-3.15309238	0.52200556	-0.34282410
6	-1.56799245	-1.49959445	2.17607594	6	-3.08189249	1.14160550	-2.18502402
6	-0.50449252	-0.65859449	2.60577607	6	-4.60589266	-0.41419449	0.55347592
1	-0.60349256	0.37250552	2.92357612	1	-3.33559251	0.34010550	-2.88952398
1	1.65720749	-1.08269453	2.92597604	1	-3.82609248	1.93170547	-2.34082389
1	1.06040740	-3.56289434	2.04507589	1	-2.11539245	1.55940557	-2.48292398
1	-1.59589267	-3.63799453	1.56067586	1	-4.54479265	-1.50149441	0.42217591
1	-2.61189246	-1.21999443	2.11287594	1	-4.65859270	-0.21389449	1.62867594
6	0.96970743	-2.90389442	-1.39122415	1	-5.56899261	-0.10669449	0.13107590
6	-0.34669256	-3.36209440	-1.14392412	1	-1.71749258	-0.41269448	-0.09412409
6	-1.25199258	-2.41189456	-1.67052412	1	0.89420742	2.20390558	2.68677592
6	-0.49039257	-1.37989450	-2.29762411	1	0.46360743	3.85790539	2.29337597
6	0.87240750	-1.68579447	-2.13022399	1	1.91450739	3.16000557	1.60587585
1	1.69970751	-1.08839452	-2.49352407	1	2.33540750	0.64620554	1.38117588
1	1.87970757	-3.43289447	-1.13722408	6	4.27550745	1.98260546	-0.25602409
1	-0.61059254	-4.28099442	-0.63662410	1	4.16420746	2.48270559	0.70957589
1	-2.33329248	-2.48039436	-1.64752412	1	4.52180719	2.73870564	-1.00342417
1	-0.89529258	-0.50919449	-2.79972410	1	5.11060715	1.28270555	-0.18182409

I-5pp-HH

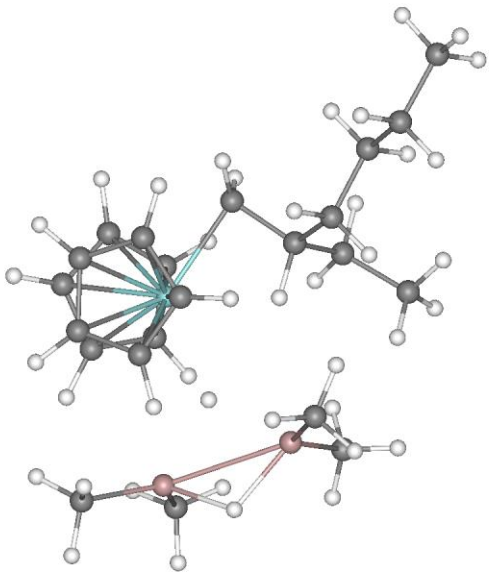

Zero-point vibrational energy	1504335.1 (Joules/Mol)
	359.54471 (Kcal/Mol)
Zero-point correction=	0.572971 (Hartree/Particle)
Thermal correction to Energy=	0.606750
Thermal correction to Enthalpy=	0.607694
Thermal correction to Gibbs Free Energy=	0.509438
Sum of electronic and zero-point Energies=	-4887.514400
Sum of electronic and thermal Energies=	-4887.480621
Sum of electronic and thermal Enthalpies=	-4887.479677
Sum of electronic and thermal Free Energies=	-4887.577933

cartesian

6	-1.21695745	-1.47740149	0.91270459	1	0.98534250	2.80379844	1.73880458
6	-1.50255752	-0.04400142	0.50470459	1	2.18404245	3.90679860	1.05910456
6	-1.60245740	0.92679864	1.69820464	1	0.46604249	4.19559860	0.79550457
1	-1.87255752	-2.19280148	0.41990456	13	3.72934246	1.08599854	-0.53059542
1	-1.28355753	-1.62500143	1.98990452	6	4.42994261	1.73369861	1.16300464
1	-2.34185767	0.53219861	2.40310454	6	4.65934277	-0.05600142	-1.80099547
1	-0.64405751	0.92969853	2.23450446	1	4.91494274	0.94629854	1.75020456
6	-0.26105753	-2.58690143	-1.68319547	1	5.20954275	2.47499847	0.94560456
6	0.97804248	-3.21370149	-1.40309536	1	3.70034242	2.23429847	1.80600452
6	2.02204251	-2.34000158	-1.82759547	1	5.01174259	-0.99020141	-1.35139537
6	1.42064250	-1.17490149	-2.34929562	1	4.12834263	-0.30340141	-2.72269559
6	0.01064250	-1.31410146	-2.24239564	1	5.56374264	0.48799860	-2.10389543
1	-0.72005749	-0.58890140	-2.58059549	1	1.86894250	0.66079861	-0.26509544
1	-1.23845744	-3.01790142	-1.51899540	6	-2.69365764	0.05859858	-0.46769544
1	1.10334253	-4.20760155	-0.99279541	1	-2.73055768	1.05459857	-0.92469543
1	3.08444238	-2.54580140	-1.78109539	6	-4.03895760	-0.23420142	0.20340456
1	1.93604255	-0.32370141	-2.77579546	1	-2.54755759	-0.65890145	-1.28459537
6	1.29804254	-2.74460149	2.15430450	1	0.47924250	1.56559849	-3.10079551
6	2.46194243	-2.83810139	1.35640454	1	-0.00705752	3.22089863	-2.77089548
6	3.09624243	-1.56930137	1.34430456	1	-0.89665747	1.89969850	-2.03489566
6	2.33954239	-0.70000142	2.18560457	6	-5.19615746	-0.21220142	-0.79679543
6	1.23684251	-1.42240143	2.67700458	1	-4.23615742	0.50279856	0.99110460
1	0.47534248	-1.03840137	3.34340453	1	-4.00235748	-1.21250141	0.69880456
1	0.59374249	-3.54220152	2.35490441	1	-5.01575756	-0.96100140	-1.57689548
1	2.81394243	-3.72530150	0.84580457	1	-5.22035742	0.76089853	-1.30089545
1	4.04634237	-1.35250139	0.87010455	6	-1.97675741	2.35429859	1.29930460
1	2.57524252	0.33149856	2.41780448	1	-1.81685746	3.04899859	2.12710452
40	0.91364253	-1.27110147	0.14210457	1	-3.02625751	2.42549849	1.00310457
6	0.10954249	2.23059845	-2.31439543	1	-1.37665749	2.70479846	0.45210454
1	-0.62585747	0.37999856	-0.10909544	6	-6.54445744	-0.47880143	-0.12969545
13	1.30274248	2.37329841	-0.78549540	1	-7.35795736	-0.45960140	-0.85809541
6	1.22814250	3.41209841	0.85950458	1	-6.75955725	0.27599859	0.63210458
1	2.93014240	2.36219859	-1.35299540	1	-6.55435753	-1.45790148	0.35790455

I-5pp-HHa

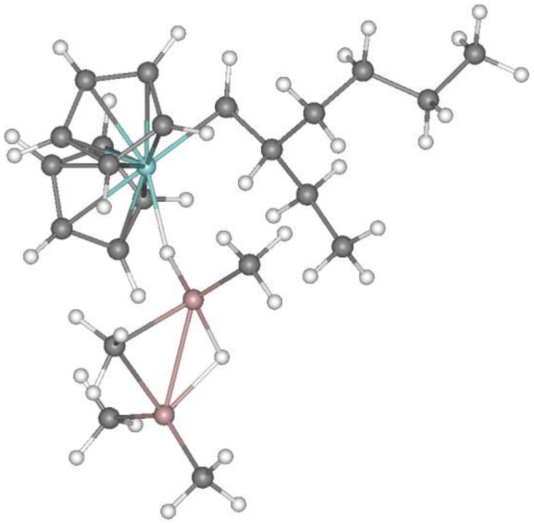

Zero-point vibrational energy	1501137.0 (Joules/Mol)
	358.78035 (Kcal/Mol)
Zero-point correction=	0.571753 (Hartree/Particle)
Thermal correction to Energy=	0.605633
Thermal correction to Enthalpy=	0.606577
Thermal correction to Gibbs Free Energy=	0.506095
Sum of electronic and zero-point Energies=	-4887.515912
Sum of electronic and thermal Energies=	-4887.482032
Sum of electronic and thermal Enthalpies=	-4887.481088
Sum of electronic and thermal Free Energies=	-4887.581570

cartesian

6	-2.05574393	-1.42942882	0.60655910	1	4.30655622	0.68457127	2.83125901
6	-1.80434394	0.03227127	0.26815909	1	4.33555603	-0.61192876	1.63085914
6	-1.67434382	0.92867124	1.51145911	1	5.78475618	0.33117127	1.95425904
1	-2.86164379	-1.87072873	0.02185909	13	2.04645610	0.97517121	-0.84864092
1	-2.26054382	-1.58302879	1.66655910	6	3.94185615	0.36247122	-1.37234092
1	-2.65474391	0.98787129	1.99655914	6	0.98455614	1.84327126	-2.21894097
1	-1.01204395	0.43707126	2.23395920	1	3.87225604	0.60947120	-2.44024086
6	-1.20344377	-2.55502868	-2.04984093	1	5.00645590	0.57207119	-1.17824090
6	-0.36484385	-3.63442874	-1.67474091	1	3.88265610	-0.72762871	-1.26574087
6	0.98535615	-3.24022865	-1.89524090	1	1.00145614	1.28547120	-3.16234088
6	0.97635615	-1.91462874	-2.37654090	1	-0.06384385	1.96317124	-1.92364097
6	-0.37774384	-1.47992873	-2.45724082	1	1.37165618	2.84467125	-2.43744087
1	-0.71454382	-0.51382875	-2.81574082	1	1.39655614	-0.30992875	-0.07454091
1	-2.28414392	-2.56272864	-2.03904080	6	-2.77804399	0.56427121	-0.80474091
1	-0.69854385	-4.60572863	-1.33094096	1	4.47835588	3.70907116	-1.07014096
1	1.86375618	-3.85172868	-1.72964096	1	6.03455591	3.26327133	-0.37044090
1	1.84835625	-1.34882879	-2.68504095	1	4.85685587	4.14197111	0.59385908
6	-0.08314383	-3.41642880	2.01245904	1	-2.75544381	-0.13832876	-1.64524090
6	1.02475619	-3.84922886	1.24625909	1	-2.42274380	1.52317131	-1.20164096
6	2.01685619	-2.83352876	1.28665912	6	-1.13324380	2.32587123	1.21625912
6	1.53265619	-1.79102874	2.12555909	1	-1.05874395	2.91787124	2.13135910
6	0.23915616	-2.14512873	2.56085920	1	-1.76854372	2.87567115	0.51695907
1	-0.39594382	-1.56012869	3.21315908	1	-0.12714383	2.27277136	0.78045911
1	-1.00324392	-3.96352863	2.17445898	6	-4.23024368	0.71627128	-0.31334090
1	1.10335612	-4.79352856	0.72235906	1	-4.90514374	0.45437127	-1.13534093
1	2.98945618	-2.87412882	0.81085908	1	-4.43104410	-0.01332876	0.48185909
1	2.06325626	-0.88152874	2.38115907	6	-4.60794401	2.12087131	0.16745909
40	0.09565617	-1.86762869	0.02165909	1	-3.98954391	2.41957116	1.02045906
6	4.96415615	3.35157132	-0.15584092	1	-4.39874411	2.83997130	-0.63334090
1	-0.78944379	0.15957123	-0.24644092	6	-6.08224392	2.20727134	0.56155908
13	4.21425629	1.67647123	0.46385908	1	-6.34764385	3.21377134	0.89215910
6	4.69645596	0.39957124	1.84845912	1	-6.31024408	1.51487124	1.37725914
1	2.46195602	1.89117122	0.50725907	1	-6.72734404	1.95057130	-0.28354090

I-5pp-HCl

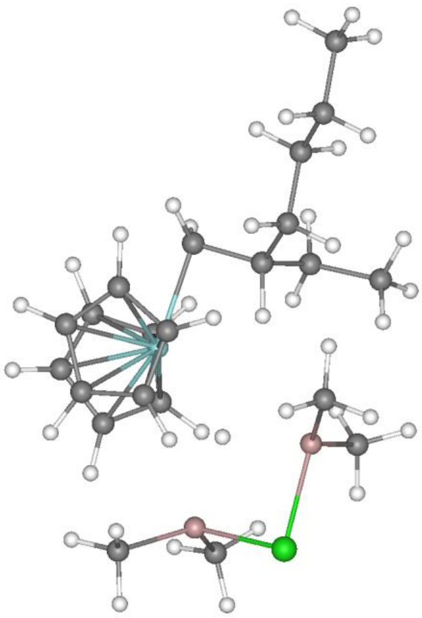

Zero-point vibrational energy	1487053.2 (Joules/Mol)
	355.41424 (Kcal/Mol)
Zero-point correction=	0.566389 (Hartree/Particle)
Thermal correction to Energy=	0.600565
Thermal correction to Enthalpy=	0.601509
Thermal correction to Gibbs Free Energy=	0.503588
Sum of electronic and zero-point Energies=	-5347.141470
Sum of electronic and thermal Energies=	-5347.107294
Sum of electronic and thermal Enthalpies=	-5347.106349
Sum of electronic and thermal Free Energies=	-5347.204270

cartesian

6	1.42939830	-1.40468633	-0.59354544	1	-1.13380170	2.20161343	-2.24894547
6	1.53229833	0.10281365	-0.46364546	1	-2.55640173	3.19261360	-1.90294528
6	1.62829828	0.82881367	-1.82014537	1	-0.94220167	3.84991360	-1.66674542
1	2.13389826	-1.93098640	0.04725456	13	-4.00380135	0.56021363	0.22435457
1	1.56889844	-1.74348640	-1.61924553	6	-4.63580132	0.81651366	-1.59254551
1	2.46089840	0.39301366	-2.38264537	6	-4.76270199	-0.57558632	1.60505462
1	0.72419834	0.61201364	-2.40284538	1	-4.87810135	-0.11838635	-2.10854530
6	0.48229837	-2.13888645	2.12415457	1	-5.57000160	1.38951361	-1.53314543
6	-0.63880163	-2.97798634	1.91265464	1	-3.96400166	1.39301360	-2.23494530
6	-1.81720161	-2.20838642	2.14435458	1	-4.91120148	-1.61558640	1.29825449
6	-1.41520166	-0.89788634	2.47945452	1	-4.24630165	-0.57128632	2.56815457
6	0.00449836	-0.84458637	2.44455457	1	-5.76570129	-0.17158635	1.79925466
1	0.60749829	0.02451365	2.67795467	1	-2.05380154	0.52271366	0.14515457
1	1.51789832	-2.44358635	2.07465458	6	2.63049841	0.52861363	0.52965456
1	-0.60150164	-4.03388643	1.67615461	1	2.54379845	1.59641361	0.75995457
1	-2.83600163	-2.57248640	2.09605455	6	4.04579830	0.24801365	0.01525456
1	-2.06790161	-0.07098635	2.73335457	1	2.48759842	-0.01088635	1.47405457
6	-0.80650163	-3.14258623	-1.69484544	1	-0.71730167	2.05461359	2.69845462
6	-1.98360169	-3.28568649	-0.92584544	1	-0.36390167	3.64921379	2.06285453
6	-2.79570174	-2.14138651	-1.13924551	1	0.67189837	2.30121374	1.62065458
6	-2.13000154	-1.30938637	-2.08754539	6	5.11229801	0.62481368	1.04535460
6	-0.90890169	-1.92308640	-2.42134547	1	4.23069859	0.80851364	-0.90904540
1	-0.17580166	-1.53608632	-3.11724544	1	4.14709854	-0.81338632	-0.24294543
1	0.01459831	-3.84668636	-1.74484539	1	4.93039799	0.07171366	1.97425461
1	-2.23310161	-4.12728643	-0.29254544	1	5.01439857	1.68781364	1.29385448
1	-3.79290175	-2.00658631	-0.73844546	6	1.81799841	2.34101343	-1.70094538
1	-2.49880171	-0.37368634	-2.48964548	1	1.65449834	2.83281374	-2.66254544
40	-0.75110167	-1.32898641	0.06295456	1	2.82739830	2.59381342	-1.36644554
6	-0.36910164	2.58241367	1.80445457	1	1.11779833	2.77861357	-0.98084539
1	0.56769836	0.52961367	0.00345457	6	6.52779865	0.34071365	0.54615456
13	-1.56040168	2.34151363	0.29095456	1	7.27439880	0.61751366	1.29375458
6	-1.54840159	2.94401360	-1.55654550	1	6.74329853	0.90471363	-0.36584544
17	-3.67230177	2.62261343	1.15925455	1	6.65819836	-0.72158635	0.32045457

I-5pp-HCla

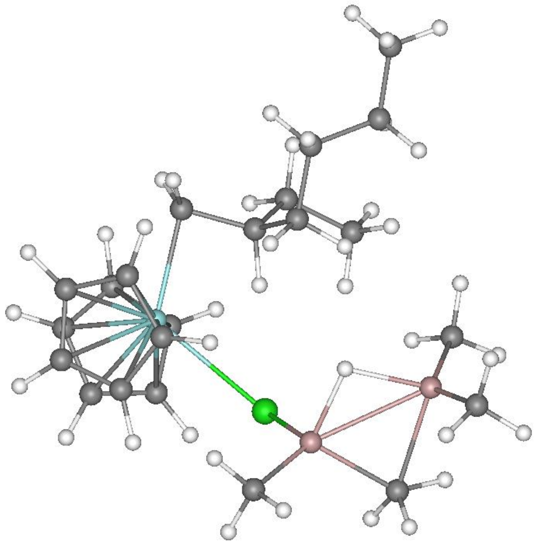

Zero-point vibrational energy	1487800.2 (Joules/Mol)
	355.59278 (Kcal/Mol)
Zero-point correction=	0.566673 (Hartree/Particle)
Thermal correction to Energy=	0.602626
Thermal correction to Enthalpy=	0.603570
Thermal correction to Gibbs Free Energy=	0.496919
Sum of electronic and zero-point Energies=	-5347.142933
Sum of electronic and thermal Energies=	-5347.106980
Sum of electronic and thermal Enthalpies=	-5347.106036
Sum of electronic and thermal Free Energies=	-5347.212687

cartesian

6	-2.11511064	1.61048484	-0.53378487	1	2.28438950	0.03358489	1.93131518
6	-0.81801057	1.56768489	0.26481512	1	3.98918962	0.00128490	2.38671517
6	-0.77941060	2.62198496	1.38481510	1	3.46998954	0.99548489	1.03571510
1	-1.94951057	1.84188485	-1.58438492	13	1.44598937	-2.91381502	-0.01798489
1	-2.82841039	2.32768488	-0.13088490	6	2.88338947	-3.33631516	1.36961508
1	-0.66151059	3.60718489	0.92041516	6	0.94468939	-4.38281536	-1.17188489
1	-1.76021063	2.64228487	1.87331510	1	3.45828962	-4.19781494	1.01551509
6	-3.41551042	-0.86011511	-2.35308480	1	3.53588963	-2.79731512	2.07411504
6	-3.48061037	-2.13741517	-1.73478484	1	2.12848949	-3.72121501	2.07321525
6	-2.16291046	-2.60541511	-1.54378486	1	0.35268942	-5.12661505	-0.62728488
6	-1.27411056	-1.62741518	-2.08668494	1	0.36778942	-4.08341503	-2.05178475
6	-2.05051064	-0.57011509	-2.60248494	1	1.84068930	-4.89641523	-1.53718483
1	-1.66991055	0.31198490	-3.09858489	17	-0.19321057	-1.94431520	1.14001513
1	-4.25841045	-0.23941511	-2.63148475	1	5.52368975	-1.34841514	-1.35498488
1	-4.38581038	-2.65701509	-1.44758487	1	4.87948942	-2.98711514	-1.28698492
1	-1.88071060	-3.54551506	-1.08378482	1	5.95208931	-2.40271521	-0.01608489
1	-0.19331059	-1.69301510	-2.14588475	6	0.44178942	1.53748488	-0.62738484
6	-4.71081066	-0.61391509	0.79731512	1	1.31078935	1.24518490	-0.02408489
6	-4.08381081	-1.77041507	1.35121512	1	0.31268945	0.73678488	-1.36778486
6	-3.10941052	-1.33461511	2.27081513	6	0.30738941	2.37778497	2.42891502
6	-3.11251044	0.08578488	2.28291512	1	0.26378945	3.12988496	3.22021508
6	-4.12511063	0.52918488	1.39411509	1	1.31028938	2.41418505	1.99431515
1	-4.41451073	1.55428481	1.21301508	1	0.18498942	1.39318490	2.89721513
1	-5.52701044	-0.61001509	0.08551511	6	0.73658943	2.86238503	-1.35608482
1	-4.32221079	-2.80151510	1.12271512	1	1.10448945	2.64328504	-2.36458492
1	-2.45941043	-1.97261500	2.85551524	1	-0.19481057	3.42698503	-1.49418485
1	-2.48371053	0.71238488	2.90431523	6	1.77318943	3.75198507	-0.66248488
40	-2.38491058	-0.59441513	-0.05758489	1	1.44238937	4.00888491	0.34941509
6	5.12878942	-2.11201501	-0.67768484	1	2.70658946	3.18678498	-0.54738486
1	-0.74161059	0.60208493	0.85681516	6	2.04388952	5.03378487	-1.44878483
13	3.60718966	-1.46691513	0.33411512	1	2.79758954	5.65148497	-0.95558488
6	3.30498958	0.03228489	1.53091514	1	1.13268936	5.63118505	-1.54658484
1	2.17718959	-1.56471515	-0.73078489	1	2.40298963	4.80608511	-2.45658493

I-5pp-HClb

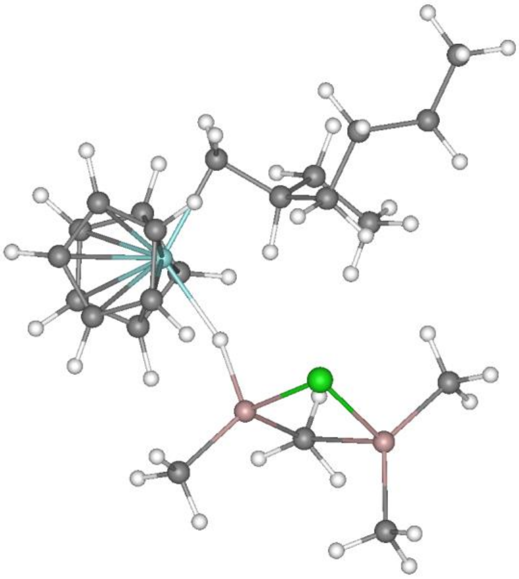

Zero-point vibrational energy	1484528.2 (Joules/Mol)
	354.81076 (Kcal/Mol)
Zero-point correction=	0.565427 (Hartree/Particle)
Thermal correction to Energy=	0.601443
Thermal correction to Enthalpy=	0.602387
Thermal correction to Gibbs Free Energy=	0.496505
Sum of electronic and zero-point Energies=	-5347.145259
Sum of electronic and thermal Energies=	-5347.109243
Sum of electronic and thermal Enthalpies=	-5347.108299
Sum of electronic and thermal Free Energies=	-5347.214181

cartesian

6	2.07384253	1.58858633	0.29006666	1	-3.22825766	0.57238626	-0.97253335
6	0.63094246	1.55538630	-0.18873335	1	-4.49635744	-0.19141369	-1.93543339
6	0.43984249	2.30558634	-1.51743340	1	-4.85885763	0.41268632	-0.32343334
1	2.16844249	1.95308626	1.31326663	13	-0.90535748	-2.61231351	0.21356666
1	2.72684240	2.17348647	-0.35663337	6	-1.95275748	-2.45331359	-1.53793335
1	0.56444252	3.37538648	-1.31863332	6	-0.41705751	-4.36781359	0.86356664
1	1.25734246	2.03408647	-2.19453335	1	-1.27205753	-3.22501349	-1.93763328
6	3.44304228	-0.41241369	2.27226663	1	-2.89695740	-2.84111357	-1.95053339
6	3.69924259	-1.71421373	1.77826667	1	-1.71785748	-1.53041375	-2.07773328
6	2.49804258	-2.46661353	1.85996664	1	0.35334247	-4.83631372	0.23976666
6	1.50914252	-1.63151371	2.45156670	1	-0.03535751	-4.34251356	1.88966668
6	2.08734250	-0.36731368	2.69336653	1	-1.27815747	-5.04501343	0.86566663
1	1.59174252	0.48428634	3.14156675	1	0.29604250	-1.56941366	-0.12383334
1	4.15674257	0.39918634	2.33766675	1	-5.29415751	-3.20841360	1.16976666
1	4.64954233	-2.07521367	1.40596664	1	-3.97565746	-4.23431349	0.62136668
1	2.37304258	-3.50521350	1.57686663	1	-5.25145769	-3.75781369	-0.49943334
1	0.49874249	-1.92061377	2.71766663	6	-0.38965750	1.92268622	0.90976667
6	4.07924271	-0.90161371	-1.34273338	1	-1.40175748	1.65148628	0.58266664
6	3.44294262	-2.17641354	-1.36183333	1	-0.18445751	1.28268623	1.77676666
6	2.19774246	-2.03051376	-2.00433326	6	-0.89765751	2.03848648	-2.20153332
6	2.05264235	-0.66611367	-2.38623333	1	-0.95135748	2.54228640	-3.16953325
6	3.23104239	0.02058631	-2.00403333	1	-1.74145758	2.38928652	-1.60143340
1	3.45554256	1.06078625	-2.19263339	1	-1.03875756	0.96398628	-2.37953329
1	5.06154251	-0.68461370	-0.94093335	6	-0.36735749	3.39988637	1.34186661
1	3.84634256	-3.09721351	-0.95943332	1	-0.57205749	3.45418644	2.41686654
1	1.48544252	-2.82451367	-2.19253325	1	0.64014250	3.81418657	1.20746660
1	1.21084249	-0.24251369	-2.92213345	6	-1.38935757	4.29248619	0.62936664
40	2.09544253	-0.68051368	0.14956667	1	-1.19445753	4.31968641	-0.44793338
6	-4.62415743	-3.40251350	0.32546666	1	-2.38835764	3.85678625	0.75236666
1	0.31404248	0.48888633	-0.46293336	6	-1.38095748	5.71878624	1.17786670
13	-3.63495731	-1.81101370	-0.15513335	1	-1.61435747	5.72728634	2.24646664
6	-4.09455776	-0.09571368	-0.92073333	1	-2.11715746	6.34498644	0.66906667
17	-2.16865730	-1.40461373	1.64506662	1	-0.39885753	6.18258619	1.04756665

TS-56pp-HH

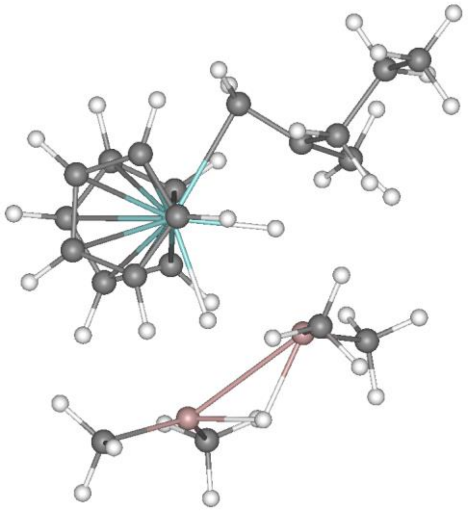

Zero-point vibrational energy	1342398.4 (Joules/Mol)
	320.84091 (Kcal/Mol)
Zero-point correction=	0.511293 (Hartree/Particle)
Thermal correction to Energy=	0.542234
Thermal correction to Enthalpy=	0.543178
Thermal correction to Gibbs Free Energy=	0.452111
Sum of electronic and zero-point Energies=	-4808.966751
Sum of electronic and thermal Energies=	-4808.935810
Sum of electronic and thermal Enthalpies=	-4808.934865
Sum of electronic and thermal Free Energies=	-4809.025933

cartesian

6	-1.74107826	-1.75923347	0.84960830	1	-0.62087822	0.38606659	0.27200833
6	-2.01357818	-0.38463342	0.88860834	13	0.37242177	2.13566661	-0.22199169
6	-1.97137821	0.34246656	2.21760845	6	0.24662177	2.89706659	1.57370842
1	-2.23767805	-2.35513353	0.09150831	1	1.99622178	2.68996644	-0.73379171
1	-1.55677831	-2.26933336	1.78920841	1	0.42772177	2.17256665	2.37520838
1	-2.01607823	1.42456651	2.08540845	1	0.98352182	3.69946647	1.69180834
1	-2.84777808	0.03116658	2.79590845	1	-0.73507822	3.34666657	1.75990832
1	-1.08257830	0.09996659	2.80170846	13	3.12902188	1.52146661	-0.34679168
6	-0.69897819	-2.15683341	-2.05169153	6	3.82472181	1.80476665	1.44870842
6	0.51962179	-2.85823345	-1.90129161	6	4.07222176	0.80366659	-1.89429164
6	1.58262169	-1.94773340	-2.16949153	1	4.30802202	0.92626655	1.88930833
6	1.01292181	-0.69123340	-2.47289157	1	4.59942198	2.58026648	1.39280832
6	-0.39927825	-0.80843341	-2.37389159	1	3.07322192	2.17316651	2.15450835
1	-1.11347830	-0.02113342	-2.57729173	1	4.08452177	-0.29113340	-1.93069160
1	-1.68797827	-2.58603334	-1.96829164	1	3.67962193	1.16176653	-2.85079169
1	0.62252176	-3.91183352	-1.67259157	1	5.12102175	1.11946654	-1.84899163
1	2.63902187	-2.18553329	-2.17139173	1	1.47752178	0.59076655	-0.19879168
1	1.55872178	0.20266658	-2.74689174	6	-2.95967817	0.20436659	-0.13809168
6	1.08932173	-3.03063345	1.60780835	1	-0.04477823	2.12536645	-2.77079153
6	2.24022174	-2.85653353	0.80510831	1	-0.53817821	3.63116646	-2.02379179
6	2.76182175	-1.56413341	1.05150843	1	-1.60107827	2.23466659	-1.93709159
6	1.95902169	-0.95423341	2.06420827	1	-2.75627804	1.27136660	-0.27589166
6	0.93882179	-1.85863340	2.40490842	6	-4.42167807	0.03016660	0.31120831
1	0.18222177	-1.70253348	3.16240835	1	-2.82527804	-0.29103342	-1.10539162
1	0.47112179	-3.91893339	1.65240836	6	-5.38857794	0.55556655	-0.74899167
1	2.64522195	-3.58163333	0.11070831	1	-4.58537817	0.56556654	1.25140834
1	3.66802192	-1.16003346	0.61440831	1	-4.61777830	-1.02903342	0.50870830
1	2.11642170	0.02516657	2.49990845	1	-6.42367792	0.44036660	-0.42209169
40	0.52162176	-1.19393337	-0.03679168	1	-5.27227831	0.01476657	-1.69229162
6	-0.55197823	2.54196644	-1.89359164	1	-5.21587801	1.61736655	-0.94559169

TS-56pp-HHa

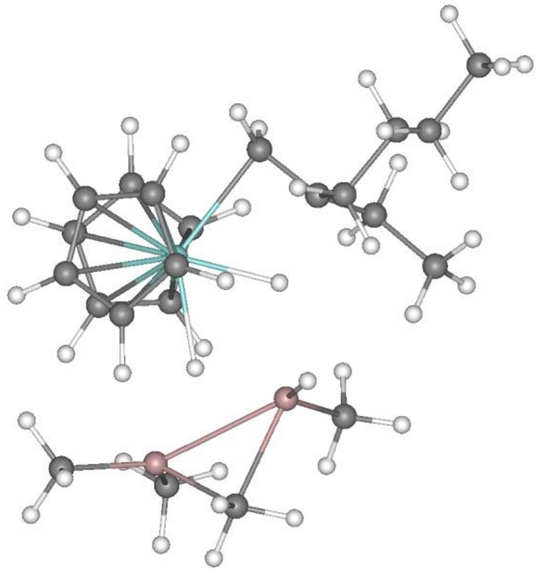

Zero-point vibrational energy	1490062.8 (Joules/Mol)
	356.13355 (Kcal/Mol)
Zero-point correction=	0.567535 (Hartree/Particle)
Thermal correction to Energy=	0.601113
Thermal correction to Enthalpy=	0.602057
Thermal correction to Gibbs Free Energy=	0.504705
Sum of electronic and zero-point Energies=	-4887.487091
Sum of electronic and thermal Energies=	-4887.453513
Sum of electronic and thermal Enthalpies=	-4887.452568
Sum of electronic and thermal Free Energies=	-4887.549921

cartesian

6	1.54489541	-1.59110904	-1.13544238	1	-0.78040463	2.40429091	-2.01424217
6	1.82769525	-0.25660905	-0.80924237	1	-2.03520465	3.39579082	-1.26644242
6	1.88669527	0.77179092	-1.93584239	1	-0.34640464	3.82549071	-1.07734239
1	2.00599527	-2.37420917	-0.54194236	1	-3.70000458	3.03069091	1.73715758
1	1.40969539	-1.83190894	-2.18494225	1	-2.45260477	2.37289095	2.78245783
1	2.68399525	0.43109095	-2.60814238	1	-2.11610460	3.55879092	1.52005768
1	0.96229541	0.72479099	-2.51834249	13	-3.57200480	1.08109093	0.72155762
6	0.55429536	-2.49940920	1.66225767	6	-4.39880466	1.57499099	-0.97464240
6	-0.56880462	-3.29330921	1.33205760	6	-4.44200468	-0.11240904	1.99765766
6	-1.73710465	-2.58010912	1.72745764	1	-4.86120462	0.72709101	-1.49194241
6	-1.32540464	-1.34900904	2.28435755	1	-5.22290468	2.26529098	-0.74724239
6	0.09099537	-1.28040910	2.21925783	1	-3.75920486	2.09549093	-1.69234240
1	0.69519532	-0.45780903	2.58105755	1	-4.34000492	-1.17470908	1.74955761
1	1.58809531	-2.78850913	1.53025758	1	-4.12310457	0.01239097	3.03725767
1	-0.54100466	-4.28680897	0.90125763	1	-5.51900482	0.09629095	1.97725761
1	-2.75700474	-2.93410921	1.64375758	1	-1.84300458	0.36599094	0.31425762
1	-1.97580469	-0.59320903	2.70425749	6	2.68109560	0.02769095	0.40705764
6	-1.14070463	-2.74110913	-2.22514248	1	2.48849535	1.03059101	0.79965764
6	-2.28010464	-2.88850904	-1.40324235	1	2.46029520	-0.68430901	1.20485759
6	-2.93990469	-1.63640904	-1.33024240	6	2.17549515	2.20739079	-1.50124240
6	-2.23280478	-0.72240901	-2.16794252	1	2.01139545	2.89579082	-2.33274221
6	-1.12730455	-1.39920902	-2.71054220	1	3.20969534	2.32779098	-1.16914237
1	-0.40320462	-0.97840905	-3.39684248	1	1.53029537	2.52169085	-0.67784238
1	-0.43620464	-3.52260900	-2.48214245	6	4.17199516	-0.11870904	0.04405763
1	-2.58670473	-3.79790902	-0.90184236	1	4.42319536	0.50109094	-0.82394236
1	-3.86620474	-1.44160914	-0.80194235	1	4.36869526	-1.15670896	-0.25024235
1	-2.49860477	0.31189096	-2.34974241	6	5.07479525	0.26779097	1.21655762
40	-0.73180461	-1.30460906	-0.18334237	1	4.88709545	1.31379104	1.48415768
1	0.08509538	1.93829083	1.73885763	1	4.80609512	-0.33120903	2.09425783
1	0.31509537	0.36459094	-0.19264238	6	6.55509520	0.07359096	0.89305764
13	-0.93570465	1.93179083	0.53755760	1	7.18389511	0.36059093	1.73835766
6	-1.03640461	2.97489095	-1.11384237	1	6.85189533	0.68089098	0.03335763
6	-2.66770458	2.63469100	1.74285758	1	6.77089548	-0.97170901	0.65485764

TS-56pp-HHb

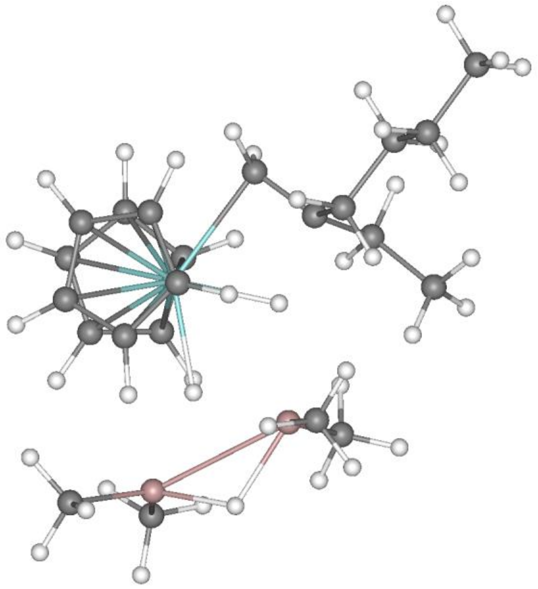

Zero-point vibrational energy	1490318.8 (Joules/Mol)
	356.19474 (Kcal/Mol)
Zero-point correction=	0.567632 (Hartree/Particle)
Thermal correction to Energy=	0.601580
Thermal correction to Enthalpy=	0.602525
Thermal correction to Gibbs Free Energy=	0.503519
Sum of electronic and zero-point Energies=	-4887.499169
Sum of electronic and thermal Energies=	-4887.465221
Sum of electronic and thermal Enthalpies=	-4887.464276
Sum of electronic and thermal Free Energies=	-4887.563282

cartesian

6	-1.28868484	-1.56283498	1.22032273	1	0.76431513	2.38606501	2.06942272
6	-1.66128492	-0.25923499	0.85172278	1	2.11291504	3.33686495	1.44252276
6	-1.74578500	0.80596495	1.94092274	1	0.46531516	3.88996506	1.21002269
1	-1.73518491	-2.39533496	0.68542278	13	3.70731497	1.26556504	-0.78457725
1	-1.10608482	-1.74563503	2.27442288	6	4.68231535	1.67406499	0.85092276
1	-2.52058482	0.45746499	2.63522291	6	4.43381548	0.29166502	-2.30787706
1	-0.81408483	0.81496501	2.51342273	1	5.12511539	0.79896498	1.33802271
6	-0.34968483	-2.50403500	-1.58027732	1	5.52681541	2.31556511	0.56592280
6	0.84251511	-3.20973492	-1.29557729	1	4.11821508	2.23076510	1.60392272
6	1.93631506	-2.42083502	-1.75387728	1	4.41511536	-0.79693502	-2.18517709
6	1.41111517	-1.23233497	-2.30887723	1	3.95261502	0.52806503	-3.26147723
6	-0.00168484	-1.26653504	-2.17927718	1	5.49021530	0.56606501	-2.41427708
1	-0.68268484	-0.50163502	-2.53177714	1	1.96931505	0.49356502	-0.35357726
1	-1.35228491	-2.86593509	-1.39737725	6	-2.60188484	-0.08503500	-0.32137725
1	0.90661514	-4.19623470	-0.85327721	1	0.40371513	1.70946503	-2.96637726
1	2.98241520	-2.69803500	-1.71067727	1	0.00091514	3.33536506	-2.44817710
1	1.98231506	-0.43773499	-2.76997709	1	-1.08098483	2.00426507	-2.05187726
6	1.43161511	-2.57813501	2.24582291	1	-2.50828481	0.91346502	-0.75657725
6	2.53831506	-2.74173498	1.38512266	1	-2.38188481	-0.81173497	-1.10657728
6	3.18651509	-1.48813498	1.25172269	6	-2.10058498	2.21126509	1.45942271
6	2.50931501	-0.55693495	2.09542274	1	-1.96178484	2.93586493	2.26422286
6	1.42771506	-1.22233498	2.69532275	1	-3.14158487	2.27266502	1.13202274
1	0.72871518	-0.78623497	3.39822292	1	-1.47628474	2.52716494	0.61932278
1	0.74471515	-3.35693502	2.55312276	6	-4.05608463	-0.30883500	0.13802275
1	2.83321500	-3.66253495	0.89812279	1	-4.30218458	0.35466498	0.97472274
1	4.09241533	-1.30683494	0.68432277	1	-4.16258478	-1.33373499	0.51312280
1	2.77651501	0.48246503	2.24312282	6	-5.04758453	-0.06803499	-1.00157726
40	0.93031514	-1.19613504	0.18492275	1	-4.94218445	0.96266496	-1.35867727
6	-0.02678487	2.28416491	-2.13787723	1	-4.79408455	-0.71773499	-1.84727728
1	-0.23308486	0.41526502	0.15892276	6	-6.49128485	-0.32063499	-0.57097721
13	1.00881517	2.06596494	-0.49947724	1	-7.18598461	-0.13733500	-1.39317727
6	1.09681511	2.99456501	1.22122276	1	-6.77358484	0.33436501	0.25802276
1	2.62001514	2.46736503	-1.18697727	1	-6.62758446	-1.35453498	-0.24157724

TS-56pp-HHc

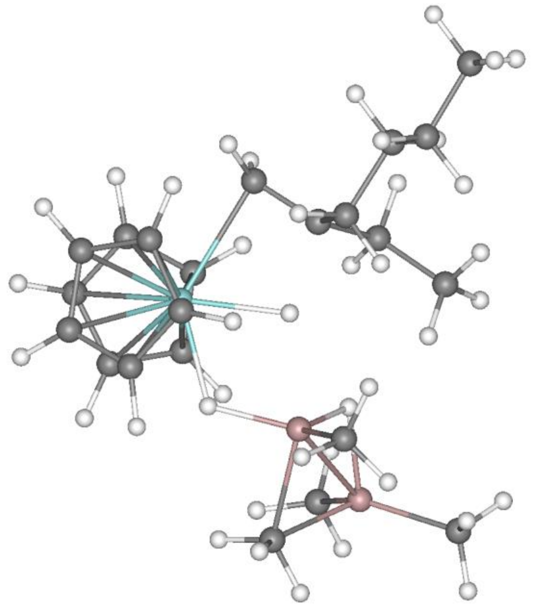

Zero-point vibrational energy	1493167.4 (Joules/Mol)
	356.87558 (Kcal/Mol)
Zero-point correction=	0.568717 (Hartree/Particle)
Thermal correction to Energy=	0.602666
Thermal correction to Enthalpy=	0.603610
Thermal correction to Gibbs Free Energy=	0.503292
Sum of electronic and zero-point Energies=	-4887.500889
Sum of electronic and thermal Energies=	-4887.466940
Sum of electronic and thermal Enthalpies=	-4887.465996
Sum of electronic and thermal Free Energies=	-4887.566314

cartesian

6	-1.55392265	-1.33569527	1.44403493	1	5.05907726	0.37280464	1.16663492
6	-1.45402265	-0.03129533	0.93573487	1	5.54317760	2.02970457	1.48813486
6	-0.86832273	1.06390464	1.82703495	1	4.22657728	1.32320464	2.40163469
1	-2.38182282	-1.95549536	1.11333489	13	1.54977727	0.69180465	-1.10266507
1	-1.23302269	-1.50619531	2.46693468	6	3.61747718	0.80100465	-1.66316509
1	-1.52052271	1.11780465	2.70723486	6	0.49587727	1.51830471	-2.51356530
1	0.11107728	0.74600470	2.19673491	1	4.14347744	-0.11429533	-1.37406504
6	-0.77252269	-3.91989517	-1.06266510	1	3.23287725	0.64280462	-2.68156528
6	0.44447726	-3.72349548	-1.77696514	1	4.40067720	1.54820478	-1.87556505
6	0.35447729	-2.49339533	-2.46356511	1	0.50987726	0.88850462	-3.41286516
6	-0.90752268	-1.90879536	-2.16386509	1	-0.55492270	1.68330467	-2.25796509
6	-1.61122274	-2.81199551	-1.32646513	1	0.91257727	2.48690462	-2.81206512
1	-2.62372279	-2.69079542	-0.96556515	1	1.72147727	-0.96949530	-0.97386515
1	-1.03092265	-4.78589535	-0.46516511	1	4.01567745	4.39410448	-0.86866516
1	1.28407729	-4.40629530	-1.80836511	1	2.80457711	4.53710461	0.39403489
1	1.10977733	-2.07609534	-3.11696529	1	2.33167720	4.02100468	-1.22186506
1	-1.28042269	-0.97169530	-2.56066513	6	-2.47352290	0.41330469	-0.09326513
6	0.87907732	-3.23349524	2.15823483	1	-2.69742274	-0.40089533	-0.78726512
6	1.67367733	-3.82869530	1.15093493	1	-2.09662271	1.25300467	-0.68376511
6	2.61917710	-2.87139511	0.70703489	6	-0.76892269	2.45080471	1.19213498
6	2.44537711	-1.69419539	1.49183488	1	-0.20502272	3.12190485	1.84403491
6	1.37427735	-1.91489530	2.37753487	1	-1.75502264	2.89480448	1.03473496
1	1.00507724	-1.21199536	3.11363482	1	-0.26062271	2.42900467	0.22393487
1	0.07547727	-3.71439552	2.70223475	6	-3.78022265	0.82690465	0.60933489
1	1.56397736	-4.83699560	0.77243483	1	-3.57922292	1.57660472	1.38353491
1	3.37357712	-3.02219534	-0.05516512	1	-4.20382261	-0.04329532	1.12473488
1	3.04097724	-0.79069531	1.42633486	6	-4.80022240	1.38960469	-0.38186511
40	0.37577727	-1.97609532	0.02263488	1	-4.37362242	2.27070475	-0.87486511
6	3.11327720	3.92520475	-0.46036512	1	-4.98582268	0.65060472	-1.16986513
1	-0.08652273	-0.05269533	-0.07856513	6	-6.11692238	1.76400459	0.29623488
13	3.44077730	2.08190489	0.06843487	1	-6.83112240	2.16730452	-0.42446512
6	4.67237759	1.37000465	1.40183496	1	-5.95842266	2.52080488	1.06963491
1	1.82007730	1.42790473	0.39403489	1	-6.57572269	0.89190471	0.77063483

TS-56pp-HHd

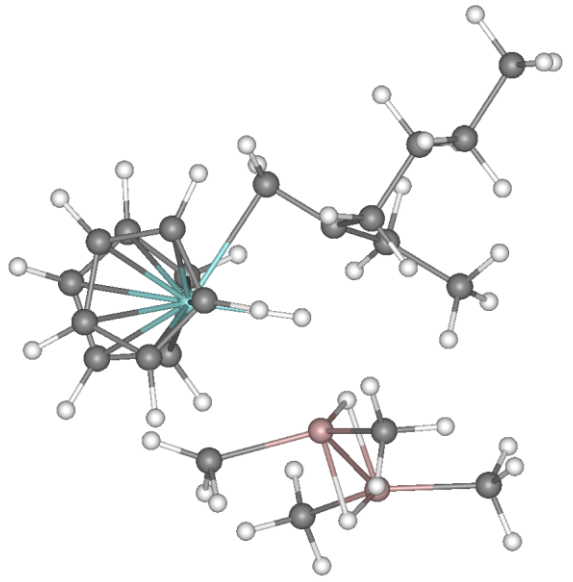

Zero-point vibrational energy	1492812.0 (Joules/Mol)
	356.79063 (Kcal/Mol)
Zero-point correction=	0.568582 (Hartree/Particle)
Thermal correction to Energy=	0.602395
Thermal correction to Enthalpy=	0.603339
Thermal correction to Gibbs Free Energy=	0.503148
Sum of electronic and zero-point Energies=	-4887.479245
Sum of electronic and thermal Energies=	-4887.445433
Sum of electronic and thermal Enthalpies=	-4887.444488
Sum of electronic and thermal Free Energies=	-4887.544679

cartesian

6	-0.92170441	-1.43354702	1.53357267	1	4.74879551	1.92475295	0.12687272
6	-1.23110437	-0.21644697	0.89787275	1	4.90139532	3.62555289	0.52557272
6	-0.95230436	1.07835305	1.65547264	1	4.15349579	2.53815293	1.67717266
1	-1.57390440	-2.28074694	1.35007262	13	1.22469556	1.14835298	-1.34702730
1	-0.54520440	-1.36844695	2.54937267	6	2.65589547	-0.24254698	-1.66422737
1	-1.54380441	1.00725305	2.57707262	6	-0.20250443	1.52605295	-2.62352729
1	0.09389558	1.09595299	1.97307265	1	2.89059544	-1.30464697	-1.60252738
6	0.38679558	-4.07064676	-0.59822726	1	2.65229559	-0.06394696	-2.75152731
6	1.42389560	-3.68354702	-1.49822736	1	3.54149556	0.26565304	-1.26592731
6	0.90909553	-2.67324710	-2.33902740	1	0.23989558	1.66695309	-3.61712742
6	-0.42510441	-2.40014696	-1.94802737	1	-0.95110440	0.73245299	-2.71322727
6	-0.75550437	-3.29434705	-0.89652729	1	-0.73770440	2.45465302	-2.39592743
1	-1.72400439	-3.39184690	-0.42602727	1	2.35959554	2.47395301	-1.74002731
1	0.44469559	-4.85764694	0.14347270	1	1.95669556	5.66395330	-0.48162729
1	2.41479540	-4.11764669	-1.55532730	1	1.18289554	5.05775309	0.97467273
1	1.43949556	-2.18174696	-3.14382744	1	0.47539556	4.72115326	-0.60972726
1	-1.08710444	-1.68894696	-2.42552733	6	-2.36810446	-0.18214697	-0.09792729
6	1.80789566	-2.52514696	2.32777262	1	-2.38540459	-1.09724700	-0.69562727
6	2.69619560	-3.11674690	1.40217268	1	-2.26080441	0.66205299	-0.78522730
6	3.43179560	-2.08394694	0.77297270	6	-1.30830443	2.37805295	0.93627274
6	3.03659558	-0.84594697	1.35967267	1	-0.94330442	3.23325300	1.51007271
6	2.03939557	-1.11704695	2.31237268	1	-2.38870454	2.49475288	0.81907272
1	1.54609561	-0.38394699	2.93777275	1	-0.86350441	2.43845296	-0.05892729
1	1.11539555	-3.05414701	2.97027278	6	-3.71090460	-0.07034698	0.65197271
1	2.78649545	-4.17604685	1.19717264	1	-3.69430447	0.77835298	1.34517264
1	4.20629549	-2.21774697	0.02817271	1	-3.86020470	-0.96774697	1.26427269
1	3.43159556	0.13425305	1.11817265	6	-4.88110447	0.09735304	-0.31892729
40	1.04049563	-1.74334705	0.04717271	1	-4.72010469	0.99855304	-0.92152727
6	1.42109561	4.80475330	-0.06402729	1	-4.89680433	-0.74684697	-1.01772738
1	0.05789557	-0.02894697	-0.21202728	6	-6.22220469	0.19415304	0.40627271
13	2.49389553	3.18435311	-0.18492728	1	-7.04360437	0.31955305	-0.30212727
6	4.23159552	2.76245308	0.60727274	1	-6.23670435	1.04765296	1.08987272
1	1.41609561	1.82665300	0.22207272	1	-6.41950464	-0.70814693	0.99187273

TS-56pp-HCl

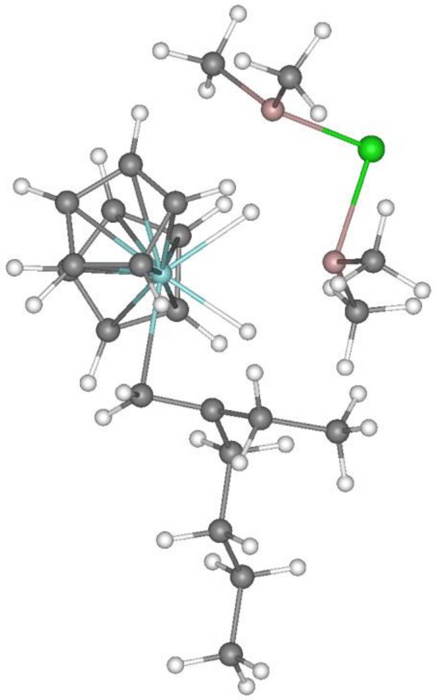

Zero-point vibrational energy	1475391.0 (Joules/Mol)
	352.62692 (Kcal/Mol)
Zero-point correction=	0.561947 (Hartree/Particle)
Thermal correction to Energy=	0.596957
Thermal correction to Enthalpy=	0.597901
Thermal correction to Gibbs Free Energy=	0.497472
Sum of electronic and zero-point Energies=	-5347.125687
Sum of electronic and thermal Energies=	-5347.090678
Sum of electronic and thermal Enthalpies=	-5347.089734
Sum of electronic and thermal Free Energies=	-5347.190162

cartesian

6	1.47510302	-1.62942100	-0.75099707	1	-0.88329709	1.89997900	-2.49069715
6	1.72300303	-0.24572109	-0.69079703	1	-2.33939695	2.80777884	-2.06329703
6	1.76870286	0.54377890	-1.99629700	1	-0.76599705	3.57097888	-1.95429695
1	1.96650302	-2.26682115	-0.02209703	13	-4.00819731	0.82197887	0.33890295
1	1.36880291	-2.06812119	-1.73759699	6	-4.75349712	0.93767887	-1.44969702
1	2.59460306	0.11137891	-2.57479715	6	-4.75649738	-0.20262109	1.81150305
1	0.86190295	0.34317893	-2.57329702	1	-5.07009697	-0.02382109	-1.86669695
6	0.43290293	-2.00072122	2.15800285	1	-5.66219711	1.54867899	-1.36369705
6	-0.72419709	-2.80372119	2.02450299	1	-4.12369728	1.44017899	-2.18839717
6	-1.86169708	-1.97182107	2.23540282	1	-4.99599743	-1.23892105	1.55100298
6	-1.39609706	-0.66222107	2.49440289	1	-4.18699694	-0.20542109	2.74390292
6	0.02040291	-0.66932112	2.42080283	1	-5.71729708	0.27507889	2.04770303
1	0.65970290	0.18707891	2.59400296	1	-2.03659725	0.45637888	0.17250296
1	1.45370281	-2.35432124	2.10340285	6	2.60720301	0.26307893	0.42700297
1	-0.73669708	-3.87142110	1.84460294	1	2.42710304	1.32227898	0.62490296
1	-2.89679718	-2.29032111	2.22990298	6	4.08710289	0.06207891	0.04420296
1	-2.00599718	0.20227890	2.72790289	1	2.41600275	-0.28902107	1.35050297
6	-1.04429710	-3.10182118	-1.61409700	1	-0.47259706	2.29907894	2.55390286
6	-2.18739700	-3.18392110	-0.79209703	1	-0.22329709	3.79407883	1.68050301
6	-2.96049690	-2.01142120	-0.98859710	1	0.98090291	2.51437879	1.56700301
6	-2.31619692	-1.22872102	-1.99349701	6	5.02740288	0.62787890	1.10950303
6	-1.13629699	-1.89122105	-2.36729717	1	4.30210304	0.54377890	-0.91639704
1	-0.43349707	-1.55102110	-3.11769700	1	4.28270292	-1.00802112	-0.09399704
1	-0.26649708	-3.85062122	-1.69689703	1	4.81360292	0.15267892	2.07370305
1	-2.43129706	-3.99702120	-0.12019704	1	4.82580280	1.69747901	1.23710299
1	-3.92689705	-1.82142103	-0.53739709	6	1.99290287	2.04727888	-1.84949696
1	-2.66889715	-0.28712109	-2.39559722	1	1.84240282	2.54767895	-2.80819702
40	-0.81829709	-1.23582101	0.08590297	1	3.00800276	2.27227879	-1.51279700
6	-0.09419706	2.70667887	1.60980296	1	1.30310297	2.49797893	-1.13109696
1	0.22990292	0.43067890	-0.20979702	6	6.49630260	0.41927892	0.74490297
13	-1.13009715	2.08137894	0.08320297	1	6.72660303	-0.64442110	0.63690293
6	-1.29689705	2.62737894	-1.78259695	1	7.15580273	0.82817888	1.51300299
17	-3.26029730	2.81217885	1.01080298	1	6.73840284	0.91217893	-0.20089704

S1.5. Mononuclear O[SiMe$_2$(η^5-C$_5$H$_4$)]$_2$Zr-based catalytic species

I-2pβ

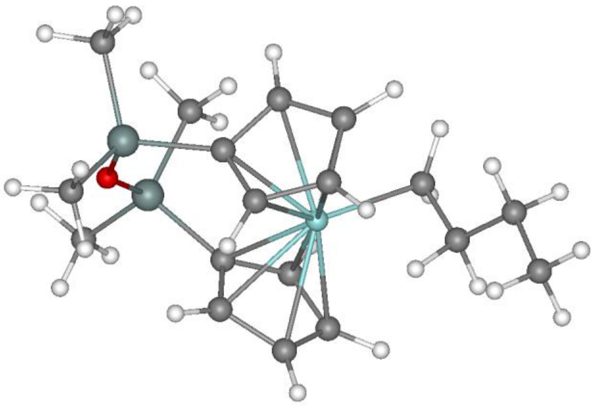

Zero-point vibrational energy	1122521.6 (Joules/Mol)
	268.28910 (Kcal/Mol)
Zero-point correction=	0.427546 (Hartree/Particle)
Thermal correction to Energy=	0.454764
Thermal correction to Enthalpy=	0.455709
Thermal correction to Gibbs Free Energy=	0.370191
Sum of electronic and zero-point Energies=	-4897.535341
Sum of electronic and thermal Energies=	-4897.508123
Sum of electronic and thermal Enthalpies=	-4897.507179
Sum of electronic and thermal Free Energies=	-4897.592697

cartesian

6	-4.48997879	0.32487062	-0.62186080	1	0.50292146	1.64727056	-2.12186074
6	-2.33657861	-0.86302936	-1.44236076	14	2.68202138	1.57767057	0.30443922
6	-3.36577868	-0.67742932	-0.34436077	1	0.04122148	1.94467056	2.19713926
1	-2.26407862	-1.89352942	-1.78826082	40	-0.82027847	-0.25172937	0.09173922
1	-2.49047852	-0.18282938	-2.28036070	6	3.11052132	2.30717063	1.95643926
1	-2.90247869	-0.28572938	0.64223921	6	3.43422127	2.46357059	-1.14536083
1	-4.05827856	1.26887059	-0.97006083	8	3.16282129	-0.00712937	0.28703922
1	-5.08067846	-0.07072937	-1.45346081	1	4.52562141	2.46877074	-1.06596076
1	-3.77687860	-1.63152945	-0.00316078	1	3.09822130	3.50287080	-1.20706081
6	-0.03857851	-2.63462925	0.03343922	1	3.18222141	1.97207057	-2.09116077
6	1.04332149	-1.80992937	0.48473921	1	4.19222116	2.27417064	2.11713934
6	0.65602148	-1.32122946	1.77413917	1	2.64752150	1.75407064	2.78013921
6	-0.64027852	-1.79422939	2.08153939	1	2.79482150	3.35207081	2.03163934
6	-1.06817853	-2.61452937	0.99663919	6	3.94552135	-2.76452923	0.17213923
1	-2.01667857	-3.13422918	0.92883921	6	2.45262146	-1.37402940	-2.17276073
1	-0.07547851	-3.16972923	-0.90926081	1	3.38432145	-1.11552942	-2.68656063
14	2.71532130	-1.47432935	-0.32586077	1	1.71202147	-0.61142933	-2.44666076
1	1.25972152	-0.67392933	2.40163922	1	2.11072135	-2.32772923	-2.58876061
1	-1.19787848	-1.59862936	2.99093938	1	4.92282152	-2.56382918	-0.27626076
6	-1.28667855	2.03967071	-0.86736083	1	3.62802148	-3.76112938	-0.14816079
6	0.07452149	1.76067054	-1.13026083	1	4.07222128	-2.78442931	1.25783920
6	0.79492152	1.66897058	0.10193922	6	-5.38027859	0.56607068	0.59413922
6	-0.16957851	1.91157055	1.13313925	1	-6.19067860	1.25747061	0.35643923
6	-1.44027853	2.13707066	0.54333919	1	-4.81057882	0.99567062	1.42523921
1	-2.35857868	2.37387061	1.07053924	1	-5.82967854	-0.36712939	0.94443917
1	-2.05757856	2.18967080	-1.61186075				

I-2pβ-c-syn

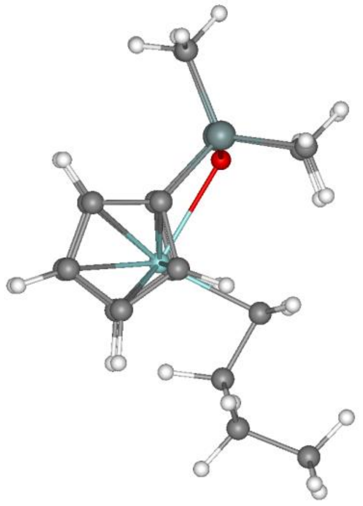

Zero-point vibrational energy	1124744.4 (Joules/Mol)
	268.82037 (Kcal/Mol)
Zero-point correction=	0.428393 (Hartree/Particle)
Thermal correction to Energy=	0.455068
Thermal correction to Enthalpy=	0.456013
Thermal correction to Gibbs Free Energy=	0.374078
Sum of electronic and zero-point Energies=	-4897.550898
Sum of electronic and thermal Energies=	-4897.524222
Sum of electronic and thermal Enthalpies=	-4897.523278
Sum of electronic and thermal Free Energies=	-4897.605212

cartesian

6	1.49550784	-0.19124709	-1.19573927	40	0.45910779	-0.38674706	0.79206085
6	2.76760769	-0.62674707	-0.49393916	6	-1.28299212	2.88195276	-1.54753923
6	3.95730758	0.33315289	-0.61873919	6	-3.38369226	2.24455285	0.67436087
1	1.56720781	0.82275295	-1.59383917	8	-1.54169214	0.23975290	-0.37893915
1	1.17860782	-0.87184709	-1.98543930	1	-4.17089224	2.07445288	-0.06633916
1	2.62160778	-0.74834704	0.63596082	1	-3.45679235	3.29095292	0.98816085
1	4.74190807	0.04255292	0.08716084	1	-3.60479236	1.62355292	1.54736090
1	3.63350773	1.34305286	-0.33993918	1	-2.04319215	2.74405289	-2.32213926
6	0.21730781	-2.45614719	2.27226067	1	-0.32399219	2.56565285	-1.96863914
6	-1.07229221	-2.06564713	1.82966089	1	-1.22359216	3.95285296	-1.33123922
6	-1.14859211	-2.23784709	0.41416085	6	-2.21879220	-1.68584716	-2.44913912
6	0.11680783	-2.74994707	-0.00303915	6	-4.05349207	-1.18944716	0.03016083
6	0.94960785	-2.88594723	1.13746083	1	-4.67889214	-0.48044711	-0.52093917
1	1.96350777	-3.26874709	1.14736080	1	-4.07379198	-0.90364707	1.08606076
1	0.56930786	-2.46354723	3.29686069	1	-4.52879190	-2.17214704	-0.05423915
14	-2.33099222	-1.25634718	-0.65233916	1	-2.86039233	-1.03514707	-3.05073929
1	0.39520779	-3.00034714	-1.01973915	1	-2.53709221	-2.71734715	-2.62903929
6	1.79650784	1.44165289	2.00536084	1	-1.19669211	-1.57624710	-2.82303929
6	0.87600785	0.91615289	2.94716072	1	-1.86539221	-1.69094718	2.46796083
6	-0.43119219	1.21795285	2.48726082	1	-1.35069215	0.94665295	2.99506068
6	-0.33579218	1.92145276	1.24826086	6	4.51870775	0.34305292	-2.03983927
6	1.05620790	2.05535293	0.96216083	1	4.87340784	-0.65144706	-2.32443929
1	1.47760785	2.55105281	0.09606083	1	5.36030817	1.03395283	-2.11913919
1	2.87720776	1.39845288	2.08396077	1	3.75960779	0.64965290	-2.76433921
1	1.13070786	0.41095293	3.87116075	1	3.05650783	-1.64294708	-0.78133911
14	-1.70239210	1.90945292	-0.02963915				

I-2pβ-c-anti

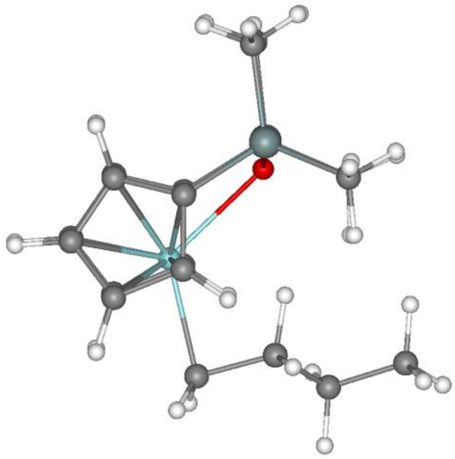

Zero-point vibrational energy	1126178.8 (Joules/Mol)
	269.16319 (Kcal/Mol)
Zero-point correction=	0.428939 (Hartree/Particle)
Thermal correction to Energy=	0.455480
Thermal correction to Enthalpy=	0.456424
Thermal correction to Gibbs Free Energy=	0.374762
Sum of electronic and zero-point Energies=	-4897.552717
Sum of electronic and thermal Energies=	-4897.526176
Sum of electronic and thermal Enthalpies=	-4897.525232
Sum of electronic and thermal Free Energies=	-4897.606895

cartesian

6	2.39017081	2.26416659	-1.20816278	14	-1.40172935	1.04836655	1.48563719
6	2.81867075	-0.20443341	-0.52526277	40	0.92297065	-0.97683340	0.40893722
6	1.96357059	0.79396653	-1.30526280	6	-1.12042940	2.83896661	1.10303724
1	3.38597083	-0.86243343	-1.18136275	6	-3.04132915	0.65536660	2.25253725
1	3.50407076	0.30826658	0.15133721	8	-1.18502939	0.14946657	0.03503720
1	0.87977064	0.80856657	-0.97736275	1	-3.87492919	0.97136664	1.61853719
1	2.40657067	2.56786656	-0.15486279	1	-3.13842916	1.18876660	3.20383716
1	3.42057085	2.34356666	-1.56686282	1	-3.16282916	-0.41113341	2.46273732
1	1.88557065	0.50306660	-2.35766268	1	-1.85502934	3.21326661	0.38363719
6	0.88447064	-3.48383331	0.09173721	1	-0.12612936	3.00576663	0.67523718
6	-0.45722938	-3.01093340	0.08663721	1	-1.19932938	3.45156670	2.00643730
6	-0.65672934	-2.20103335	-1.06886280	6	-1.96542943	0.05336660	-2.74706268
6	0.58667064	-2.19023347	-1.77266276	6	-3.61292934	-1.39993346	-0.51966280
6	1.52437067	-2.98473334	-1.06916285	1	-4.31072903	-0.56253338	-0.42766279
1	2.54567075	-3.18553352	-1.36536276	1	-3.55532932	-1.89973342	0.45173723
1	1.32547069	-4.14893341	0.82453722	1	-4.05112934	-2.11053324	-1.22806275
14	-1.95172942	-0.85443342	-1.13266277	1	-2.61202931	0.93456662	-2.69726276
1	0.78467065	-1.65443337	-2.69486284	1	-2.33582926	-0.58263338	-3.55666280
6	2.26157069	-0.40583342	2.51743722	1	-0.96332932	0.39726657	-3.02286267
6	1.44197059	-1.51693344	2.82853723	1	-1.20252931	-3.22993350	0.84323722
6	0.08537064	-1.09553337	2.74493718	1	-0.77902937	-1.72193336	2.93563724
6	0.05227064	0.27936661	2.37283731	6	1.47717059	3.18466663	-2.01506281
6	1.41207063	0.69016659	2.22383714	1	1.51287067	2.93306661	-3.07896280
1	1.74467063	1.68016648	1.92983723	1	1.77407062	4.23026657	-1.91046286
1	3.34367085	-0.39823341	2.51093721	1	0.43477064	3.09726667	-1.68836284
1	1.78947067	-2.49983335	3.12363720				

I-5pp-c-syn

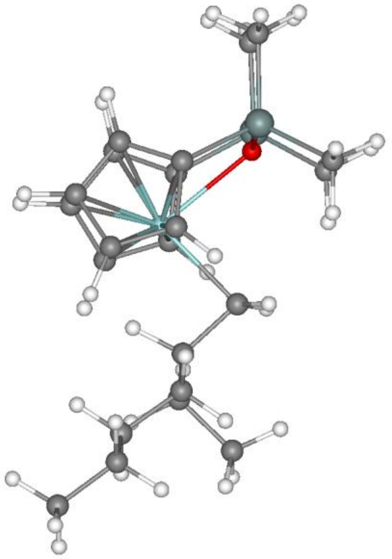

Zero-point vibrational energy	1427632.2 (Joules/Mol)
	341.21230 (Kcal/Mol)
Zero-point correction=	0.543756 (Hartree/Particle)
Thermal correction to Energy=	0.575603
Thermal correction to Enthalpy=	0.576547
Thermal correction to Gibbs Free Energy=	0.482894
Sum of electronic and zero-point Energies=	-5054.620604
Sum of electronic and thermal Energies=	-5054.588758
Sum of electronic and thermal Enthalpies=	-5054.587814
Sum of electronic and thermal Free Energies=	-5054.681467

cartesian

6	0.38965702	0.57373178	-1.17272854	1	-5.67124319	1.70673180	0.74807143
6	1.69415700	0.78933179	-0.43172860	1	-5.12904310	0.13233173	1.33117151
6	2.18915701	2.24963164	-0.46132857	1	-4.06334305	1.76513171	-2.51672840
1	0.00495702	1.48013175	-1.64502847	1	-2.44814301	2.35243154	-2.12132859
1	0.43725705	-0.23256826	-1.90722847	1	-3.87764311	3.21983171	-1.53572857
1	1.54805696	0.59293175	0.70007145	6	-2.26334286	-2.37666845	-2.44772863
1	2.92915726	2.40913153	0.33027142	6	-4.33004284	-2.57606840	-0.11682855
1	1.34785700	2.91303158	-0.23922856	1	-5.14864302	-2.17256832	-0.72062856
6	2.79075718	-0.21986827	-0.80772859	1	-4.53014326	-2.32646847	0.92917144
6	-0.05714297	-1.87596822	2.40577149	1	-4.36864281	-3.66646838	-0.20702855
6	-1.36704302	-2.06116843	1.89557147	1	-3.04004288	-2.02276826	-3.13222861
6	-1.28554296	-2.31956840	0.49397147	1	-2.17164302	-3.45846844	-2.58392859
6	0.09985703	-2.29776835	0.15487143	1	-1.32054305	-1.91426826	-2.75472856
6	0.85135698	-2.02696824	1.32837152	1	-2.28004313	-2.00866818	2.47897148
1	1.93205702	-1.96526825	1.39987147	1	-2.89484310	0.53183174	2.87557149
1	0.20865703	-1.68986821	3.43957138	6	4.03425694	-0.15996826	0.07847144
14	-2.68214321	-1.95316827	-0.69532859	1	3.06655693	-0.04146827	-1.85322857
1	0.50525701	-2.46836829	-0.83472854	1	2.37515688	-1.23326826	-0.78382856
6	-0.21194297	2.29963160	1.97727144	6	5.01355696	-1.30046821	-0.20352857
6	-0.86854297	1.45783174	2.90937138	1	3.73825693	-0.18956827	1.13847148
6	-2.15344286	1.15683174	2.38977146	1	4.55085707	0.79723179	-0.06382857
6	-2.30224299	1.80013180	1.12427151	1	5.29145670	-1.28156829	-1.26342857
6	-1.08914304	2.50983167	0.88167143	1	4.51085711	-2.26086831	-0.03482856
1	-0.88164294	3.11503172	0.00767145	6	6.26995707	-1.21686828	0.66117144
1	0.77855706	2.72363162	2.09747148	1	6.80865669	-0.28296828	0.47757143
1	-0.47074297	1.13623178	3.86447144	1	6.95265675	-2.04306841	0.45167142
14	-3.47154284	1.18403172	-0.19832857	1	6.01715708	-1.25216818	1.72507143
40	-0.57084298	0.04393174	0.80117142	6	2.78785706	2.64393163	-1.81252861
6	-3.46104288	2.22653174	-1.72822857	1	3.73035669	2.12563157	-2.00552845
6	-5.16084290	0.78393173	0.45317143	1	2.99385691	3.71603155	-1.83572841
8	-2.59314299	-0.25446826	-0.49912858	1	2.10165691	2.41423154	-2.63322854
1	-5.78554296	0.29883173	-0.30272859				

TS-56pp-c-syn

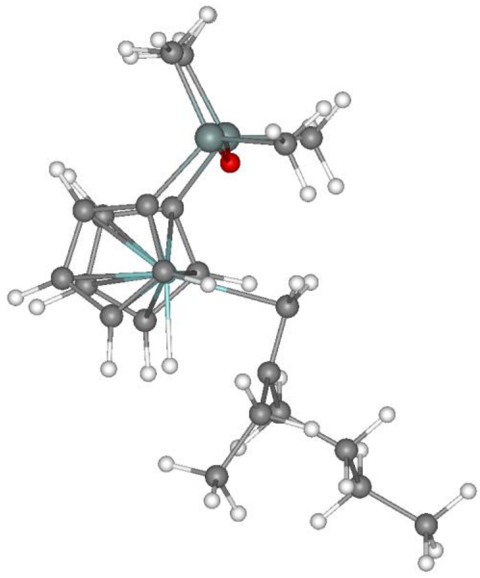

Zero-point vibrational energy	1416059.4 (Joules/Mol)
	338.44631 (Kcal/Mol)
Zero-point correction=	0.539349 (Hartree/Particle)
Thermal correction to Energy=	0.571119
Thermal correction to Enthalpy=	0.572063
Thermal correction to Gibbs Free Energy=	0.478168
Sum of electronic and zero-point Energies=	-5054.602465
Sum of electronic and thermal Energies=	-5054.570695
Sum of electronic and thermal Enthalpies=	-5054.569751
Sum of electronic and thermal Free Energies=	-5054.663646

cartesian

6	-2.20664430	-2.08894920	0.76457775	8	2.24995565	0.66575074	0.72887778
6	-0.72444445	-0.08554925	1.08777785	1	4.82135582	2.58995080	0.43827775
6	-1.78984427	-0.66884929	0.43417779	1	4.27435541	3.48495078	-0.97722220
6	-2.82214427	0.19215076	-0.25452223	1	4.65655565	1.77045071	-1.12102222
1	-0.64394444	0.99935079	1.13107777	1	2.13235569	3.62665081	1.88487768
1	-0.19294441	-0.63824928	1.86007786	1	0.56835556	3.22155070	1.18137777
1	-0.93654448	-1.24464929	-1.27492225	1	1.59715557	4.43355083	0.40857777
1	-3.17394447	-0.28744924	-1.17302227	6	2.68805552	-0.93324924	3.10757780
1	-2.38344431	1.15395069	-0.53342223	6	4.99685574	-0.34574926	1.14127779
1	-1.32924438	-2.67264915	1.05227780	1	5.34165573	0.51845074	1.71687782
1	-2.81644440	-2.00534916	1.67417777	1	5.30185556	-0.20334925	0.10117778
6	1.74415565	-2.66914916	-1.87862217	1	5.53505564	-1.22084928	1.52147782
6	2.78335571	-1.96014929	-1.21892226	1	2.95965552	-0.07704925	3.73247790
6	2.51315570	-1.93784928	0.17897776	1	3.20645571	-1.80974925	3.50857782
6	1.29375553	-2.65514922	0.36527780	1	1.61415553	-1.09744930	3.23117781
6	0.82365561	-3.11074924	-0.89422226	1	3.64035559	-1.50684929	-1.70422220
1	-0.05864441	-3.71234918	-1.07372224	1	2.92875552	0.60985076	-2.86452222
1	1.68155551	-2.87424922	-2.94052219	6	-4.01994419	0.45205075	0.67767775
14	3.17505574	-0.63274926	1.34337783	1	-4.51244450	-0.49364924	0.93277776
1	0.82205558	-2.84064937	1.32327783	1	-3.66114426	0.88435072	1.61977780
6	-0.39934444	0.84325075	-2.70302224	6	-5.04084444	1.39115071	0.03367777
6	0.76515555	0.28645074	-3.29092216	1	-5.38174438	0.95925069	-0.91412222
6	1.89505553	0.83555073	-2.62862229	1	-4.55264425	2.34075069	-0.21392223
6	1.44795561	1.71815073	-1.60522223	6	-6.24014425	1.65045071	0.94387782
6	0.02015558	1.71375072	-1.66552222	1	-6.76704454	0.71995074	1.17287779
1	-0.62964445	2.30655074	-1.03242218	1	-6.95314455	2.33125067	0.47417775
1	-1.41944432	0.65355074	-3.01142216	1	-5.92354441	2.09755063	1.89057779
1	0.78385556	-0.40064925	-4.12832212	6	-3.00914431	-2.82214928	-0.30872223
14	2.42775559	2.16225076	-0.07682224	1	-3.97484446	-2.34394932	-0.49062222
40	0.73735559	-0.56634927	-0.91232228	1	-3.20924425	-3.84724927	0.00977777
6	1.60415554	3.47625065	0.93867779	1	-2.46534443	-2.85614920	-1.25712216
6	4.20675564	2.52005076	-0.46352223				

I-6pp-c-syn

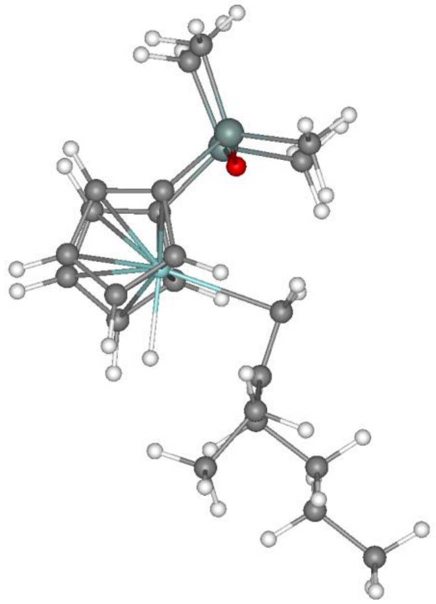

Zero-point vibrational energy	1415453.1 (Joules/Mol)
	338.30141 (Kcal/Mol)
Zero-point correction=	0.539118 (Hartree/Particle)
Thermal correction to Energy=	0.570987
Thermal correction to Enthalpy=	0.571931
Thermal correction to Gibbs Free Energy=	0.477237
Sum of electronic and zero-point Energies=	-5054.602819
Sum of electronic and thermal Energies=	-5054.570950
Sum of electronic and thermal Enthalpies=	-5054.570005
Sum of electronic and thermal Free Energies=	-5054.664700

cartesian

6	-2.21743321	-2.07288408	0.82000637	8	2.23696685	0.66951585	0.74800634
6	-0.72433317	-0.08198416	1.11430633	1	4.78346634	2.63281584	0.52250636
6	-1.81863308	-0.64528418	0.51600635	1	4.25436687	3.52901602	-0.89899361
6	-2.83073330	0.20561585	-0.20519365	1	4.66726685	1.82191586	-1.04559362
1	-0.60473323	1.00011587	1.13050628	1	2.05046678	3.61841583	1.91680622
1	-0.15513319	-0.64248419	1.85400629	1	0.50756681	3.21351576	1.16830635
1	-0.86023319	-1.28238416	-1.39879370	1	1.55266678	4.43301582	0.43190637
1	-3.13553309	-0.27778417	-1.13889360	6	2.66926670	-0.95228416	3.11410642
1	-2.39303327	1.17421579	-0.46289366	6	4.99076653	-0.31628418	1.18070626
1	-1.33273315	-2.64738417	1.10530627	1	5.31896687	0.54871583	1.76470637
1	-2.83553314	-2.01378417	1.72620630	1	5.30756664	-0.16548416	0.14540634
6	1.82146680	-2.65588427	-1.90409362	1	5.53366661	-1.18738413	1.56350636
6	2.83836675	-1.93738413	-1.21959364	1	2.91006684	-0.09388416	3.74860620
6	2.54026675	-1.92618418	0.17230636	1	3.20886683	-1.81668413	3.51350641
6	1.32576680	-2.66038418	0.32930633	1	1.59976685	-1.14958417	3.22640634
6	0.88786680	-3.11638403	-0.94089365	1	3.70006680	-1.47288418	-1.68529367
1	0.01746681	-3.72818422	-1.14059365	1	2.99156690	0.64721584	-2.83229375
1	1.78286684	-2.85528421	-2.96819377	6	-4.07163334	0.44331586	0.67580640
14	3.16976690	-0.62488419	1.35810637	1	-4.54913330	-0.51248419	0.92110634
1	0.83606684	-2.85768414	1.27600634	1	-3.76123309	0.89331585	1.62660635
6	-0.34273320	0.83741581	-2.74849367	6	-5.09023285	1.34901583	-0.01779366
6	0.84276682	0.29971585	-3.31239367	1	-5.38033295	0.89851582	-0.97399366
6	1.94976676	0.85691583	-2.61849380	1	-4.61793327	2.30911589	-0.25559366
6	1.46776676	1.72441590	-1.59869373	6	-6.33213329	1.58371580	0.84010637
6	0.04136682	1.70211589	-1.69299364	1	-6.84063339	0.64091581	1.05980635
1	-0.63153315	2.27981567	-1.07019365	1	-7.04473305	2.23701572	0.33220634
1	-1.35253322	0.63881582	-3.08299375	1	-6.06813335	2.05201578	1.79260635
1	0.88966680	-0.38078415	-4.15399361	6	-3.00283313	-2.80368423	-0.26889366
14	2.40956688	2.17171574	-0.04739365	1	-3.96973300	-2.33068419	-0.45809364
40	0.76726681	-0.57548416	-0.94749367	1	-3.19963312	-3.83158398	0.04230635
6	1.54896677	3.47211599	0.95560634	1	-2.44773316	-2.82818413	-1.21039367
6	4.19056702	2.55951595	-0.39349365				

I-7ppp-syn

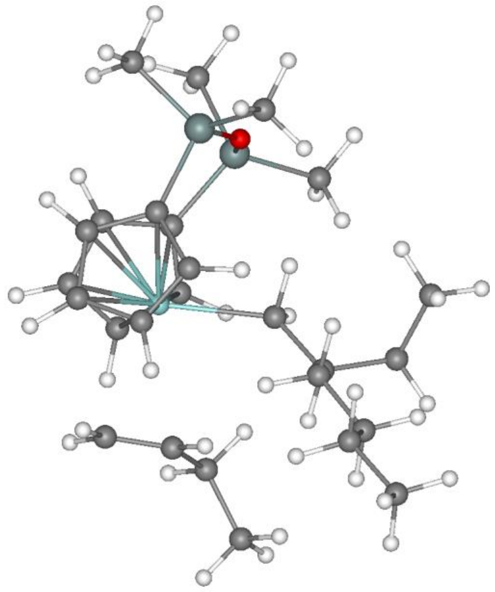

Zero-point vibrational energy	1415453.1 (Joules/Mol)
	338.30141 (Kcal/Mol)
Zero-point correction=	0.539118 (Hartree/Particle)
Thermal correction to Energy=	0.570987
Thermal correction to Enthalpy=	0.571931
Thermal correction to Gibbs Free Energy=	0.477237
Sum of electronic and zero-point Energies=	-5054.602819
Sum of electronic and thermal Energies=	-5054.570950
Sum of electronic and thermal Enthalpies=	-5054.570005
Sum of electronic and thermal Free Energies=	-5054.664700

cartesian

6	-2.21743321	-2.07288408	0.82000637	8	2.23696685	0.66951585	0.74800634
6	-0.72433317	-0.08198416	1.11430633	1	4.78346634	2.63281584	0.52250636
6	-1.81863308	-0.64528418	0.51600635	1	4.25436687	3.52901602	-0.89899361
6	-2.83073330	0.20561585	-0.20519365	1	4.66726685	1.82191586	-1.04559362
1	-0.60473323	1.00011587	1.13050628	1	2.05046678	3.61841583	1.91680622
1	-0.15513319	-0.64248419	1.85400629	1	0.50756681	3.21351576	1.16830635
1	-0.86023319	-1.28238416	-1.39879370	1	1.55266678	4.43301582	0.43190637
1	-3.13553309	-0.27778417	-1.13889360	6	2.66926670	-0.95228416	3.11410642
1	-2.39303327	1.17421579	-0.46289366	6	4.99076653	-0.31628418	1.18070626
1	-1.33273315	-2.64738417	1.10530627	1	5.31896687	0.54871583	1.76470637
1	-2.83553314	-2.01378417	1.72620630	1	5.30756664	-0.16548416	0.14540634
6	1.82146680	-2.65588427	-1.90409362	1	5.53366661	-1.18738413	1.56350636
6	2.83836675	-1.93738413	-1.21959364	1	2.91006684	-0.09388416	3.74860620
6	2.54026675	-1.92618418	0.17230636	1	3.20886683	-1.81668413	3.51350641
6	1.32576680	-2.66038418	0.32930633	1	1.59976685	-1.14958417	3.22640634
6	0.88786680	-3.11638403	-0.94089365	1	3.70006680	-1.47288418	-1.68529367
1	0.01746681	-3.72818422	-1.14059365	1	2.99156690	0.64721584	-2.83229375
1	1.78286684	-2.85528421	-2.96819377	6	-4.07163334	0.44331586	0.67580640
14	3.16976690	-0.62488419	1.35810637	1	-4.54913330	-0.51248419	0.92110634
1	0.83606684	-2.85768414	1.27600634	1	-3.76123309	0.89331585	1.62660635
6	-0.34273320	0.83741581	-2.74849367	6	-5.09023285	1.34901583	-0.01779366
6	0.84276682	0.29971585	-3.31239367	1	-5.38033295	0.89851582	-0.97399366
6	1.94976676	0.85691583	-2.61849380	1	-4.61793327	2.30911589	-0.25559366
6	1.46776676	1.72441590	-1.59869373	6	-6.33213329	1.58371580	0.84010637
6	0.04136682	1.70211589	-1.69299364	1	-6.84063339	0.64091581	1.05980635
1	-0.63153315	2.27981567	-1.07019365	1	-7.04473305	2.23701572	0.33220634
1	-1.35253322	0.63881582	-3.08299375	1	-6.06813335	2.05201578	1.79260635
1	0.88966680	-0.38078415	-4.15399361	6	-3.00283313	-2.80368423	-0.26889366
14	2.40956688	2.17171574	-0.04739365	1	-3.96973300	-2.33068419	-0.45809364
40	0.76726681	-0.57548416	-0.94749367	1	-3.19963312	-3.83158398	0.04230635
6	1.54896677	3.47211599	0.95560634	1	-2.44773316	-2.82818413	-1.21039367
6	4.19056702	2.55951595	-0.39349365				

TS-78

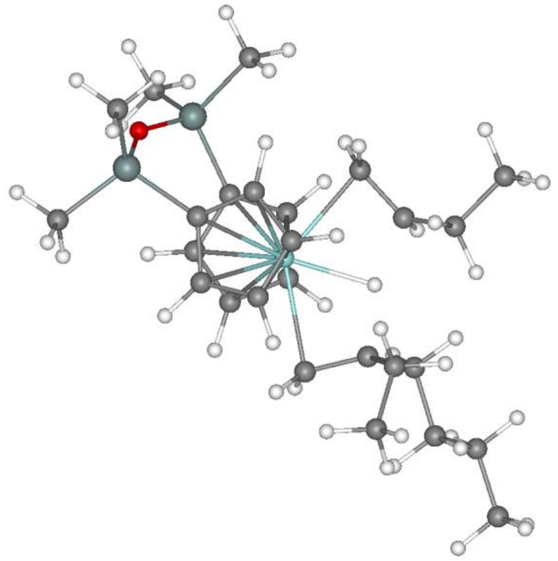

Zero-point vibrational energy	1713278.0 (Joules/Mol)
	409.48326 (Kcal/Mol)
Zero-point correction=	0.652553 (Hartree/Particle)
Thermal correction to Energy=	0.690119
Thermal correction to Enthalpy=	0.691063
Thermal correction to Gibbs Free Energy=	0.585864
Sum of electronic and zero-point Energies=	-5211.629481
Sum of electronic and thermal Energies=	-5211.591916
Sum of electronic and thermal Enthalpies=	-5211.590971
Sum of electronic and thermal Free Energies=	-5211.696170

cartesian

6	0.95171463	-2.33066654	0.76562667
6	2.15651464	-1.59776664	0.96062666
6	1.90911460	-0.74176663	2.08502674
6	0.58871466	-0.91886663	2.53052664
6	-0.01878536	-1.90076661	1.69542670
1	-1.01688528	-2.30616665	1.80482674
1	0.79171467	-3.10386658	0.02232669
14	3.88801455	-1.78676665	0.25142670
1	2.63511467	-0.07366665	2.53442669
1	0.13651463	-0.42156664	3.37822652
6	0.76491463	2.11653328	-1.38637328
6	2.04121470	1.49643338	-1.30017328
6	2.55071449	1.64453340	0.01622668
6	1.55261469	2.36623335	0.74852663
6	0.48311463	2.68123341	-0.12157332
1	-0.39568537	3.25893331	0.13342668
1	0.15471464	2.19663334	-2.27877331
1	2.56851459	1.02643335	-2.12197351
14	4.34601450	1.30223334	0.50382668
1	1.62821460	2.66943336	1.78762674
6	4.68311501	2.07143331	2.16452670
6	5.41851473	2.01673341	-0.83657336
8	4.60141468	-0.33036664	0.57022667
1	6.47721481	1.87903333	-0.59657335
1	5.23981476	3.08753324	-0.97187334
1	5.23541498	1.52253330	-1.79627323
1	5.71141481	1.85683334	2.47182655
1	4.02731466	1.70453334	2.95992661
1	4.57571459	3.16003346	2.11962652
6	4.78891468	-3.11396670	1.18562675
6	3.87401462	-2.11776662	-1.58547330
1	4.90041494	-2.24976659	-1.94377339
1	3.43591452	-1.28886664	-2.15067339
1	3.32711458	-3.03156662	-1.84227324
1	5.82931471	-3.18326664	0.85422665
1	4.32491493	-4.09426689	1.04062665
1	4.79191494	-2.90036654	2.25822663
40	0.48261464	0.14083335	0.20032668
6	-1.70498538	-0.40246665	-2.91337347
1	-1.27828526	0.56113333	-3.20997334
6	0.55781466	-1.04736662	-1.99077332
6	-0.83408540	-1.04306662	-1.84657323
1	-1.28148532	-1.90626669	-1.34547329
1	1.13441467	-1.92706668	-1.73837328
1	0.97711468	-0.41906664	-2.77127337
1	-1.32438540	-0.07686665	-0.69077337
1	-2.71208549	-0.21346664	-2.53687334
6	-1.48348546	0.97043335	1.45882666
6	-2.24578547	0.66173333	0.30932668
6	-3.20768547	-0.51946664	0.42022669
1	-1.27348542	2.00203323	1.71562672
1	-1.61618543	0.31163335	2.31012654
6	-2.72388554	1.79303336	-0.61647332
1	-3.43248534	-0.93466663	-0.56937337
1	-2.74368548	-1.31626666	1.00952673
6	-1.78358531	-1.33616662	-4.12847328
1	-0.79548532	-1.49236667	-4.56667328
1	-2.43738532	-0.90936667	-4.89147329
1	-2.18758535	-2.31186676	-3.84527349
6	-4.52618504	-0.10926665	1.09232664
1	-4.31098509	0.38703334	2.04662657
6	-5.44128513	-1.31186664	1.33272672
1	-5.05348539	0.62043333	0.46632668
1	-3.49748540	1.38713336	-1.27477324
1	-1.92268538	2.13993335	-1.27317333
6	-3.30298543	2.99203324	0.14612669
1	-3.74678540	3.69743323	-0.55967337
1	-2.53698540	3.52843332	0.71082664
1	-4.07608509	2.68323326	0.85292667
6	-6.76268530	-0.90676665	1.98372674
1	-4.92338514	-2.03836656	1.96932673
1	-5.63728523	-1.81546664	0.37922668
1	-7.39868498	-1.77666664	2.16082668
1	-7.31678534	-0.21116665	1.34732676
1	-6.59068537	-0.41636664	2.94592667

TS-79ppp-syn

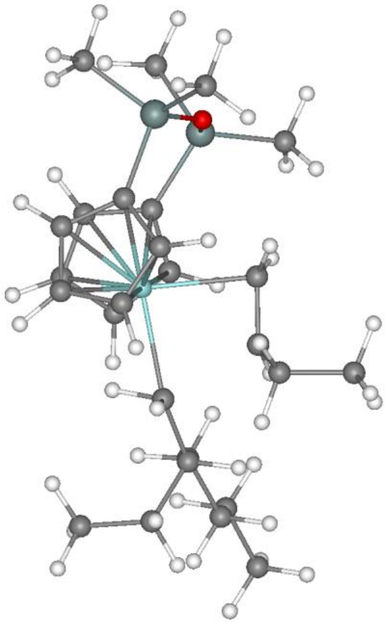

Zero-point vibrational energy	1726258.0 (Joules/Mol)
	412.58557 (Kcal/Mol)
Zero-point correction=	0.657497 (Hartree/Particle)
Thermal correction to Energy=	0.694399
Thermal correction to Enthalpy=	0.695343
Thermal correction to Gibbs Free Energy=	0.591252
Sum of electronic and zero-point Energies=	-5211.642884
Sum of electronic and thermal Energies=	-5211.605982
Sum of electronic and thermal Enthalpies=	-5211.605038
Sum of electronic and thermal Free Energies=	-5211.709128

cartesian

1	2.62699604	0.84238136	-1.28909206
6	0.58699596	0.63338137	-0.74139202
6	1.94669592	0.00828135	-1.06569195
6	1.84209597	-0.81451869	-2.37149215
6	2.57599592	-0.79581869	0.07590798
1	-0.13730401	-0.21941864	-0.55679202
1	0.22589600	1.13888133	-1.63879204
1	2.85169601	-1.01651859	-2.74239206
1	1.36399603	-0.19051865	-3.13629198
6	0.53879595	2.99188137	1.42230797
6	1.32119596	2.50478148	0.35870796
6	1.46909595	3.33158135	-0.89839202
1	0.87619603	2.79448128	2.43410802
1	0.03229600	3.94278145	1.28170800
1	2.17359591	1.88068140	0.61860800
1	1.76179612	2.72038150	-1.75459194
1	0.51279604	3.80578136	-1.14079213
1	1.99809611	-1.71191859	0.24530798
1	2.52359581	-0.22111866	1.01160800
6	-0.89580399	0.85828137	3.40690804
6	-2.01070404	0.14608136	2.90720797
6	-1.55720401	-0.90791857	2.05470800
6	-0.12700400	-0.85131860	2.09960794
6	0.27709597	0.22838137	2.90740800
1	1.29969597	0.51558137	3.12420797
1	-0.92960399	1.70778143	4.07920790
14	-2.53140402	-2.16951871	1.04360795
1	0.53759599	-1.53851867	1.59380794
6	-2.14050388	3.26898146	-0.16799203
6	-2.96220398	2.81418133	0.89520800
6	-3.44390392	1.53078139	0.53830802
6	-2.89560413	1.15028143	-0.72169203
6	-2.09490395	2.25638127	-1.14959192
1	-1.55910397	2.31778145	-2.09009218
1	-1.65740395	4.23618126	-0.22339201
1	-3.21300411	3.36808133	1.79260802
14	-3.17380381	-0.51591861	-1.55909204
40	-0.97240400	1.21358144	0.88820797
6	-2.25610399	-0.55551863	-3.17489219
6	-4.99790430	-0.83081865	-1.72489190
8	-2.49440384	-1.63341856	-0.53149199
1	-5.18490410	-1.83371866	-2.12119198
1	-5.46420431	-0.11061865	-2.40389204
1	-5.51110411	-0.75461864	-0.76089203
1	-2.32220411	-1.55051863	-3.62619209
1	-1.19160402	-0.32871863	-3.04859209
1	-2.67330408	0.16038135	-3.89019203
6	-1.63530397	-3.79321861	1.09670806
6	-4.28190422	-2.25161862	1.67540789
1	-4.89620399	-2.88671851	1.02900791
1	-4.76980400	-1.27221859	1.72540808
1	-4.31320381	-2.68111873	2.68200803
1	-2.17670393	-4.55231857	0.52450800
1	-1.51810396	-4.16161871	2.11990809
1	-0.63850403	-3.70371866	0.65230799
1	-3.04440403	0.37468135	3.13810802
1	-4.13020420	0.93608141	1.12990797
6	4.03269577	-1.18721867	-0.18049201
1	4.09189606	-1.85591865	-1.04749203
1	4.61359596	-0.29221866	-0.43989199
6	4.67459583	-1.87851858	1.02300799
1	4.63679600	-1.20711863	1.88930798
1	4.08329582	-2.76251864	1.28960800
6	1.08419597	-2.13841867	-2.23969197
1	0.92649603	-2.58411860	-3.22489214
1	0.09589601	-2.01611853	-1.77929211
1	1.64389598	-2.86521864	-1.64389205
6	6.12139606	-2.29021859	0.75550801
1	6.56589603	-2.77421856	1.62790799
1	6.73539591	-1.42011857	0.50570798
1	6.17989588	-2.99071860	-0.08229202
6	2.54009581	4.40718126	-0.66049200
1	2.26219606	5.06458139	0.16610798
1	2.66679621	5.01508141	-1.55839205
1	3.50469589	3.95058131	-0.42309201

I-9ppp-c-syn

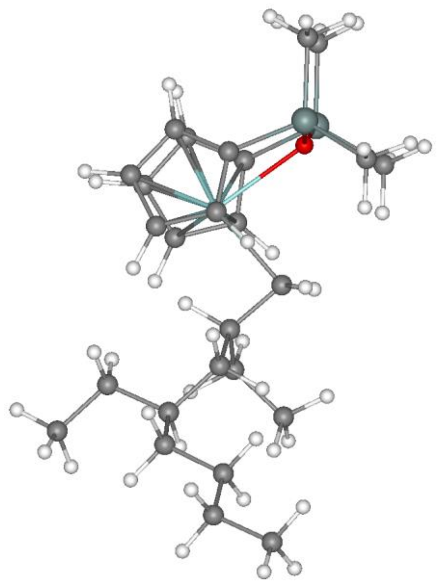

Zero-point vibrational energy	1729098.4 (Joules/Mol)
	413.26445 (Kcal/Mol)
Zero-point correction=	0.658579 (Hartree/Particle)
Thermal correction to Energy=	0.695811
Thermal correction to Enthalpy=	0.696756
Thermal correction to Gibbs Free Energy=	0.590299
Sum of electronic and zero-point Energies=	-5211.686099
Sum of electronic and thermal Energies=	-5211.648866
Sum of electronic and thermal Enthalpies=	-5211.647922
Sum of electronic and thermal Free Energies=	-5211.754378

cartesian

6	0.39400399	0.06941064	-1.12849998
6	-0.73119599	-0.80338937	-0.59540004
6	-1.06829596	-1.98548937	-1.52590001
1	0.77320397	-0.26658937	-2.09619999
1	0.12920398	1.12671053	-1.18820000
1	-0.39809602	-1.31508946	0.38130000
1	-1.68009591	-2.72148919	-0.99219996
1	-0.14189601	-2.49678922	-1.80429995
6	-1.98139596	0.00161063	-0.19059999
6	1.33750391	-0.06498937	3.12129998
6	2.49810386	0.62151062	2.68569994
6	2.11450386	1.63611054	1.75610006
6	0.69570398	1.56341052	1.63480008
6	0.22220397	0.51981062	2.47200012
1	-0.81149608	0.22951064	2.61380005
1	1.29960394	-0.87128937	3.84410000
14	3.28440404	2.29921079	0.45760000
1	0.08280396	2.19951057	1.00769997
6	1.70100403	-3.06748939	0.19030000
6	2.44360399	-2.85088921	1.37810004
6	3.58670402	-2.08228922	1.03920007
6	3.55830407	-1.80428946	-0.36010003
6	2.38180399	-2.42458940	-0.87640005
1	2.07080388	-2.41168928	-1.91389990
1	0.78370398	-3.64088941	0.11470000
1	2.19780397	-3.23838925	2.35960007
14	4.41120386	-0.32948938	-1.13009989
40	1.65820396	-0.53628939	0.63180000
6	4.21380377	-0.25548938	-2.96919990
6	6.14800358	-0.09098937	-0.52620000
8	3.37280393	0.82681066	-0.41200000
1	6.57420397	0.85281062	-0.87920004
1	6.78750372	-0.89538938	-0.90410000
1	6.21620369	-0.10288937	0.56559998
1	4.62460375	0.67661065	-3.36859989
1	3.16040397	-0.30048937	-3.26049995
1	4.73720360	-1.08228946	-3.45879984
6	2.51820397	3.59301066	-0.62260002
6	4.95270395	2.74411058	1.13410008
1	5.67750359	2.93101072	0.33600003
1	5.36410379	1.96771061	1.78570008
1	4.87990379	3.66291070	1.72530007
1	3.18870401	3.86451077	-1.44339991
1	2.30360389	4.50491047	-0.05739999
1	1.58240390	3.24041080	-1.06599998
1	3.51160407	0.40401062	3.00580001
1	4.34920406	-1.75248945	1.73660004
6	-3.17789602	-0.80078936	0.34720001
1	-2.29399586	0.55181062	-1.08419991
1	-1.70269608	0.76861066	0.54479998
6	-4.33649635	0.14611064	0.69999999
1	-3.53519607	-1.46238947	-0.45550001
1	-5.20139599	-0.45608938	0.99799997
1	-4.05769634	0.74161065	1.58180010
6	-1.79189587	-1.54568946	-2.80069995
1	-2.79029608	-1.15548944	-2.58789992
1	-1.90809608	-2.39258933	-3.48029995
1	-1.23329592	-0.76608938	-3.32659984
6	-2.81209588	-1.68908942	1.54690003
1	-2.50849605	-1.05188942	2.38849998
1	-1.94489598	-2.31558919	1.30010009
6	-3.94419599	-2.61188936	2.00699997
1	-4.77199602	-2.05398941	2.44860005
1	-3.58729577	-3.31808925	2.76040006
1	-4.33799601	-3.19068933	1.16550004
6	-4.77909613	1.08531058	-0.42559999
1	-4.01149607	1.84471059	-0.61669999
1	-4.89279604	0.51451063	-1.35829997
6	-6.09629631	1.79361057	-0.10469998
1	-6.88129616	1.04351056	0.04150000
1	-5.99509621	2.32461071	0.84909999
6	-6.51619625	2.77371073	-1.19859993
1	-7.46119642	3.26421070	-0.95429999
1	-6.64399624	2.26031065	-2.15629983
1	-5.76119614	3.55351067	-1.33709991

S1.7. Zr-Al1 O[SiMe2(η5-C5H4)]2Zr-based catalytic species

I-5-pp-H

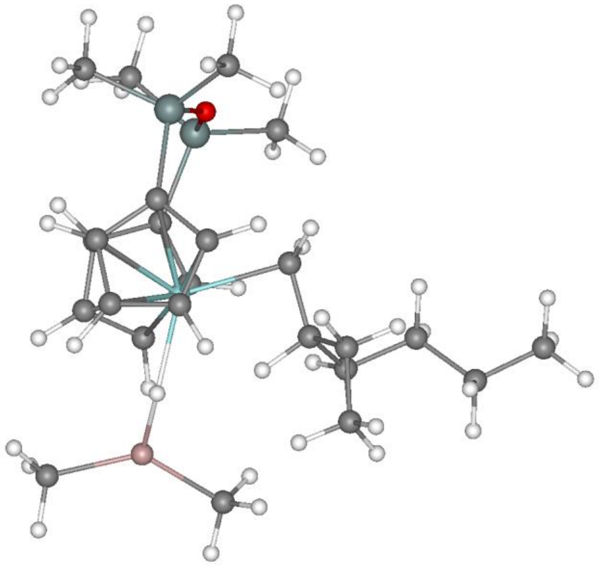

Zero-point vibrational energy	1639725.2 (Joules/Mol)
	391.90374 (Kcal/Mol)
Zero-point correction=	0.624538 (Hartree/Particle)
Thermal correction to Energy=	0.663250
Thermal correction to Enthalpy=	0.664194
Thermal correction to Gibbs Free Energy=	0.554247
Sum of electronic and zero-point Energies=	-5377.317571
Sum of electronic and thermal Energies=	-5377.278860
Sum of electronic and thermal Enthalpies=	-5377.277916
Sum of electronic and thermal Free Energies=	-5377.387863

cartesian

1	-0.77011502	2.61716437	-0.65859038
1	-1.65311491	0.66666436	-0.57429039
13	-1.61461508	3.51256442	0.44690961
6	-3.35531497	2.76986432	0.86980963
6	-0.85501504	5.26036453	0.79840958
1	-3.81351471	2.29826450	-0.00709039
1	-3.30201483	2.00086427	1.64860964
1	-4.04381514	3.54256439	1.22610962
1	0.01168501	5.49206448	0.17210960
1	-1.60191500	6.04266453	0.62540960
1	-0.54431498	5.36376429	1.84500968
6	-0.29891500	-1.03953564	-0.23329039
6	-1.67961514	-0.48043561	-0.52299035
6	-2.68021488	-0.73923564	0.62400961
6	-2.21841502	-0.87083560	-1.90969038
1	0.05228502	-1.75553572	-0.97489041
1	-0.23501500	-1.49243569	0.75530958
1	-3.59861469	-0.16523565	0.45650962
1	-2.24231482	-0.33983564	1.54630971
1	-2.48691487	-1.93263566	-1.87789035
1	-1.41151500	-0.79003567	-2.64469051
6	1.29458499	2.39056444	-2.32839036
6	2.44138503	1.82236433	-1.74169028
6	2.36828494	0.39286435	-1.83859038
6	1.16568506	0.11446435	-2.55359054
6	0.48768499	1.32436430	-2.82509041
1	-0.45311502	1.43316436	-3.35379052
1	1.06848502	3.44766450	-2.40019035
14	3.61638498	-0.89233559	-1.23619032
1	0.83188498	-0.87223560	-2.84619045
6	0.12468499	2.04536438	2.06080961
6	1.28628504	2.69176435	1.53570962
6	2.28168511	1.70596433	1.39150965
6	1.76928496	0.43486434	1.80360973
6	0.43508500	0.66936433	2.22770953
1	-0.23591498	-0.07873563	2.63360953
1	-0.75661504	2.52426434	2.48740959
1	1.39698505	3.74946451	1.32560968
14	2.76968503	-1.17003572	1.78920972
40	0.51448500	1.07926428	-0.31369039
6	1.80618501	-2.55243564	2.57140946
6	4.37418509	-0.83823568	2.67400956
8	3.07188511	-1.51383567	0.20030960
1	5.02898502	-1.71373570	2.62350965
1	4.20728493	-0.60803562	3.73080945
1	4.91948509	0.00356436	2.23480964
1	2.44658518	-3.43423557	2.67500949
1	0.93768501	-2.85483551	1.98060977
1	1.46438491	-2.28183556	3.57550955
6	3.68688512	-2.27513552	-2.47059035
6	5.24698496	-0.02753563	-0.97469038
1	5.99018526	-0.72013563	-0.56669039
1	5.16888523	0.80956435	-0.27169037
1	5.64448500	0.36946434	-1.91409028
1	4.41088486	-3.03283548	-2.15659046
1	3.98088503	-1.91823566	-3.46189046
1	2.71678519	-2.77323556	-2.56329036
1	3.25118518	2.38436437	-1.29059029
1	3.29078507	1.89116430	1.04080963
6	-3.40431499	-0.02833565	-2.37479043
1	-4.26181507	-0.11703564	-1.70219028
1	-3.73491478	-0.33503562	-3.36959052
1	-3.13011479	1.03276432	-2.42989039
6	-3.03271484	-2.22793555	0.81100959
1	-3.06731486	-2.45903563	1.88120961
1	-2.23341489	-2.85563564	0.39570960
6	-4.37561512	-2.63463569	0.19610961
1	-4.39001513	-2.40163565	-0.87469041
1	-5.17121506	-2.03383565	0.65180963
6	-4.67201471	-4.11973572	0.39730960
1	-3.90771484	-4.73963547	-0.08069039
1	-5.63991499	-4.39383554	-0.02799039
1	-4.68911505	-4.37373543	1.46110964

I-5-pp-Cl

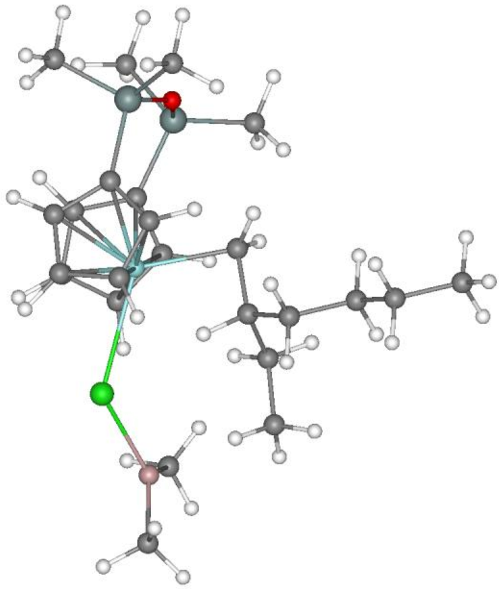

Zero-point vibrational energy	1625116.2 (Joules/Mol)
	388.41209 (Kcal/Mol)
Zero-point correction=	0.618974 (Hartree/Particle)
Thermal correction to Energy=	0.659274
Thermal correction to Enthalpy=	0.660219
Thermal correction to Gibbs Free Energy=	0.546601
Sum of electronic and zero-point Energies=	-5836.950385
Sum of electronic and thermal Energies=	-5836.910085
Sum of electronic and thermal Enthalpies=	-5836.909141
Sum of electronic and thermal Free Energies=	-5837.022759

cartesian

17	-2.18330836	-2.48832321	1.22288632
1	-1.56350839	0.10287669	0.82718629
13	-4.09230852	-2.50482321	0.07458626
6	-3.90330839	-2.97062325	-1.79431367
6	-5.59500837	-2.34002328	1.26958632
1	-4.88330841	-2.95692325	-2.28321362
1	-3.25020838	-2.29782343	-2.35851359
1	-3.50490832	-3.98492336	-1.91091371
1	-5.83310843	-3.31042337	1.71958637
1	-5.39410830	-1.64862323	2.09288621
1	-6.49390841	-1.99972332	0.74648625
6	0.09439154	0.78727674	-0.43811375
6	-1.36620843	0.85417676	0.00838627
6	-2.33960843	0.45107672	-1.11351371
6	-1.73730838	2.18317676	0.69568628
1	0.69989151	1.60557675	-0.04651374
1	0.18429154	0.77717674	-1.52431369
1	-2.23090839	1.14647675	-1.95221376
1	-2.04190850	-0.53242332	-1.50421381
1	-2.71090841	2.08947682	1.19278622
1	-1.00820839	2.37477684	1.49248624
6	0.46769154	-1.77222323	3.00728631
6	1.77359164	-1.80492330	2.47838640
6	2.13989162	-0.49222329	2.03388619
6	1.03489161	0.34997669	2.35608625
6	0.00139154	-0.42902330	2.92548633
1	-0.96090847	-0.06912330	3.27238631
1	-0.08310847	-2.61542320	3.40738630
14	3.78669167	0.08267668	1.30948615
1	0.99709153	1.41967678	2.19388628
6	-0.15100847	-2.88742328	-1.35371375
6	0.69549155	-3.52902317	-0.40271372
6	1.94059157	-2.86222339	-0.43451375
6	1.87549162	-1.76872325	-1.35051370
6	0.57189155	-1.82592332	-1.93211365
1	0.19529153	-1.14952326	-2.69061375
1	-2.84600830	5.76667690	-1.23441362
1	-1.16610837	-3.18032336	-1.59381366
1	0.44079155	-4.39122343	0.20248626
14	3.29159164	-0.58022332	-1.72191381
40	0.27819154	-1.24702334	0.54238629
6	2.79999161	0.61887670	-3.05371356
6	4.78629160	-1.59112322	-2.18261361
8	3.60219169	0.25437668	-0.32481372
1	5.66349173	-0.94932330	-2.31091356
1	4.62689161	-2.13082337	-3.12121367
1	5.02969170	-2.33232331	-1.41421366
1	3.66609168	1.21787667	-3.35211372
1	2.02749157	1.31547678	-2.71611357
1	2.43699169	0.10507670	-3.94951367
6	4.19499159	1.74737668	2.02138638
6	5.07079172	-1.21702325	1.68028617
1	6.02149153	-0.95952332	1.20198631
1	4.78829145	-2.21102333	1.31608629
1	5.25449181	-1.30222332	2.75608635
1	5.16159153	2.09867668	1.64858627
1	4.24499178	1.71947670	3.11378622
1	3.44729161	2.49367666	1.73508620
1	2.39979148	-2.68772316	2.42908621
1	2.81039166	-3.13912320	0.15008627
6	-1.76600838	3.37717676	-0.25881374
1	-0.81490844	3.44007683	-0.80251372
1	-2.54710841	3.23417664	-1.01681376
6	-2.02590847	4.69617653	0.47138625
1	-2.97540832	4.62737656	1.01478624
1	-1.24740839	4.84997654	1.22728634
6	-3.80060840	0.41147670	-0.66421372
1	-4.44360828	-0.07332331	-1.40671372
1	-4.20700836	1.41397679	-0.50281376
1	-3.90640831	-0.08982331	0.31288627
6	-2.06220841	5.88977671	-0.48141372
1	-1.10910845	5.99967670	-1.00681376
1	-2.25750852	6.82077694	0.05498627

I-5-pp-c-H

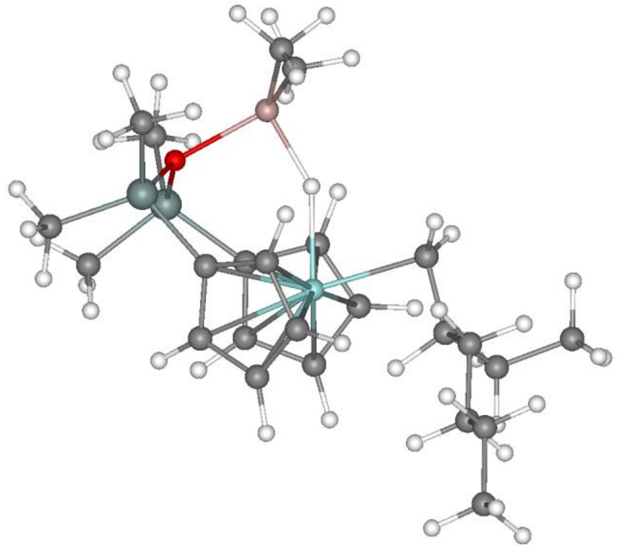

Zero-point vibrational energy	1649093.0 (Joules/Mol)
	394.14269 (Kcal/Mol)
Zero-point correction=	0.628106 (Hartree/Particle)
Thermal correction to Energy=	0.665595
Thermal correction to Enthalpy=	0.666539
Thermal correction to Gibbs Free Energy=	0.561638
Sum of electronic and zero-point Energies=	-5377.333299
Sum of electronic and thermal Energies=	-5377.295811
Sum of electronic and thermal Enthalpies=	-5377.294867
Sum of electronic and thermal Free Energies=	-5377.399768

cartesian

1	0.97531223	-0.43985069	-1.22097945
1	-2.29828763	-0.54385072	0.74502057
13	2.52261233	-0.42605069	-1.92257953
6	3.21491241	-2.22585082	-2.23317957
6	2.38121223	0.68834931	-3.51747942
1	2.49761224	-2.71675086	-2.90607953
1	4.14631224	-2.14675069	-2.80887938
1	3.41131234	-2.92225075	-1.41737950
1	1.53971231	1.38724923	-3.53387952
1	3.28551221	1.24394929	-3.78237939
1	2.20741224	-0.02025071	-4.33827925
6	-1.49028766	-1.14465082	-1.21057940
6	-2.66158772	-0.97225070	-0.26647946
6	-3.36618757	-2.27435064	0.17332056
6	-3.66628790	0.07244929	-0.78507948
1	-1.56508768	-0.53055072	-2.10967946
1	-1.27518773	-2.18015075	-1.48117948
1	-4.02868748	-2.05725074	1.01792061
1	-2.62068772	-2.97595072	0.55112058
1	-4.24008751	-0.40225071	-1.58837950
1	-3.12568760	0.88674933	-1.27717948
6	-0.85868764	1.73944926	1.68862057
6	0.55071229	1.73634923	1.75632060
6	1.08611226	1.91444921	0.44762054
6	-0.04328772	2.08484936	-0.42677945
6	-1.21988773	1.99204922	0.33172056
1	-2.22968769	2.11904931	-0.03227944
1	-1.54218769	1.65294921	2.52552056
14	2.88611221	2.04904914	-0.00497944
1	0.00551230	2.26034927	-1.49617946
6	-0.02478772	-2.84315085	1.07702053
6	-0.12648770	-2.06765079	2.26922059
6	1.07151234	-1.32895076	2.39352059
6	1.90771234	-1.58585072	1.25822055
6	1.20291233	-2.55425072	0.46832055
1	1.53921235	-2.98295069	-0.46647948
1	-0.74718773	-3.55495071	0.70452052
1	-0.94068766	-2.09165072	2.98462057
14	3.59171224	-0.81285071	0.98812056
40	-0.11258772	-0.35025069	0.40192056
6	4.96641254	-1.94525075	0.45232055
6	4.06421232	-0.03405071	2.60932064
8	3.34771228	0.37204930	-0.29927945
1	4.99251223	0.53984928	2.56032062
1	4.25271225	-0.88215071	3.28092051
1	3.29941225	0.58094931	3.08642054
1	5.81331253	-1.81795073	1.13422060
1	5.31611252	-1.73065078	-0.55927944
1	4.67011213	-2.99675083	0.48942056
6	3.16651225	3.04194927	-1.54557943
6	3.90751243	2.81424928	1.34882057
1	4.97241211	2.59194922	1.22852051
1	3.61571240	2.56904912	2.36912060
1	3.79841232	3.89874935	1.23792052
1	4.13041210	2.80494928	-2.00577950
1	3.20141220	4.09594965	-1.24887943
1	2.39501238	2.94174933	-2.30717945
1	1.12731230	1.61934924	2.66692066
1	1.30941224	-0.67665070	3.22452044
6	-4.16378784	-2.94735074	-0.94527948
1	-5.06288767	-2.38235068	-1.20127940
1	-4.48618746	-3.94245076	-0.63127947
1	-3.56348777	-3.05805087	-1.85327947
6	-4.61848783	0.63344932	0.26892057
1	-5.26848745	-0.16375071	0.65042055
1	-4.04478788	0.99744934	1.13552058
6	-5.49008751	1.76934922	-0.26937944
1	-6.04758787	1.41184926	-1.14227951
1	-4.84708786	2.58274913	-0.62827945
6	-6.46138763	2.30674934	0.78002053
1	-7.13718748	1.51944923	1.12582052
1	-7.07168770	3.11884928	0.37922055
1	-5.92338753	2.69094920	1.65202057

I-5-pp-c-Cl

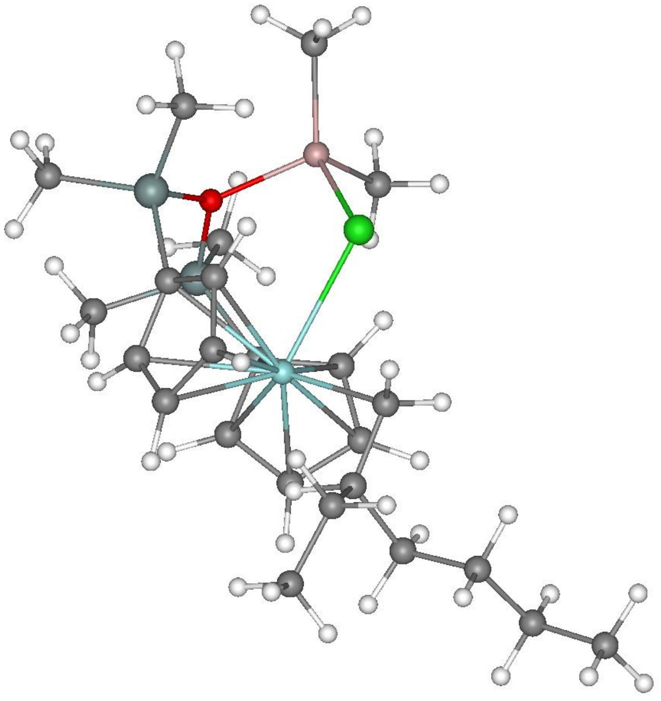

Zero-point vibrational energy	1635450.1 (Joules/Mol)
	390.88196 (Kcal/Mol)
Zero-point correction=	0.622910 (Hartree/Particle)
Thermal correction to Energy=	0.661536
Thermal correction to Enthalpy=	0.662481
Thermal correction to Gibbs Free Energy=	0.556379
Sum of electronic and zero-point Energies=	-5836.967365
Sum of electronic and thermal Energies=	-5836.928739
Sum of electronic and thermal Enthalpies=	-5836.927795
Sum of electronic and thermal Free Energies=	-5837.033896

cartesian

17	-1.16310132	-0.78006440	2.08218908
1	2.25329852	1.00633562	-0.67041093
13	-3.25750136	-1.57426441	1.50318909
6	-3.02870131	-3.40186453	0.84838909
6	-4.56150103	-1.32676446	2.92348909
1	-2.40130138	-3.91346431	1.59218907
1	-4.00260115	-3.90606451	0.88828909
1	-2.60200143	-3.62946439	-0.12941092
1	-4.24730110	-0.69866443	3.76058912
1	-5.51720095	-0.93456441	2.55688906
1	-4.77830124	-2.31896448	3.33738899
6	1.98889875	0.07223560	1.29738903
6	2.89079857	0.66713560	0.22948907
6	3.88369870	-0.33856443	-0.38881090
6	3.60859847	1.96153557	0.65448910
1	1.96099854	0.64993560	2.22438908
1	2.19519854	-0.97726440	1.51778901
1	4.26999903	0.05363560	-1.33721101
1	3.36579871	-1.26816440	-0.63281089
1	4.28549910	1.70813560	1.47628903
1	2.88769865	2.66243553	1.07418907
6	0.27249870	2.88773561	-0.54421091
6	-0.99370134	2.42473555	-0.96671087
6	-1.75010133	2.01443553	0.17588907
6	-0.92090130	2.29773569	1.31138909
6	0.30359867	2.82013559	0.88128906
1	1.09769869	3.15113568	1.53328907
1	1.05329859	3.27893567	-1.18681097
14	-3.50830126	1.43013561	0.40848908
1	-1.18340135	2.10873556	2.34538913
6	0.92979860	-1.81256437	-1.11111093
6	1.00269866	-0.80816442	-2.11891103
6	-0.31260133	-0.36656442	-2.37971091
6	-1.22580135	-1.07616436	-1.52871096
6	-0.42130134	-1.98116446	-0.76901090
1	-0.77090132	-2.67386436	-0.01691092
1	1.74549866	-2.39166451	-0.70261091
1	1.90179873	-0.46516442	-2.61871099
14	-3.07920146	-0.86506444	-1.57791102
40	0.19309869	0.39853558	-0.02431092
6	-4.09970140	-2.37446451	-1.95561099
6	-3.46920133	0.42053559	-2.86681104
8	-3.51060128	-0.29076442	0.02888908
1	-4.54480124	0.60513556	-2.93771100
1	-3.16550136	-0.01556440	-3.82751107
1	-2.96500134	1.38373554	-2.77791095
1	-4.71720123	-2.16656446	-2.83541107
1	-4.77040100	-2.64346433	-1.13661098
1	-3.48470140	-3.24836445	-2.18201089
6	-3.94310141	1.68303561	2.19238901
6	-4.76310110	2.29553556	-0.65681094
1	-5.62200117	1.65023553	-0.86641091
1	-4.37880135	2.67733550	-1.60321093
1	-5.14000130	3.15343547	-0.08971092
1	-4.96900129	1.37843561	2.41388893
1	-3.88130140	2.76423550	2.37008905
1	-3.27960134	1.19483554	2.90878892
1	-1.32010138	2.39973569	-1.99831104
1	-0.58160132	0.37483558	-3.12331104
6	5.06019878	-0.67796439	0.53198910
1	5.72039890	0.19063561	0.63938910
1	4.68929911	-0.91436440	1.53768897
6	5.88029909	-1.85556436	0.00148908
1	6.20219898	-1.64016438	-1.02411091
1	5.24199867	-2.74526453	-0.05361092
6	4.37749910	2.64743567	-0.47411090
1	5.22519875	2.04873562	-0.81641090
1	4.77039909	3.61063552	-0.14171092
1	3.73109841	2.83323550	-1.34011102
6	7.10039902	-2.15086436	0.87218910
1	7.66809893	-3.00026441	0.48608908
1	6.80219889	-2.38506436	1.89818907
1	7.77169847	-1.28816438	0.90828907

TS-56pp-H

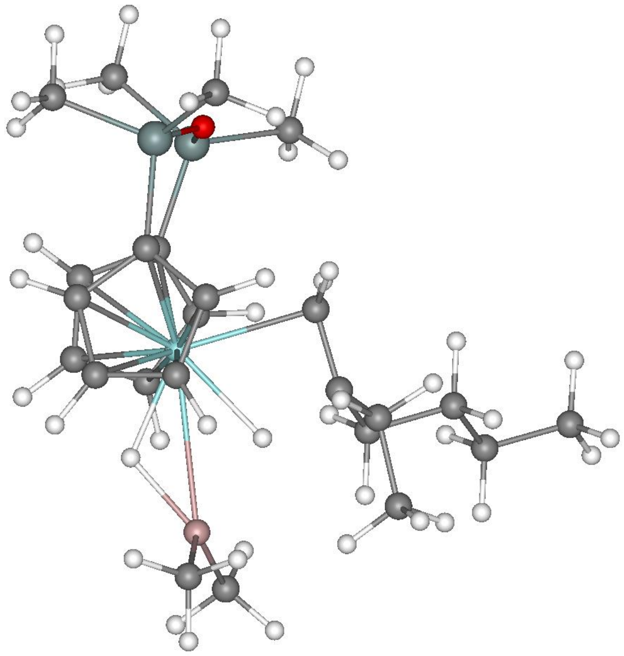

Zero-point vibrational energy	1631429.0 (Joules/Mol)
	389.92090 (Kcal/Mol)
Zero-point correction=	0.621379 (Hartree/Particle)
Thermal correction to Energy=	0.660216
Thermal correction to Enthalpy=	0.661160
Thermal correction to Gibbs Free Energy=	0.551439
Sum of electronic and zero-point Energies=	-5377.317577
Sum of electronic and thermal Energies=	-5377.278739
Sum of electronic and thermal Enthalpies=	-5377.277795
Sum of electronic and thermal Free Energies=	-5377.387516

cartesian

1	0.48367962	-2.34798765	1.62928629
1	1.43927956	-1.06368756	0.04918627
13	2.09577966	-2.52988768	1.13368630
6	3.40107965	-1.67718756	2.29888630
6	2.33127975	-4.17398787	0.11108626
1	4.40227938	-1.70708764	1.85338628
1	3.18197966	-0.63238764	2.53848624
1	3.47447968	-2.21758771	3.25068641
1	1.48697960	-4.86268806	0.22678627
1	2.46137977	-4.00498772	-0.96351379
1	3.22707963	-4.70638800	0.44938624
6	0.22667965	0.70341241	-0.93541378
6	1.54097962	0.16691238	-0.85471374
6	2.56657982	0.92221236	-0.02291374
6	2.08037972	-0.56798762	-2.08061361
1	-0.30412036	0.60231233	-1.87701368
1	0.06247965	1.65251243	-0.43581372
1	3.40667963	0.27231237	0.24118626
1	2.11657977	1.25331235	0.91918623
1	2.11317968	0.18301237	-2.87911367
1	1.34897959	-1.31438756	-2.40421367
6	-1.31722033	-3.35308766	0.05118627
6	-2.47012019	-2.55768752	0.20388627
6	-2.50922036	-1.56858754	-0.83551377
6	-1.36542034	-1.81948757	-1.64841366
6	-0.61462033	-2.88258767	-1.09601367
1	0.28887966	-3.31818748	-1.50631368
1	-1.02522039	-4.18398762	0.68118626
14	-3.87042022	-0.31098762	-1.19431365
1	-1.11672032	-1.28858757	-2.55921364
6	-0.01552036	0.04511237	2.88018632
6	-1.11542034	-0.84278762	3.06928635
6	-2.21862030	-0.30688763	2.37238622
6	-1.82512033	0.89411235	1.70298636
6	-0.45992035	1.10631239	2.06528616
1	0.13797966	1.95941234	1.76598632
1	0.96987963	-0.06678762	3.31768632
1	-1.10992038	-1.74578762	3.66718626
14	-2.96552038	2.00431252	0.69178623
6	-2.08852029	3.58141232	0.23978627
6	-4.50622034	2.31171250	1.68998635
8	-3.34562016	1.17901242	-0.69481373
1	-5.24382067	2.86801243	1.10308623
1	-4.28272057	2.89801240	2.58678627
1	-4.98062038	1.38111246	2.01748633
1	-2.80252028	4.29041195	-0.19101374
1	-1.30392039	3.42971230	-0.50711375
1	-1.64432037	4.06321239	1.11638629
6	-4.14002037	-0.23738763	-3.02901363
6	-5.38722038	-0.81998765	-0.23721375
1	-6.18072033	-0.07428762	-0.35051376
1	-5.19592047	-0.92558765	0.83638626
1	-5.78032064	-1.77688754	-0.59511375
1	-4.94122028	0.46691236	-3.27191377
1	-4.41582060	-1.21488762	-3.43541360
1	-3.23992038	0.10411239	-3.54951358
1	-3.21892023	-2.69258761	0.97568625
1	-3.21322036	-0.73848760	2.35398626
40	-0.55222034	-0.99968767	0.61548626
6	3.09267974	2.14551234	-0.79021376
1	3.51407981	1.83321238	-1.75371373
1	2.26067972	2.82191229	-1.02021372
6	4.16447926	2.89141250	0.00598627
1	4.98827934	2.20471239	0.23148626
1	3.74817967	3.20511246	0.97018623
6	3.46457982	-1.19388759	-1.92911363
1	4.24177933	-0.43548763	-1.80731368
1	3.71247983	-1.78148758	-2.81531358
1	3.52777982	-1.86998761	-1.06931365
6	4.69897938	4.10851240	-0.74691373
1	5.46827936	4.62431240	-0.16861372
1	3.89917970	4.82491207	-0.95451373
1	5.14097929	3.81381249	-1.70301366

TS-56pp-Cl

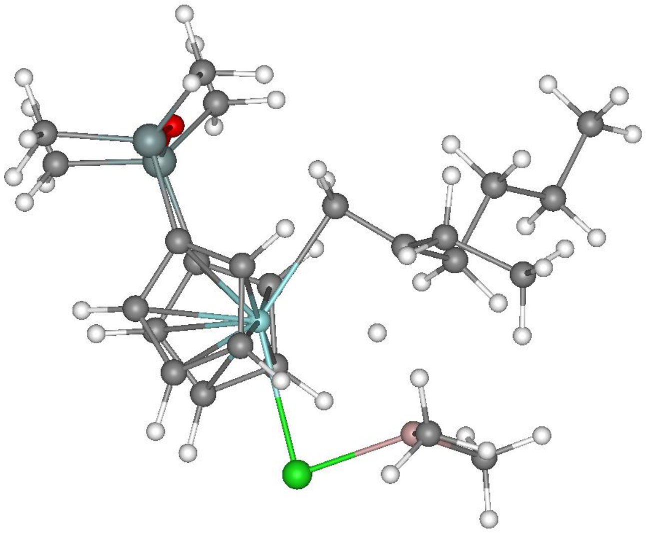

Zero-point vibrational energy	1615776.4 (Joules/Mol)
	386.17982 (Kcal/Mol)
Zero-point correction=	0.615417 (Hartree/Particle)
Thermal correction to Energy=	0.655394
Thermal correction to Enthalpy=	0.656338
Thermal correction to Gibbs Free Energy=	0.545834
Sum of electronic and zero-point Energies=	-5836.935152
Sum of electronic and thermal Energies=	-5836.895175
Sum of electronic and thermal Enthalpies=	-5836.894231
Sum of electronic and thermal Free Energies=	-5837.004735

cartesian

17	1.28919458	-3.34624791	1.68176997
1	1.56779456	-1.03974795	0.01486991
13	2.87539458	-2.40344810	0.43456993
6	4.15219450	-1.47024798	1.56476998
6	3.14849448	-3.40174794	-1.21833003
1	4.92709446	-0.93944800	1.00196993
1	3.68459463	-0.75334799	2.24766994
1	4.66899443	-2.20694804	2.19226980
1	2.83529449	-4.44564819	-1.10493004
1	2.61439443	-2.99344802	-2.08352995
1	4.21099472	-3.41354799	-1.48683012
6	-0.01660550	0.59645212	-0.66623008
6	1.38709450	0.37475201	-0.70433009
6	2.27239442	1.18505204	0.22326992
6	1.99089456	-0.01384795	-2.05523014
1	-0.54490554	0.50235200	-1.61003006
1	-0.36600551	1.42775202	-0.06253009
1	3.27679443	0.75645208	0.26936993
1	1.87959456	1.17125201	1.24396992
1	1.65959454	0.76525211	-2.75213003
1	1.52669454	-0.94024795	-2.40473008
6	-1.38960552	-3.60934782	-0.03803007
6	-2.46780539	-2.69954801	-0.04343008
6	-2.25770545	-1.71284795	-1.05473006
6	-1.04500556	-2.08844805	-1.71203005
6	-0.50320554	-3.23084784	-1.08743012
1	0.40979448	-3.74674797	-1.36233008
1	-1.26460552	-4.45574808	0.62606990
14	-3.44500542	-0.31084794	-1.49463010
1	-0.61440551	-1.57474792	-2.56413007
6	0.12039450	-0.53434795	2.98696995
6	-0.83100545	-1.58464789	3.15036988
6	-2.04670548	-1.15344799	2.58676982
6	-1.87720549	0.15215203	2.02386999
6	-0.53390551	0.52825212	2.33206987
1	-0.09050551	1.48935211	2.09996986
1	1.14609456	-0.54914796	3.33836985
1	-0.65160549	-2.53484797	3.63716984
14	-3.19380546	1.23585212	1.21346986
6	-2.71300554	3.01455212	1.45206988
6	-4.84110546	0.79275203	1.95806992
8	-3.20170546	0.92275202	-0.41443008
1	-5.65170527	1.32635212	1.45206988
1	-4.88030529	1.05495203	3.01996994
1	-5.05910540	-0.27754796	1.87706995
1	-3.47480559	3.67855191	1.03276992
1	-1.77100551	3.25085211	0.94586992
1	-2.59960556	3.26305199	2.51156998
6	-3.06110549	0.30445203	-3.20592999
6	-5.18120527	-0.96434796	-1.33743012
1	-5.91130543	-0.16164795	-1.48103011
1	-5.37350559	-1.40664792	-0.35463005
1	-5.38120556	-1.73554790	-2.08783007
1	-3.77120543	1.08655202	-3.49183011
1	-3.14300537	-0.49954796	-3.94392991
1	-2.06010556	0.73885202	-3.28833008
1	-3.33010554	-2.75514793	0.60976994
1	-2.96820545	-1.72204792	2.59616995
40	-0.42700550	-1.39474797	0.65946990
6	2.35949445	2.64035201	-0.27043009
1	2.63299441	2.66505218	-1.33273005
1	1.37209451	3.11225200	-0.19733007
6	3.37979460	3.44865203	0.53246993
1	4.36769438	2.98585200	0.42846990
1	3.12429452	3.39985204	1.59736991
6	3.51349449	-0.11694795	-2.12683010
1	3.99069452	0.85435212	-1.97263002
1	3.81139469	-0.47984797	-3.11283016
1	3.93379450	-0.80904794	-1.39283001
6	3.44129443	4.90685177	0.08126992
1	4.17869473	5.46765184	0.65916991
1	2.47229457	5.39805174	0.20766991
1	3.71809435	4.97905207	-0.97433007

TS-56pp-c-H

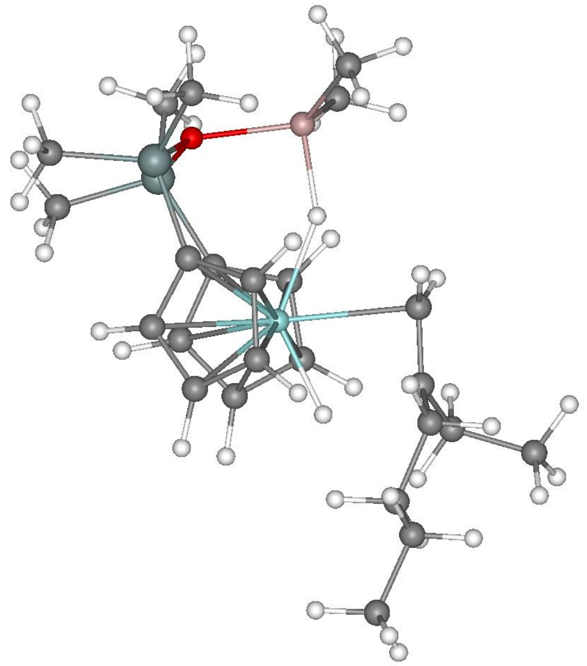

Zero-point vibrational energy	1641100.6 (Joules/Mol)
	392.23245 (Kcal/Mol)
Zero-point correction=	0.625062 (Hartree/Particle)
Thermal correction to Energy=	0.661976
Thermal correction to Enthalpy=	0.662920
Thermal correction to Gibbs Free Energy=	0.559885
Sum of electronic and zero-point Energies=	-5377.317384
Sum of electronic and thermal Energies=	-5377.280470
Sum of electronic and thermal Enthalpies=	-5377.279526
Sum of electronic and thermal Free Energies=	-5377.382562

cartesian

1	0.89587384	-0.56777269	-1.11625075
1	-1.97882617	-0.33747271	0.90374929
13	2.42257404	-0.82597268	-1.78255069
6	2.94927406	-2.70767260	-1.68265069
6	2.40647364	-0.07907270	-3.58665061
1	2.17667389	-3.27487254	-2.22145057
1	3.86937380	-2.84607267	-2.26525068
1	3.10627365	-3.21257257	-0.72855073
1	1.59367383	0.62262732	-3.79745054
1	3.34177399	0.39362729	-3.90135050
1	2.25807381	-0.93537265	-4.25765085
6	-1.44332612	-1.14907277	-1.45695078
6	-2.58472633	-1.04617274	-0.67625076
6	-3.16102600	-2.30177259	-0.03675072
6	-3.57462597	0.06722730	-0.96625078
1	-1.25942612	-0.41537270	-2.23915052
1	-0.96162617	-2.11737251	-1.57925069
1	-3.46832609	-2.11377263	0.99564928
1	-2.38802624	-3.07297254	-0.01965071
1	-4.26022625	-0.35267270	-1.71435070
1	-3.06482601	0.88502735	-1.48415077
6	-0.81202614	2.14142728	1.44964921
6	0.59697390	2.07272744	1.48574924
6	1.10297394	2.01182723	0.15294927
6	-0.04372615	2.09172750	-0.70955074
6	-1.20332611	2.17922735	0.08354928
1	-2.21992636	2.28272748	-0.26995075
1	-1.47582614	2.20332742	2.30334949
14	2.88877392	1.95112729	-0.36815071
1	-0.01722613	2.09222746	-1.79415071
6	-0.26772612	-2.43597269	1.70244932
6	-0.24912614	-1.43307269	2.71024942
6	0.99977386	-0.78557265	2.63434935
6	1.76087391	-1.33037269	1.54664922
6	0.94627386	-2.37977266	0.99814928
1	1.20787382	-3.02027273	0.16624928
1	-1.07102609	-3.13807273	1.53294921

1	-1.02982616	-1.24217272	3.43574929
14	3.47737408	-0.71227264	1.13084924
40	-0.17752612	-0.14777270	0.49744928
6	4.81277370	-1.97107267	0.82184929
6	4.00587368	0.33702731	2.57634950
8	3.28677368	0.23172730	-0.35395074
1	4.97457361	0.81732732	2.41894937
1	4.13757372	-0.37417269	3.40274930
1	3.29727364	1.09202731	2.92044950
1	5.68987370	-1.70647275	1.42104924
1	5.12397385	-2.01437259	-0.22345071
1	4.50157356	-2.97547269	1.12124932
6	3.13397408	2.62072730	-2.08045053
6	4.01137400	2.89192748	0.77904928
1	5.04647350	2.54472733	0.70624924
1	3.72027397	2.89512730	1.82904923
1	4.00327396	3.93422747	0.44274926
1	4.08277369	2.28522730	-2.50945067
1	3.18567371	3.71172738	-1.99345076
1	2.34147406	2.38762736	-2.79035068
1	1.19467378	2.08592749	2.38904929
1	1.32907391	-0.00557270	3.30834937
6	-4.35352612	-2.83857250	-0.84265071
1	-5.21922636	-2.17487264	-0.78505075
1	-4.65632629	-3.80967259	-0.44625074
1	-4.08952618	-2.97027254	-1.89575064
6	-4.39722633	0.58972734	0.21024927
1	-4.93432617	-0.23957270	0.68754929
1	-3.72922611	0.99872732	0.97924924
6	-5.40222645	1.65882730	-0.22035073
1	-6.08442640	1.23642731	-0.96665072
1	-4.87042618	2.47782731	-0.72105074
6	-6.20382643	2.21172738	0.95634925
1	-6.76372623	1.41632724	1.45644927
1	-6.91922617	2.96872735	0.62834924
1	-5.54472637	2.67292738	1.69814932

TS-56pp-c-Cl

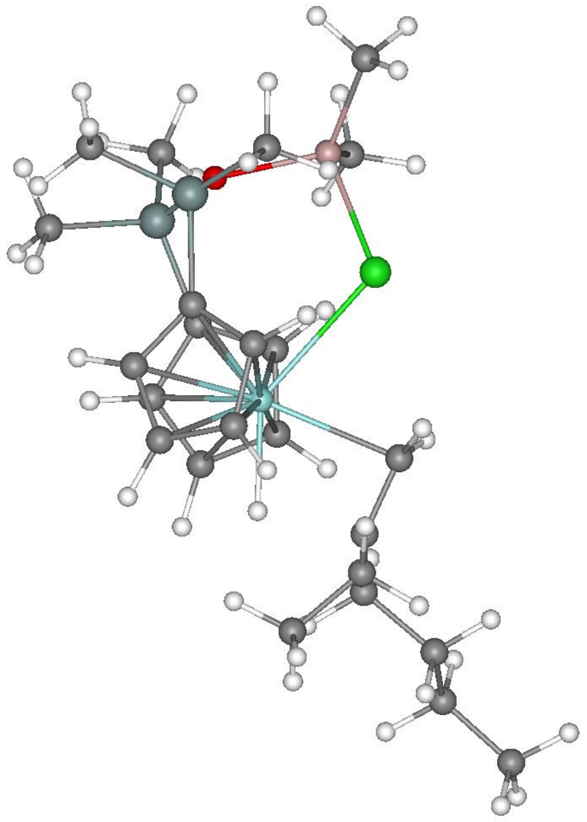

Zero-point vibrational energy	1623671.8 (Joules/Mol)
	388.06688 (Kcal/Mol)
Zero-point correction=	0.618424 (Hartree/Particle)
Thermal correction to Energy=	0.657070
Thermal correction to Enthalpy=	0.658014
Thermal correction to Gibbs Free Energy=	0.549742
Sum of electronic and zero-point Energies=	-5836.953483
Sum of electronic and thermal Energies=	-5836.914837
Sum of electronic and thermal Enthalpies=	-5836.913892
Sum of electronic and thermal Free Energies=	-5837.022164

cartesian

17	1.06137526	-0.67109871	-2.05034256
1	-1.92392468	0.94260132	0.78095758
13	3.15067530	-1.52419865	-1.61384249
6	2.92437530	-3.38609862	-1.06804252
6	4.42727518	-1.20479870	-3.04784250
1	2.36897516	-3.86139870	-1.88924253
1	3.90387535	-3.87959862	-1.05024242
1	2.41497540	-3.66799879	-0.14624242
1	4.08397532	-0.54779869	-3.85094237
1	5.38377523	-0.81029868	-2.68534255
1	4.65207529	-2.17499876	-3.50724244
6	-1.95242476	-0.13469866	-1.48054242
6	-2.91302466	0.44660133	-0.66264242
6	-3.85112476	-0.41659868	0.16265757
6	-3.44662476	1.81830132	-1.03064251
1	-1.59622478	0.41570133	-2.35074258
1	-1.85932469	-1.21849871	-1.52854252
1	-4.02642488	0.02470133	1.14875758
1	-3.40172458	-1.39809871	0.31945759
1	-4.29532480	1.61220133	-1.69504249
1	-2.71952462	2.33860135	-1.65654242
6	-0.17822474	2.86160135	1.04115748
6	1.10417533	2.32090139	1.25715756
6	1.70907521	2.00470138	0.00015758
6	0.76727527	2.43220139	-0.99824244
6	-0.37342471	2.94890141	-0.36744240
1	-1.23652482	3.37900138	-0.85524243
1	-0.86352473	3.20520139	1.80585754
14	3.42957544	1.41930127	-0.41544241
1	0.90867525	2.34830141	-2.07014251
6	-0.94342476	-1.80419874	1.28785753
6	-0.94262475	-0.82359868	2.32155752
6	0.39477527	-0.40939867	2.50355744
6	1.24187529	-1.10319865	1.57215750
6	0.37727529	-1.98009872	0.84595758
1	0.66587532	-2.64759874	0.04505758
1	-1.79612470	-2.36319876	0.93035758

1	-1.79852462	-0.49919865	2.89905763
14	3.09987545	-0.95039868	1.50515747
40	-0.22722471	0.43100134	0.23515756
6	4.06747532	-2.52139878	1.74695754
6	3.65507531	0.23990133	2.82505751
8	3.46197534	-0.31569865	-0.09264243
1	4.74197531	0.36450133	2.80505753
1	3.41317534	-0.23509866	3.78425741
1	3.20547533	1.23200130	2.83505750
1	4.58437538	-2.46099877	2.71035743
1	4.83097553	-2.65569878	0.97615761
1	3.44487524	-3.41689873	1.76135755
6	3.72517538	1.75880134	-2.21364236
6	4.77147532	2.22910142	0.58845758
1	5.64007521	1.57150126	0.69485760
1	4.46647549	2.56160140	1.58135748
1	5.10717535	3.11530137	0.03915758
1	4.73197556	1.47570133	-2.52944255
1	3.64617538	2.84770131	-2.32744241
1	3.00677538	1.30290127	-2.89764237
1	1.54837537	2.18840122	2.23365760
1	0.72687531	0.29820132	3.25365758
6	-5.19932461	-0.61969870	-0.55044240
1	-5.75512457	0.32450134	-0.58314240
1	-5.02392483	-0.92299867	-1.59014249
6	-6.05292463	-1.67539871	0.15415758
1	-6.19582462	-1.38509870	1.20145750
1	-5.51132488	-2.62829876	0.16555758
6	-3.92302465	2.69340134	0.12515758
1	-4.72842455	2.21130133	0.68585759
1	-4.30912447	3.64300132	-0.25114241
1	-3.11042476	2.90650129	0.82575756
6	-7.41112471	-1.86399865	-0.51924241
1	-8.00052547	-2.62959862	-0.01024242
1	-7.29172468	-2.17129874	-1.56204247
1	-7.98762465	-0.93489867	-0.50764245

S2. NMR spectra of 2'

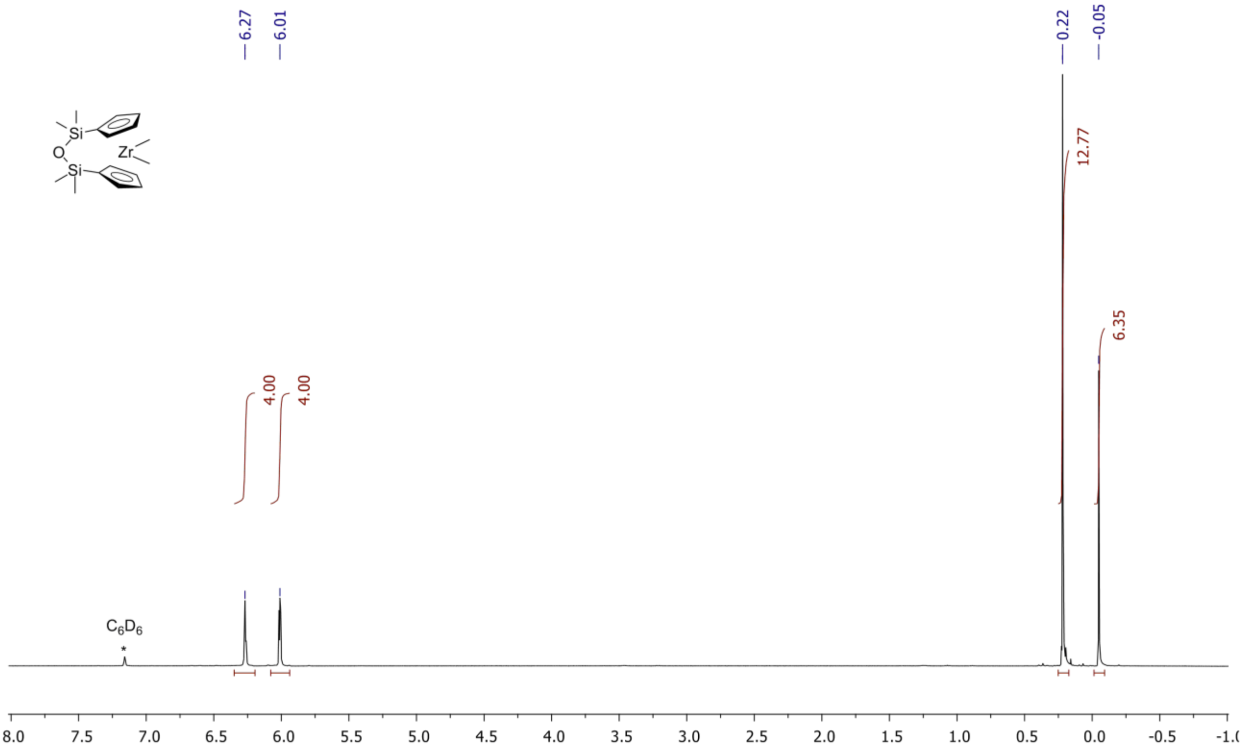

Figure S1. ^{1}H NMR spectrum (C_6D_6, 400 MHz, 20 °C) of **2'**.

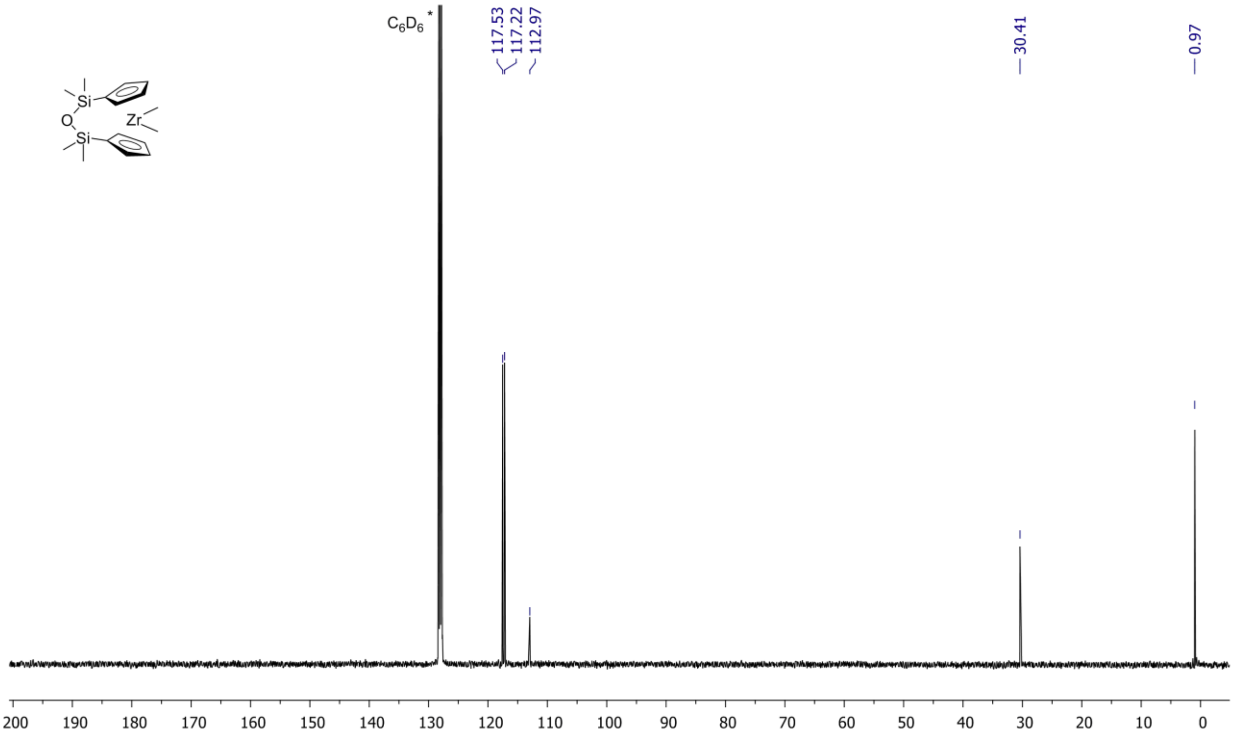

Figure S2. ^{13}C NMR spectrum (C_6D_6, 101 MHz, 20 °C) of **2'**.
